# Rational design, synthesis, molecular modeling, biological activity, and mechanism of action of polypharmacological norfloxacin hydroxamic acid derivatives†

**DOI:** 10.1039/d3md00309d

**Published:** 2023-09-19

**Authors:** Ahmed M. Kamal El-sagheir, Ireny Abdelmesseh Nekhala, Mohammed K. Abd El-Gaber, Ahmed S. Aboraia, Jonatan Persson, Ann-Britt Schäfer, Michaela Wenzel, Farghaly A. Omar

**Affiliations:** a Medicinal Chemistry Department, Faculty of Pharmacy, Assiut University Assiut 71526 Egypt farghalyomar@pharm.aun.edu.eg; b Division of Chemical Biology, Department of Life Sciences, Chalmers University of Technology 412 96 Gothenburg Sweden wenzelm@chalmers.se; c Center for Antibiotic Resistance Research in Gothenburg (CARe) Gothenburg Sweden

## Abstract

Fluoroquinolones are broad-spectrum antibiotics that target gyrase and topoisomerase IV, involved in DNA compaction and segregation. We synthesized 28 novel norfloxacin hydroxamic acid derivatives with additional metal-chelating and hydrophobic pharmacophores, designed to enable interactions with additional drug targets. Several compounds showed equal or better activity than norfloxacin against Gram-positive, Gram-negative, and mycobacteria, with MICs as low as 0.18 μM. The most interesting derivatives were selected for *in silico*, *in vitro*, and *in vivo* mode of action studies. Molecular docking, enzyme inhibition, and bacterial cytological profiling confirmed inhibition of gyrase and topoisomerase IV for all except two tested derivatives (10f and 11f). Further phenotypic analysis revealed polypharmacological effects on peptidoglycan synthesis for four derivatives (16a, 17a, 17b, 20b). Interestingly, compounds 17a, 17b, and 20b, showed never seen before effects on cell wall synthetic enzymes, including MreB, MurG, and PonA, suggesting a novel mechanism of action, possibly impairing the lipid II cycle.

## Abstract 2

Addition of metal-chelating and lipophilic groups to norfloxacin yielded dual-action compounds inhibiting DNA gyrase/topoisomerase IV and bacterial cell wall synthesis.

## Graphical abstract

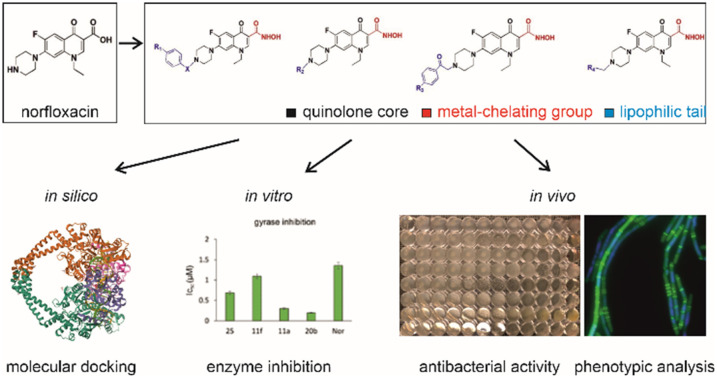

## Introduction

Multidrug-resistant bacteria have become a major health concern around the globe.^1,2^ Most prominently, the problem of methicillin-resistant *Staphylococcus aureus* (MRSA), which has the propensity to accumulate multiple resistances and is a common nosocomial pathogen, has been widely discussed.^3^ Even more problematic are Gram-negative bacteria, such as *Escherichia coli*, *Klebsiella pneumoniae*, and *Pseudomonas aeruginosa*, which are protected by their impermeable outer membrane. Several cases of untreatable Gram-negative infections have been reported.^4,5^ Mycobacteria, the causative agent of severe diseases like tuberculosis and leprosy, are evolutionarily closer to Gram-positive bacteria but possess an impermeable mycolic acid layer, which is analogous to the Gram-negative outer membrane. Tuberculosis is the second leading cause of death by a single infectious agent, only recently being surpassed by SARS-CoV-2 (https://www.who.int/teams/global-tuberculosis-programme/tb-reports/global-tuberculosis-report-2022).

Broad-spectrum antibiotics that are active against all three of these classes of pathogens are uncommon, one being fluoroquinolones. Fluoroquinolones are among the most prescribed antibiotic classes,^6^ mostly due to their good oral bioavailability and relatively rare side effects.^7^ They inhibit two bacterial enzymes involved in DNA packing, gyrase and topoisomerase IV. Gyrase controls DNA supercoiling and relieves topological stress, while topoisomerase IV is a decatenating enzyme that resolves interlinked daughter chromosomes following DNA replication.^8^ In Gram-negative bacteria, the primary target of fluoroquinolones is DNA gyrase, while DNA topoisomerase IV serves as secondary target. In Gram-positive bacteria, this is reversed.^9^ Bactericidal activity is mediated by the formation of a non-functional ternary complex of the drug with the targeted enzymes and DNA, blocking DNA replication and cell division.^10^ Unfortunately, clinical resistance to fluoroquinolones can develop by simple point mutations, resulting in comparably high resistance development despite their dual targets.^11^

Fluoroquinolones are synthetic drugs and thus easily accessible to chemical modification. Almost any rest group of the quinolone ring can be modified, resulting in a variety of reported derivatives.^12,13^ An interesting approach are fluoroquinolone hybrid molecules designed to possess more than one pharmacophore allowing inhibition of additional targets.^14^ This is known as polypharmacology and is an emerging strategy in antibacterial drug design, promising lower resistance development than classical single-target drugs.^15^ The easily modifiable structure of fluoroquinolones ideally lends itself to this approach (see Text S1 and Fig. S1† for similar fluoroquinolone derivatives from other studies).

Here, we aimed at designing and synthesizing norfloxacin derivatives with secondary targets in bacterial cell envelope synthesis. The cell envelope, in particular the peptidoglycan cell wall, is the most successful clinical antibiotic target,^16^ yet its full potential has not been exploited by far. Many cell envelope targets remain to be explored, among them the proteins LpxC and NagA, which we aimed at in this study.

The UDP-3-*O*-(*R*-3-hydroxymyristoyl)-*N*-acetylglucosamine deacetylase LpxC is the key enzyme in the synthesis of lipid A, the lipid anchor of lipopolysaccharides, which decorate the outer membrane of Gram-negative bacteria and confer its impermeability. Imbalances in lipid A levels are detrimental and both the deletion and overexpression of *lpxC* are lethal.^17^ LpxC is highly conserved among Gram-negative bacteria and does not have a known homologue in humans, making it an interesting potential drug target.^18,19^ Several LpxC inhibitors have been identified and characterized, yet none have made it to clinical application so far.^20,21^

The *N*-acetylglucosamine-6-phosphate deacetylase NagA catalyzes the deacetylation of *N*-acetylglucosamine-6-phosphate (GlcNAc6P) to glucosamine-6-phosphate (GlcN6P). It is involved in the turnover and recycling of cell wall components. NagA is conserved in Gram-positive, Gram-negative, and mycobacteria, yet it appears to be essential only in the latter. It has been put forward as potential drug target in *M. tuberculosis* for the crucial role of glucosamine derivatives in peptidoglycan synthesis,^22^ yet no inhibitors have so far been described.

**Fig. 1:**
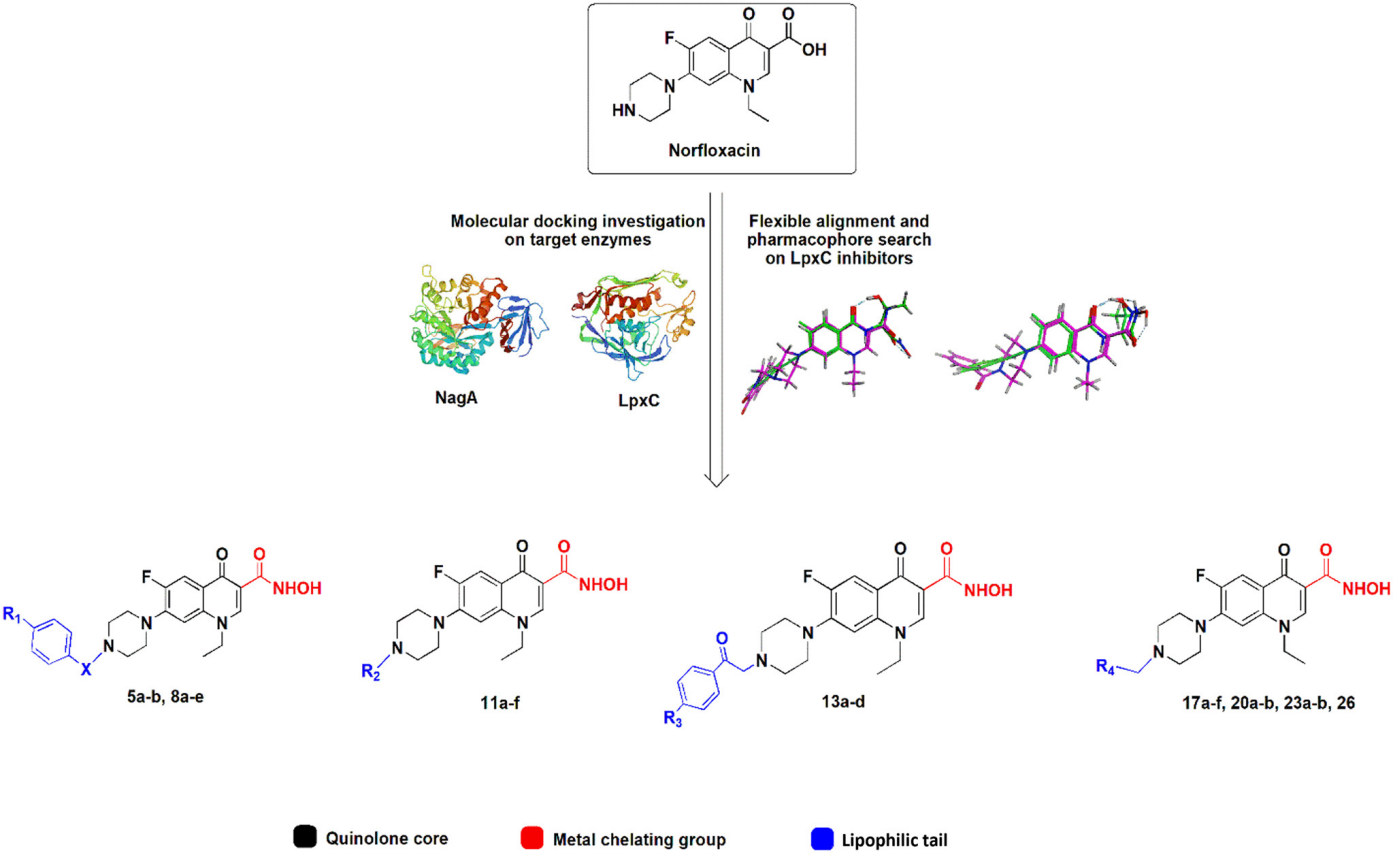
Design of norfloxacin derivatives with multiple pharmacophores.

The active center of LpxC contains a catalytic zinc ion and a hydrophobic tunnel accommodating a myristate fatty acid side chain. Similarly, the active center of NagA contains both a Cd^2+^ and Zn^2+^ ion. Most known LpxC inhibitors share a hydroxamate head group, capable of binding zinc, and a lipophilic tail, occupying the hydrophobic tunnel, as common features (Fig. S2†).^23,24^ Here, we incorporated these features into the norfloxacin lead structure, creating hybrid molecules with a quinolone core, a hydroxamic acid metal-chelating group, and a hydrophobic tail (Fig. 1), aiming at creating polypharmacological norfloxacin derivatives that retain their activity against gyrase and topoisomerase IV, but can also interact with additional targets through metal chelation and hydrophobic interactions for example, LpxC and NagA.

Fifty-six norfloxacin derivatives, including 28 novel hydroxamic acid derivatives, were tested for their activity against Gram-negative, Gram-positive, and mycobacterial test strains. The most promising compounds were selected for mode of action studies, comprising *in silico* (molecular docking), *in vitro* (enzyme activity and metal chelation), and *in vivo* (phenotypic analysis) experiments. Together, these data give information about the effects of the structural modifications on the activity and polypharmacological mechanisms of the synthesized norfloxacin derivatives.

## Results and discussion

### Chemical synthesis and characterization

**Scheme 1:**
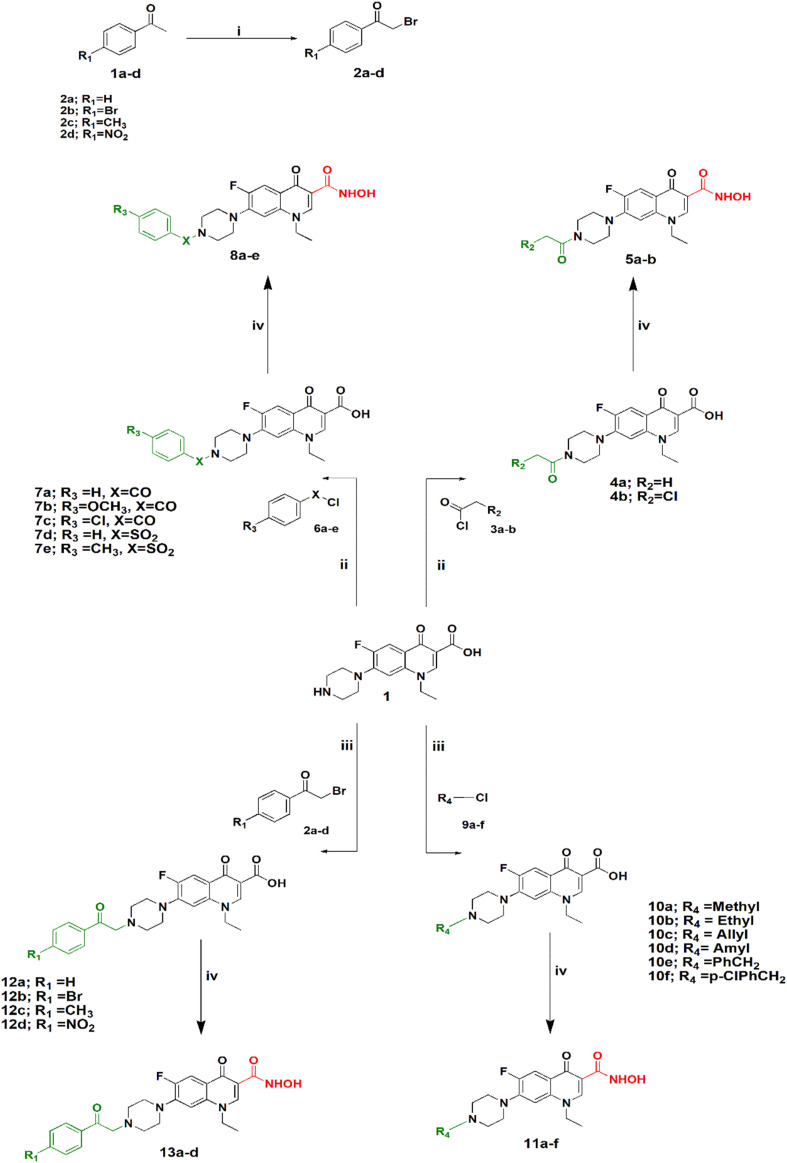
Synthesis of *N*4-substiuted piperazinyl norfloxacin hydroxamic acid derivatives. Reagents and conditions: (i) NBS, PTSA, CH_3_CN, reflux. (ii) THF, Et_3_N, reflux. (iii) CH_3_CN, K_2_CO_3_, KI, reflux. (iv) DCM, Et_3_N, ClCOOEt, NH_2_OH·HCl.

**Scheme 2:**
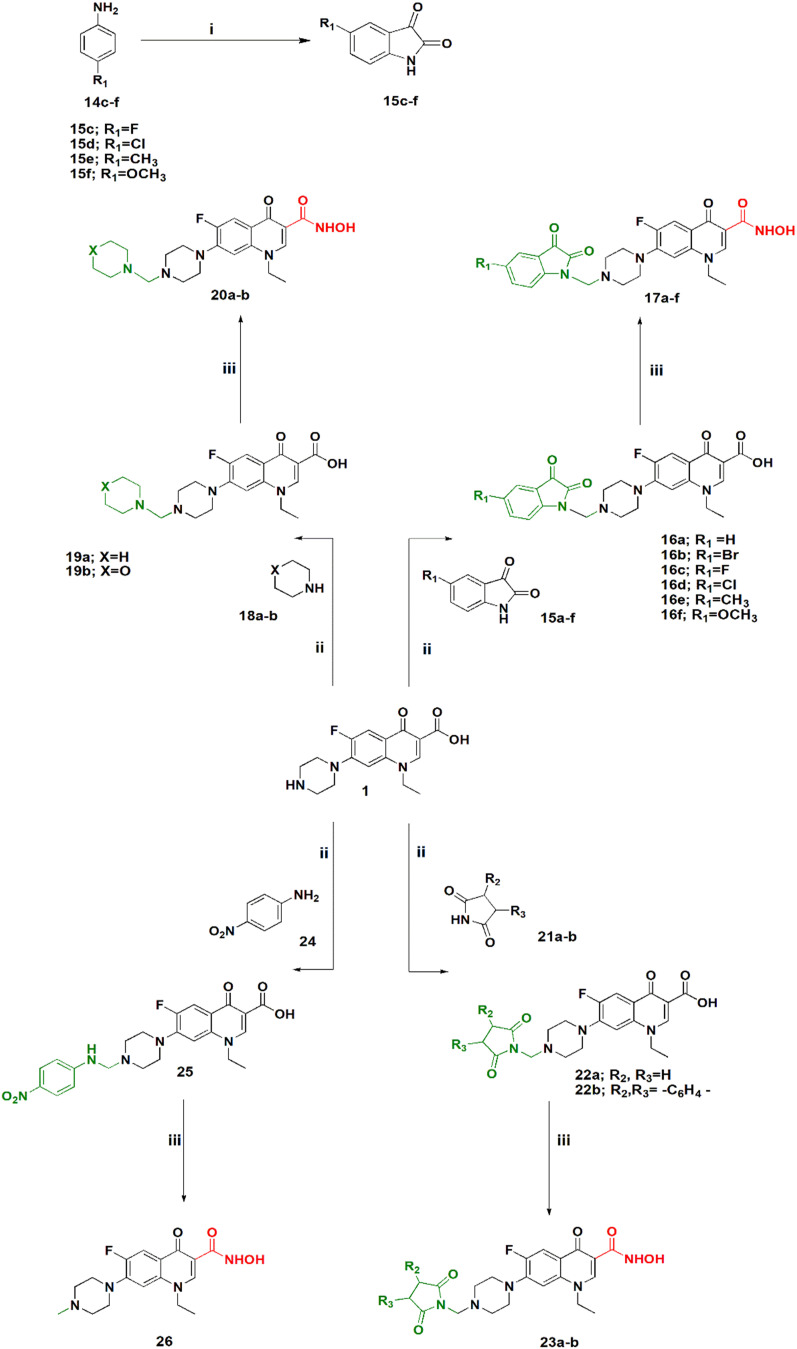
Synthesis of hydroxamic acid norfloxacin Mannich base derivatives. Reagents and conditions: (i) chloral hydrate, Na_2_SO_4_, H_2_SO_4_, 60–80 °C. (ii) EtOH, HCHO, reflux. (iii) DCM, Et_3_N, ClCOOEt, NH_2_OH·HCl.

Synthesis of the target compounds is outlined in Schemes 1 and 2. In Scheme 1, preparation of a series of hydroxamic acids of *N*-acyl, sulphonyl, alkyl and phenacylpiperazinyl derivatives of norfloxacin is depicted. Different acyl, sulphonyl, alkyl, or phenacyl norfloxacin derivatives 4–12 were synthesized as reported in the literature.^25,26^ Hydroxamic acid derivatives 5–13 were synthesized by reaction of *N*4-substituted piperazinyl norfloxacin derivatives with ethyl chloroformate in the presence of triethylamine in dichloromethane to afford a mixed anhydride, which then interacted with added hydroxylamine hydrochloride to afford hydroxamic acid in 53–84% yield. The final product was purified through crystallization with methanol and the obtained solid was triturated with diethyl ether. Different phenacylbromides 2a–d were synthesized in 59–75% yield as reported previously.^27^ All compounds were confirmed through determination of melting points as well as ^1^H NMR analysis. Newly synthesized compounds were identified by IR, ^1^H NMR, ^13^C NMR, mass spectra, and elemental analysis. The IR spectra of compounds 5a–b, 8a–e showed absorption bands at 3400 cm^−1^, 3180 cm^−1^, 1670 cm^−1^, 1640 cm^−1^, and 1615 cm^−1^ attributed to the NH, OH, hydroxamic C=O, amidic C=O, and quinolone C=O, respectively. IR spectra of compounds 11a–f showed absorption bands at 3400 cm^−1^, 3170 cm^−1^, 1680 cm^−1^, and 1630 cm^−1^ attributed to the NH, OH, hydroxamic C=O, and quinolone C=O, respectively. IR spectra of compounds 13a–d showed absorption bands at 3400 cm^−1^, 3160 cm^−1^, 1700, 1650 cm^−1^, and 1640 cm^−1^ attributed to the NH, OH, ketonic C=O, hydroxamic C=O, and quinolone C=O, respectively.

Scheme 2 shows the synthesis of 5-substituted indoline-2,3-dione derivatives 15c–f according to the literature^28^ in 65–70% yield. Their identity was confirmed by their melting points. Then, a series of hydroxamic acids of different norfloxacin Mannich bases were synthesized, whereby norfloxacin Mannich bases 16–25 were synthesized as reported through reflux of a mixture of 2 mL of formaline (37%), norfloxacin (9.39 mmol), and the respective amine (9.39 mmol) in ethanol, giving a yield of 74–90%. The obtained Mannich bases were purified through crystallization using DMF and water.^29^ Following this, hydroxamic acid derivatives 17–26 were synthesized according to the same procedure outlined in Scheme 1 in 60–80% yield. All reported Mannich bases were confirmed through determination of their melting points as well as ^1^H NMR analysis, while the newly prepared compounds were identified by their IR, ^1^H NMR, ^13^C NMR, mass spectra, and elemental analysis. The IR spectra of compounds 17a–f showed absorption bands at 3400 cm^−1^, 3150 cm^−1^, 1750 cm^−1^, 1720 cm^−1^, 1660 cm^−1^, and 1620 cm^−1^ attributed to the NH, OH, amidic C=O, ketonic C=O, hydroxamic C=O, and quinolone C=O, respectively. IR spectra of compounds 20a–b showed absorption bands at 3400 cm^−1^, 3180 cm^−1^, 1680 cm^−1^, and 1620 cm^−1^ attributed to the NH, OH, hydroxamic C=O, and quinolone C=O, respectively. IR spectra of compounds 23a–d showed absorption bands at 3400 cm^−1^, 3180 cm^−1^, 1740, 1700 cm^−1^, and 1620 cm^−1^ attributed to the NH, OH, imidic C=O, hydroxamic C=O, and quinolone C=O, respectively. In case of compound 26, the IR spectrum showed absorption bands at 3400 cm^−1^, 3140 cm^−1^, 1680 cm^−1^, and 1620 cm^−1^ attributed to the NH, OH, hydroxamic C=O, and quinolone C=O, respectively. Please see Text S2 and Fig. S3–S91† for NMR results, Fig. S92 for elemental analysis certificates, and Text S12† for detailed yields and reaction times for all compounds.

#### Biological activity

Minimal inhibitory concentrations (MICs) were determined against a panel of test strains comprising Gram-negative, Gram-positive, and mycobacteria, including a fluoroquinolone-resistant, clinical isolate of *E. coli* and an MRSA strain that is also resistant to norfloxacin and ciprofloxacin (Table 1). Several of the newly synthesized compounds showed similar or better activities than norfloxacin and ciprofloxacin. Of particular note are compounds 11a, 11d, 19a, 20b, 23a, and 25, which show nanomolar activity against *E. coli*, and 5a, 11e, and 20a, which were more active against *M. tuberculosis* than isoniazid. While activity against fluoroquinolone-resistant strains was not meeting clinical breakpoints (https://www.eucast.org/clinical_breakpoints), several compounds showed strongly increased activity when compared to norfloxacin and ciprofloxacin, suggesting that these can be used as lead structures for further improvement of their activity. This is for example the case for 7b, 8a, 16f, 17d, and 23a, which are five to six-times more active than norfloxacin against fluoroquinolone-resistant MRSA.

**Table 1:** Minimal inhibitory concentrations of norfloxacin derivatives. Activities better than norfloxacin (or isoniazid for *M. tuberculosis*) are indicated in **bold**

Compound	Gram-negative strains				Gram-positive strains			Mycobacteria
	*E. coli W3110*	*E. coli* a	*P. aeruginosa PAO1*	*K. pneumoniae ATCC10031*	*S. aureus CCUG1800T*	*MRSA* a *ATCC 43300*	*E. faecalis ATCC 19433*	*M. tuberculosis MC26020*
**INH**	—	—	—	—	—	—	—	1.82
**Nor**	0.39	50.1	6.26	9.39	3.13	100.21	4.69	1.56
**Cip**	0.37	193.17	3.01	7.54	3.01	96.58	6.03	2.26
4a	22.13	>1416.83	>1416.83	177.10	11.06	177.10	44.27	354.20
4b	>1293.54	>1293.54	>1293.54	>1293.54	161.69	646.77	>1293.54	>1293.54
5a	6.64	127.53	63.76	42.51	**2.65**	>1360.32	6.64	**1.32**
5b	9.73	>1246.25	934.69	>1246.25	**2.43**	>1246.25	7.30	>1246.25
7a	>1209.14	>1209.14	>1209.14	1209.14	75.57	604.57	113.35	>1209.14
7b	>1129.09	282.27	>1129.09	**5.51**	70.56	**17.64**	70.56	>1129.09
7c	>1118.19	>1118.19	>1118.19	279.54	69.88	>1118.19	>1118.19	>1118.19
7d	>1114.27	>1114.27	>1114.27	1114.27	69.64	1114.27	1114.27	>1114.27
7e	>1081.26	1081.26	>1081.26	>1081.26	67.57	>1081.26	>1081.26	>1081.26
8a	9.12	1167.75	291.93	18.24	**2.28**	**18.24**	**2.28**	2.28
8b	17.07	1092.89	1092.89	1092.89	**2.13**	546.44	17.07	>1092.89
8c	33.83	>1082.68	541.34	>1082.68	16.91	1082.68	**3.17**	6.34
8d	4.21	>1079	1079.00	33.71	16.85	1079.00	33.71	>1079
8e	32.75	524.02	>1048.04	262.01	32.75	16.37	>1048.04	12.28
10a	5.99	1535.87	383.96	47.99	23.99	383.96	11.99	383.96
10b	46.05	>1473.89	921.18	46.05	46.05	368.47	>1473.89	368.47
10c	5.56	>1424.63	1424.63	>1424.63	11.12	178.07	22.25	44.51
10d	6.41	1314.64	1314.64	>1314.64	20.54	328.66	82.16	328.66
10e	>1250.45	>1250.45	>1250.45	19.53	78.15	>1250.45	1250.45	>1250.45
10f	72.08	**18.02**	>1153.41	>1153.41	36.04	576.70	288.35	>1153.41
11a	**0.18**	**22.96**	45.92	45.92	**2.87**	>1469.70	8.61	2.87
11b	2.75	>1412.8	91.05	>1412.8	**2.75**	>1412.80	16.55	264.9
11c	2.67	>1357.48	64.10	85.46	**2.67**	**42.73**	10.68	10.68
11d	**0.30**	>1265.82	**2.47**	9.88	**2.47**	632.91	**3.70**	2.47
11e	2.35	>1206.21	904.65	>1206.21	**2.35**	**75.38**	**2.35**	**1.76**
11f	1.08	**34.86**	139.46	**3.26**	**2.17**	>1115.68	>1115.68	>1115.68
12a	73.14	**36.57**	>1170.39	18.28	146.29	>1170.39	>1170.39	>1170.39
12b	4.84	>991.55	>991.55	991.55	30.98	>991.55	23.23	>991.55
12c	70.87	1134.02	1134.02	**6.64**	141.75	**35.43**	1134.02	1134.02
12d	5.18	>1061.22	1061.22	>1061.22	16.58	**66.32**	33.16	>1061.22
13a	2.21	**17.68**	212.16	>1131.54	17.68	282.88	**2.21**	141.44
13b	1.88	60.22	270.99	**5.64**	15.05	963.54	963.54	60.22
13c	5.35	274.38	617.36	274.38	8.57	>1097.53	>1097.53	>1097.53
13d	5.02	64.32	64.32	16.08	8.04	>1029.18	>1029.18	>1029.18
16a	5.22	1070.07	83.59	>1070.07	133.75	>1070.07	267.51	167.19
16b	4.48	>918.59	172.23	>918.59	14.35	**68.11**	28.70	>918.59
16c	3.02	**16.11**	17.12	**8.05**	8.05	>1031.30	**3.02**	64.45
16d	3.89	>998.2	249.55	998.20	499.10	249.55	62.38	>998.2
16e	5.07	>1039.59	16.24	**3.04**	32.48	259.89	6.09	129.94
16f	4.91	1006.88	62.93	503.44	31.46	**15.73**	251.72	>1006.88
17a	3.03	**32.42**	9.11	**5.06**	**2.02**	>1037.5	**2.02**	12.15
17b	2.62	>894.51	118.8	894.51	6.98	894.51	5.24	111.81
17c	2.93	125.12	187.69	1001.01	125.12	>1001.01	7.82	5.86
17d	4.73	>969.82	22.73	90.92	**1.89**	**15.15**	7.57	121.22
17e	7.88	252.21	189.15	**3.94**	15.76	>1008.84	>1008.84	504.42
17f	15.28	>978.01	366.75	>978.01	489	489.00	244.50	244.50
19a	**0.30**	**38.41**	96.04	**7.20**	153.66	>1229.32	>1229.32	614.66
19b	19.11	>1223.44	229.39	>1223.44	152.93	1223.44	114.69	611.72
20a	2.31	>1186.55	**4.63**	74.15	4.63	1186.55	**2.31**	**1.15**
20b	**0.28**	**18.45**	**2.30**	**6.92**	**2.30**	>1181.13	36.91	4.61
22a	18.62	74.51	447.09	298.06	74.51	1192.25	149.03	298.06
22b	16.75	>1072.29	201.05	>1072.29	268.07	>1072.29	>1072.29	402.11
23a	0.56	>1149.42	**3.36**	35.91	**2.24**	**17.95**	**3.36**	2.24
23b	5.06	259.37	8.50	>1037.50	32.42	>1037.50	259.37	97.26
25	**0.27**	**17.04**	**2.13**	**4.26**	**2.13**	**34.08**	**2.13**	6.39
26	23.92	>1531.32	191.41	>1531.32	95.7	382.83	>1531.32	382.83

a Norfloxacin-resistant strains.

#### Structure–activity relationship

Compared to norfloxacin, compounds 5a and 5b showed increased activity against *S. aureus*, suggesting that the acetyl and chloroacetyl groups increase potency against this organism, yet only in the hydroxamic acid derivatives and not in their carboxylic acid counterparts 4a and 4b. Similarly, 8a–c showed increased activity against Gram-positive strains, suggesting that the addition of bulky benzoyl groups to the *N*4-piperazinyl moiety can be beneficial. This effect was only observed for the hydroxamic acid derivatives, with the exception of 7b, which was notably more active against MRSA than norfloxacin. Several compounds of series 11 showed higher activity against Gram-negative (11a, 11d, 11f) and Gram-positive bacteria (11a–f), suggesting that hydrophobic *N*-piperazinyl substitutions increase the overall potency of the molecule. The *N*-piperazinyl piperidine-substituted compounds 19a (carboxylic acid) and 20a (hydroxamic acid) showed increased activity against Gram-negative bacteria, yet 20a was also active against Gram-positive and mycobacteria. This suggests that an *N*-piperazinyl piperidine substitution is beneficial for increased activity, whereby substitution of the carboxylic acid to hydroxamic acid increases versatility of the molecule. Similar effects were observed for the hydroxamic acid 20b, which carries a morpholinomethyl substitution at the *N*-piperazinyl moiety and showed good activity against all Gram-negative test strains as well as *S. aureus*. Interestingly, the corresponding carboxylic acid 19b lost its activity almost entirely. Compound 25, carrying a nitrophenylaminomethyl rest at the *N*-piperazidine moiety, exhibited increased activity against all Gram-negative and Gram-positive test strains, making this substitution an interesting starting point for further improvement of activity. Our results support the previous notion that substitutions at the *N*-piperazinyl moiety affect the activity spectrum and antibacterial potency including potency against fluoroquinolone-resistant strains.^13^

#### Prediction of drug likeness and cytotoxicity

Physicochemical parameters were predicted using Molecular Operating Environment (MOE) software package 2020.01^30^ and pharmacokinetic and toxicological properties were predicted using the webtools SwissADME (http://www.swissadme.ch/)^31^ and pkCSM (https://biosig.lab.uq.edu.au/pkcsm/).^32^ In essence, predictions confirmed that all compounds but 17f fulfil Lipinski's rule of five and Veber's rule of oral bioavailability and are expected to have good pharmacokinetic profiles and low toxicity. Details are described in Text S3–S5 and depicted in Tables S1–S3.† Two exemplary compounds, 8b and 20b, which were the most potent *in vitro* inhibitors of topoisomerase IV and gyrase, respectively (see below), were selected for experimental assessment of cytotoxicity against human neuroblastoma (SH-SY5Y) and human fetal lung fibroblast cell lines (WI-38). For both cell lines, compounds 8b and 20b showed IC_50_ values comparable to norfloxacin and significantly higher than that of the apoptosis-inducing kinase inhibitor staurosporine (Fig. S93†). Therapeutic windows for *E. coli*, *P. aeruginosa*, and *S. aureus* were 18 to 147-fold for 8b and 19 to 169-fold for 20b, both being larger than those of norfloxacin (7 to 124-fold) (Fig. S93†).

#### Molecular docking

The mechanisms of the new derivatives were first examined *in silico* by modeling their interactions with the intended target enzymes. Molecular docking studies were carried out on the X-ray structure of *S. aureus* DNA gyrase in complex with DNA and moxifloxacin (PDB 5cdq), the crystal structure of *Acinetobacter baumannii* topoisomerase IV in complex with DNA and moxifloxacin (PDB 2xkk), the co-crystal structure of *P. aeruginosa* LpxC with inhibitor 50432 (PDB 6mod), and the crystal structure of *Mycobacterium smegmatis* NagA mutant D267A in complex with *N*-acetyl-d-glucosamine-6-phosphate (PDB 6fv4) using MOE 2020.01. Docking experiments are described in detail in Text S6–S9† and depicted in Fig. S94–131 and Tables S4–S7.† In essence, docking experiments showed that all tested derivatives interacted with gyrase and topoisomerase in a manner similar to norfloxacin and were able to interact with NagA and LpxC *in silico*. The latter was also supported by ligand-based pharmacophore modeling suggesting that our compounds share key pharmacophores with known LpxC inhibitors (Fig. S132–S134, Tables S8 and S9, see Text S10† for details).

#### *In vitro* inhibition of DNA gyrase and topoisomerase IV

**Fig. 2:**
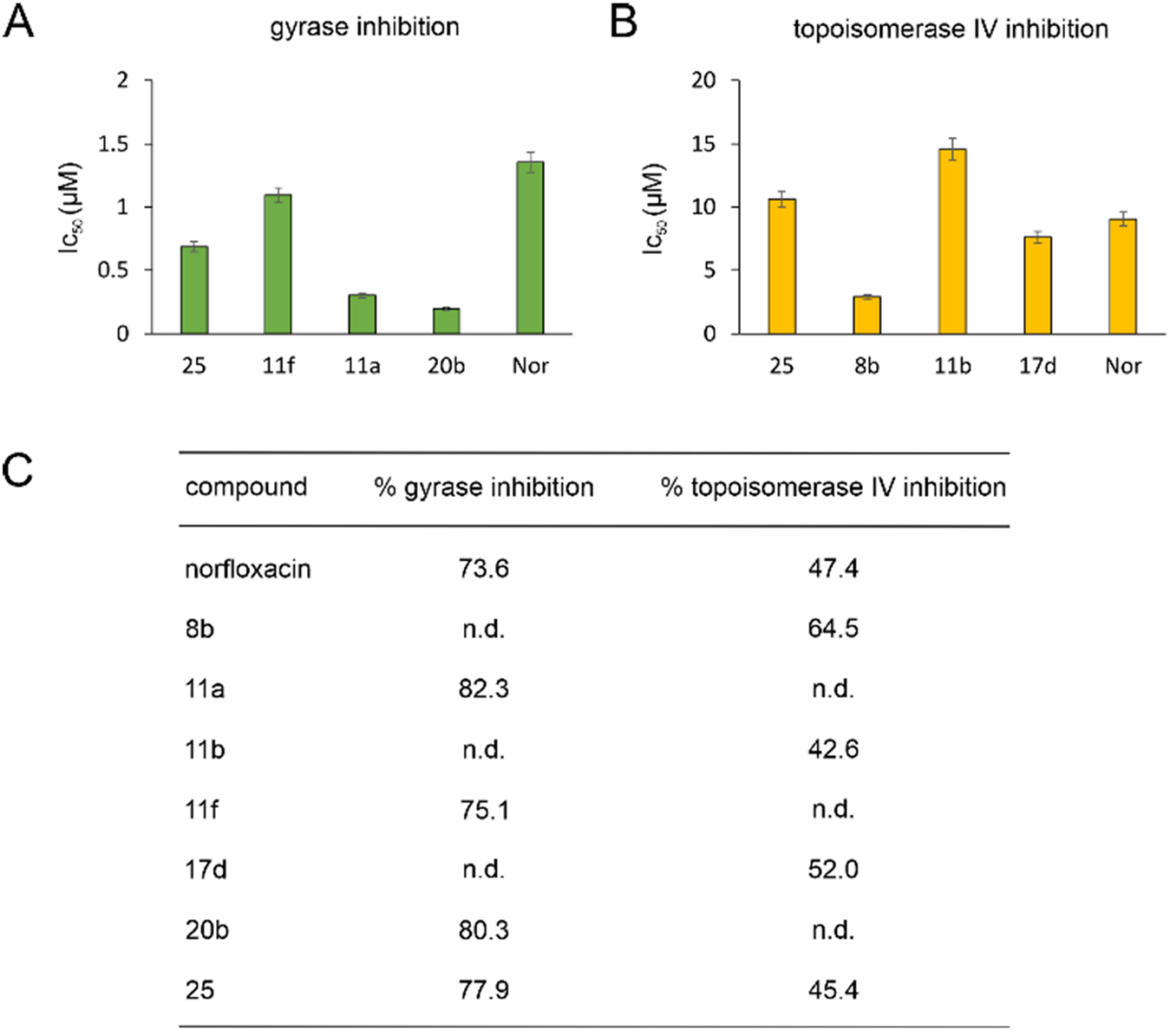
IC_50_ (A and B) and % inhibition (C) of *E. coli* DNA gyrase and topoisomerase IV. n.d.: not determined.

Derivatives 8b, 11a, 11b, 11f, 17d, 20b, and 25 were chosen for *in vitro* inhibition studies using purified *E. coli* gyrase and topoisomerase IV.^33^ All tested compounds showed activities comparable to norfloxacin. Notably, 11a and 20b were considerably more potent inhibitors of DNA gyrase than their parent compound (4.5-fold and 7-fold lower IC_50_, respectively) (Fig. 2, see also Fig. S93† for therapeutic windows).

Gyrase and topoisomerase IV contain Mg^2+^ in their active centers. Similarly, LpxC contains Zn^2+^ and NagA both Cd^2+^ and Zn^2+^. Our molecular docking experiments suggested that binding to these metals is important for the target interaction of norfloxacin and its derivatives. Therefore, we assessed the ability of the exemplary derivatives 11a, 11b, 1f, 17a, 20b, and 23a to form a complex with these metal ions using UV-vis spectroscopy (Fig. S135–S137, Table S10†). All tested compounds showed a spectral shift indicative of metal complexation. Thereby, affinity to zinc was higher than that to magnesium and cadmium, and our derivatives showed higher binding of metal ions than norfloxacin (see Text S11† for details).

#### Bacterial cytological profiling of *E. coli*

Inhibition of gyrase leads to a characteristic phenotype of extreme nucleoid condensation into an oval structure in the middle of the cell, often accompanied by cell elongation due to the cells' inability to segregate their nucleoids and, consequently, to complete cell division. This phenotype is only observed with fluoroquinolones and not with other compounds that affect bacterial DNA. Thus, it can be used to confirm gyrase inhibition *in vivo*. To this end, we used bacterial cytological profiling (BCP), a phenotypic analysis method that is based on phase contrast microscopy combined with fluorescent dyes that stain the nucleoid and cell membrane.^34,35^

**Fig. 3:**
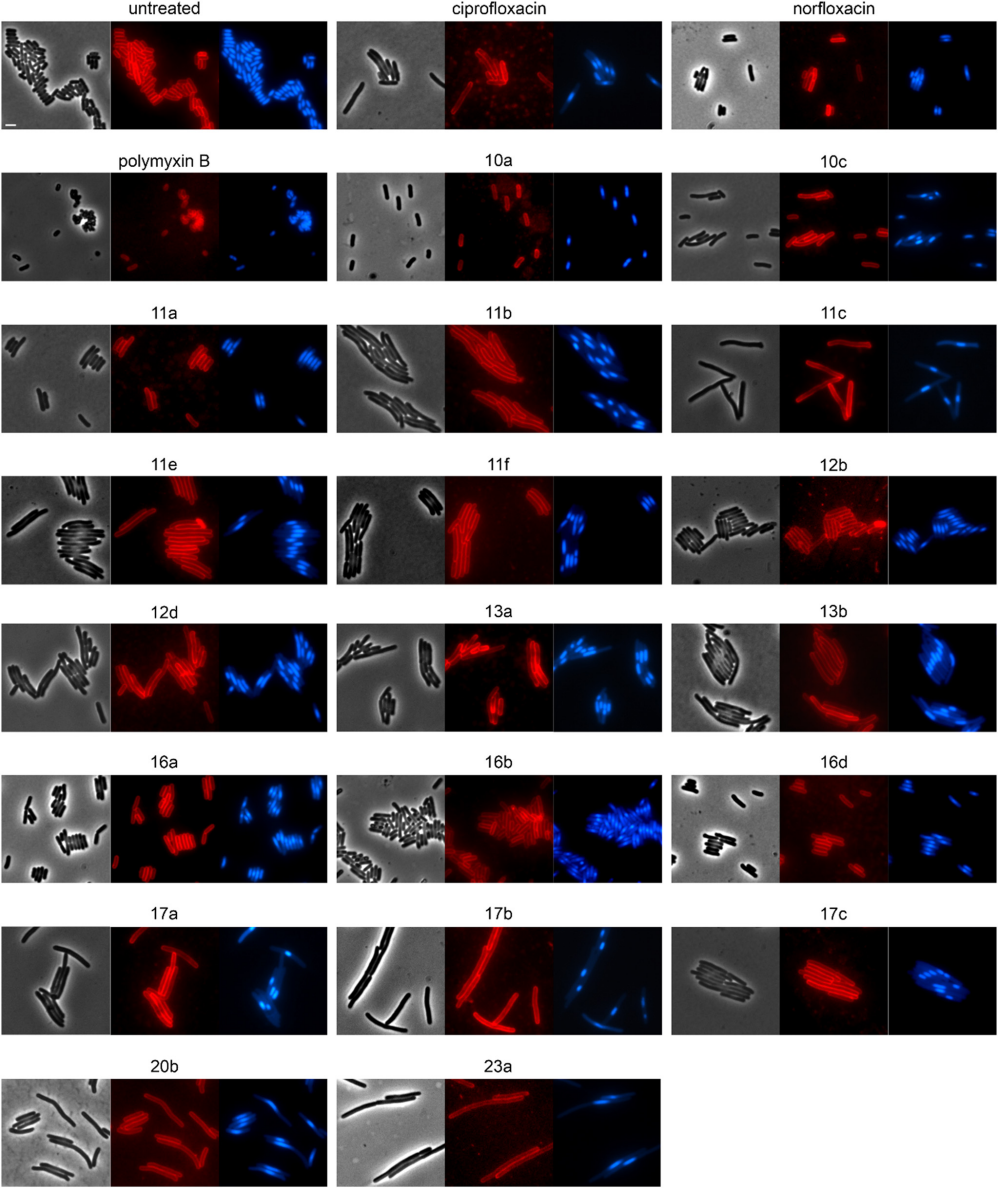
Fluorescence and phase contrast microscopy of *E. coli* W3110. Cells were treated with 1xMIC of the respective compounds for 10 min (polymyxin B) or 60 min (all other compounds) prior to staining with FM4-64 (membrane, red) and DAPI (nucleoid, blue). Norfloxacin and ciprofloxacin were used as fluoroquinolone controls. Polymyxin B was included as control for membrane damage. Scale bar 2 μm.

We selected 19 compounds with promising Gram-negative activity and performed BCP with *E. coli* W3110. All tested derivatives showed a typical nucleoid compaction phenotype and compounds 11b, 11c, 11d, 11e, 11f, 13b, 17a, 17b, 17c, 20b, and 23a additionally showed considerable cell elongation (Fig. 3 and S138, Table S11†). No membrane defects were observed with the FM4-64 membrane dye, yet this dye does not allow reliable distinction between the outer and inner membrane. Thus, we used a green-fluorescent protein (GFP) fusion to the ubiquitous membrane protein GlpT as proxy for the inner membrane (*E. coli* BCB472),^36^ yet this did not show any membrane aberrations either (Fig. S139, Table S11†).

#### Effects on the outer membrane of *E. coli*

Since our compounds were designed with the potential for additional binding sites in LpxC and *in silico* modeling suggested that they may be able to interact with this enzyme, we tested whether they had an effect on the outer membrane of *E. coli*. To this end, we determined synergy with mupirocin using checkerboard assays. Mupirocin is a translation inhibitor that binds to isoleucine tRNA synthase, but is not normally active against *E. coli* as it cannot cross its outer membrane.^37^ Yet, when combined with outer membrane-permeabilizing agents like polymyxin B nonapeptide, it shows considerable synergy (Table S12†). Compound 16b and ciprofloxacin were synergistic with mupirocin (fractional inhibitory concentration index (FICI) = 0.172 and 0.281, respectively) and 19b was just at the synergy cutoff value of 0.5 (FICI = 0.547), while all other compounds showed additive effects. While 16b and ciprofloxacin were less synergistic than polymyxin B nonapeptide, which disrupts the lipopolysaccharide layer directly, their FICI values were in the same range as that of ACHN-975, a known LpxC inhibitor (FICI = 0.297).^38^

We therefore tested whether inhibition of LpxC may be responsible for the observed synergy using a newly developed screen for inhibition of this enzyme in culture. To this end, we made use of a plasmid carrying an arabinose-inducible copy of the *lpxC* gene.^39^ If a compound specifically inhibits LpxC and this inhibition contributes to its activity, overexpression of this enzyme should decrease the compound's antibacterial activity. Indeed, when we tested the activity of ACHN-975 against an *E. coli* strain carrying the *lpxC* overexpression plasmid in the presence of rising arabinose concentrations, we saw a clear, inducer-dependent increase of the MIC (Fig. S140†). This effect was neither observed in the empty vector control, nor with the control antibiotics polymyxin B (permeabilizes both the inner and outer membrane) and nitrofurantoin (causes macromolecule damage through generation of reactive species), showing that our assay works well and is specific for inhibition of LpxC (Fig. S140†). However, we did not observe any significant effects for ciprofloxacin, 16b, 19b, or any of the other compounds, suggesting that they do not inhibit LpxC *in vivo*, at least not to an extent that would contribute to their activity.

### Bacterial cytological profiling of *B. subtilis*

To assess the mechanisms of action of the new compounds in Gram-positive bacteria, we selected 20 compounds with good Gram-positive activity and performed BCP with *Bacillus subtilis* DSM402 (see Table S13† for MICs against this strain). Except for 10f and 11f, all tested derivatives showed a clear gyrase inhibition phenotype and almost all compounds also caused cell elongation, most notably 11a, 11b, 11c, 12b, 12d, and 20b. Additionally, compounds 10a, 10c, 10f, 12b, 12d, 17a, 17b, 17c, and 17e showed clear membrane aberrations, while 4a, 11a, 11b, 11c, 11e, 11f, 16a, and 16d displayed mild membrane defects (Fig. S141, Table S13†). It should be noted, that both ciprofloxacin and norfloxacin showed similar membrane defects in *B. subtilis*, suggesting that membrane damage may be a common mechanistic component of fluoroquinolones. Therefore, we assessed the membrane potential using the fluorescence probe DiSC (3)5.^40^ While we did observe a minor, transient depolarization after treatment with 10f, 11f, and 17e, none of the other compounds affected the membrane potential, including norfloxacin and ciprofloxacin (Fig. S142†).

### Effects on cell division in *B. subtilis*

**Fig. 4:**
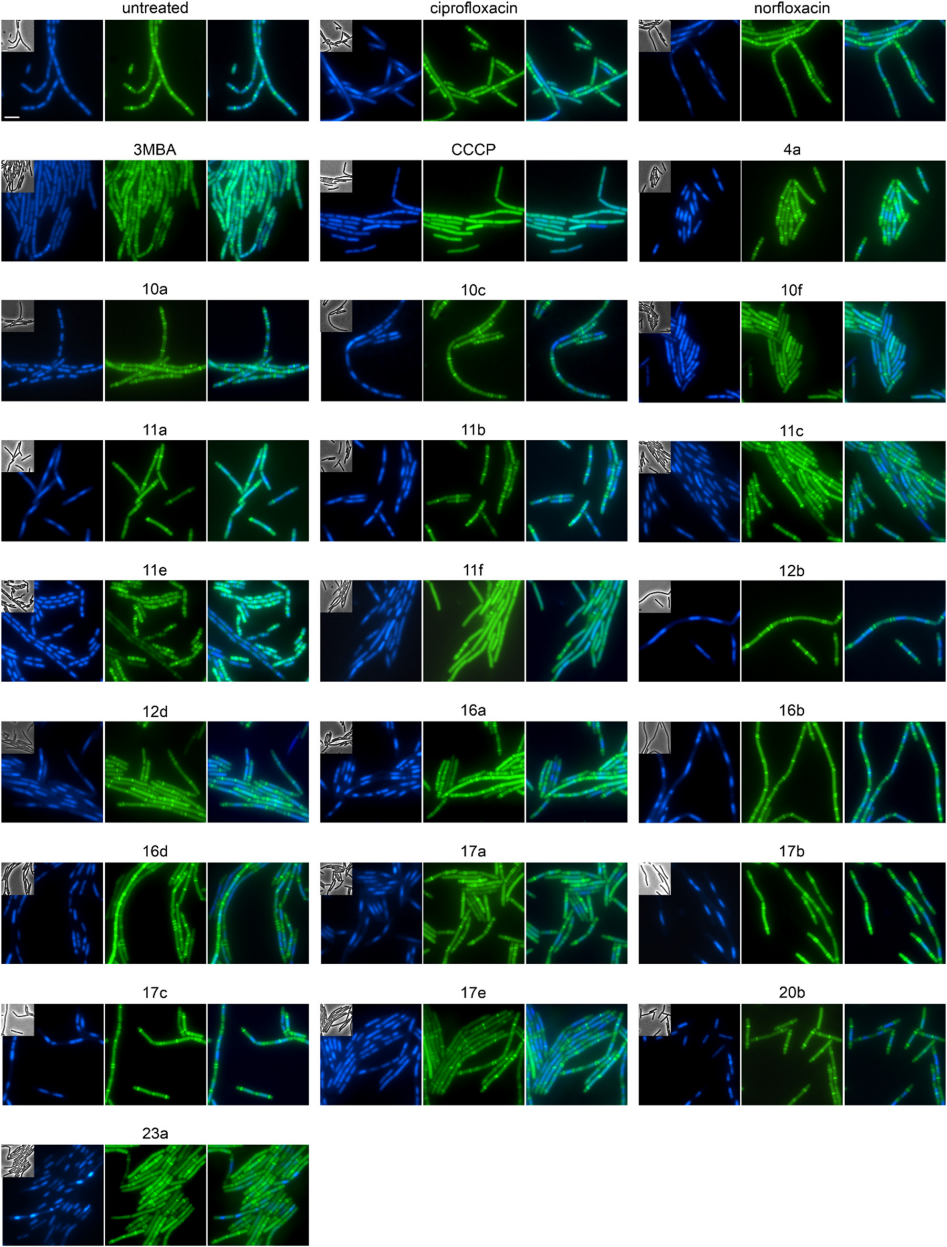
Fluorescence and phase contrast microscopy of *B. subtilis* 2020. Expression of FtsZ-GFP was induced with 0.5% xylose. Cells were treated with 1xMIC of the respective compounds 60 min prior to microscopy. The FtsZ inhibitor 3-methoxybenzamide (3MBA, 6 mM) was used as control for a cell division inhibitor and the proton ionophore carbonyl cyanide *m*-chlorophenyl hydrazone (CCCP, 75 μM) as control for membrane depolarization. DNA was stained with DAPI (blue) to visualize Z-ring placement (green) in relation to the nucleoid. Corresponding phase contrast images are shown as insets in the DAPI panels. Scale bar 2 μm.

Both *E. coli* and *B. subtilis* showed an elongated phenotype in the BCP (Fig. 3, S141, and S143, Table S13†), suggesting inhibition of cell division whereby effects were more common in *B. subtilis*. In the first place, this is not unexpected since the inhibition of topoisomerases impairs DNA replication and decatenation of nucleoids, and nucleoid segregation and cell division are coupled.^41^ Yet, several derivatives showed stronger effects on cell length than norfloxacin and ciprofloxacin, suggesting that the new derivatives may have stronger or additional effects on the cell division machinery. Therefore, we examined the localization of the cell division protein FtsZ. FtsZ is the major cell division protein in bacteria. It forms the Z-ring, which localizes at mid-cell and constricts to form the cell division septum together with the rest of the divisome machinery.^42^ Z-ring placement is tightly regulated to only occur at mid-cell through two systems, the Min system (MinCD, MinJ, and DivIVA in *B. subtilis*), which inhibits Z-ring formation at the cell poles, and the nucleoid occlusion system (Noc), which prevents formation of the Z-ring over unsegregated nucleoids.^43^ As controls, we used the FtsZ inhibitor 3-methoxybenzamide (3-MBA) and the proton ionophore carbonyl cyanide *m*-chlorophenyl hydrazone (CCCP). 3-MBA caused characteristic effects on FtsZ,^44^ namely double rings, FtsZ spirals, and septa forming over unsegregated nucleoids (Fig. 4). CCCP caused complete dissociation of FtsZ into the cytosol, which is due to the membrane anchor proteins of FtsZ, FtsA and SepF, being sensitive to dissipation of the membrane potential.^45^ Norfloxacin and ciprofloxacin showed clear, yet distinct effects. Thus, they caused double Z-rings, yet no FtsZ spirals or dissociation of FtsZ from the membrane. Instead, we observed unevenly spaced Z-rings with much larger distances than in untreated cells, consistent with the condensed nucleoid occupying the space between. Occasionally, Z-rings were observed over the nucleoid, which is consistent with a partial decoupling of Noc and FtsZ. The absence of mini cells indicates that the Min system remained undisturbed.^46^ Most derivatives showed similar but stronger effects, presenting as a high number of double or even multiple Z-rings (most notably 16d, 17b, and 17c), even larger spacing between Z-rings (most notably 12b, 16b, and 17b), and rings over unsegregated nucleoids (most notably 10a, 10c, and 11b). Only two compounds did not show effects on Z-ring placement, 10f and 11e, both of which did not show a clear gyrase inhibition phenotype in the BCP (Fig. S141, Table S13†). 10f and 11f showed dissociation of FtsZ into the cytosol, which is consistent with their mild effect on the membrane potential in the DiSC(3)5 assay (Fig. S142†). Taken together, we can conclude that the new compounds have stronger effects on the cell division machinery than norfloxacin and ciprofloxacin, which are most likely mediated by their stronger effects on nucleoid packing pushing Z-ring placement closer to the cell poles and occasionally leading to failure of Noc and consequently Z-ring formation over the nucleoid.

#### Effects on peptidoglycan synthesis in *B. subtilis*

Almost all tested derivatives showed some degree of membrane aberrations in the BCP, but none was sufficiently depolarizing to explain this effect with a dissipation of the membrane potential.^47^ Other possible explanations include membrane phase separation and inhibition of cell wall synthesis.^48,49^ Therefore, we examined the effects of our norfloxacin derivatives on peptidoglycan synthesis in *B. subtilis* (see Table 2 for results summary of all cell wall assays).

**Table 2:** Summary of cell wall synthesis experiments. Positive results are highlighted in **bold**. PG: peptidoglycan, Cip: ciprofloxacin, Nor: norfloxacin, Van: vancomycin, d-cyc: d-cycloserine, Fos: fosfomycin, Tun: tunicamycin

Compound	Concentration (μM)	PG integrity	MreB mobility	MurG localization	MraY localization	PbpB localization	PonA localization	PG synthesis inhibition?
Untreated		Intact	Mobile	Spotty	Rough	Smooth	Septal	No
Cip	3.01	Intact	Mobile	Smooth	Rough	Smooth	Clusters	No
Nor	18.11	Intact	Mobile	Smooth, clusters	Rough	Smooth	Clusters	No
Van	0.68	Compromised	Static	Dispersed, clusters	Rough	Smooth	Septal	Yes
4a	74.43	Intact	Mobile	Spotty	n.d.	n.d.	n.d.	No
10a	47.99	Intact	Mobile	Spotty	n.d.	n.d.	n.d.	No
10c	33.58	Intact	Mobile	n.d.	n.d.	n.d.	n.d.	No
10f	18.02	Intact	Mobile	**Clusters**	**Clusters**	**Clusters**	**Clusters**	No^a^
11a	2.87	Intact	Mobile	Smooth	n.d.	n.d.	n.d.	No
11b	22.07	Intact	Mobile	Smooth	n.d.	n.d.	n.d.	No
11c	1.33	Intact	Mobile	Smooth	n.d.	n.d.	n.d.	No
11e	1.17	Intact	Mobile, clusters	Spotty, **clusters**	Rough	Smooth	Septal	No
11f	1.08	Intact	Mobile, clusters^c^	Smooth, **clusters**^c^	n.d.	n.d.	n.d.	No
12b	30.98	Intact	Mobile	Spotty	n.d.	n.d.	n.d.	No
12d	198.98	Intact	Mobile	Smooth	n.d.	n.d.	n.d.	No
16a	24.31	Intact	**Static, clusters**	Spotty	n.d.	n.d.	n.d.	**Yes**
16b	55.90	Intact	Mobile, clusters	n.d.	n.d.	n.d.	n.d.	No
16c	16.11	Intact	Mobile, clusters	n.d.	n.d.	n.d.	n.d.	No
16d	31.19	Intact	Mobile, clusters	Spotty	n.d.	n.d.	n.d.	No
17a	33.43	Intact	**Static, clusters**	**Polar foci**	Rough	Smooth	**Clusters**	**Yes**
17b	7.17	Intact	**Static, clusters**	**Polar foci**	Rough	Smooth	**Clusters**	**Yes**
17c	23.46	Intact	Mobile	Spotty	n.d.	n.d.	n.d.	No
17e	126.10	Intact	Mobile, clusters	n.d.	n.d.	n.d.	n.d.	No
20b	8.92	Intact	Mobile, clusters	**Polar foci**	Rough	Smooth	**Clusters**	**Possible** b
23a	17.95	Intact	Mobile	Spotty	n.d.	n.d.	n.d.	No

a Likely not specific cell wall synthesis inhibition but indirect effects mediated by membrane effects.

b No effect on MreB mobility but new phenotype for MurG, needs further clarification.

c Minor effects.

We first employed a simple screen based on phase contrast microscopy of cells fixed with acetic acid/methanol.^50,51^ This fixation results in ‘bubbles’ on the cell surface, where the protoplast protrudes through cell wall breaches when the peptidoglycan layer is damaged. However, none of the tested derivatives caused a clear increase in damaged cells (Fig. S144,†Table 2). Acetic acid/methanol fixation only tests positive, when cell wall autolysins are active, is strongly concentration-dependent, and does not react to all types of cell wall synthesis inhibition.^50,51^ Therefore, we followed up with another assay based on the mobility of MreB.

**Fig. 5:**
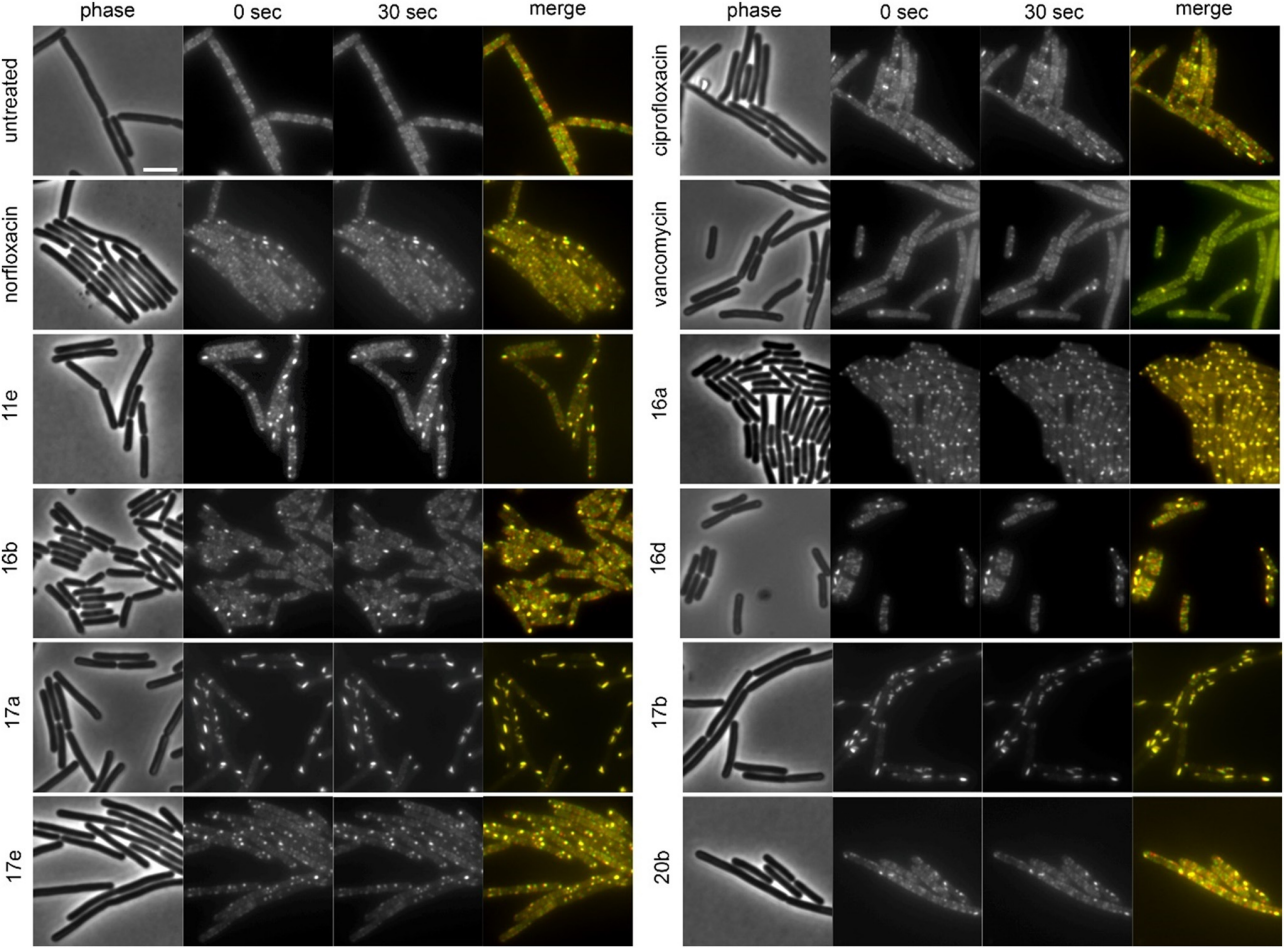
Fluorescence and phase contrast microscopy of *B. subtilis* MW10. Expression of MreB-msfGFP was induced with 0.3% xylose. Cells were treated with 1xMIC of the respective compounds for 10 min (vancomycin) and 60 min (all other compounds) prior to microscopy. Vancomycin was used as cell wall synthesis inhibition control. Images were taken 30 s apart to capture MreB mobility. Scale bar 2 μm.

The cell shape-determining protein MreB is an actin homologue that forms filaments and moves along the lateral cell axis in a spiraling motion, thereby driving forward lateral cell wall synthesis and promoting rod shape.^52,53^ While MreB localization can be affected by the membrane potential, membrane phase separation, membrane invaginations, and inhibition of cell wall synthesis, its mobility is highly sensitive and specific for the latter. In fact, every cell wall synthesis inhibitor we have tested so far abolished MreB movement. Indeed, MreB mobility was entirely abolished by compounds 16a, 17a, and 17b (Fig. 5, Table 2). Compounds 11b, 11c, 11e, 11f, 16b, 16c, 16d, 17e, 20b, norfloxacin, and ciprofloxacin showed static clusters but remaining unclustered MreB retained its mobility, which is an indication for indirect effects due to membrane damage rather than inhibition of cell wall synthesis. The remaining derivatives that were tested did not impair MreB movement (Fig. 5, Table 2, Fig. S145†). Interestingly, 17a and 17b showed a very peculiar localization of MreB: the protein was entirely displaced into brightly fluorescent, static, and elongated foci at the cell membrane. This phenotype has not yet been observed with any other antibiotic, suggesting that these two compounds may possess an entirely new mechanism of action. Yet, a similar phenotype has been seen upon expression of the toxin–antitoxin system BsrG/SR4 in *B. subtilis*,^54^ suggesting that 17a and 17b may either act by a similar mechanism or trigger expression of the toxin BsrG, in turn causing similar effects. Importantly, BsrG has been shown to impair the cell wall synthesis machinery,^54^ among other effects, supporting our case that these derivatives inhibit this pathway.

We then assessed the localization of different cell wall synthesis enzymes in *B. subtilis* to get a first idea of which step of peptidoglycan synthesis may be impaired by the compounds. To this end, we chose four proteins fused to GFP: MraY, MurG, PonA, and PbpB. MraY synthesizes lipid I, the precursor of lipid II. MurG converts lipid I to lipid II, which is then flipped to the outer membrane leaflet and incorporated into the peptidoglycan layer by penicillin-binding proteins (PBPs). PonA (PBP1) is a PBP that is involved in both lateral and septal cell wall synthesis, while PbpB (PBP2b) is part of the divisome complex and involved in septum formation.^55,56^

**Fig. 6:**
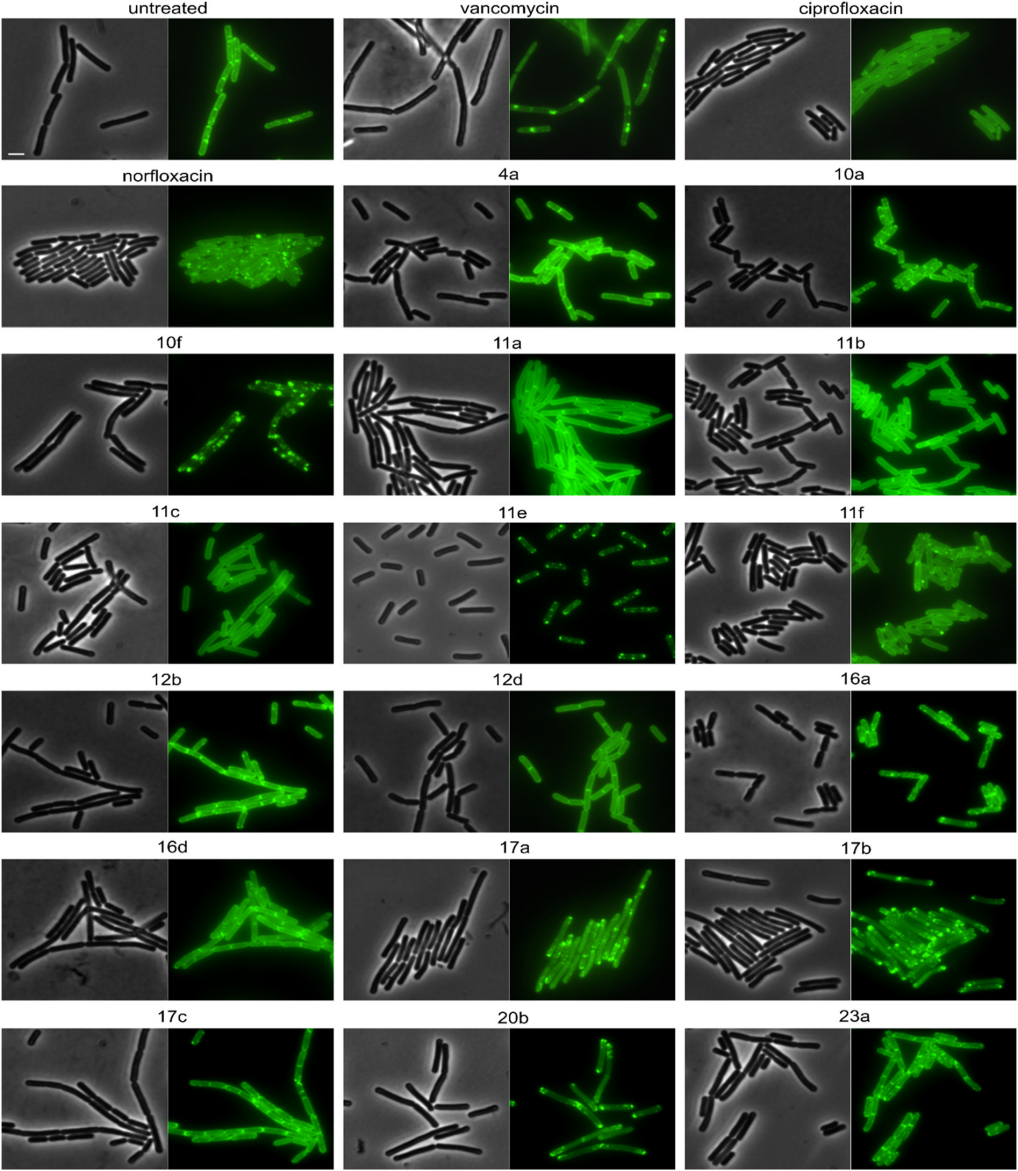
Fluorescence and phase contrast microscopy of *B. subtilis* TNVS175. Expression of MurG-msfGFP was induced with 0.05% xylose. Cells were treated with 1xMIC of the respective compounds for 30 min (vancomycin) or 60 min (all other compounds) prior to microscopy. Vancomycin was used as cell wall synthesis inhibition control. Scale bar 2 μm.

Derivatives were first tested against MurG, which is the most sensitive of the selected enzymes. It is a peripheral membrane protein that localizes in small spots at the membrane in fast-growing cells but exhibits a smooth membrane localization in slow-growing cells.^57^ Upon inhibition of cell wall synthesis, it either accumulates in large clusters (see vancomycin, Fig. 6) or is displaced into the cytosol.^57^ Interestingly, several different phenotypes were observed with this reporter protein. Compounds 4a, 10a, 12b, 16a, and 16d were similar to the untreated control, while 11a, 11b, 11c, 12d, and ciprofloxacin showed a smooth membrane localization, probably due to slowed growth as a secondary effect of impaired DNA packing. Norfloxacin, 10f, 11e, and 11f showed clustering of the protein to varying degrees, 10f having the strongest effect. The most intriguing observation was again made for 17a and 17b, which did not abolish MurG foci but appeared to relocalize them specifically to the cell poles. This is curious since cell wall synthesis in *B. subtilis* occurs at the lateral cell axis and septa, not at the poles.^58^ Similarly to their effects on MreB, this phenotype has not been observed with any other antibiotic or any other condition before, supporting the notion that 17a and 17b act through a new mechanism of action. Interestingly, 20b showed a similar effect on MurG but not on MreB (Fig. 5 and 6), indicating that these two activities may be unrelated, yet future studies will be needed to further elucidate the precise mechanisms of these compounds.

**Fig. 7:**
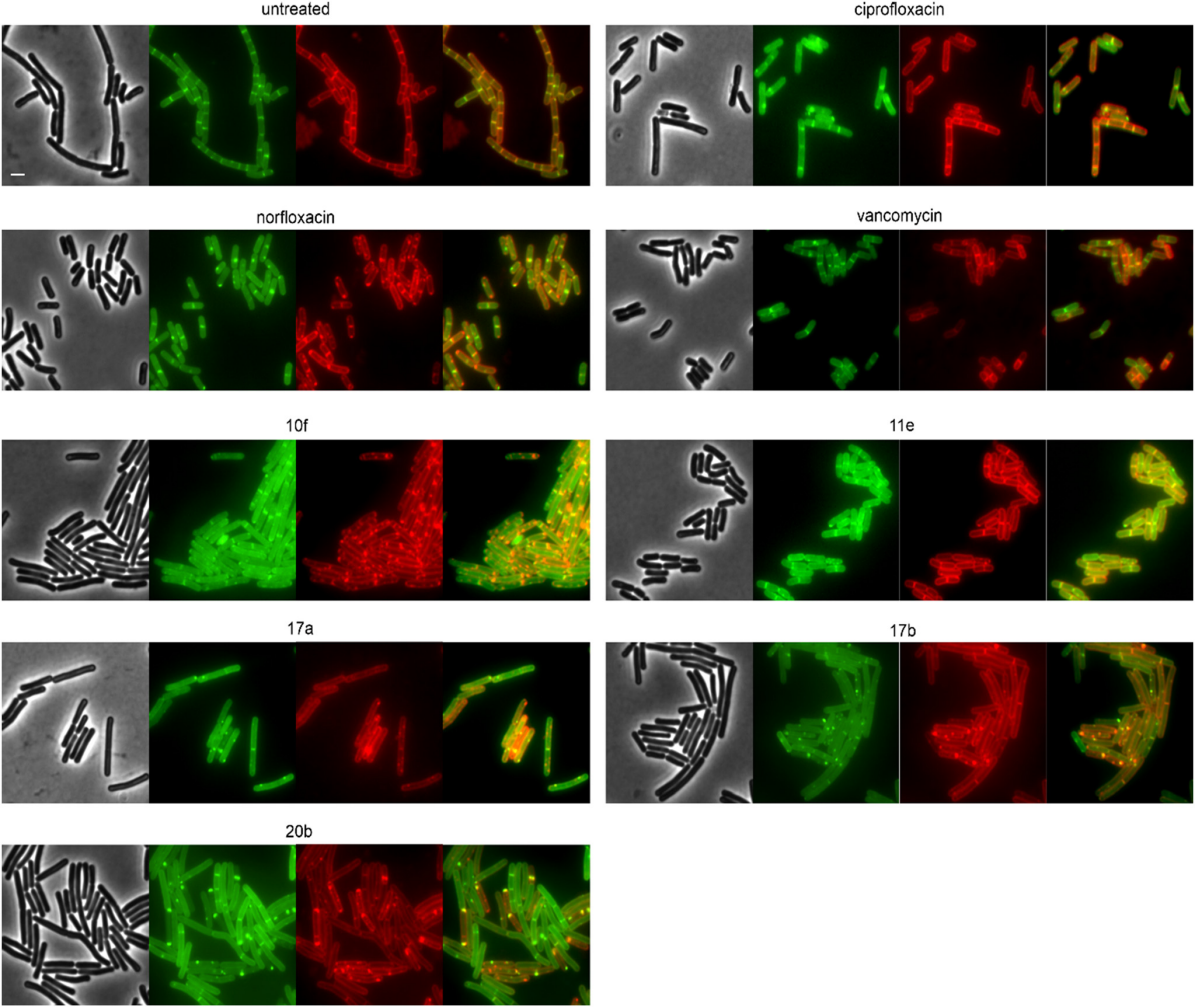
Fluorescence and phase contrast microscopy of *B. subtilis* TNVS45 (PonA-mGFP). Expression of PonA-mGFP was induced with 0.1% xylose (green). Cells were treated with 1xMIC of the respective compounds for 30 min (vancomycin) or 60 min (all other compounds) prior to microscopy. Vancomycin was used as cell wall synthesis inhibition control. Cell membranes were stained with FM4-6 (red). Composite images show overlays of GFP and FM4-64. Scale bar 2 μm.

We then selected the compounds with the most distinctive phenotypes and tested their effects on MraY, PbpB, and PonA. Only 10f affected the localization of MraY and PbpB, accumulating them into distinct clusters similarly to its effect on MurG (Fig. S146 and 147†). 10f affected PonA in the same manner and caused similar clusters in the membrane stain (Fig. 7). PonA was also clustered by 17a, 17b, and 20b, whereby the latter had the strongest effects. These results suggest that 17a, 17b, and possibly 20b inhibit cell wall synthesis likely at a step within the lipid II cycle. Whether this involves inhibition of the *B. subtilis* NagA homologue, remains to be assessed. 10f rather impairs the cell membrane, causing secondary effects on cell wall synthetic enzymes. The fact that 10f did not cause stable membrane depolarization makes it an interesting compound, since non-depolarizing membrane-active antibiotic agents are incredibly rare.

Correlating our *in vivo* mode of action data with the structure of the compounds, we can speculate about the structural determinants underlying their polypharmacological properties. Thus, the isatin-based derivatives 16a, 17a, and 17b all displayed effects on cell wall synthetic enzymes, suggesting that the isatin moiety may afford an additional interaction with a component of this pathway. Thereby, hydroxamic acid variants 17a and 17b showed novel phenotypes suggesting a not yet described mechanism of cell wall synthesis inhibition, which may involve an interaction with this residue. Similar results were obtained for the morpholinomethyl-substituted hydroxamic acid derivative 20b, suggesting that a bulky residue at the *N*-piperazinyl moiety together with a hydroxamic acid substitution are causing this new phenotype. Yet, compared to the other compounds 20b showed distinct effects on MurG and MreB, possibly reflecting the different *N*-piperazinyl substitutions. Compounds 10f and 11f were the only tested compounds that displayed clear effects on the cell membrane, suggesting that their chlorobenzyl moiety is responsible for this activity.

## Conclusion

We have designed and synthesized two series of norfloxacin hydroxamic acid derivatives with the aim to introduce additional pharmacophores that enable the compounds to interact with targets other than gyrase and topoisomerase IV. Several derivatives showed activities that were as good as or better than that of their parent compound norfloxacin. The most interesting compounds were selected for *in silico*, *in vitro*, and *in vivo* mode of action studies, revealing that all but one tested derivative inhibited gyrase and topoisomerase IV. While we originally intended LpxC as secondary target of our derivatives and *in silico* analysis supported this idea, *in vivo* data could not confirm this notion. However, our compounds still showed polypharmacological effects, targeting either the cell membrane (10f, 11f) or cell wall synthesis (16a, 17a, 17b, 20b), at least in Gram-positive bacteria.

Interestingly, compounds 17a, 17b, and 20b, showed never seen before effects on cell wall synthetic enzymes suggesting a novel mechanism of action, possibly inhibiting the lipid II cycle. Novel mechanisms and targets are urgently needed to avoid cross-resistance with already existing drugs. Further studies will be needed to fully elucidate the mechanisms behind cell wall synthesis inhibition by these compounds and to assess their true promise for further drug development.

Compound 10f is the only tested derivative that could not be confirmed to target gyrase *in vivo*. However, it elicited membrane aberrations and membrane protein clustering that appeared to be independent of membrane depolarization, as supported by membrane potential measurements and MreB localization. This suggests that 10f is a non-disruptive membrane-targeting fluoroquinolone, and thus first-in-class. Moreover, 11f, which differs from 10f only by the presence of the hydroxamic acid group, showed topoisomerase inhibition in *E. coli* but not in *B. subtilis*, rather interacting with the cell membrane in the latter. This is a curious finding since such fundamentally different mechanisms of action against different species are not often reported and may open up possibilities to finetune compounds for activity against specific species.

Polypharmacology is a desired feature of next-generation drugs, since multitarget antibiotics display slower resistance development that single-target antibiotics.^15^ Our new derivatives combine two of the most powerful and clinically successful antibacterial mechanisms of actions known to date, topoisomerase inhibition and cell wall synthesis inhibition, suggesting that they are at least very interesting lead structures for further drug development. Further studies will elucidate the detailed mechanisms of action of these new promising polypharmacological compounds.

## Experimental

Methods can be found in Text S12–S15, Fig. S148, and Tables S14–S16.†

## Author contributions

Conceptualization: MW, FO. Data curation: AK, IANA, MK, AA, JP, ABS, MW. Formal analysis: IANA, MK, AA, JP, MW. Funding acquisition: MW. Investigation: AK, IANA, MK, AA, JP, ABS, MW, FO. Project administration: MW, FO. Methodology: MK, JP, AA, MW. Resources: AA, MW, FO. Supervision: AA, MW, FO. Validation: AK, IANA, MK, AA, JP, ABS, MW, FO. Visualization: AK, IANA, MK, AA, JP, ABS, MW, FO. Writing – original draft: MK, MW, FO. Writing – review & editing: AA, MW, FO.

## Conflicts of interest

The authors have declared no conflict of interest.

## Supplementary Material

MD-014-D3MD00309D-s001
