# Supplementary material for: Rational design, synthesis, molecular modeling, biological activity, and mechanism of action of polypharmacological norfloxacin hydroxamic acid derivatives
Source: RSC Med Chem. 2023 Sep 19;14(12):2593–610. doi: 10.1039/d3md00309d (PMC10718593; doi:10.1039/d3md00309d)
Supplement: MD-014-D3MD00309D-s001 [file MD-014-D3MD00309D-s001.pdf]

Electronic Supplementary Material (ESI) for RSC Medicinal Chemistry.
This journal is © The Royal Society of Chemistry 2023

Supplementary Material

Rational design, synthesis, molecular modeling, biological activity, and mechanism of action of polypharmacological norfloxacin hydroxamic acid derivatives

Ahmed M. Kamal El-sagheir[1], Ireny Abdelmesseh Nekhala[2], Mohammed K. Abd El-Gaber[1], Ahmed S. Aboraia[1], Jonatan Persson[2,3], Ann-Britt Schäfer[2,3], Michaela Wenzel[2,3*], Farghaly A. Omar[1*].

[1]Medicinal Chemistry Department, Faculty of Pharmacy, Assiut University, Assiut, Egypt, 71526

[2]Division of Chemical Biology, Department of Life Sciences, Chalmers University of Technology, 412 96 Gothenburg, Sweden

[3]Center for Antibiotic Resistance Research in Gothenburg (CARe), Gothenburg, Sweden

*Corresponding authors: Michaela Wenzel (wenzelm@chalmers.se) and Farghaly A. Omar (farghalyomar@pharm.aun.edu.eg)

Table of contents

1. **Synthesis and characterization of compounds**
   Text S1: Similar fluoroquinolone derivatives.
   Figure S1: Selected fluoroquinolone derivatives reported in other studies.
   Figure S2: Known LpxC enzyme inhibitors and common pharmacophores.
   Text S2: NMR results
   Figure S3-91: ^{1}HNMR and ^{13}CNMR of the synthesized compounds.
   Figure S92 Elemental analysis certificates.
2. **Prediction of ADME/Tox**
   Text S3: Calculation of physicochemical properties.
   Table S1: Physicochemical parameters of norfloxacin derivatives.
   Text S4: ADME/Tox prediction using pKCSM lab
   Table S2: ADME/TOX properties predicted by pKCSM.
   Text S5: ADME prediction by SwissADME
   Table S3: Physicochemical and pharmacokinetic properties predicted by SwissADME.
   Figure S93: Cytotoxicity and therapeutic windows of compounds **8b** and **20b**.
3. **Molecular modeling**
   Text S6: Docking on *S. aureus* DNA gyrase.
   Figure S94-99: 2D and 3D interactions with *S. aureus* DNA gyrase.
   Table S4: Binding energy scores with *S. aureus* DNA gyrase.
   Text S7: Docking on *A. baumannii* topoisomerase IV.
   Figure S100-109: 2D and 3D interactions with *A. baumannii* topoisomerase IV.
   Table S5: Binding energy scores with *A. baumannii* topoisomerase IV.
   Text S8: Docking on *M. smegmatis* NagA.
   Figure S110-120: 2D and 3D interactions with *M. smegmatis* NagA.
   Table S6: Binding energy scores with *M. smegmatis* NagA.
   Text S9: Docking on *P. aeruginosa* LpxC
   Figure S121-131: 2D and 3D interactions with *P. aeruginosa* LpxC.
   Table S7: Binding energy scores with *P. aeruginosa* LpxC.
   Text S10: Ligand-based pharmacophore modeling.
   Table S8: Query features calculated from the aligned molecules.
   Table S9: Pharmacophore features with distance constraints (Å).
   Figure S132: Query features calculated from the aligned molecules.
   Figure S133: Overlaying of some target compounds with the generated pharmacophore query.
   Figure S134: Alignment of compounds **8a** and **17f** (violet) and **CHIR-12** (green).
4. **Mechanism of action**
   Text S11: Metal-chelating properties
   Figure S135: UV–vis absorption spectra of series **1** compounds **11a, 11b** and **11f.**
   Figure S136: UV–vis absorption spectra of series **2** compounds **17a, 20b** and **23a.**
   Figure S137: The molar ratio of ligand/metal in metal complex of compound **20b**.

Table S10: Absorbance of investigated compounds and their metal complexes.
Figure S138: Cell length of *E. coli* W3110 measured from BCP images.
Figure S139: Fluorescence and phase contrast microscopy of *E. coli* BCB472.
Table S11: Results summary of bacterial cytological profiling of *E. coli*.
Table S12: Results of checkerboard assays of norfloxacin derivatives combined with mupirocin.
Figure S140: Effects on LpxC.
Figure S141: Bacterial cytological profiling of *B. subtilis*.
Table S13: Results summary of bacterial cytological profiling of *B. subtilis*.
Figure S142: Effects of the membrane potential in *B. subtilis* DSM 402.
Figure S143: Cell length of *B. subtilis* DSM402 measured from BCP images.
Figure S144: Effects on peptidoglycan synthesis.
Figure S145: Fluorescence and phase contrast microscopy of *B. subtilis* MW10.
Figure S146: Fluorescence and phase contrast microscopy of *B. subtilis* TNVS284 (MraY-msfGFP).
Figure 147: Fluorescence and phase contrast microscopy of *B. subtilis* EKB46 (msfGFP-PbpB).

5. **Methods**
Text S12: Chemical synthesis.
Text S13: Molecular modeling.
Figure S148: Color scheme for 2D representations of compound interactions.
Table S14: Training set compounds.
Table S15: Validation test set compounds.
Text S14: Cytotoxicity assay.
Text S15 Mechanism of action studies.
Table S16: Strains used in this study.

6. **References**
Text S16: Supplementary references.

1. Synthesis and characterization of compounds

Text S1: Similar reported fluoroquinolone derivatives

Numerous studies have reported a variety of fluoroquinolone derivatives including polypharmacological hybrid molecules[1, 2]. Derivatives most similar to those described here include *N*4-piperazinyl derivatives of norfloxacin and ciprofloxacin[3], norfloxacin Mannich bases of isatin[4, 5], and hydroxamic acid and hydrazide derivatives of ciprofloxacin and levofloxacin. **Figure S1** shows the structures of selected derivatives.

Khan *et.al*. synthesized a series of *N*4-piperazinyl derivatives of norfloxacin (**Figure S1A**) and tested their activity against a range of Gram-negative and Gram-positive bacteria as well as fungi, revealing several derivatives with equal or better activities than norfloxacin. The authors found no toxic activity on *Artemia salina* larvae[6].

Several series of *N*-norfloxacin Mannich bases of isatin were synthesized by Pandeya *et al*. and tested against a range of pathogenic bacteria and fungi. Some compounds showed enhanced activity compared to their parent compound norfloxacin, yet no toxicity was determined[4, 5, 7]. **Figure S1B** shows selected compounds from the most recent publication of this work.

Abdullah *et al*. synthesized hydroxamic acid and hydrazide derivatives of ciprofloxacin and levofloxacin (**Figure 1C**) as inhibitors of urease. Possible interactions of the compounds with *Helicobacter pylori* urease were mapped using molecular docking and the activity of the compounds was assessed against purified *Proteus mirabilis* urease. While several compounds showed better enzyme inhibition than the reference compound acetohydroxamic acid, *in vitro* enzyme inhibition and antimicrobial activity against *P. mirabilis* did not always correlate[8]. Based on these previous efforts and development in synthesis of new fluoroquinolone derivatives, we have taken all of them in consideration to design and synthesize multi-targeted novel hydroxamic norfloxacin derivatives having the same structural moieties to create new hybrid molecules with enhanced antibacterial and antimycobacterial activity.

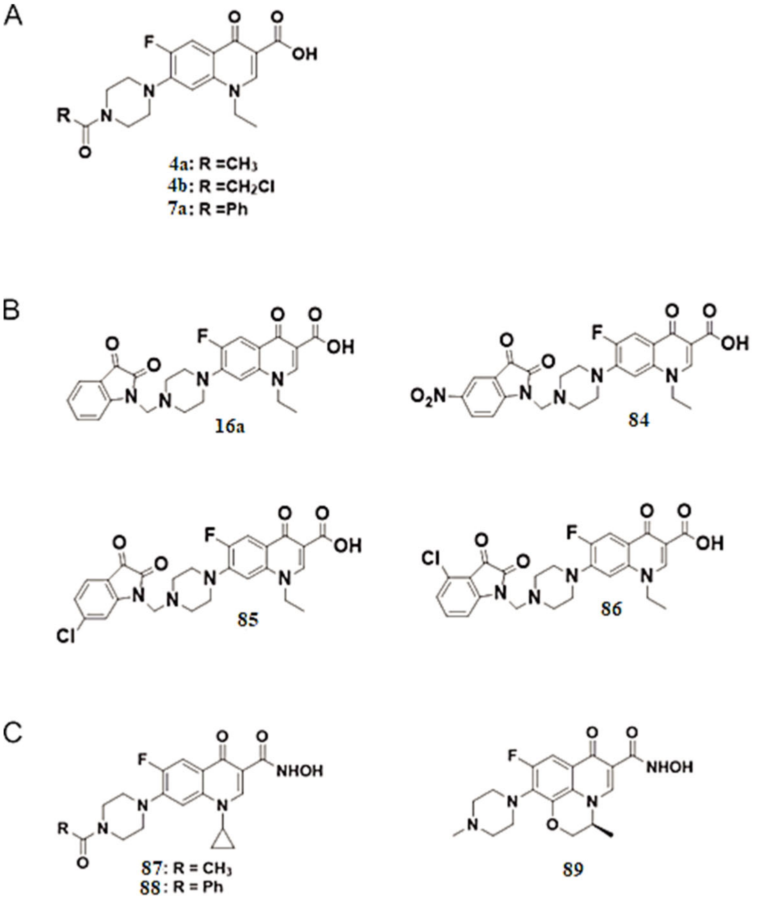

Figure S1: Selected fluoroquinolone derivatives reported in other studies. (A) *N*4-piperazinyl derivatives of norfloxacin designed by Khan *et al*.[6], (B) *N*-norfloxacin Mannich bases of isatin by Pandeya *et al*.[7], (C) hydroxamic acid and hydrazide derivatives of ciprofloxacin synthesized by Abdullah *et al*.[8].

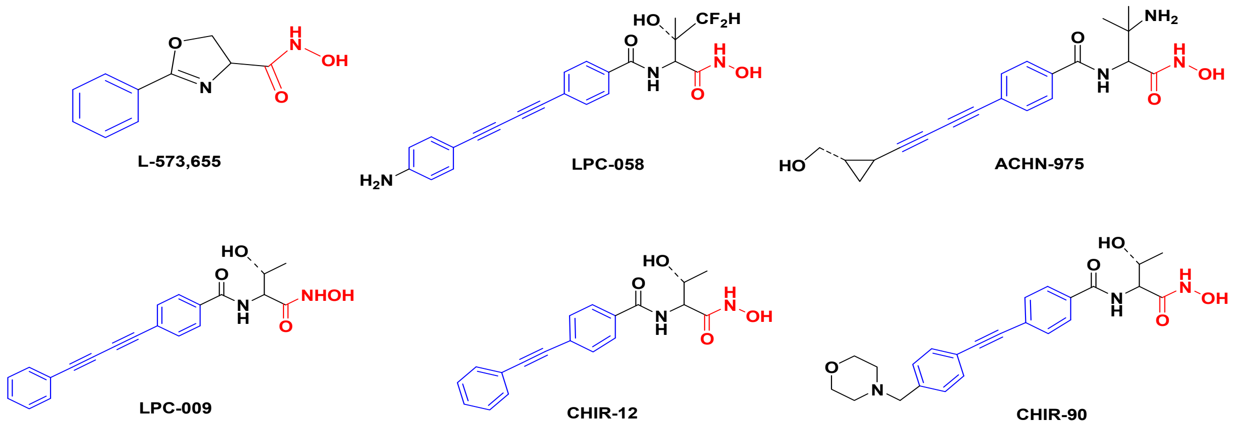

Figure S2: Known LpxC enzyme inhibitors and common pharmacophores. Red: hydroxamate head group, blue: lipophilic tail.

Text S1: NMR results

The synthesis of the target compounds is outlined in **Schemes 1** and **2** and described in the main text. **Scheme 1** shows the preparation of a series of hydroxamic acids of *N*-acyl, sulphonyl, alkyl and phenacylpiperazinyl derivatives of norfloxacin is depicted. All compounds were confirmed by determination of melting points and ^{1}H NMR analysis. New derivatives were identified by IR, ^{1}H NMR, ^{13}C NMR, mass spectra, and elemental analysis. In addition to the expected aromatic protons, the ^{1}H NMR spectra of the compounds showed two characteristic singlet signals at δ 11.77-11.7 and 10.11-9.15 ppm assigned to NH and OH of hydroxamic acid, respectively. Compound **5a** showed a singlet signal at δ 2.06 ppm assigned to the CH_3 group, while compound **5b** had a characteristic singlet peak at δ 3.27 ppm assigned to the $Cl\underline{CH_2}CO$ group. Furthermore, the ^{1}H NMR spectrum of compound **11a** showed a singlet signal at δ 2.71 ppm assigned to the CH_3 group. Compound **11b** had a triplet signal at δ 1.05 assigned to the $CH_2\underline{CH_3}$ group and a quartet signal at δ 2.45 ppm assigned to the $CH_3\underline{CH_2}$ group. In addition, compound **11c** showed a multiplet signal at δ 5.22 ppm assigned to the $\underline{CH_2}$=CH group and a multiplet signal at δ 5.86 ppm assigned to the CH_2=$\underline{CH}$ group. In the case of compounds **11e-f**, ^{1}H NMR spectra showed a singlet signal at δ 3.56 ppm assigned to the $Ph\underline{CH_2}N$ group. The phenacylnorfloxacin derivatives **13a-d** showed a characteristic singlet signal at δ 5.17-3.49 ppm assigned to the $Ph\underline{CH_2}CO$ group. In addition, D_2O exchange was performed on compound **8a** showing that the corresponding peaks for NH and OH of the hydroxamic group disappeared.

Scheme 2 depicts the synthesis of 5-substituted indoline-2,3-dione derivatives **15c-f**, followed by synthesis of a series of hydroxamic acids of different norfloxacin Mannich bases were synthesized. All reported Mannich bases were confirmed by determination of their melting points and ^{1}H NMR analysis. New compounds were identified by their IR, ^{1}H NMR, ^{13}C NMR, mass spectra, and elemental analysis. In addition to the expected aromatic protons, the ^{1}H NMR spectra of compounds **17-26** showed two characteristic singlet signals at δ 11.76-11.73 and 9.2-8.78 ppm assigned to NH and OH of hydroxamic acid, respectively, which was similar to reported chemical shifts[8, 9]. The ^{1}H NMR spectra of all Mannich bases showed a characteristic singlet signal at δ 4.59-3.14 ppm assigned to N-$\underline{CH2}$-N group. Furthermore, the isatin norfloxacin hybrids **17a-f** were characterized by aromatic protons at δ 7.92-7.01 ppm, while compound **23b** showed aromatic protons at δ 7.84-7.18 ppm. Mannich bases of aliphatic amines like compound **20a** showed

characteristic signals at δ 3.5-1.22 ppm assigned to piperidine ring protons, while ^{1}H NMR spectra of compound **20b** showed multiplet signals at δ 3.38-2.49 ppm assigned to morpholine ring protons. Compound **23a** had a singlet signal at δ 2.51 ppm assigned to two $\underline{CH_2}$ groups of succinimide. Synthesis of the hydroxamic acid derivative of the p-nitroaniline Mannich base failed and instead *N*-methyl-norfloxacin hydroxamic acid was formed, which may be explained by the presence of an active NH group in the p-Nitroaniline Mannich base that may interact with ethyl chloroformate, forming carbamate and leading to degradation of the Mannich base[10].

4a

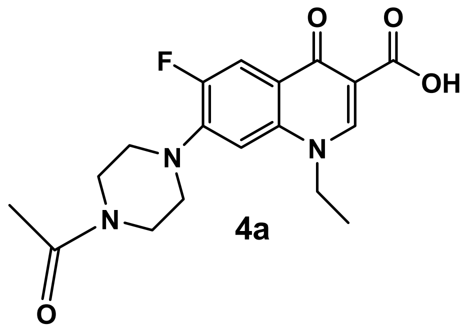

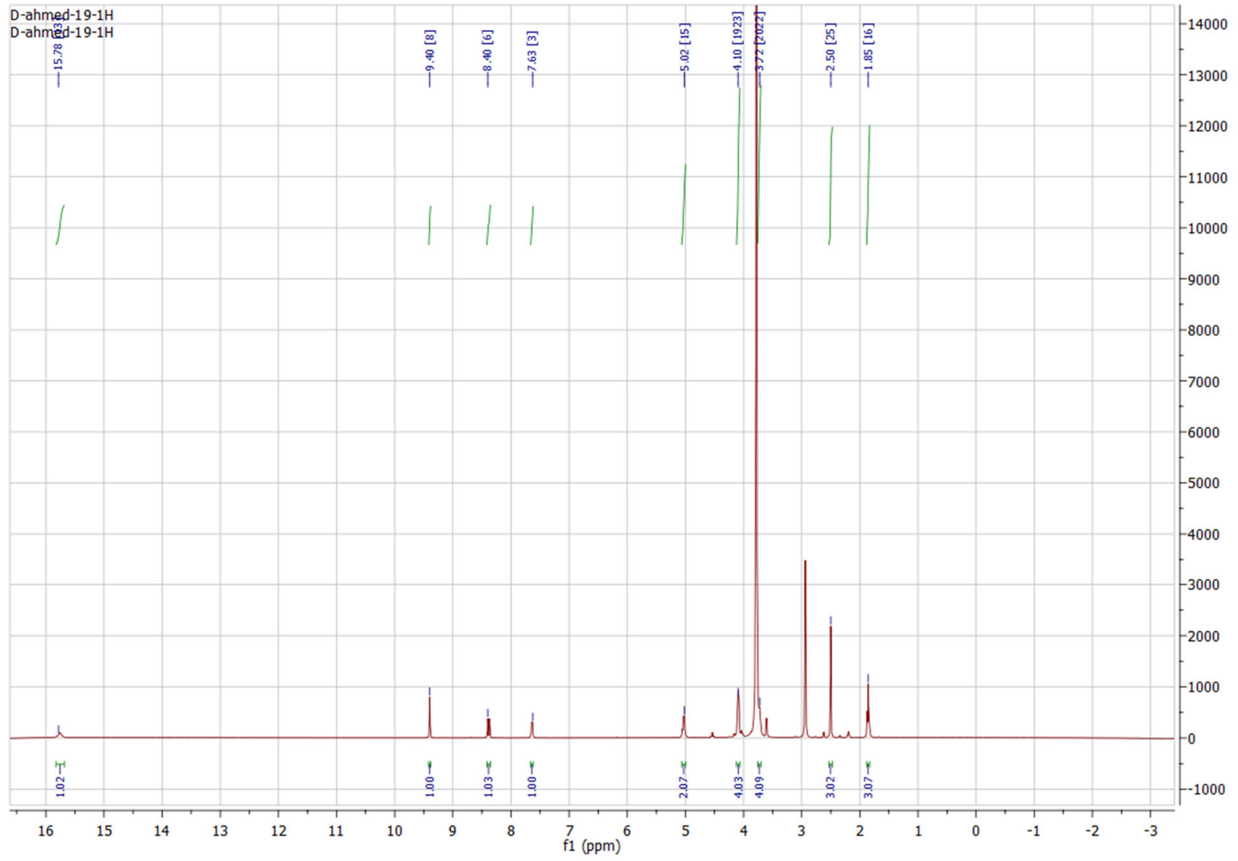

Figure S3: ^{1}H NMR of compound 4a.

4b

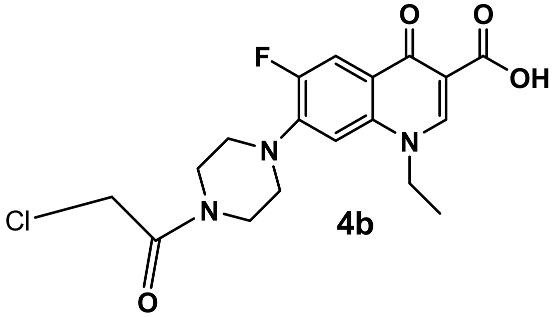

4b

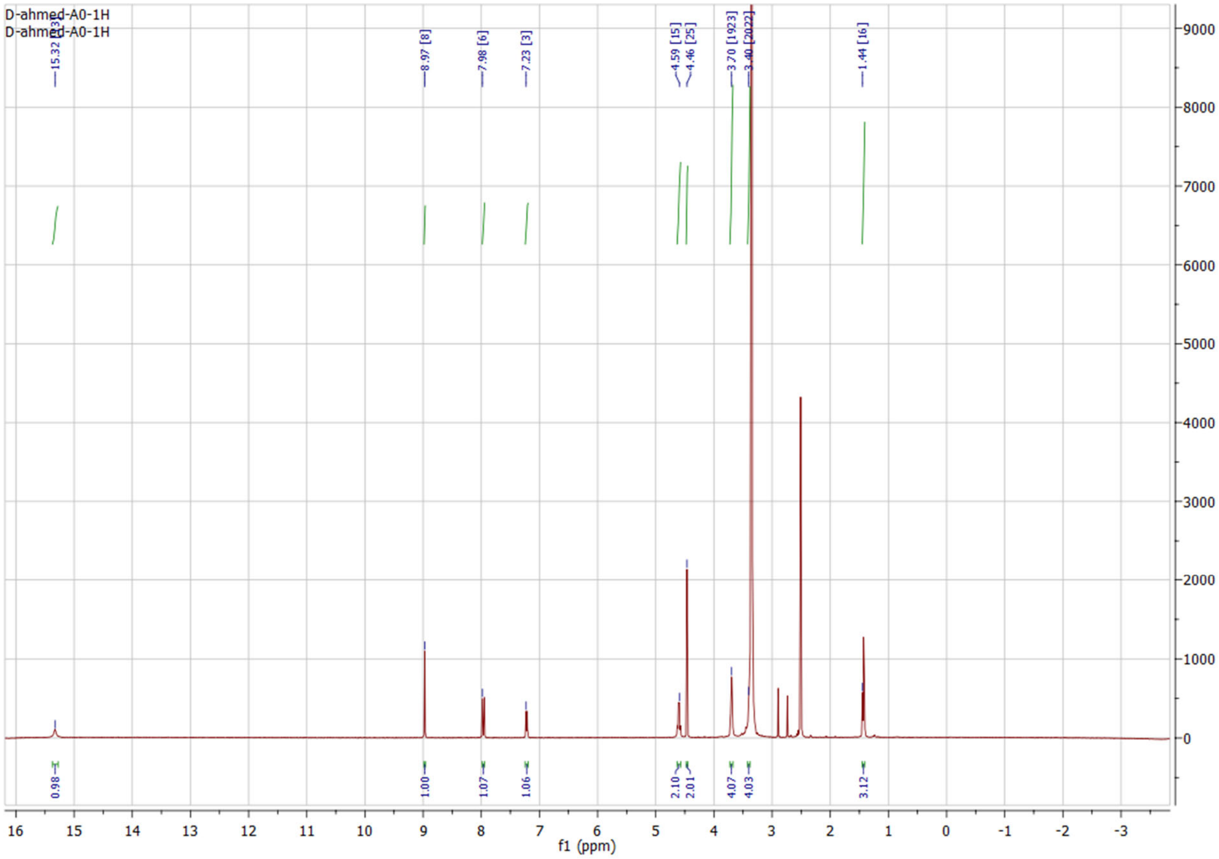

Figure S4: ^{1}H NMR of compound 4b.

5a

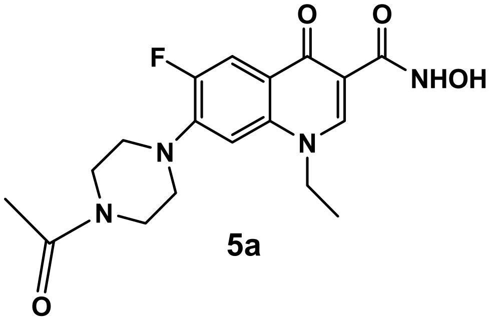

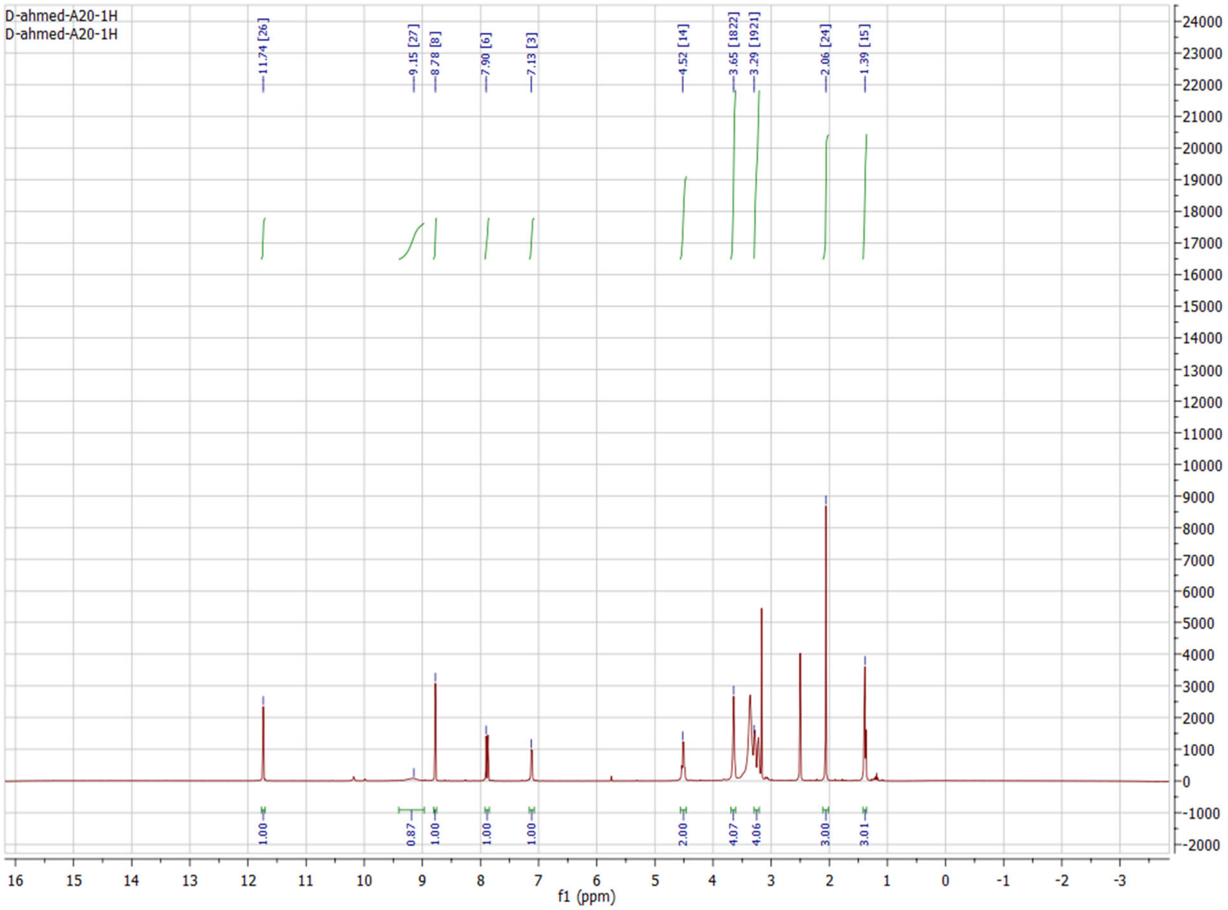

Figure S5: ^{1}H NMR of compound 5a.

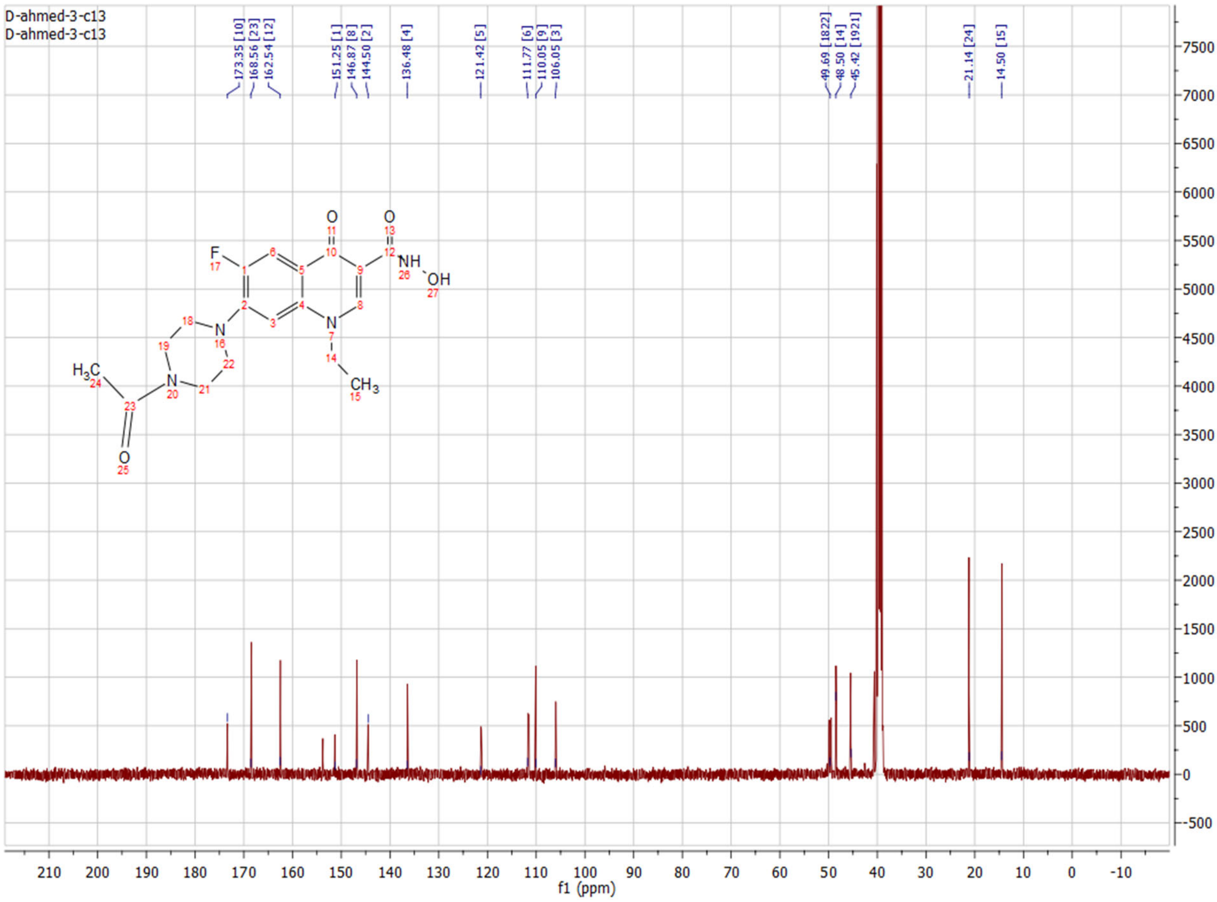

Figure S6: ^{13}C NMR of compound 5a.

5b

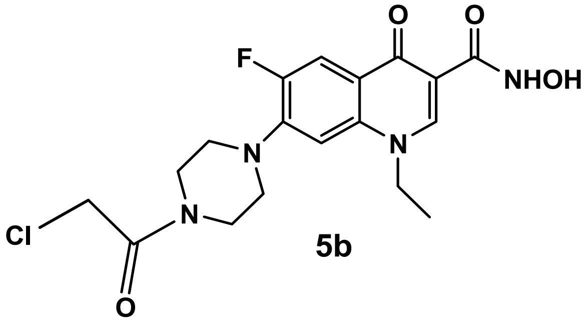

5b

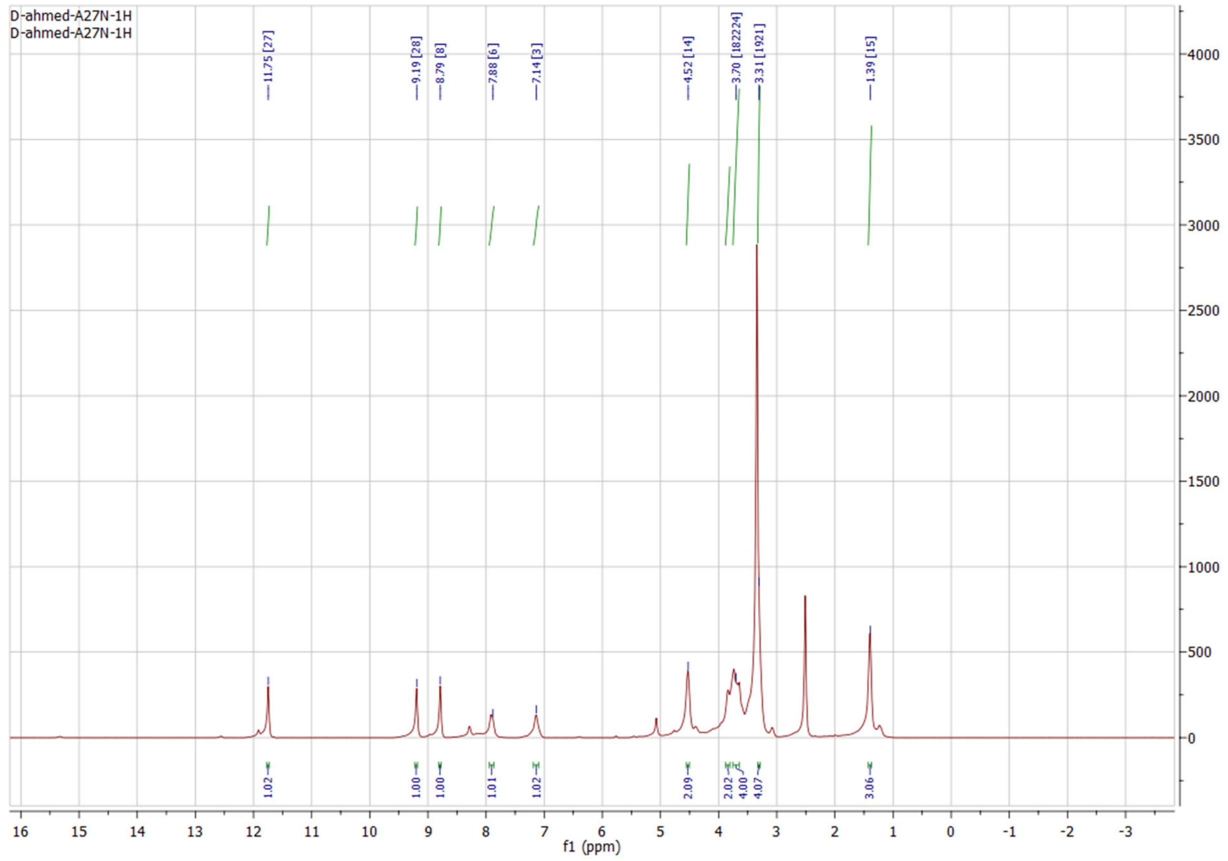

Figure S7: ^{1}H NMR of compound 5b.

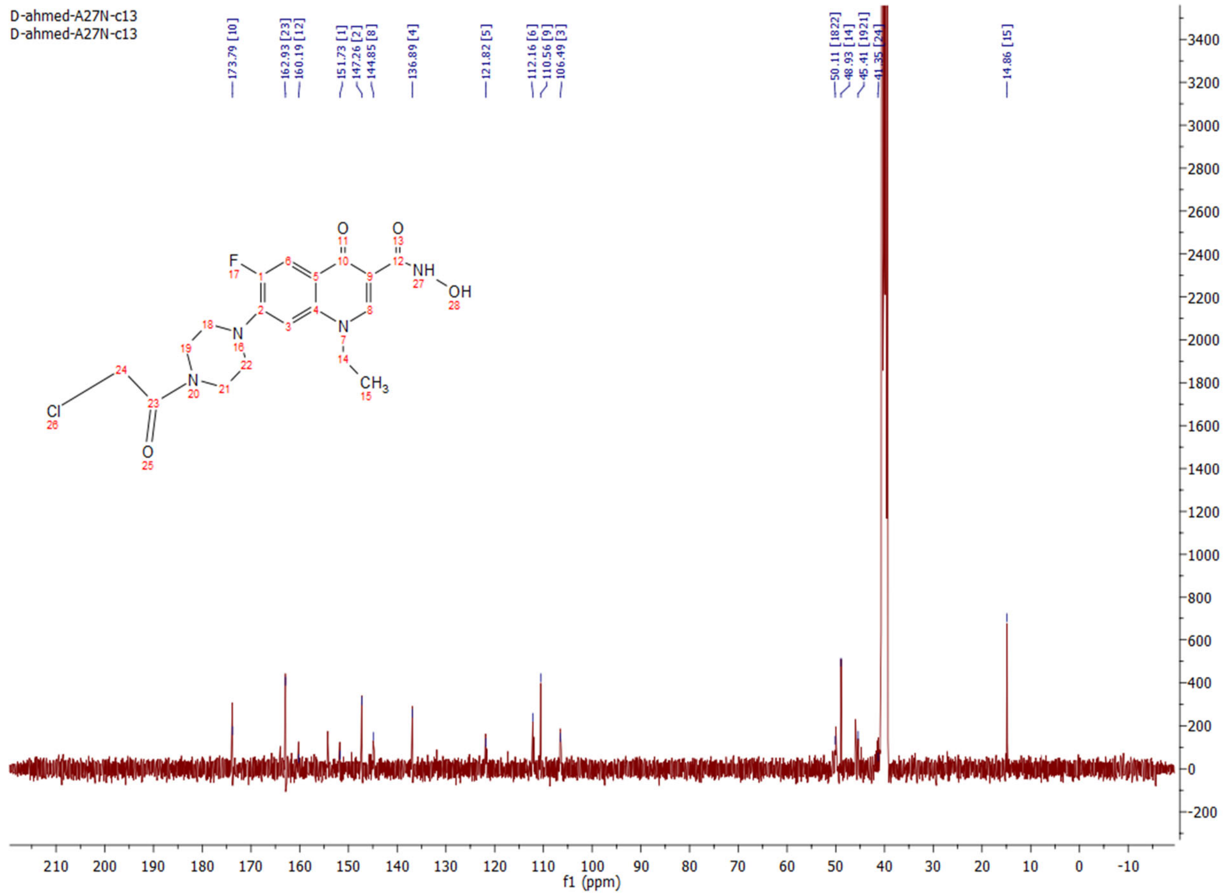

Figure S8: ^{13}C NMR of compound 5b.

7a

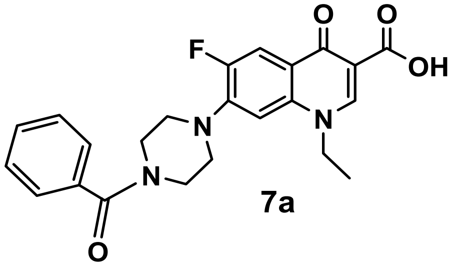

7a

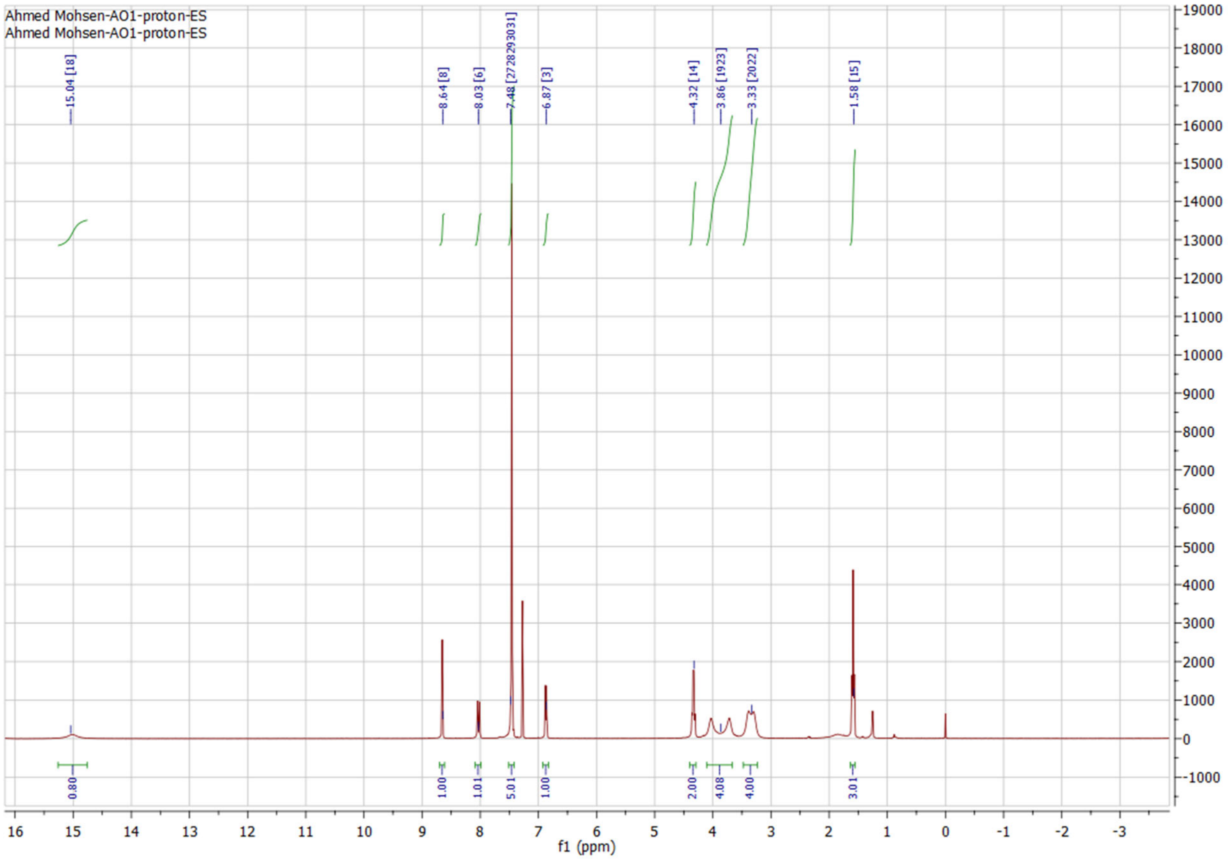

Figure S9: ^{1}H NMR of compound 7a.

7b

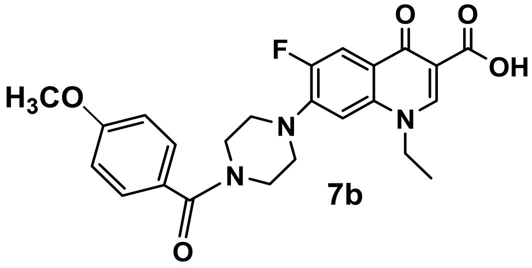

7b

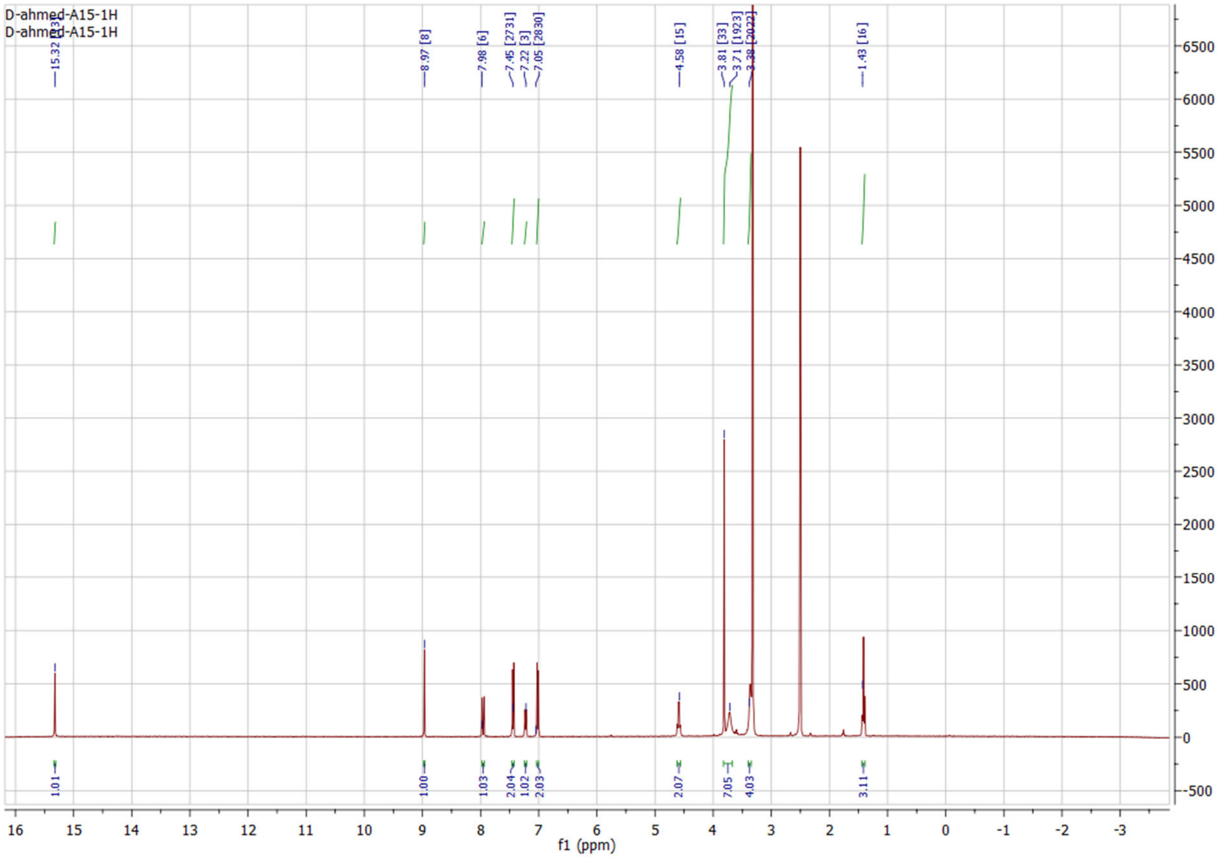

Figure S10: ^{1}H NMR of compound 7b.

7c

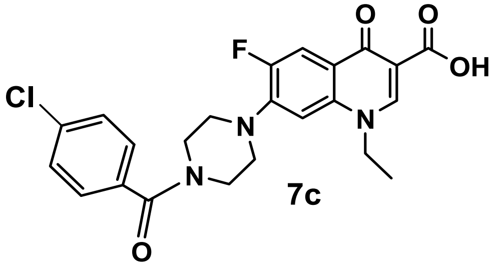

7c

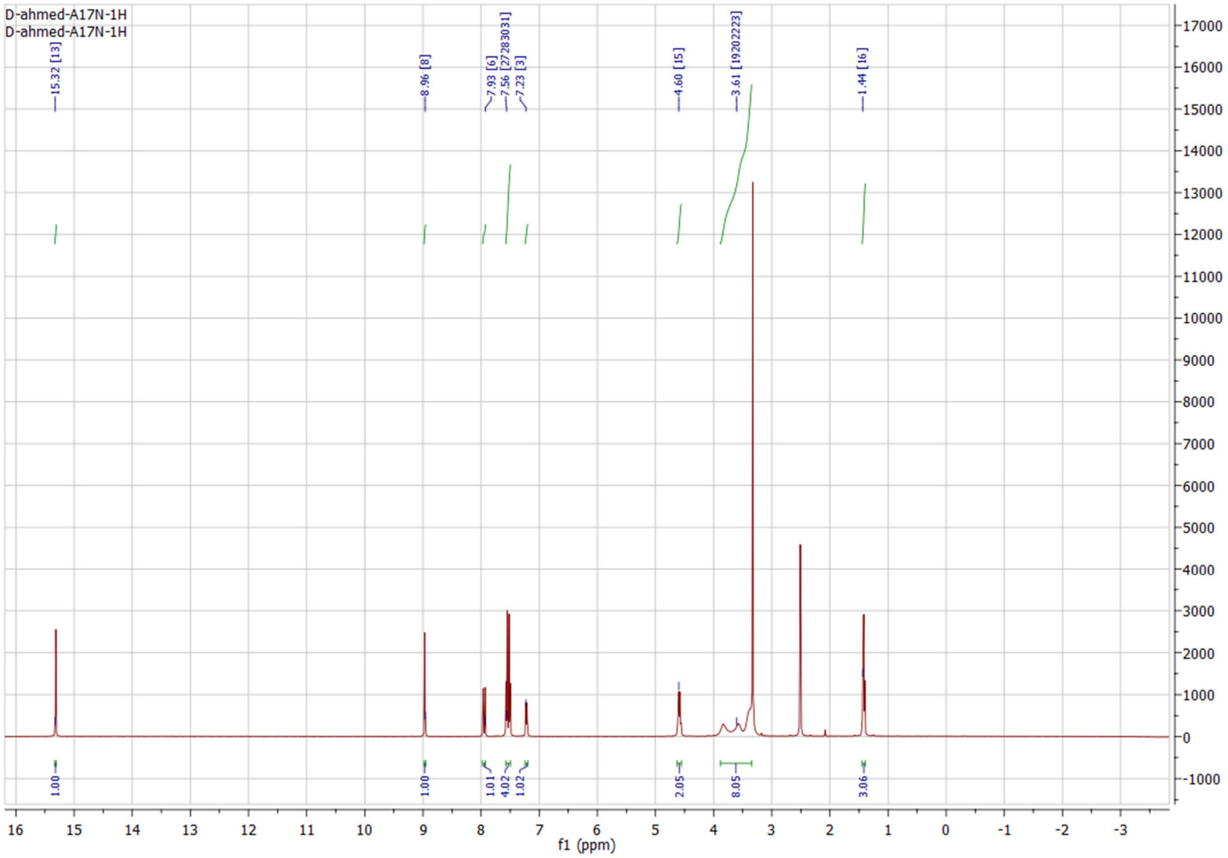

Figure S11: ^{1}H NMR of compound 7c.

7d

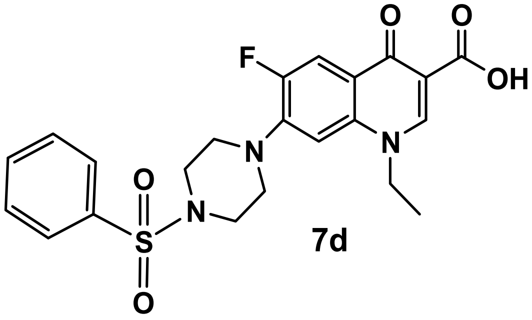

7d

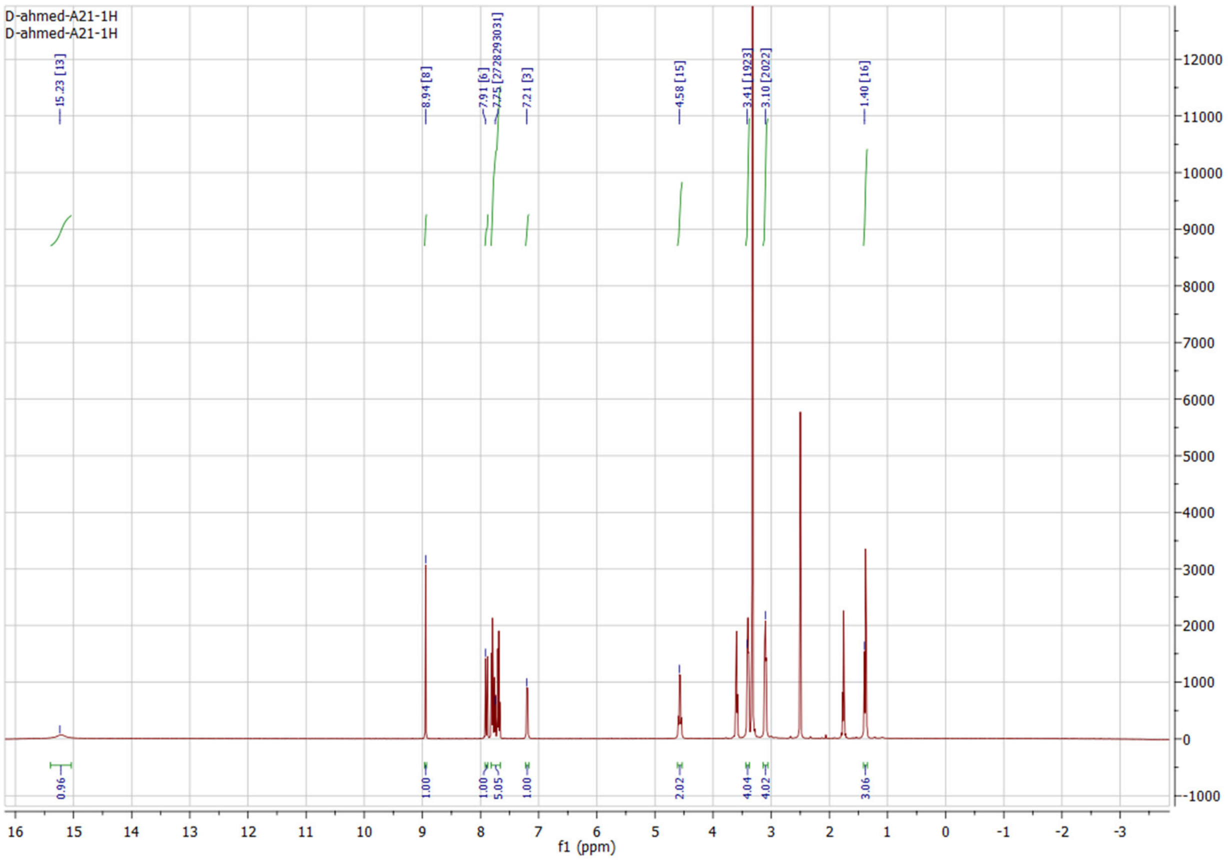

Figure S12: ^{1}H NMR of compound 7d.

7e

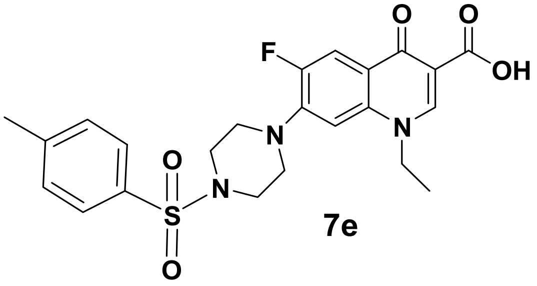

7e

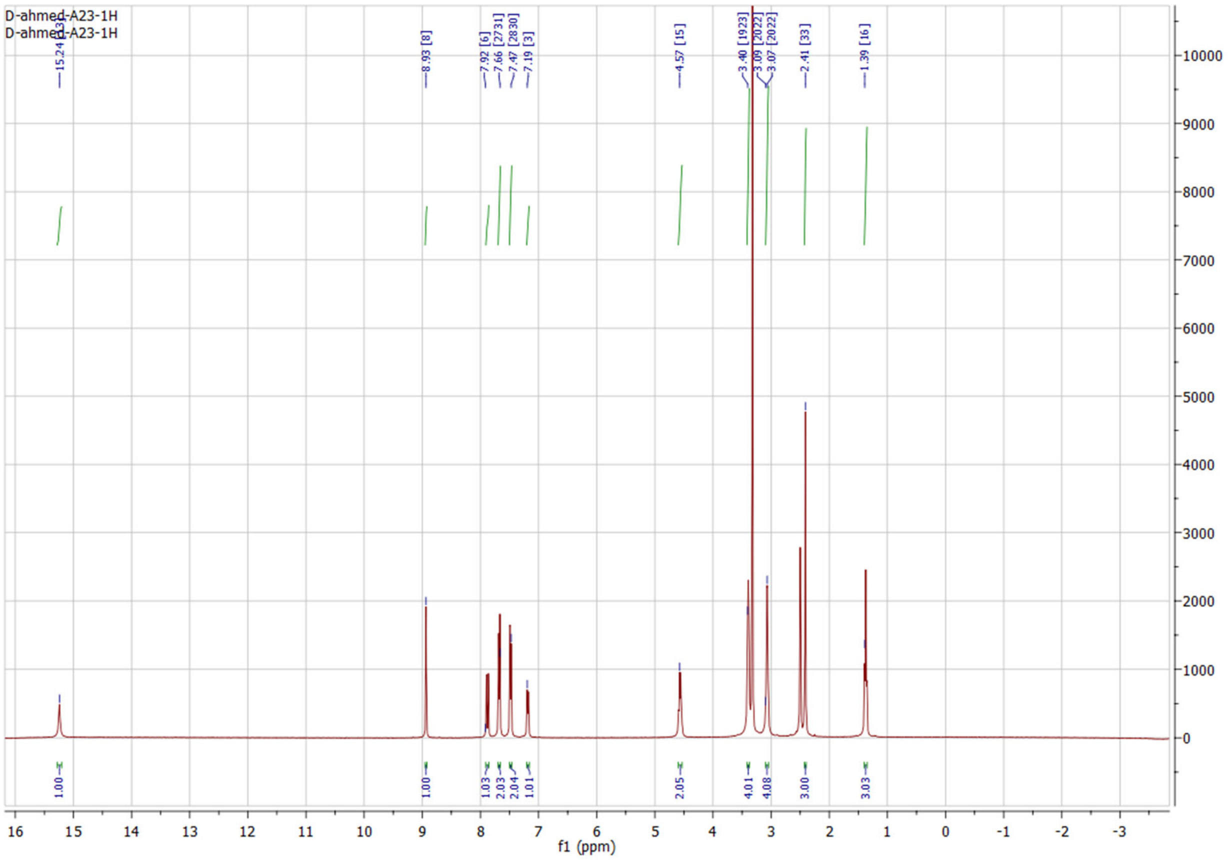

Figure S13: ^{1}H NMR of compound 7e.

8a

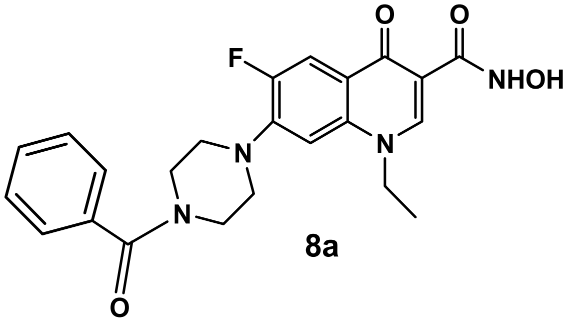

8a

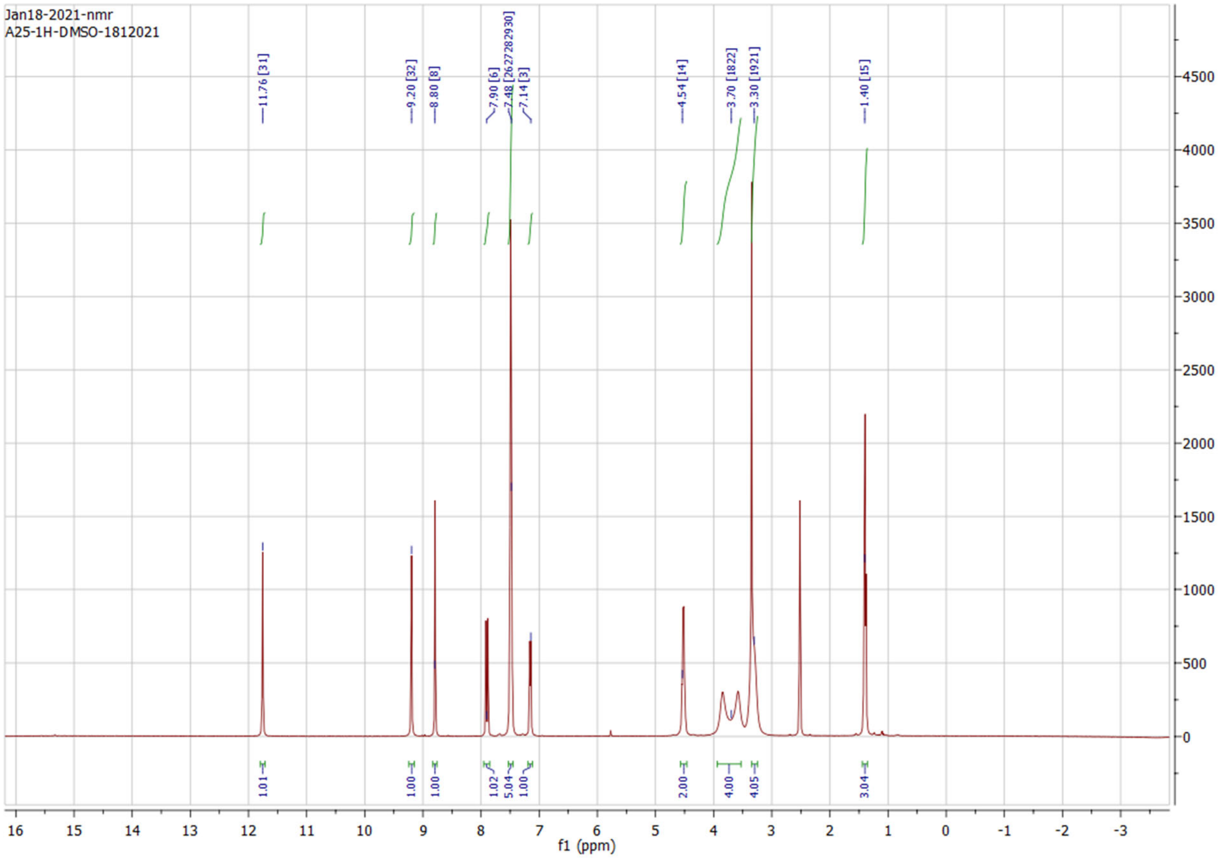

Figure S14: ^{1}H NMR of compound 8a.

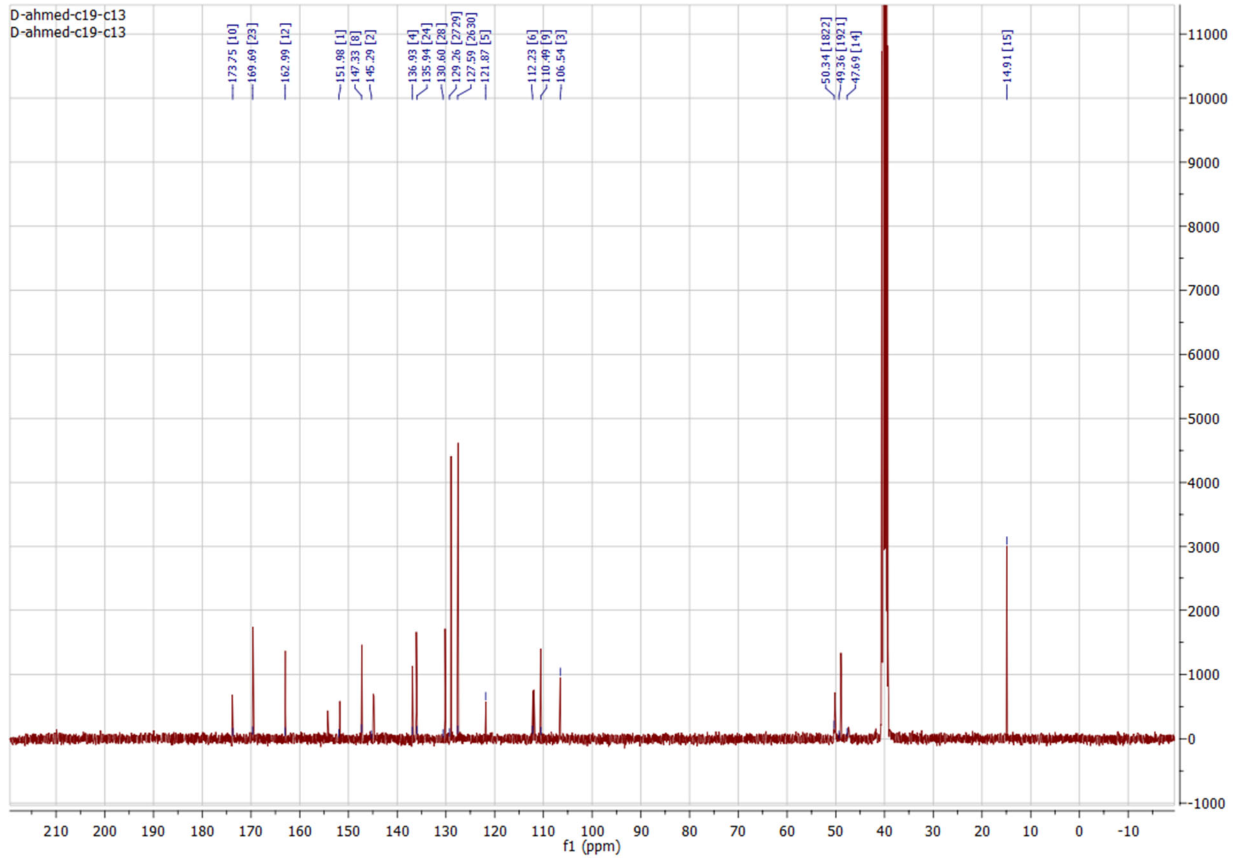

Figure S15: ^{13}C NMR of compound 8a.

8b

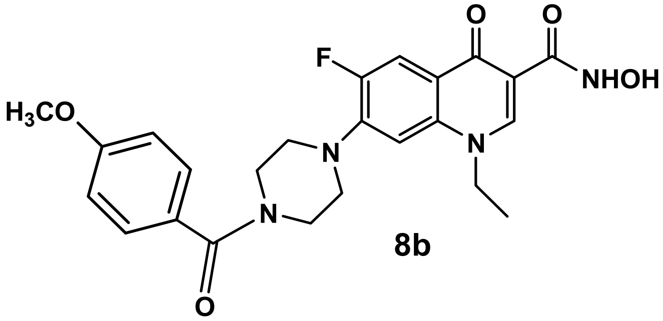

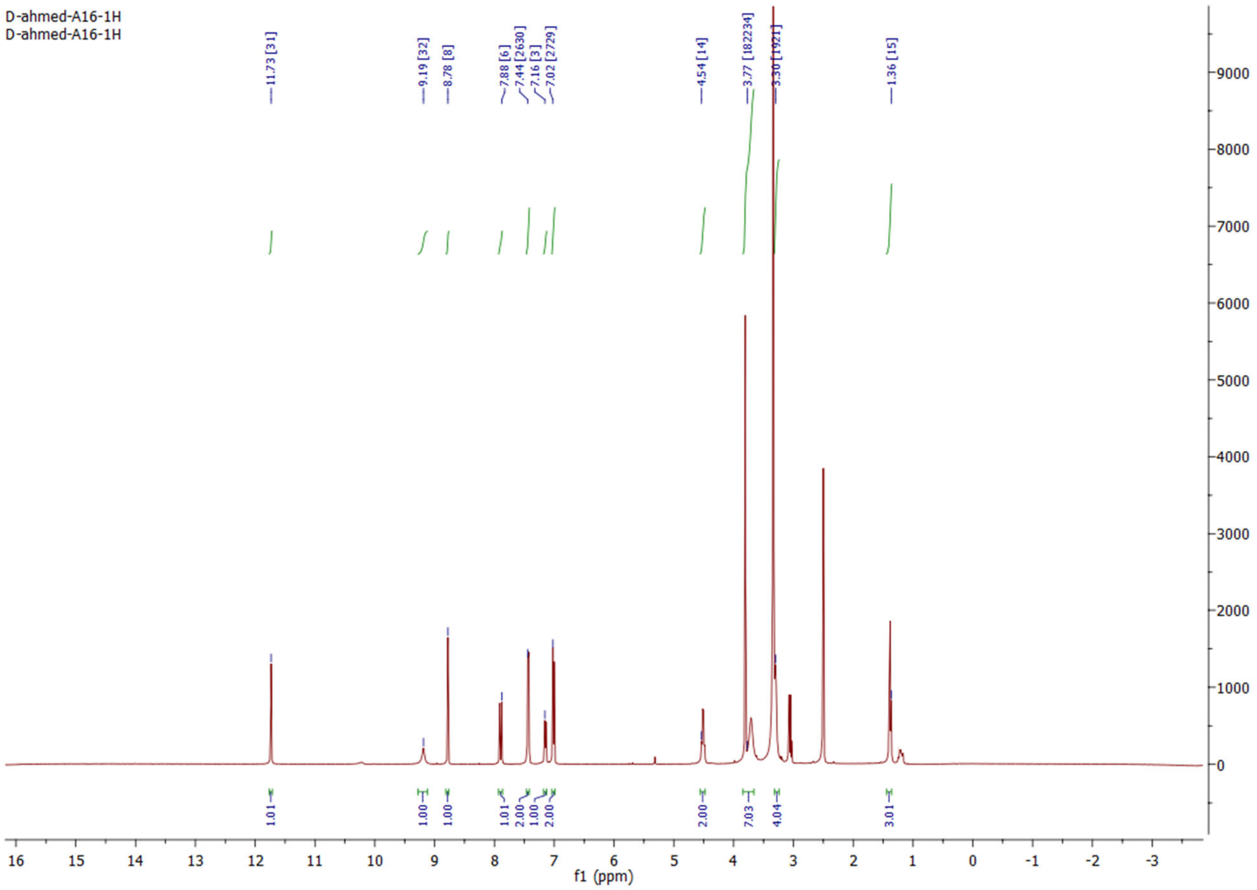

Figure S16: ^{1}H NMR of compound 8b.

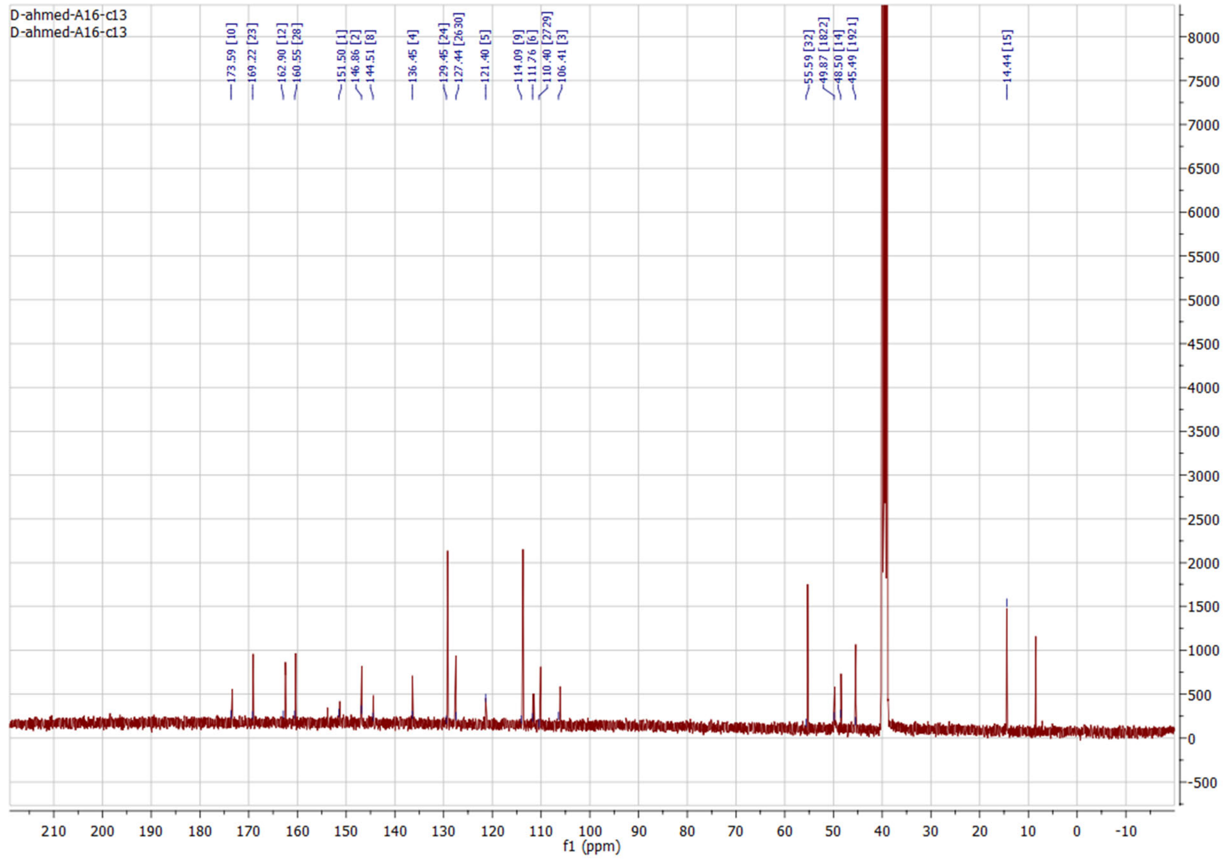

Figure S17: ^{13}C NMR of compound 8b.

8c

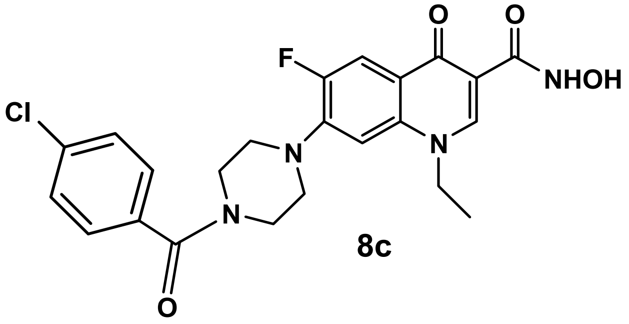

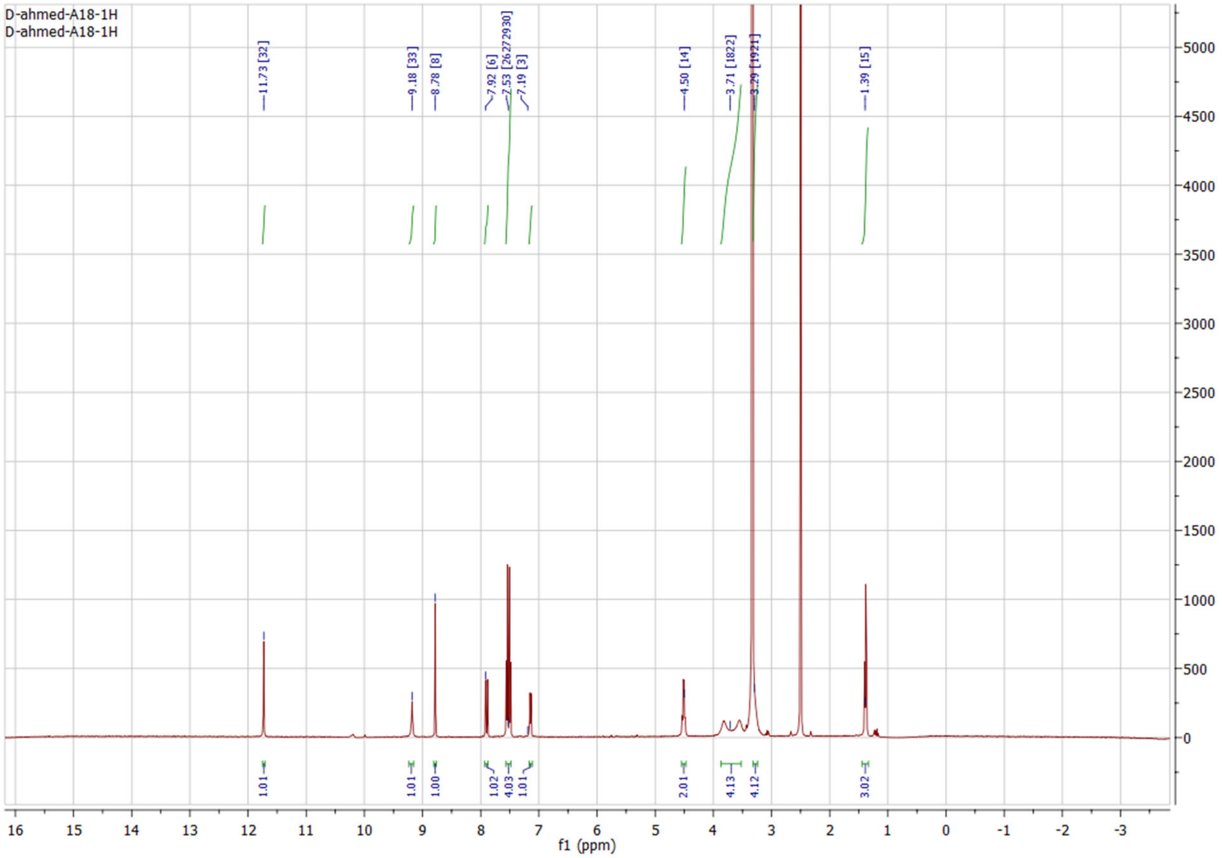

Figure S18: ^{1}H NMR of compound 8c.

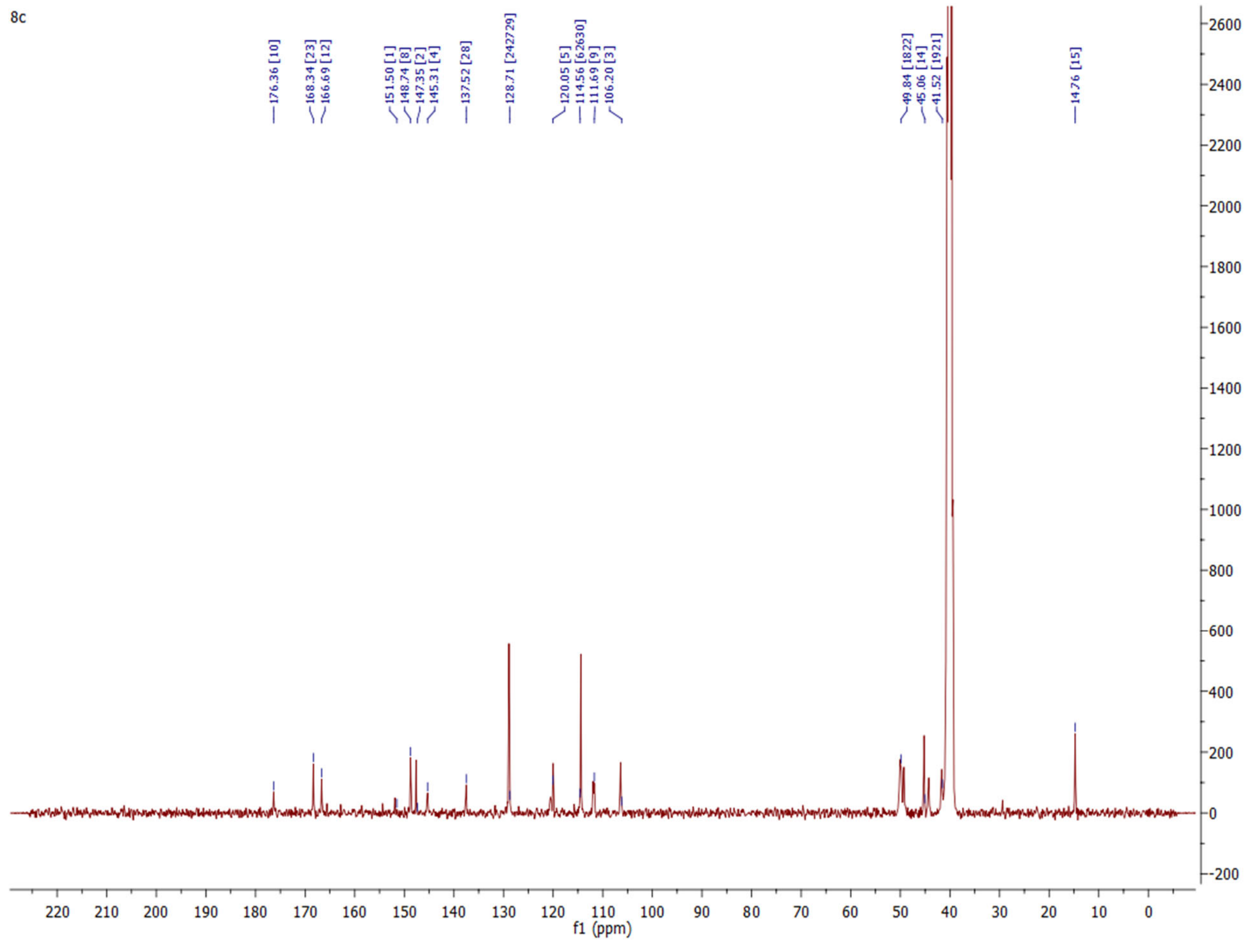

Figure S19: ^{13}C NMR of compound 8c.

8d

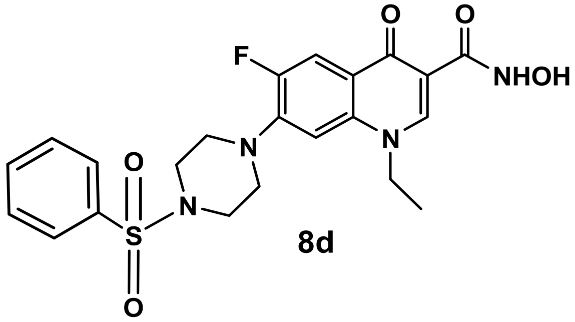

8d

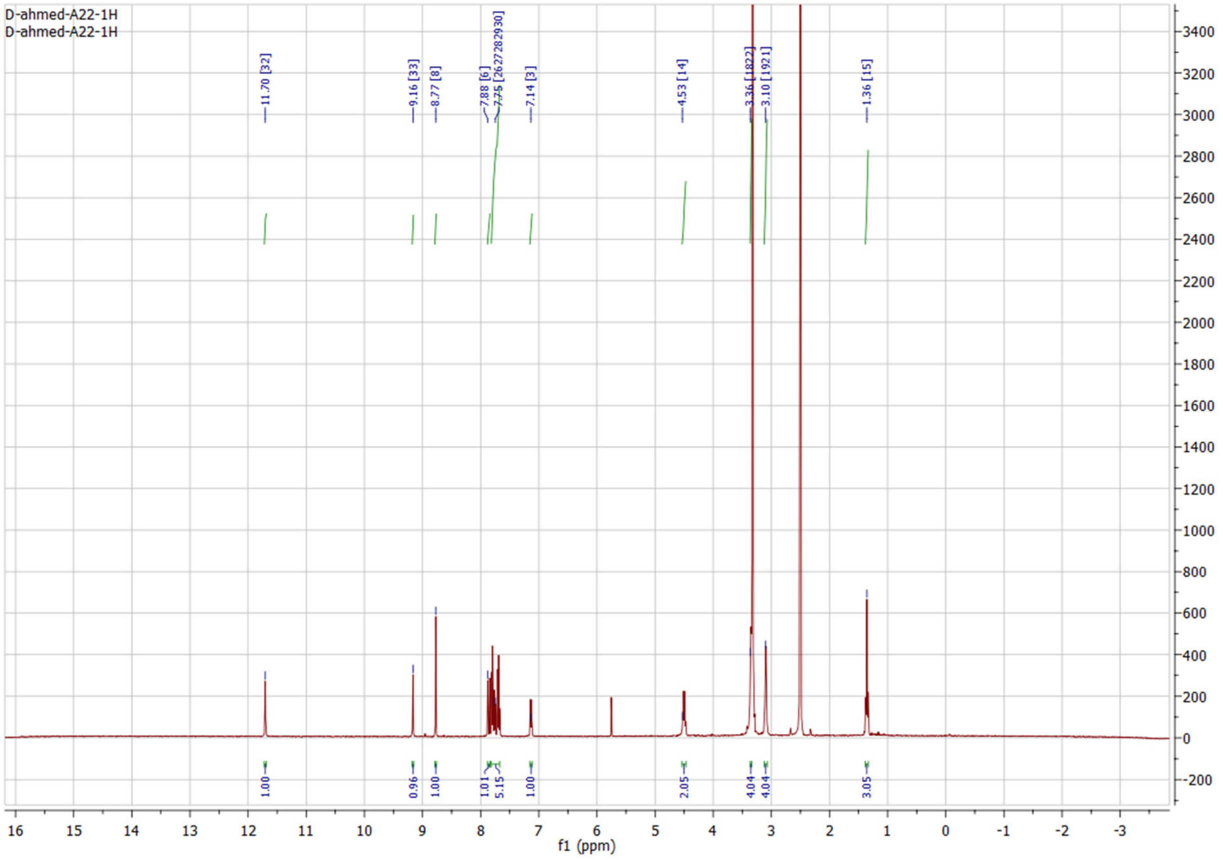

Figure S20: ^{1}H NMR of compound 8d.

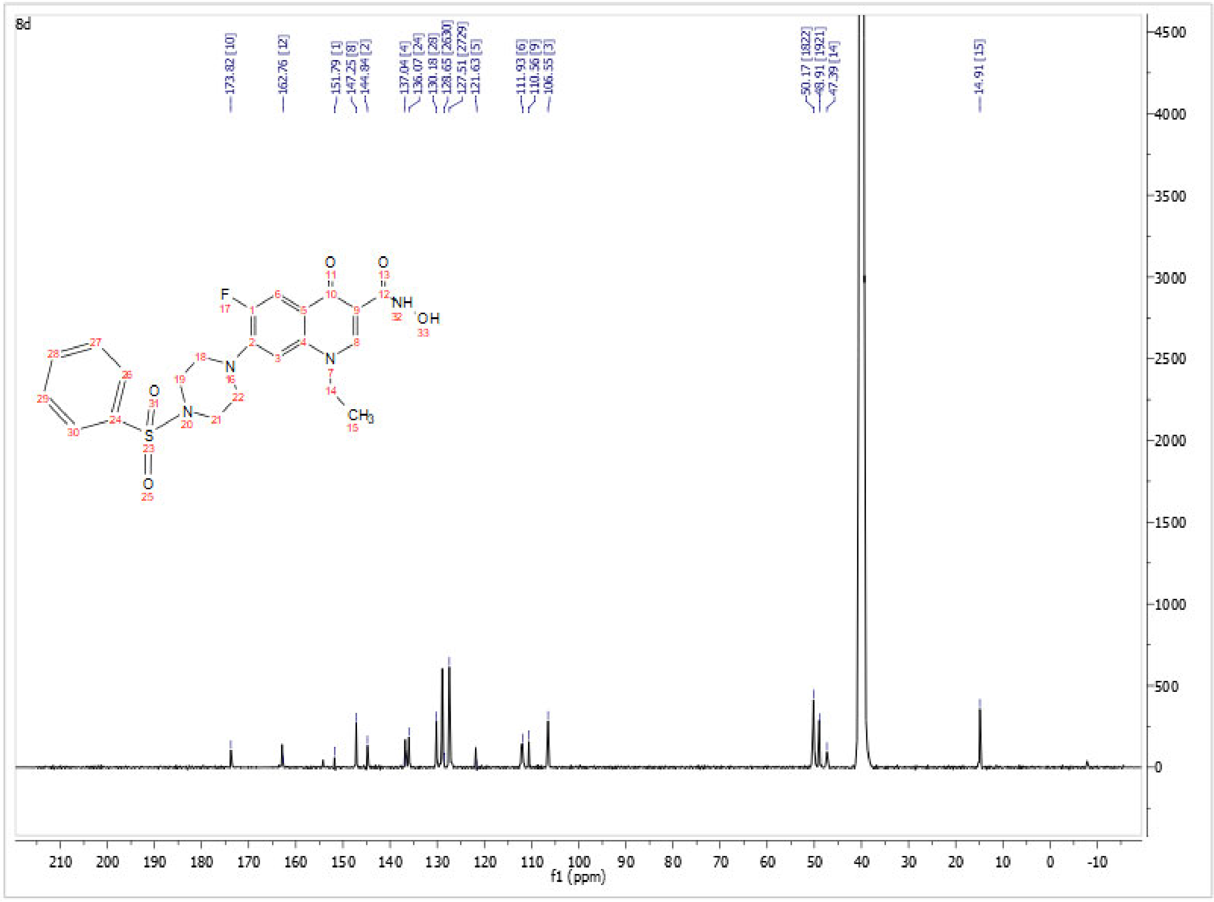

Figure S21: ^{13}C NMR of compound 8d.

8e

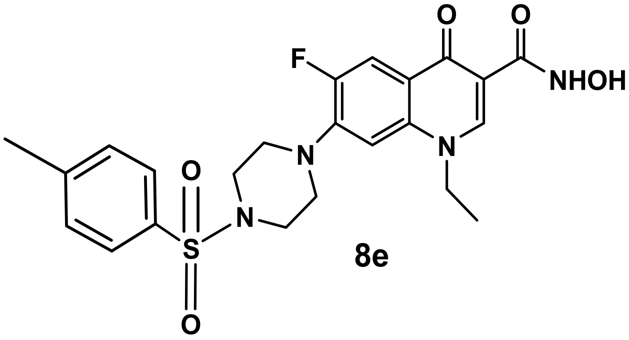

8e

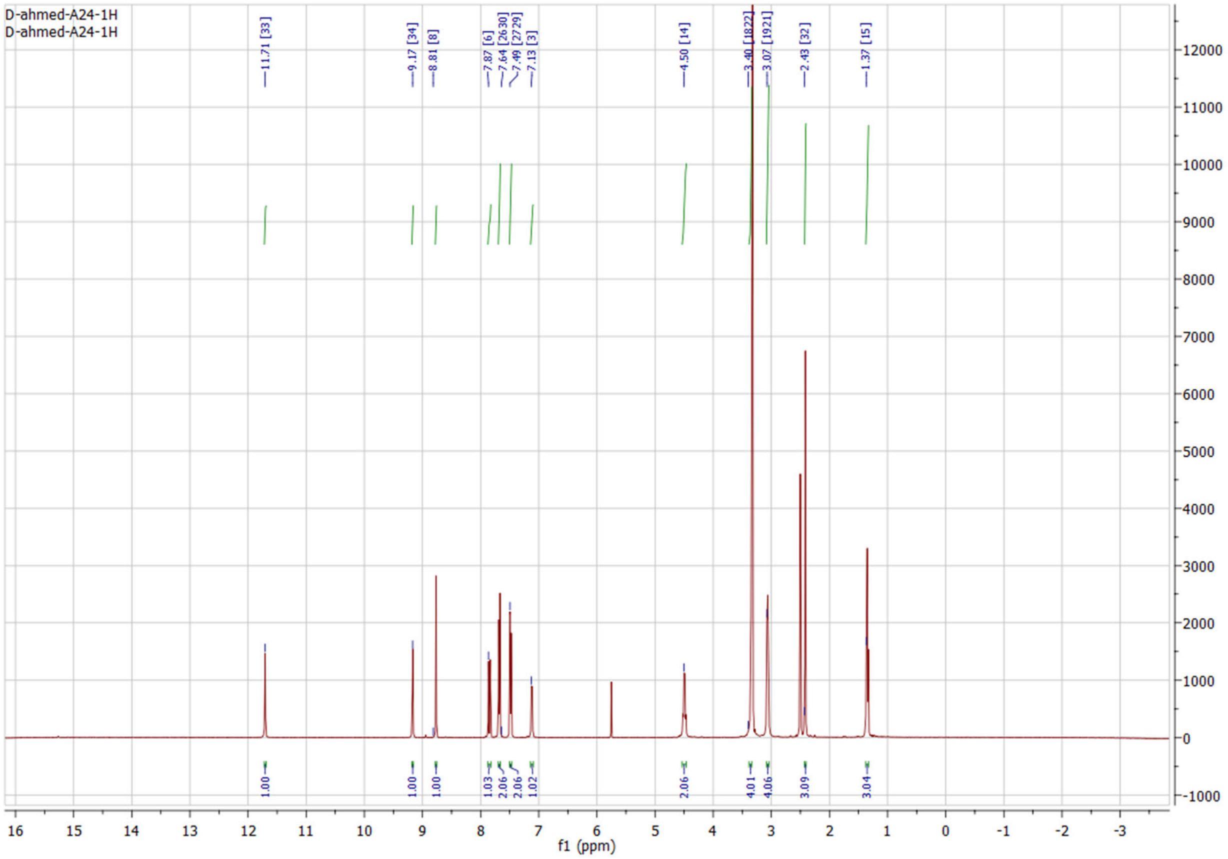

Figure S22: ^{1}H NMR of compound 8e.

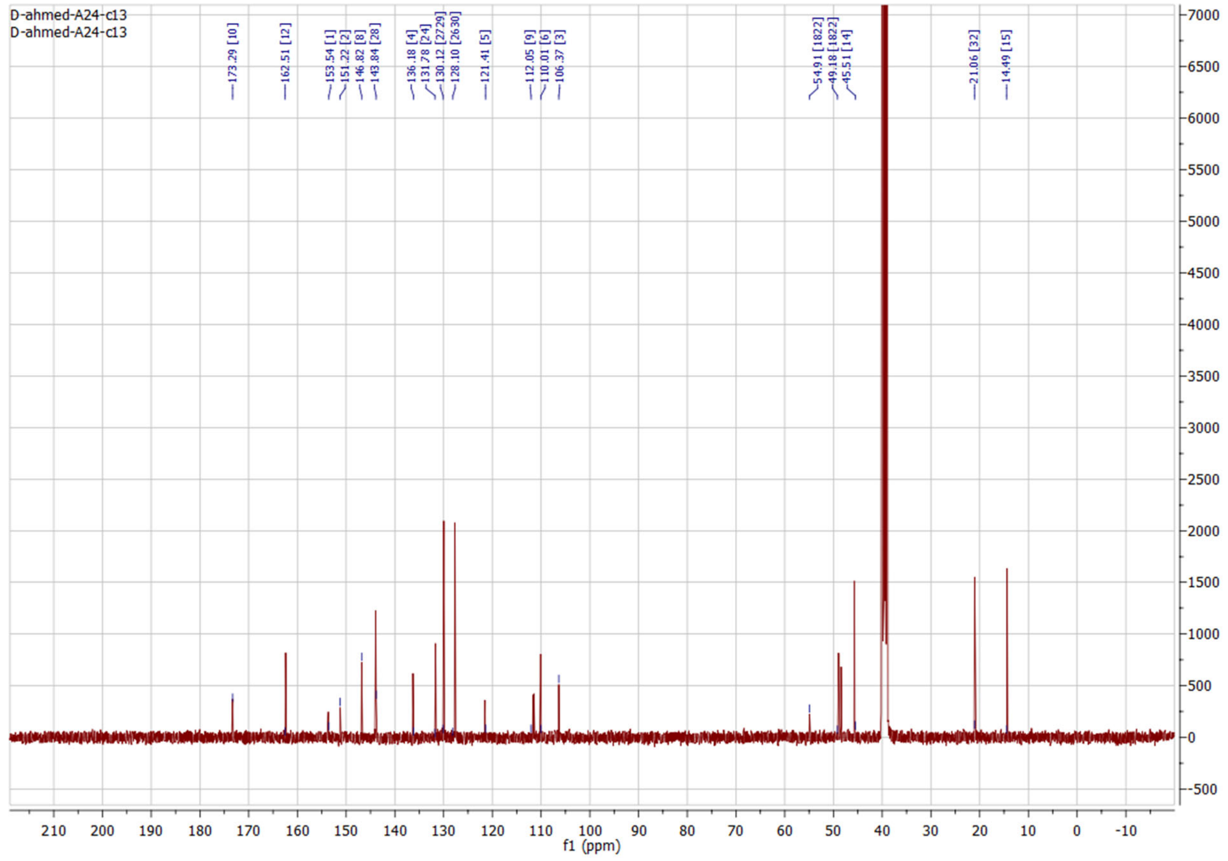

Figure S23: ^{13}C NMR of compound 8e.

10a

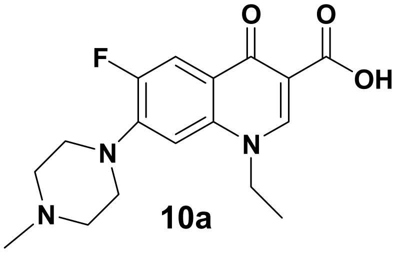

10a

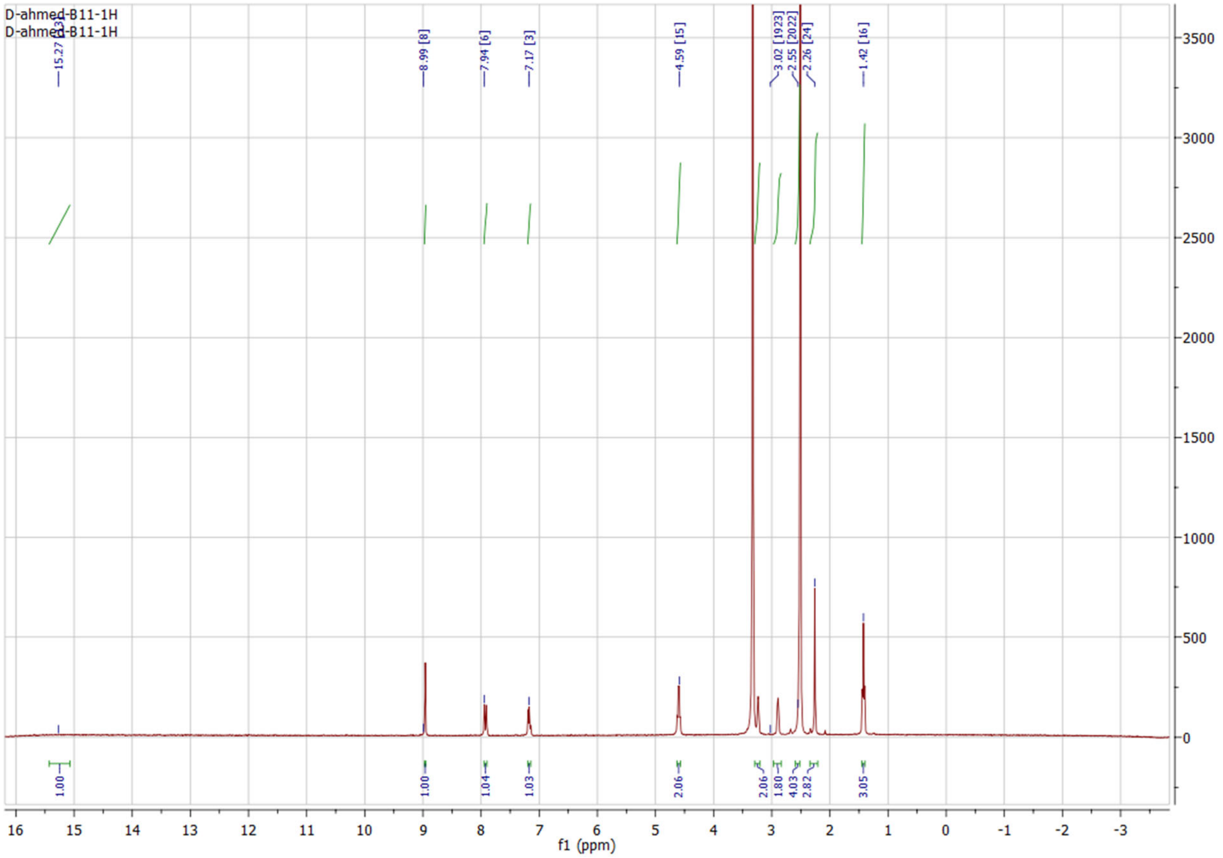

Figure S24: ^{1}H NMR of compound 10a.

10b

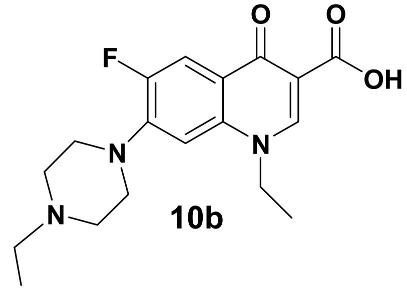

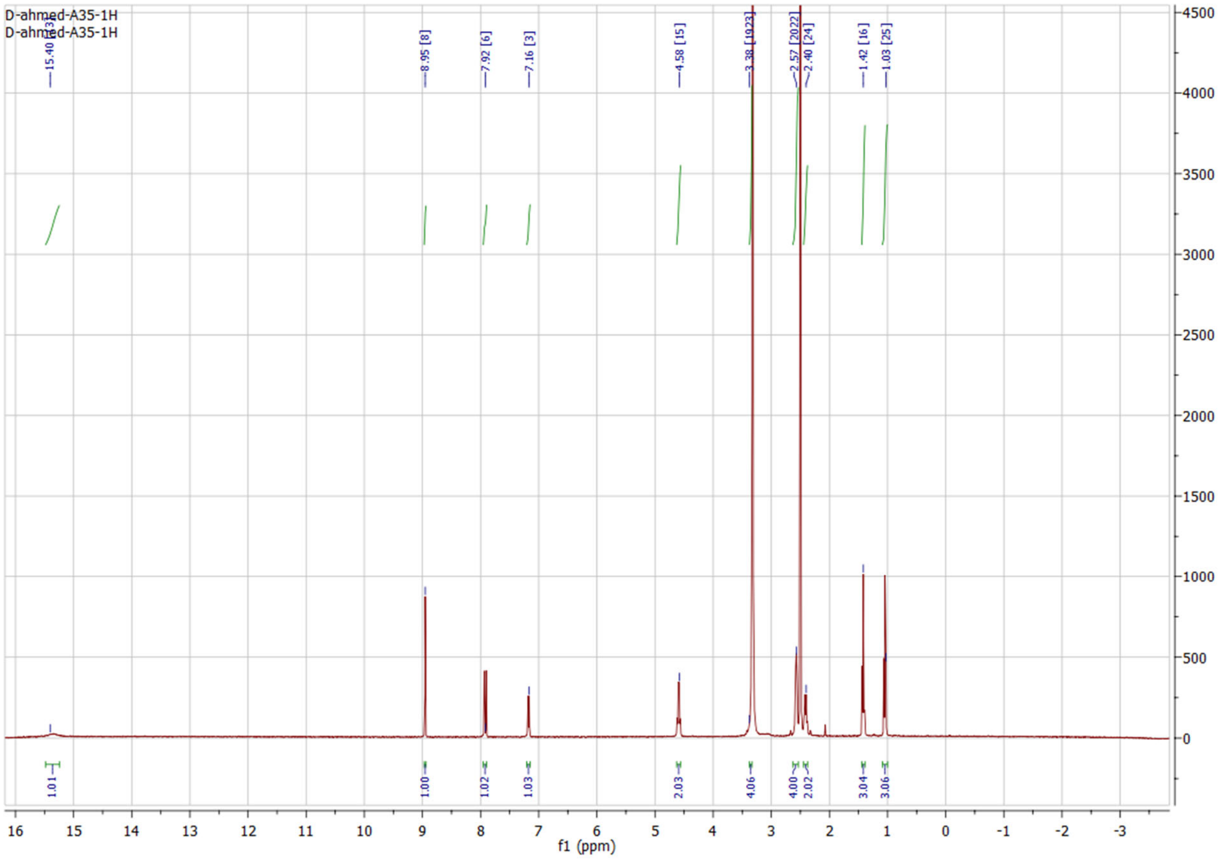

Figure S25: ^{1}H NMR of compound 10b.

10c

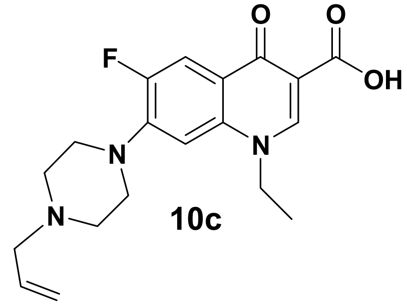

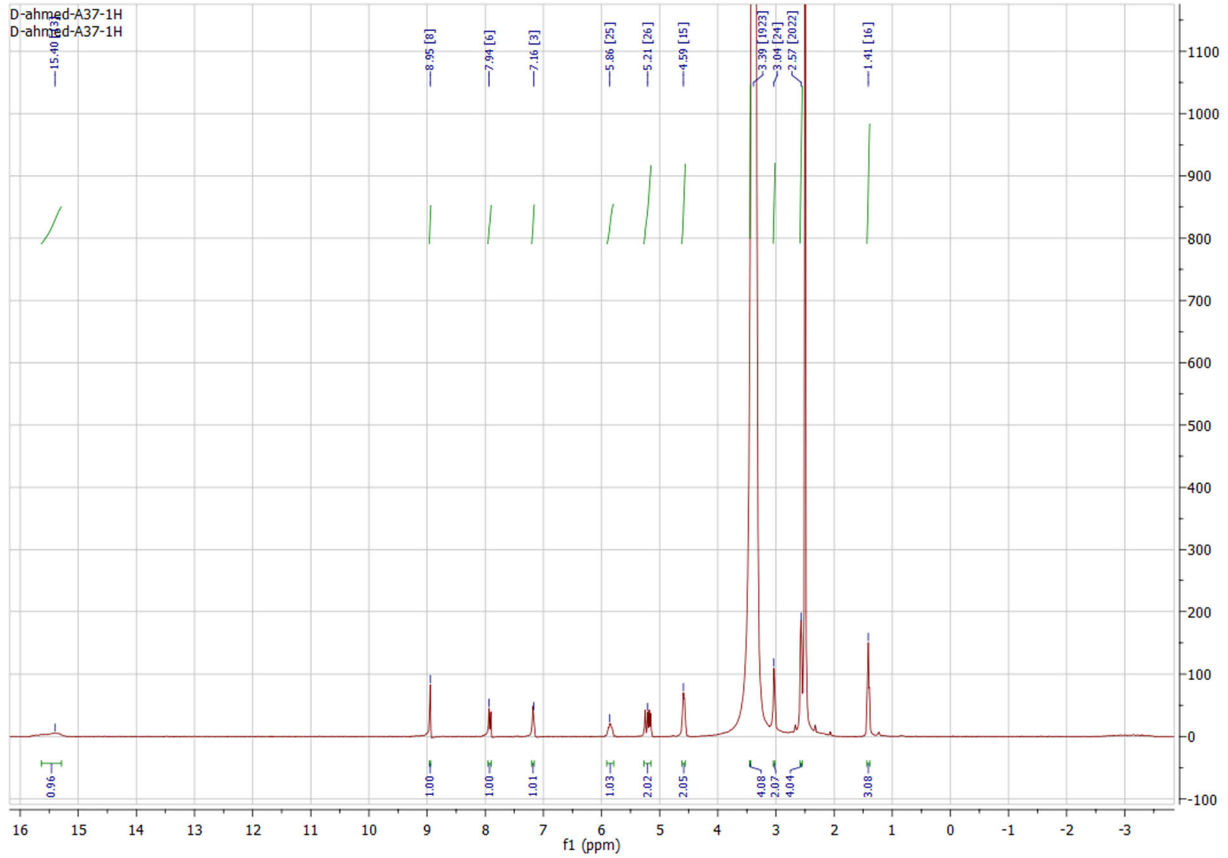

Figure S26: ^{1}H NMR of compound 10c.

10e

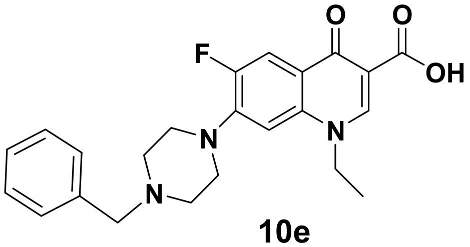

10e

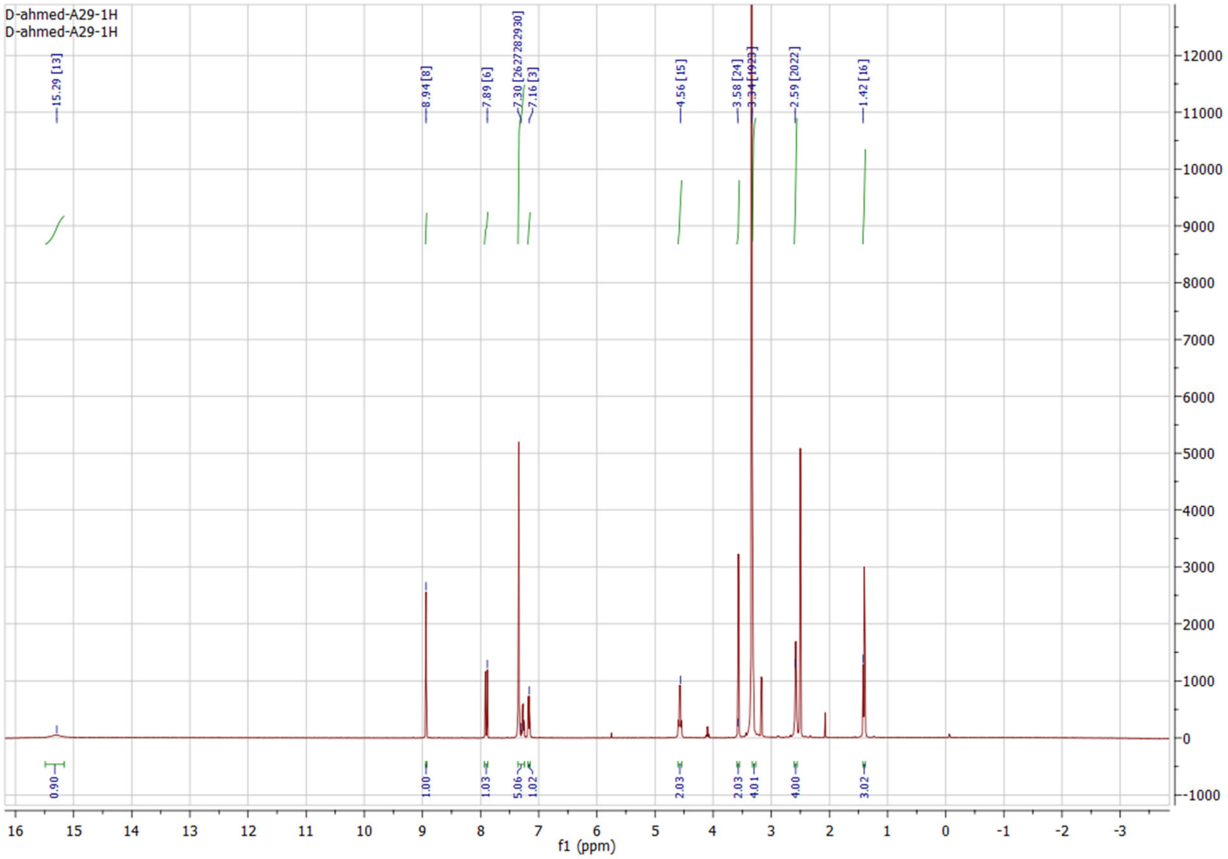

Figure S27: ^{1}H NMR of compound 10e.

10f

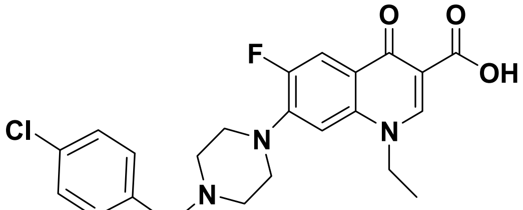

10f

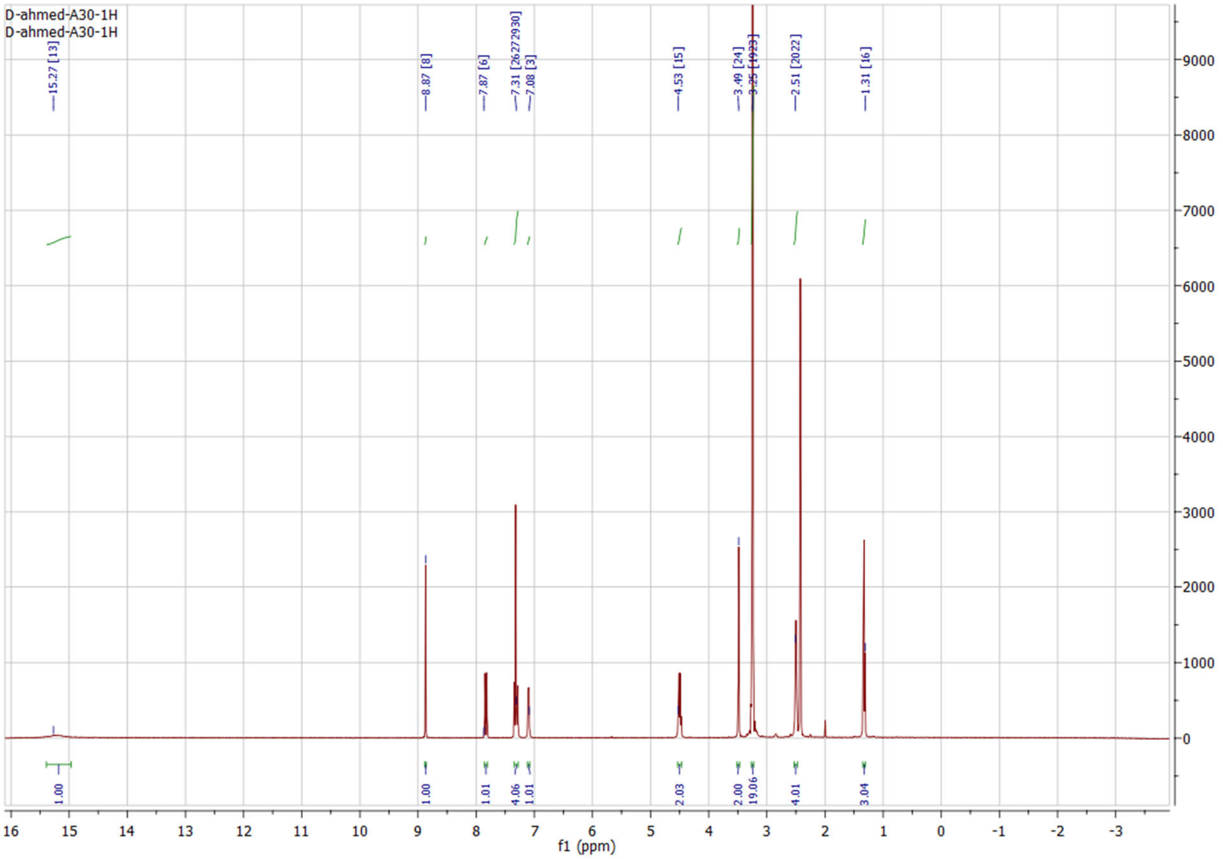

Figure S28: ^{1}H NMR of compound 10f.

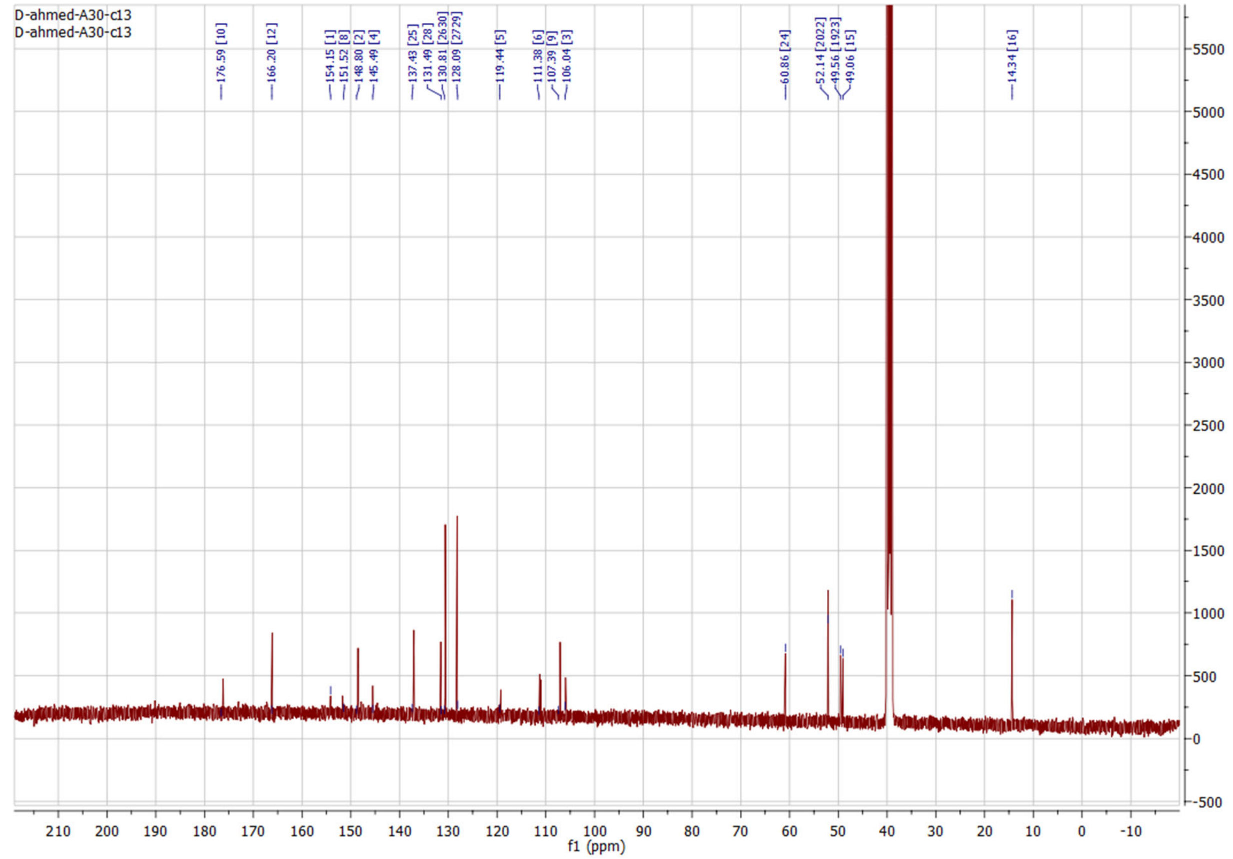

Figure S29: ^{13}C NMR of compound 10f.

11a

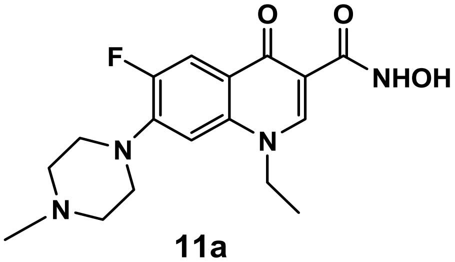

11a

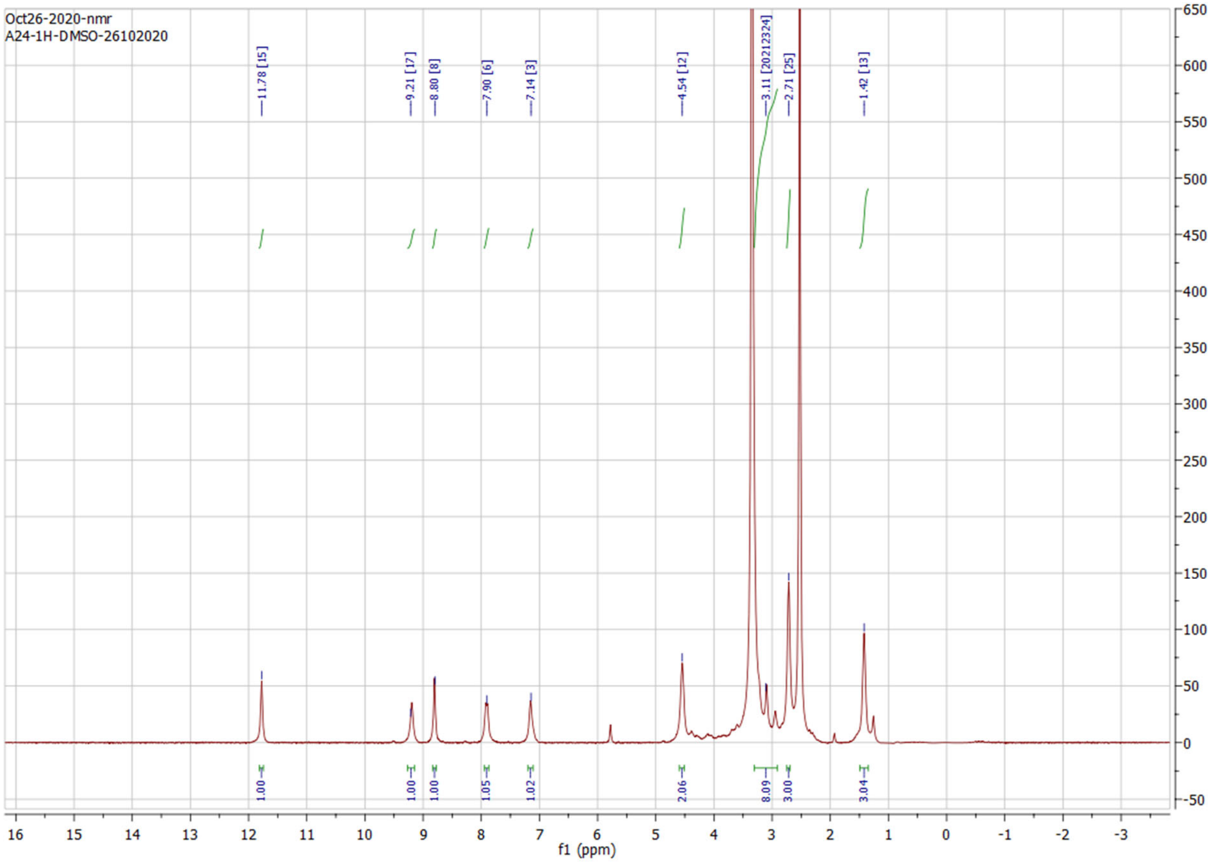

Figure S30: ^{1}H NMR of compound 11a.

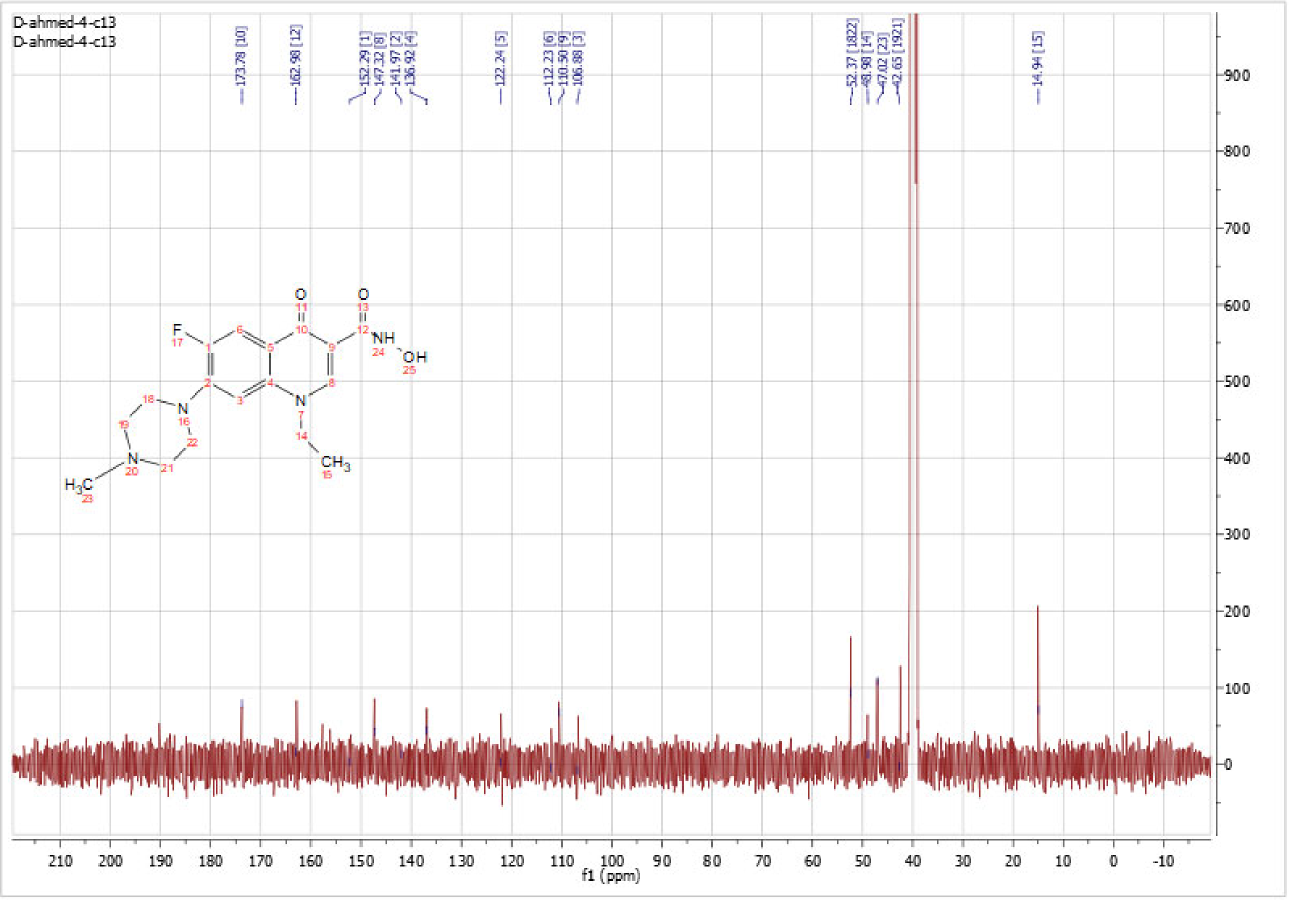

Figure S31: ^{1}H NMR of compound 11a.

11b

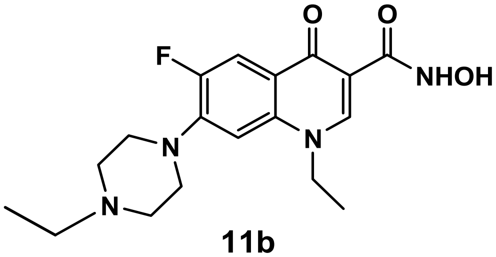

11b

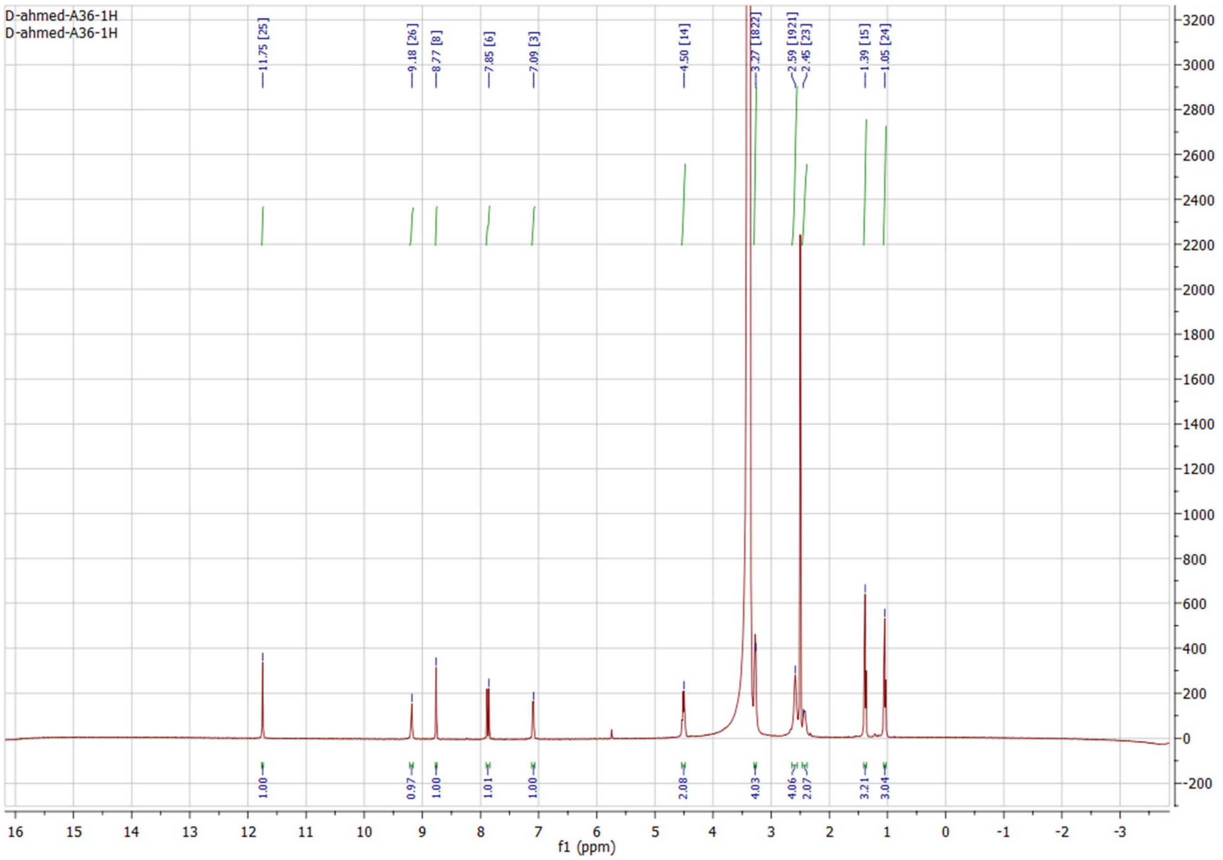

Figure S32: ^{1}H NMR of compound 11b.

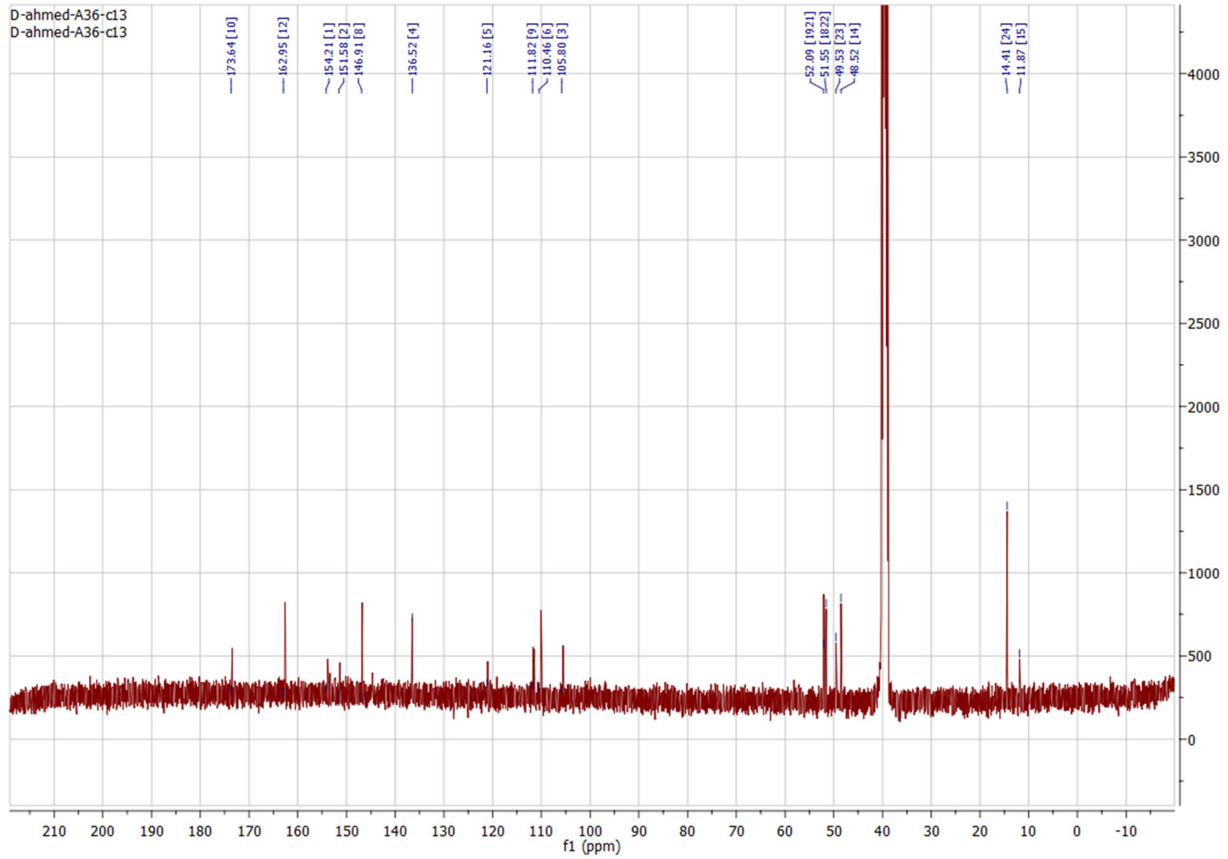

Figure S33: ^{13}C NMR of compound 11b.

11c

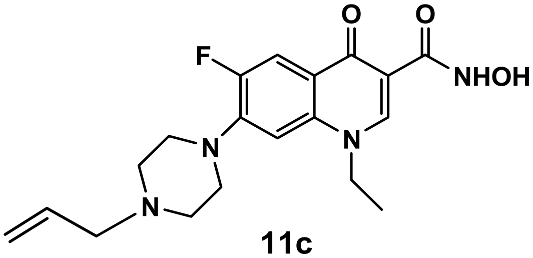

11c

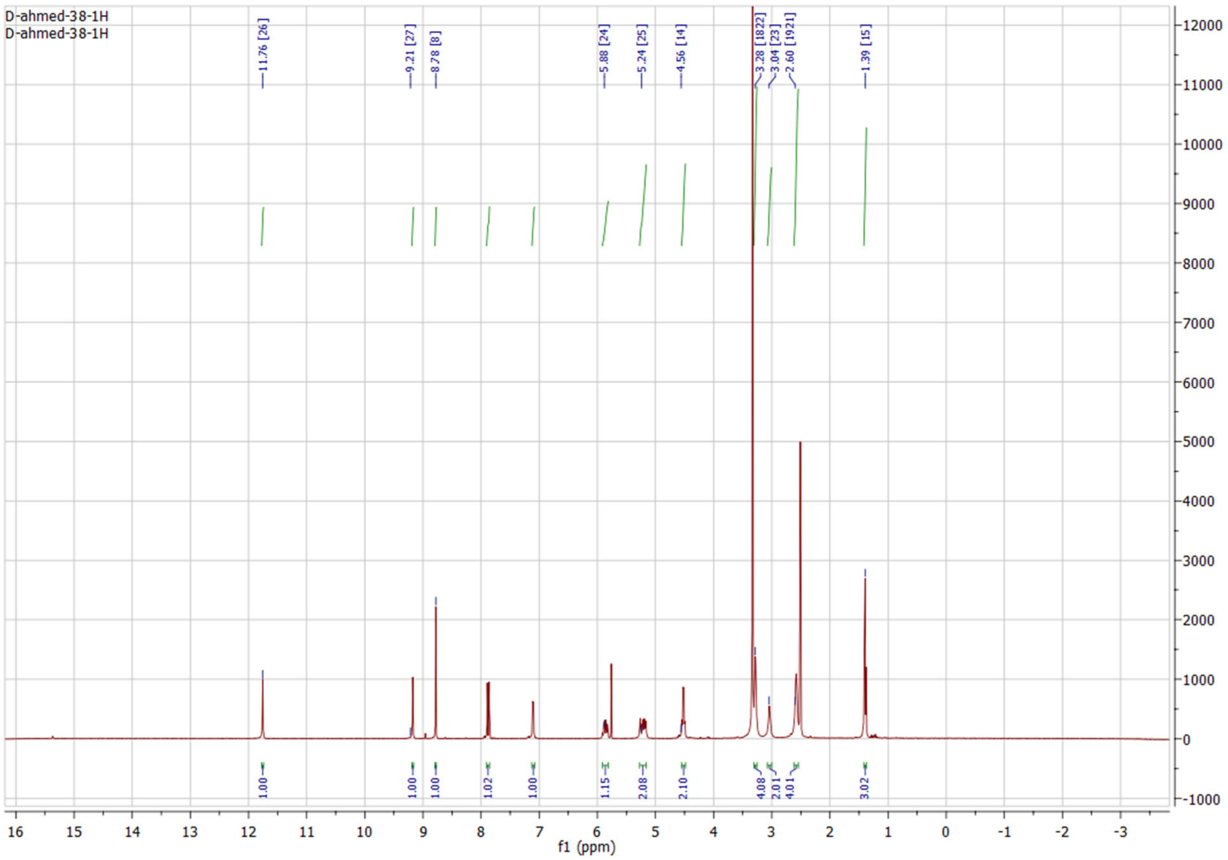

Figure S34: ^{1}H NMR of compound 11c.

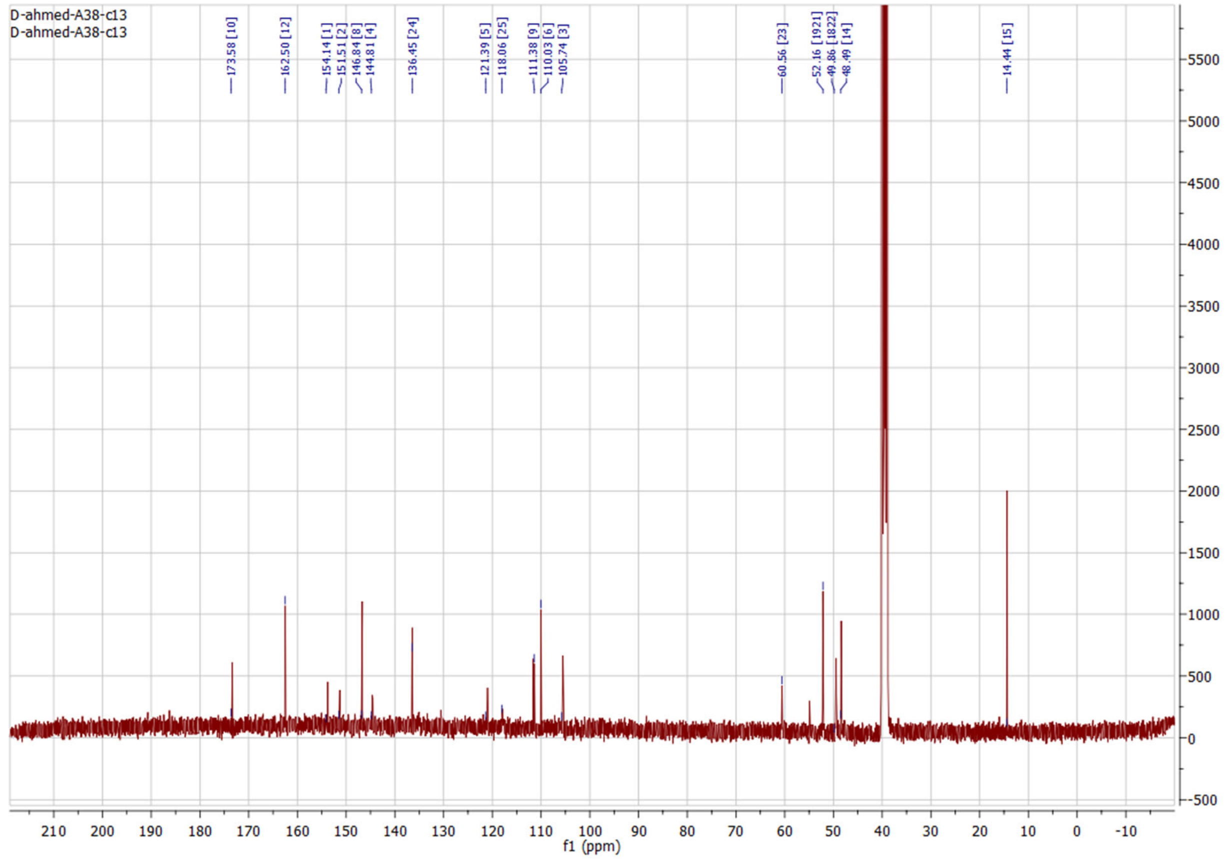

Figure S35: ^{13}C NMR of compound 11c.

10d

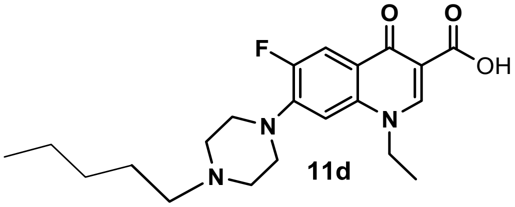
11d

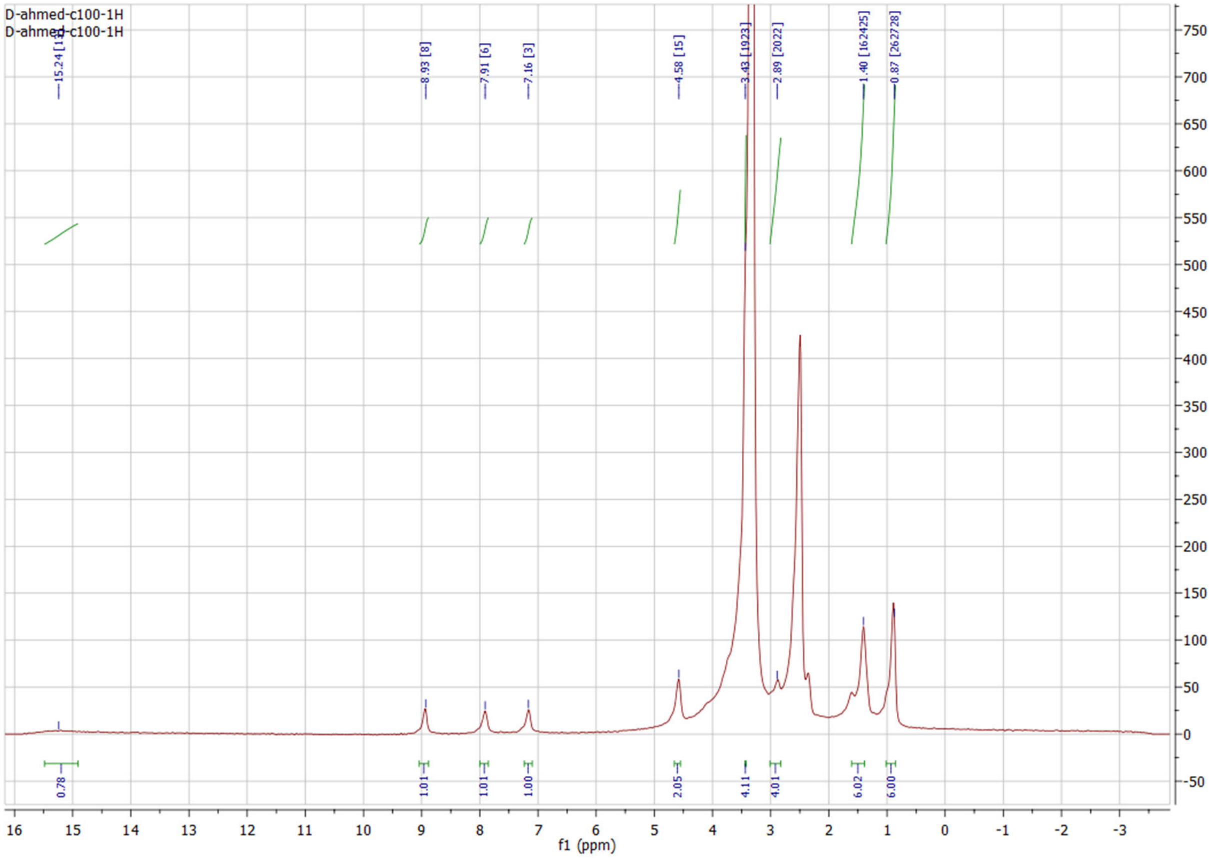

Figure S36: ^{1}H NMR of compound 10d.

11d

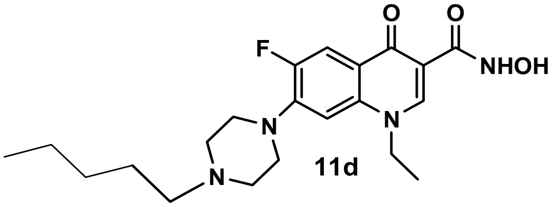

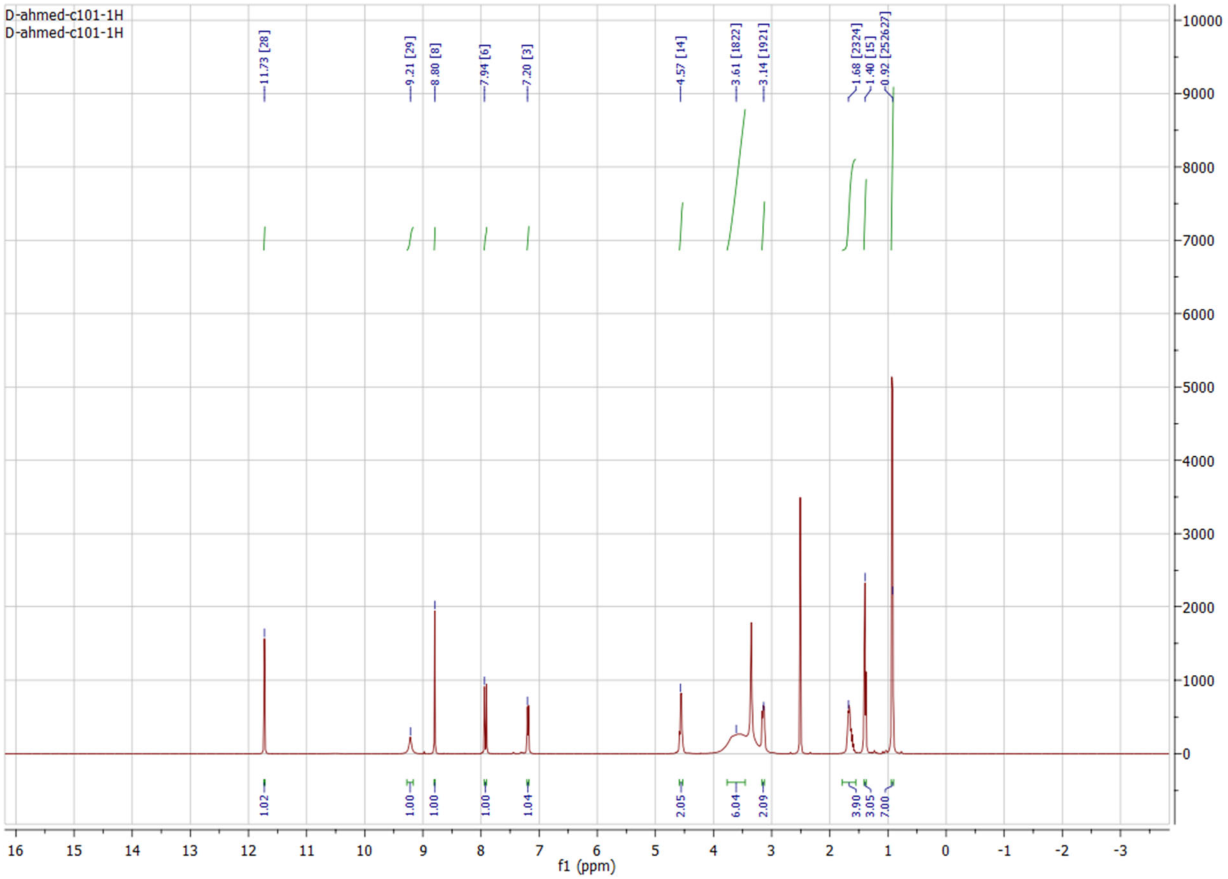

Figure S37: ^{1}H NMR of compound 11d.

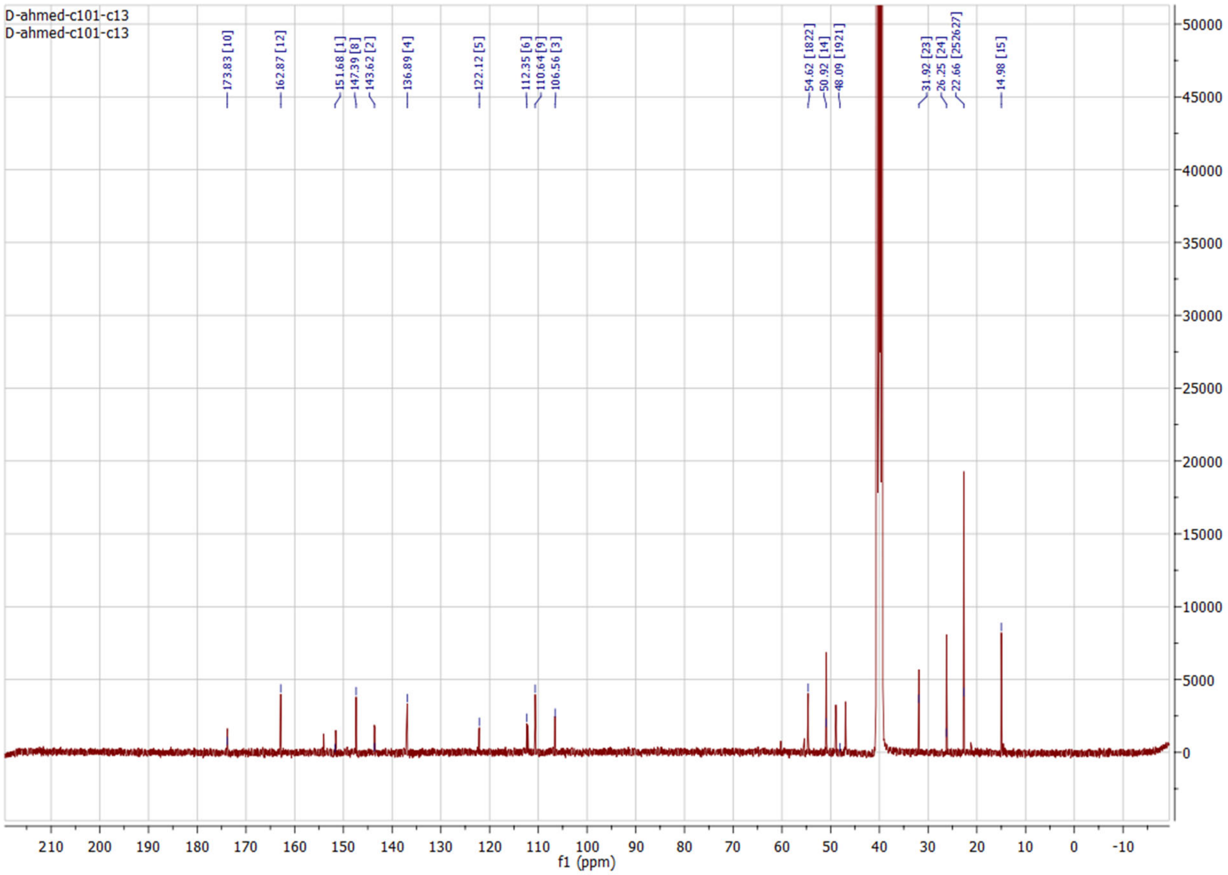

Figure S38: ^{13}C NMR of compound 11d.

11e

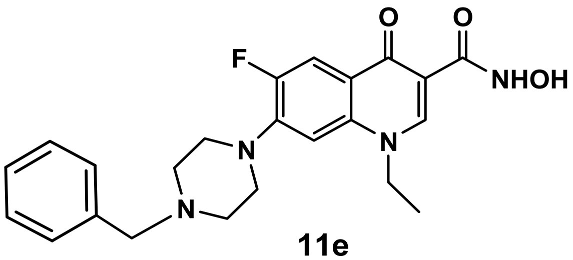

11e

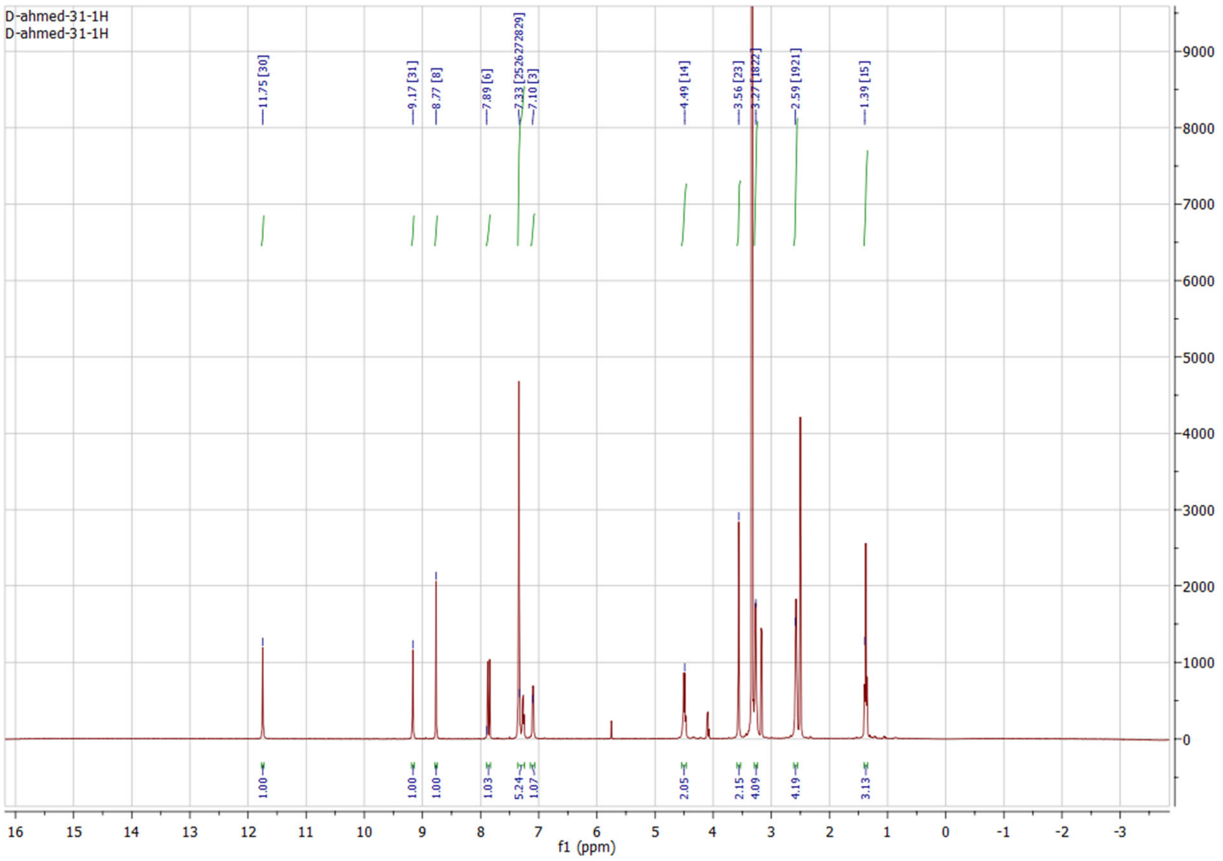

Figure S39: ^{1}H NMR of compound 11e.

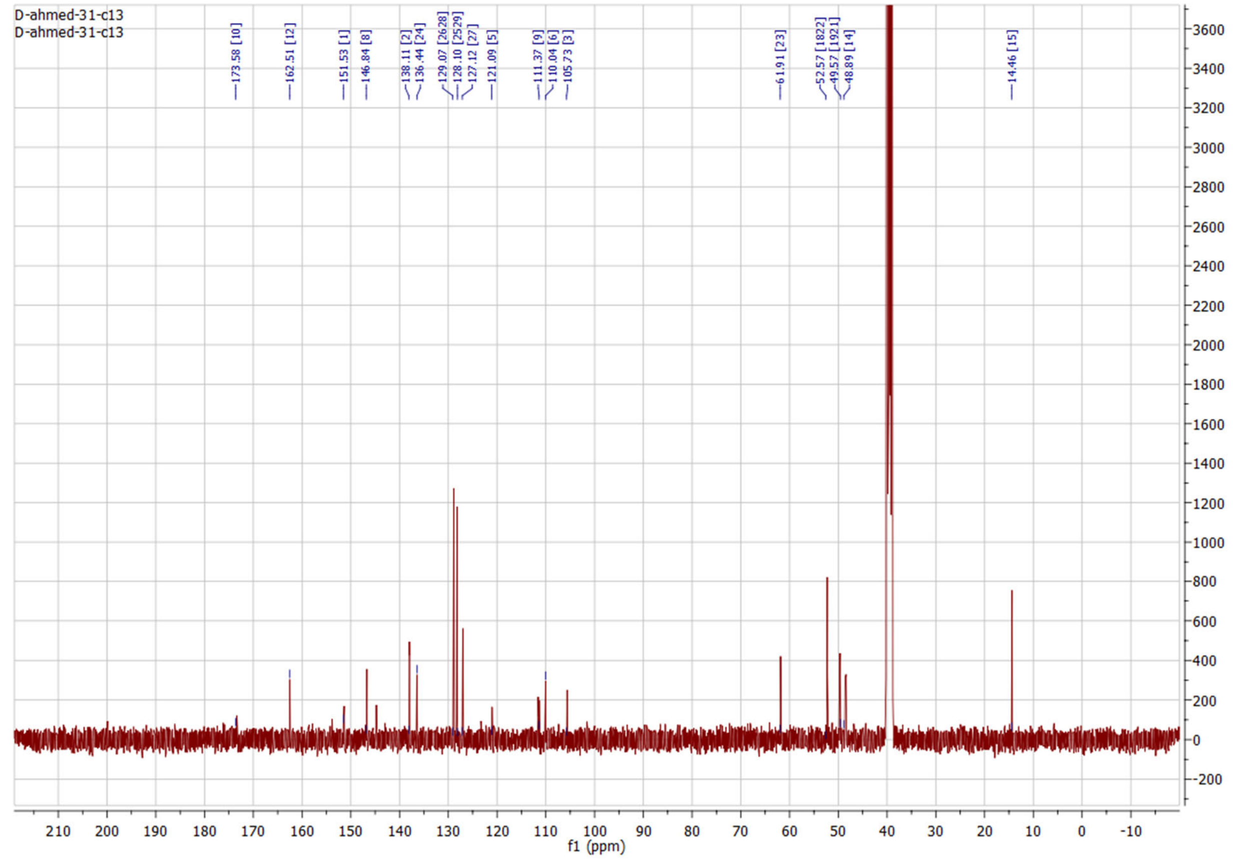

Figure S40: ^{13}C NMR of compound 11e.

11f

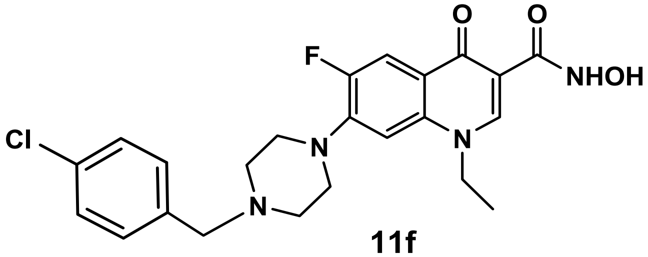

11f

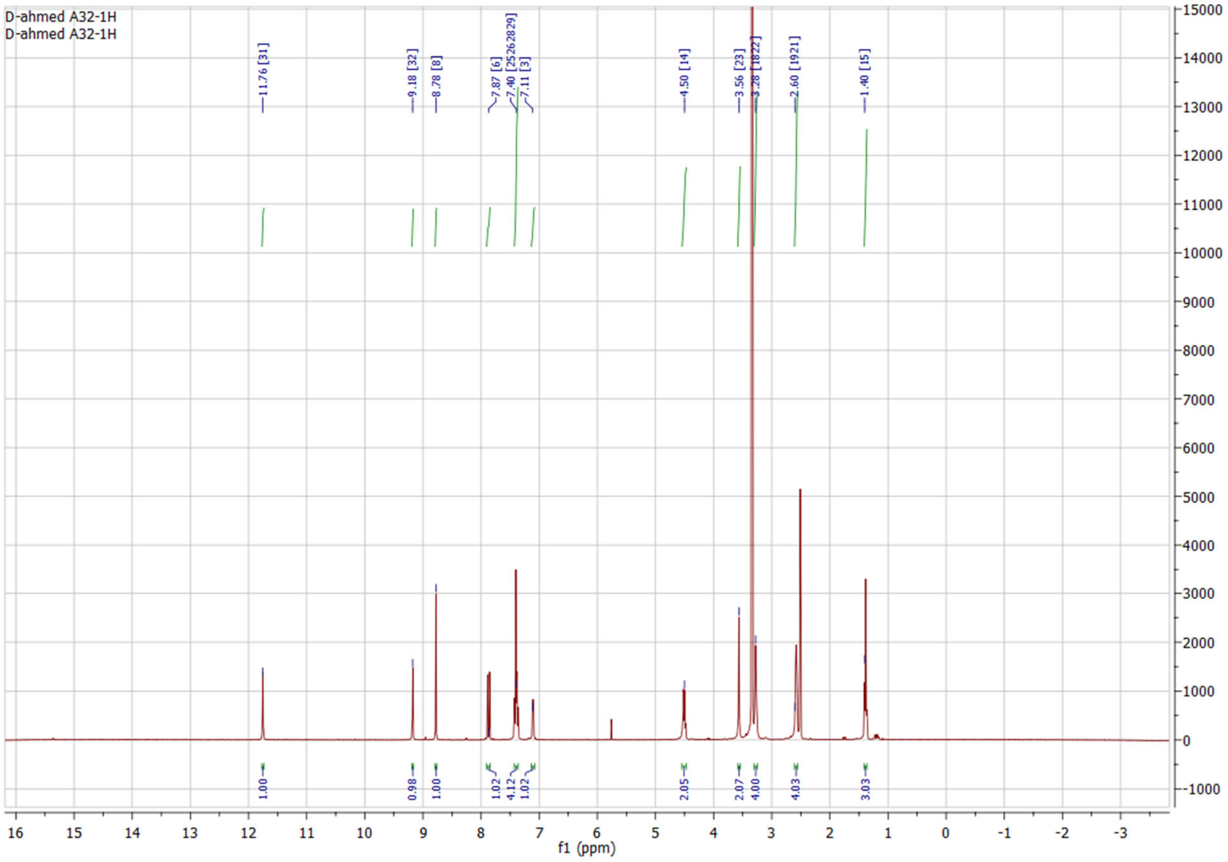

Figure S41: ^{1}H NMR of compound 11f.

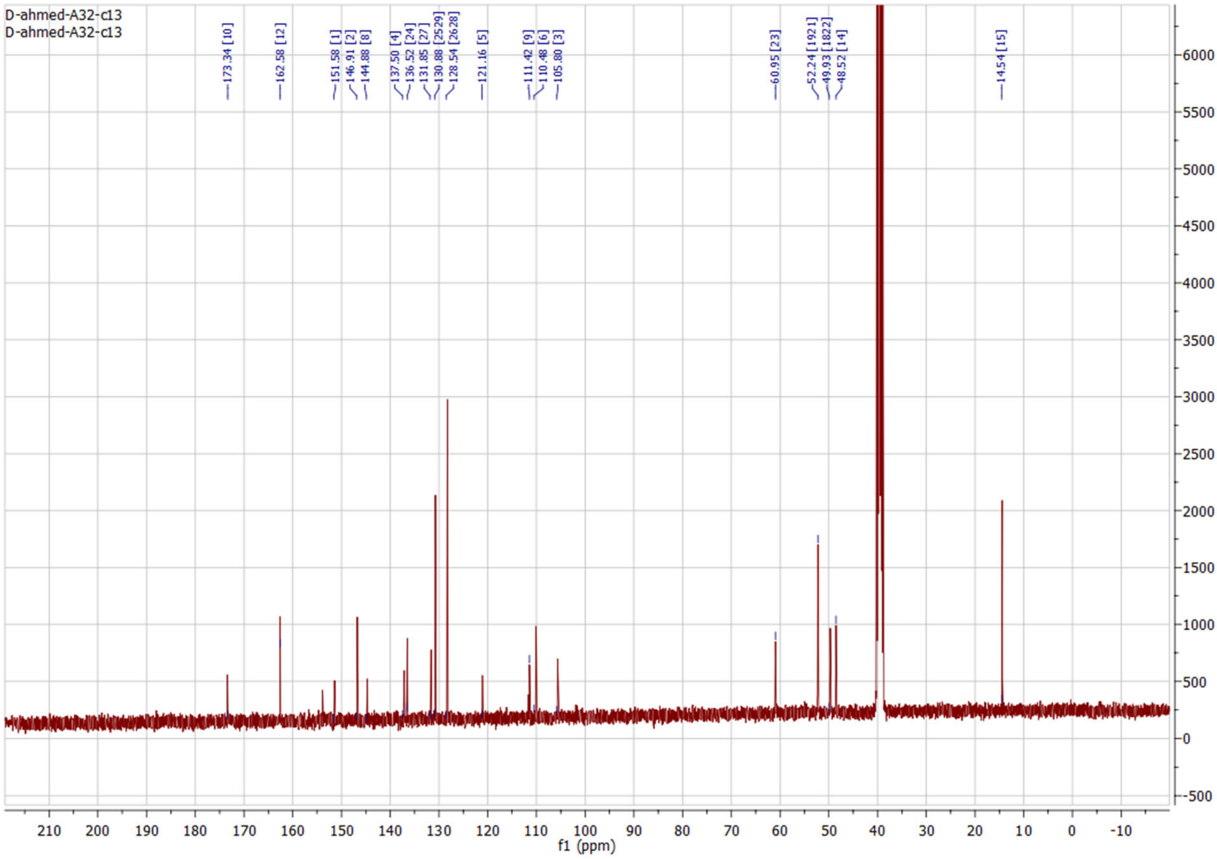

Figure S42: ^{13}C NMR of compound 11f.

12a

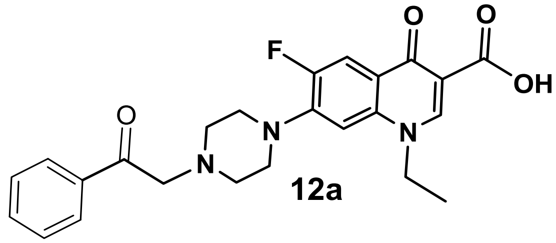

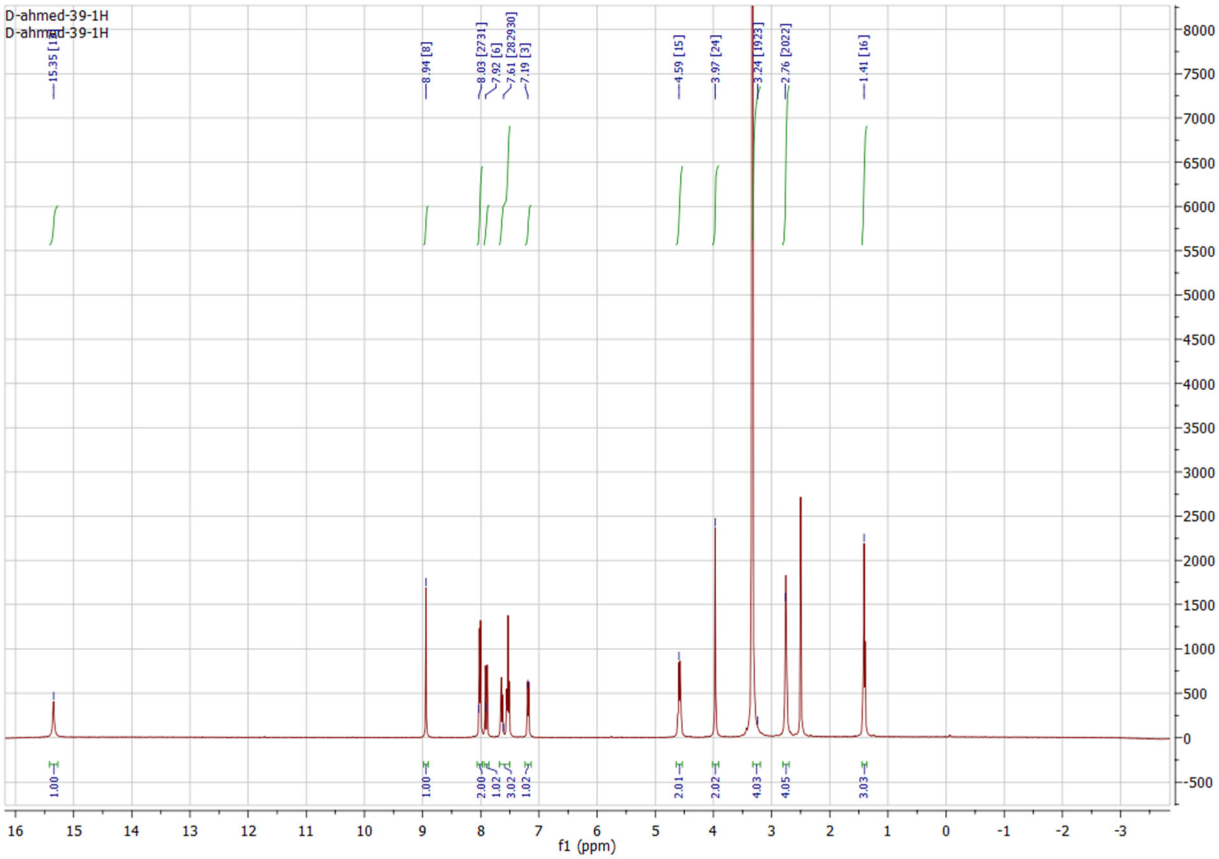

Figure S43: ^{1}H NMR of compound 12a.

12b

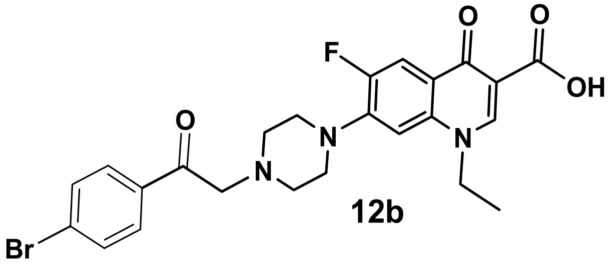

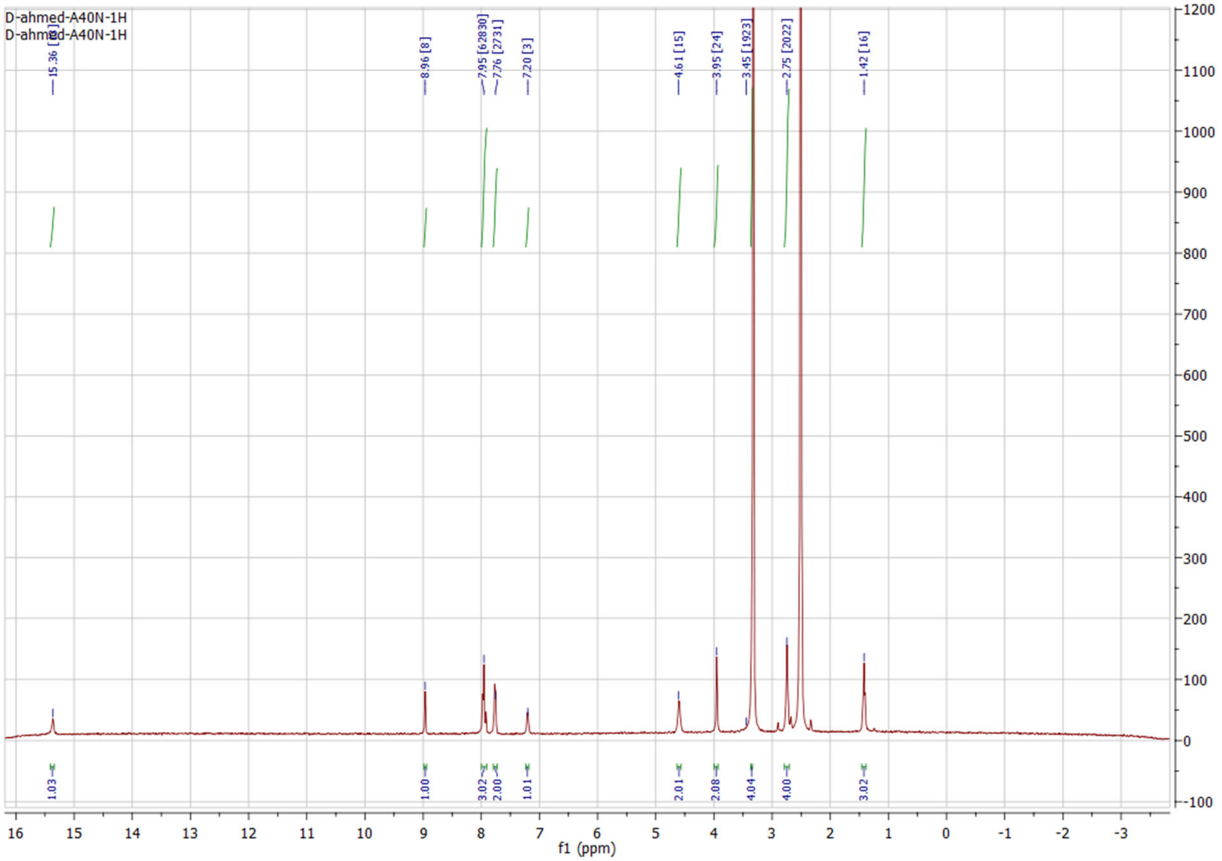

Figure S44: ^{1}H NMR of compound 12b.

12c

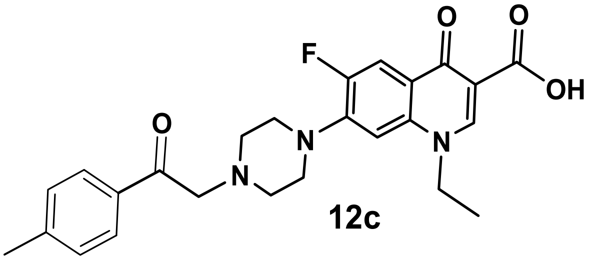

12c

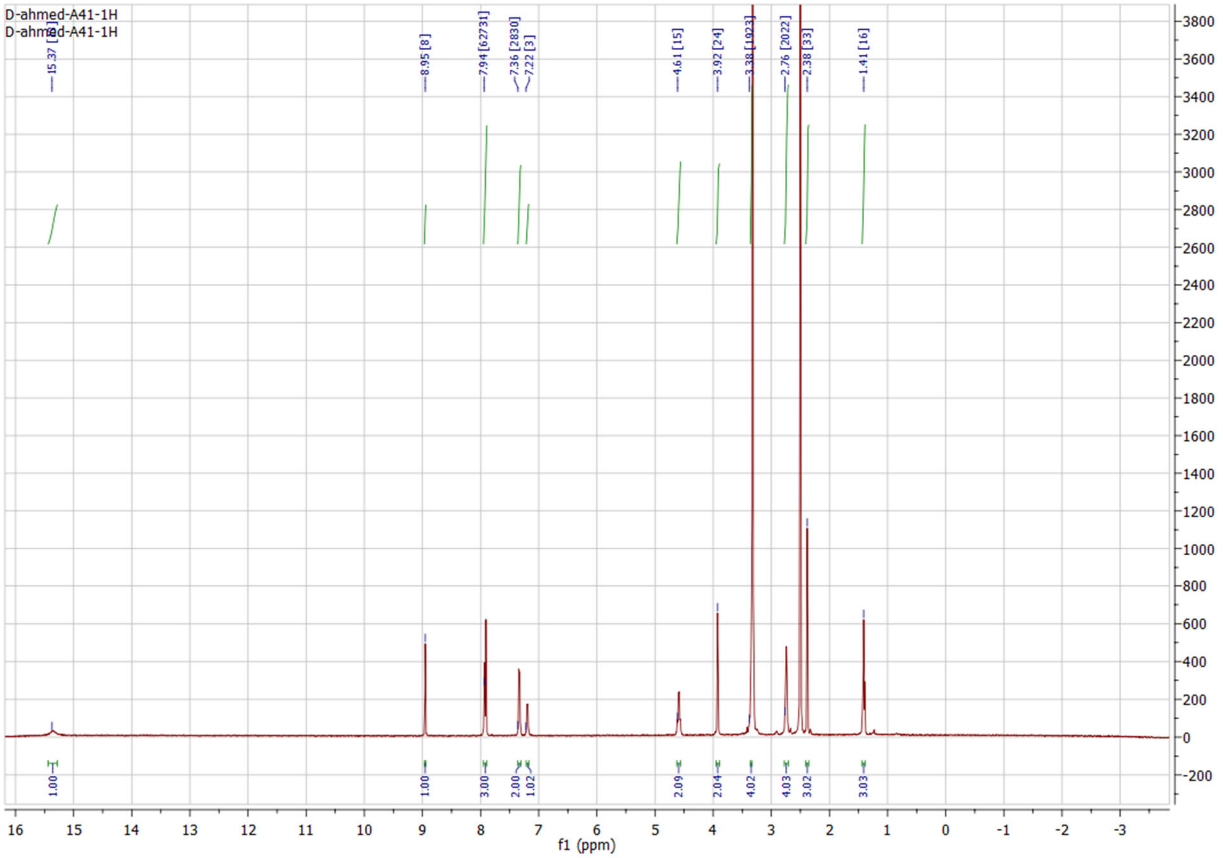

Figure S45: ^{1}H NMR of compound 12c.

12d

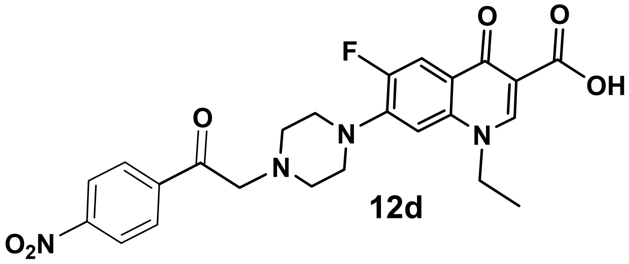

12d

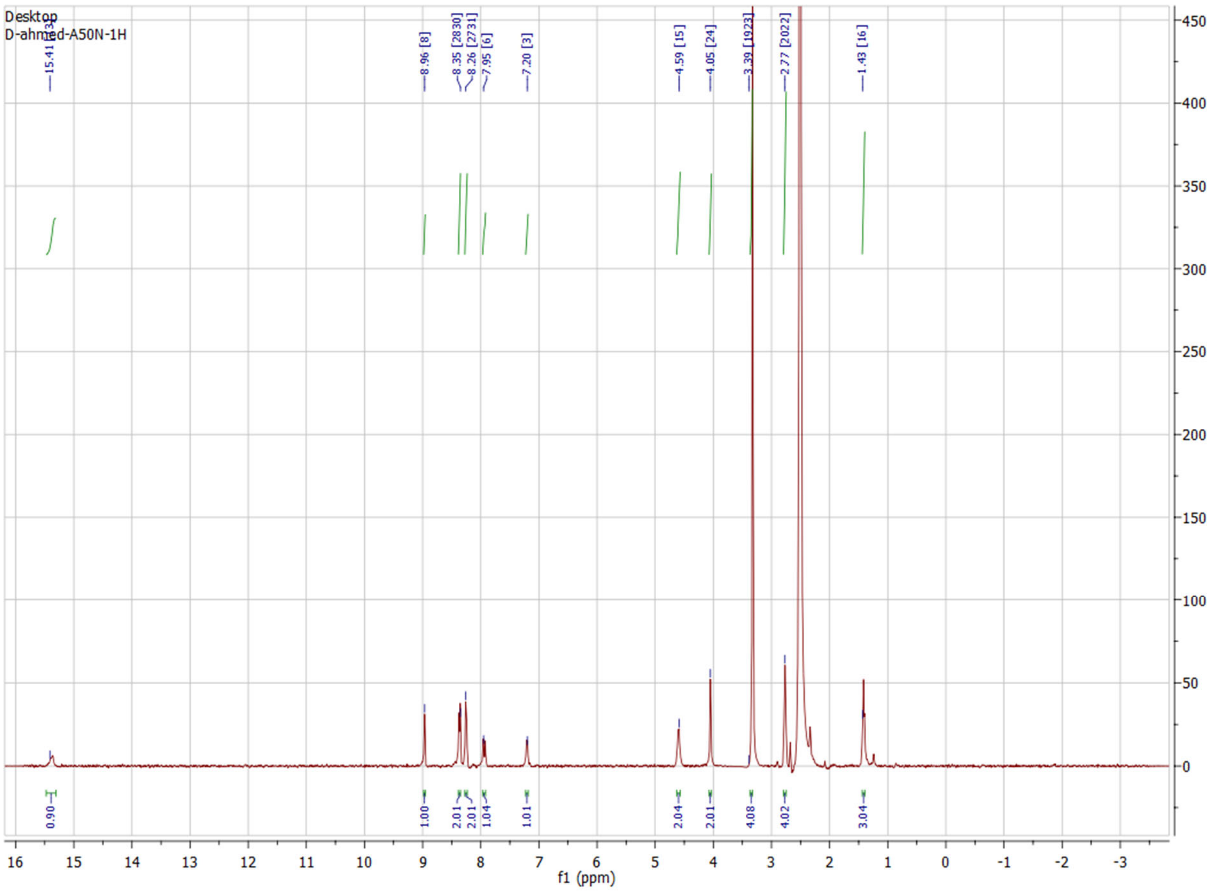

Figure S46: ^{1}H NMR of compound 12d.

13a

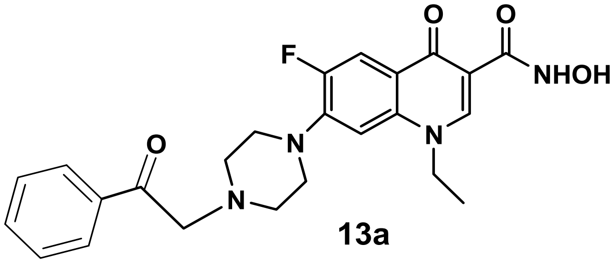

13a

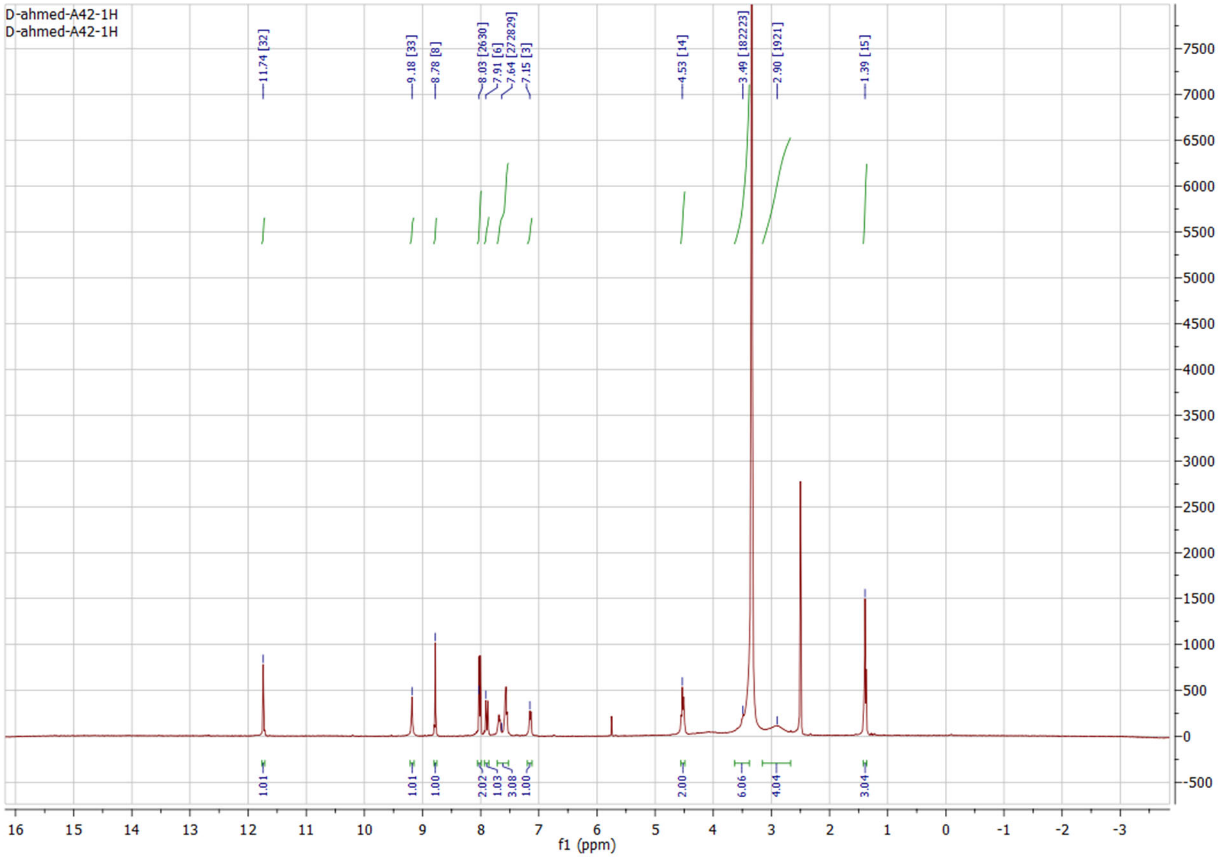

Figure S47: ^{1}H NMR of compound 13a.

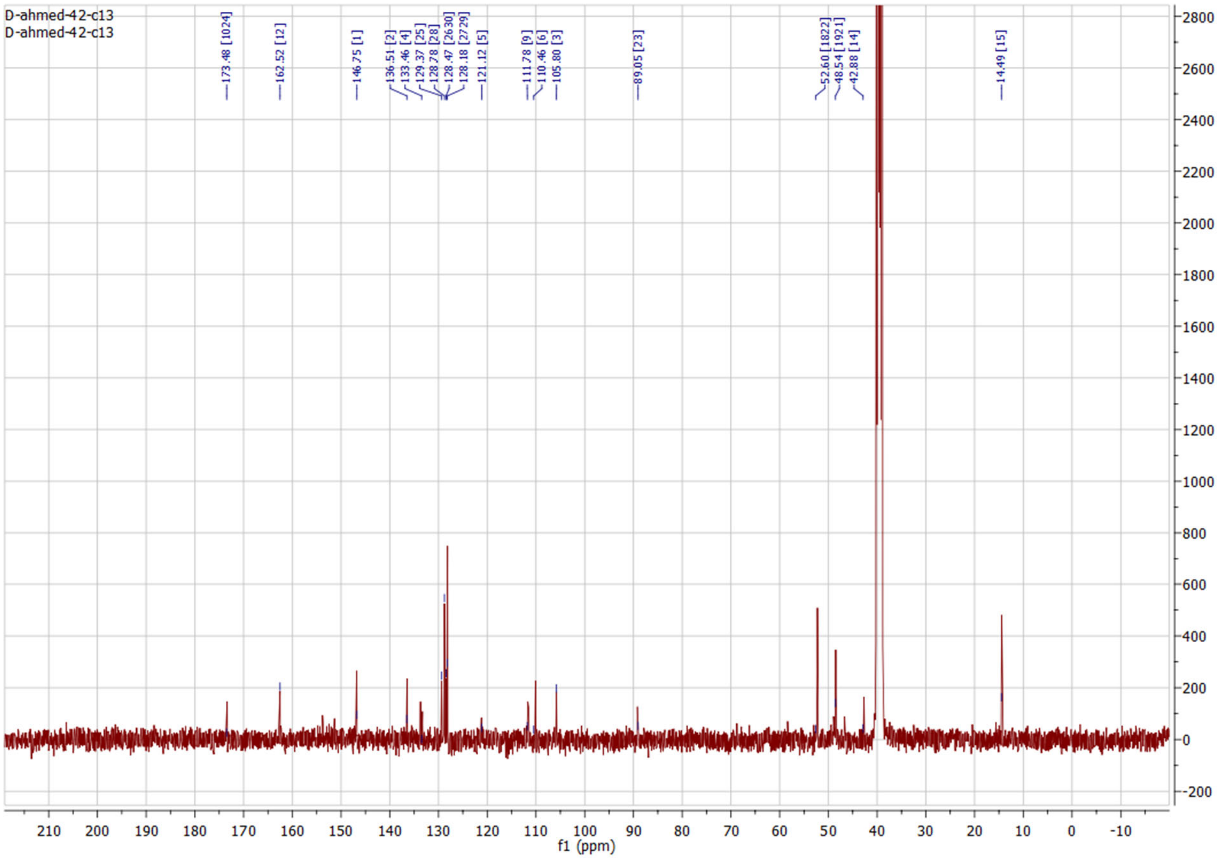

Figure S48: ^{13}C NMR of compound 13a.

13b

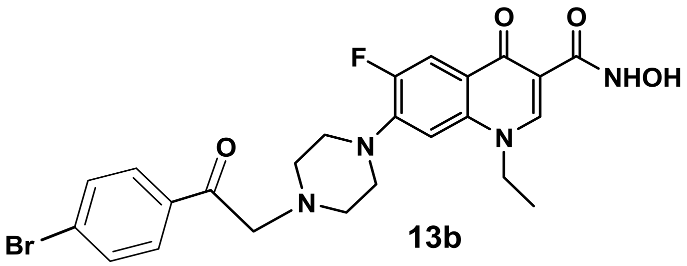

13b

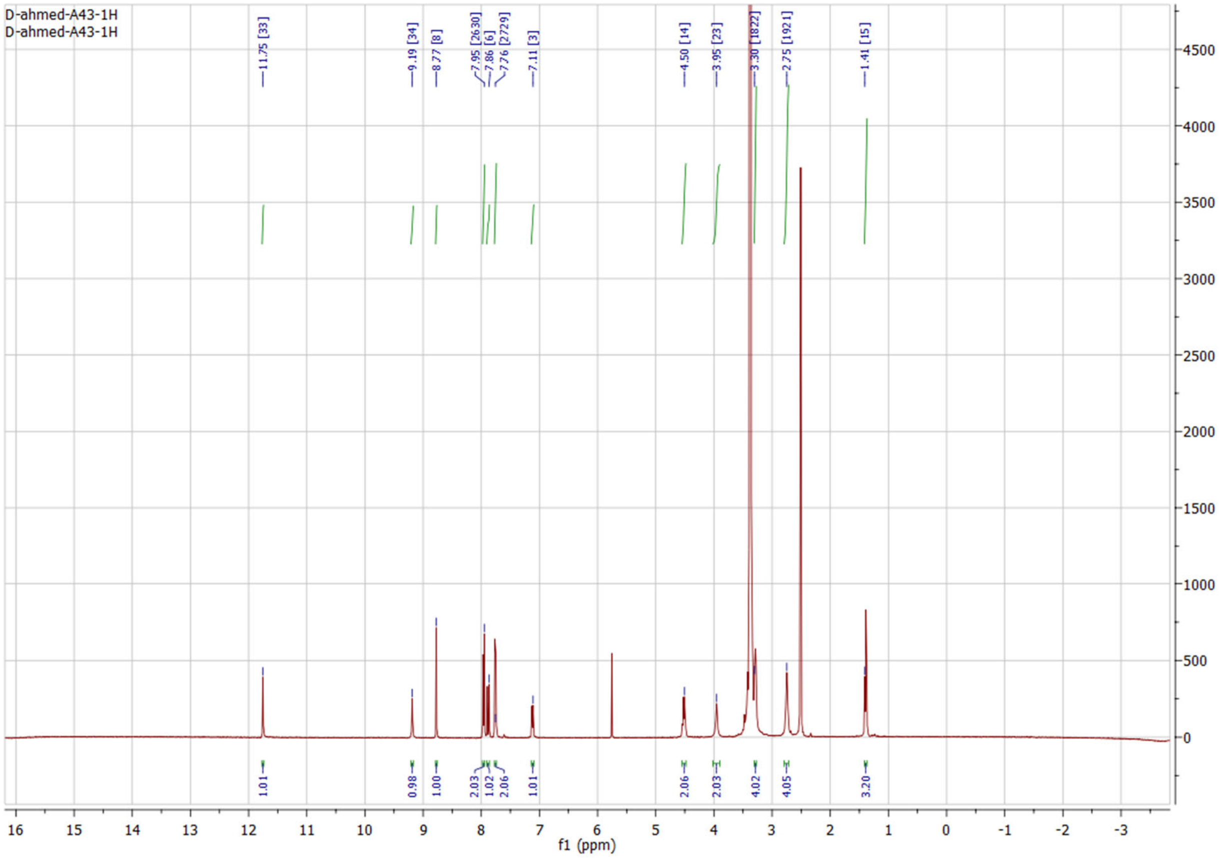

Figure S49: ^{1}H NMR of compound 13b.

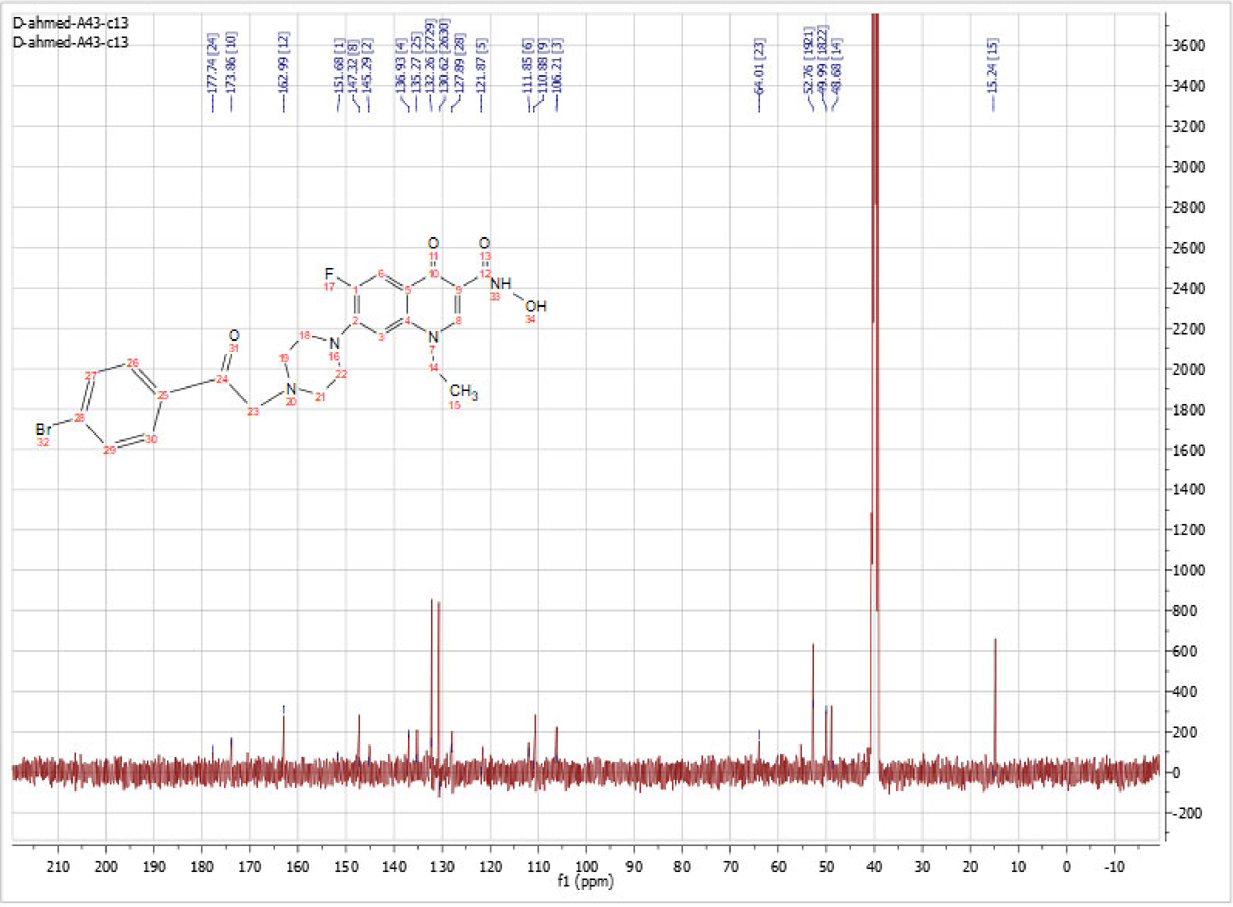

Figure S50: ^{13}C NMR of compound 13b.

13c

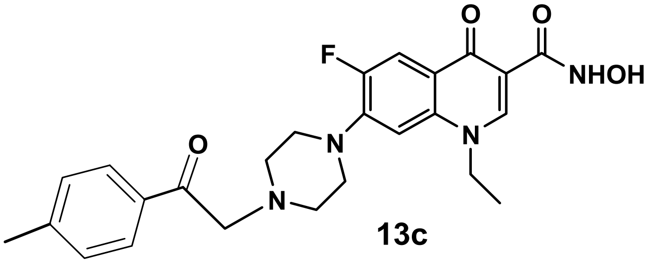

13c

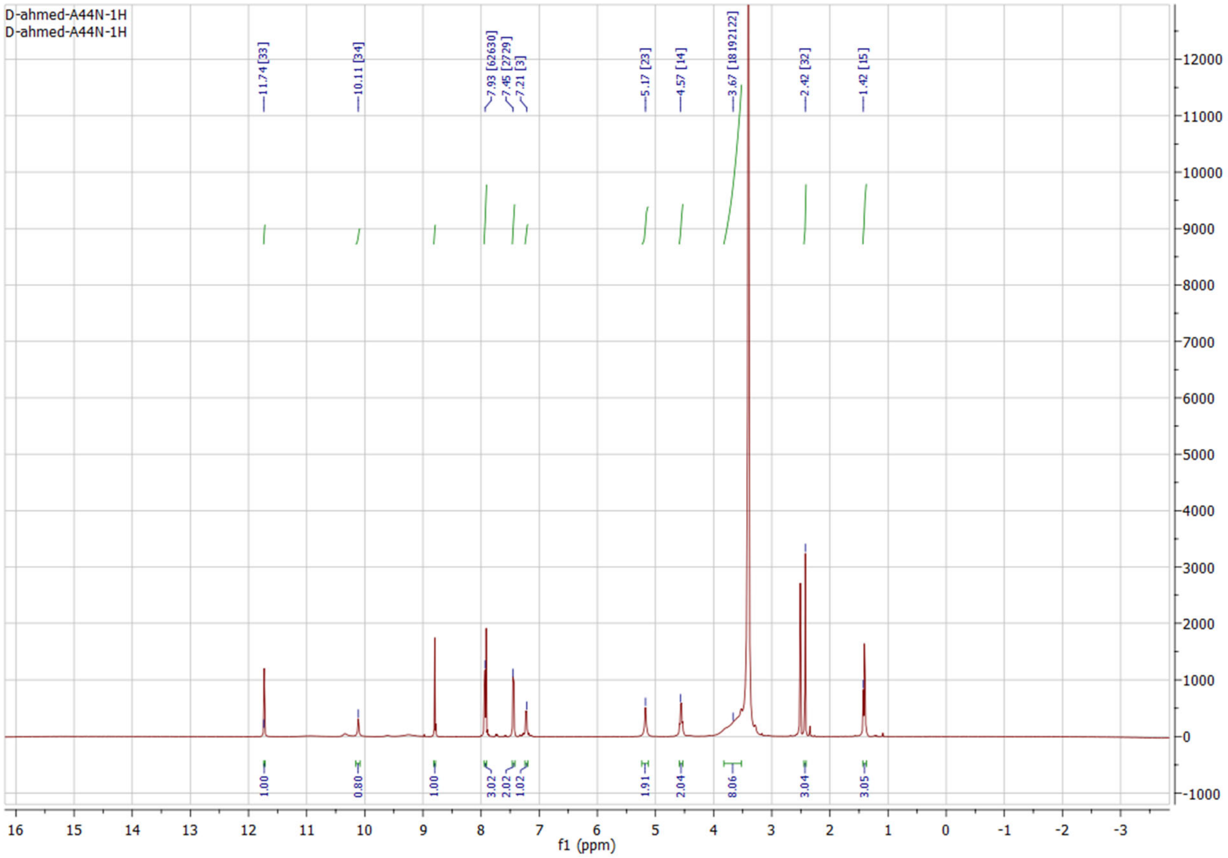

Figure S51: ^{1}H NMR of compound 13c.

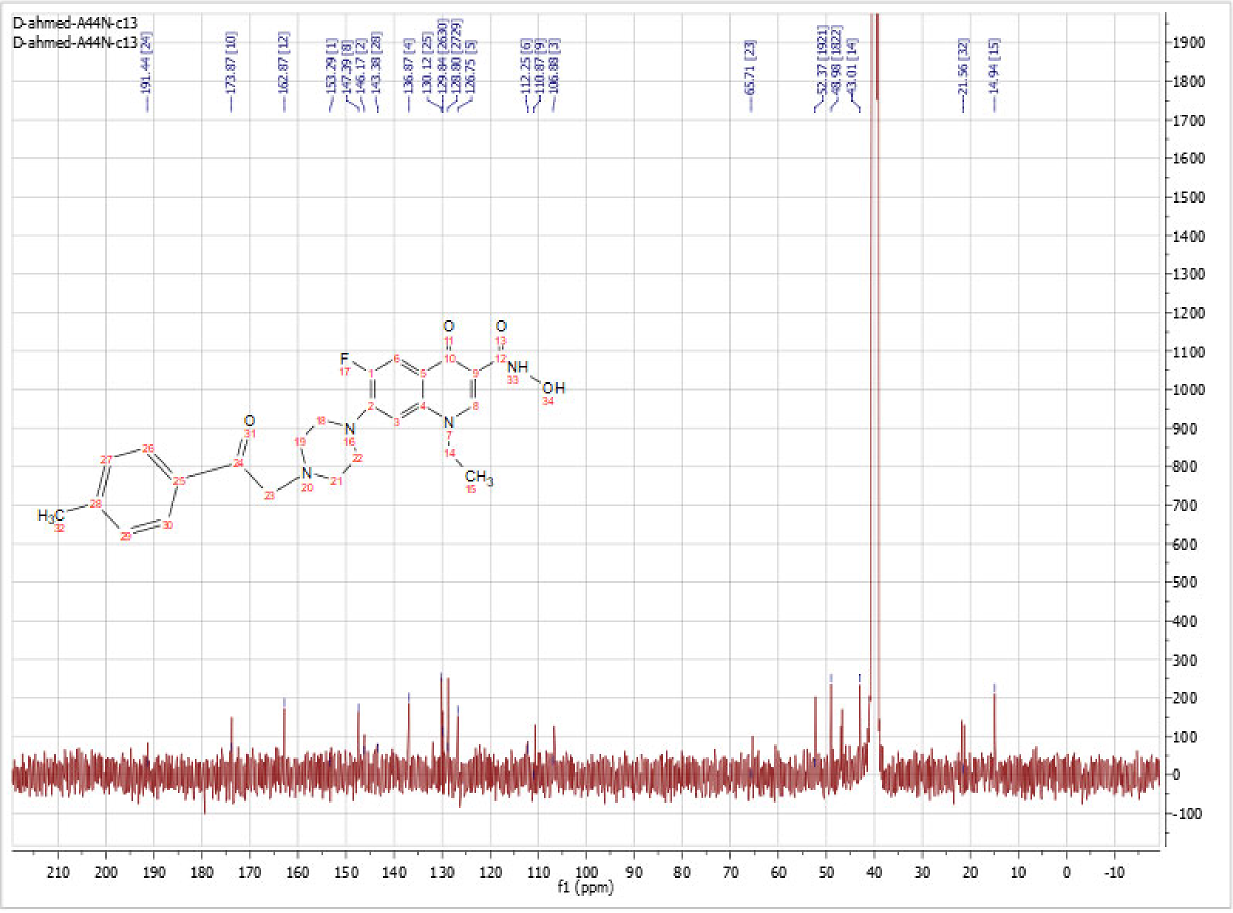

Figure S52: ^{13}C NMR of compound 13c.

13d

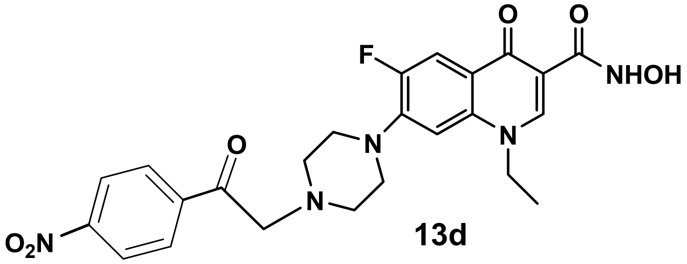

13d

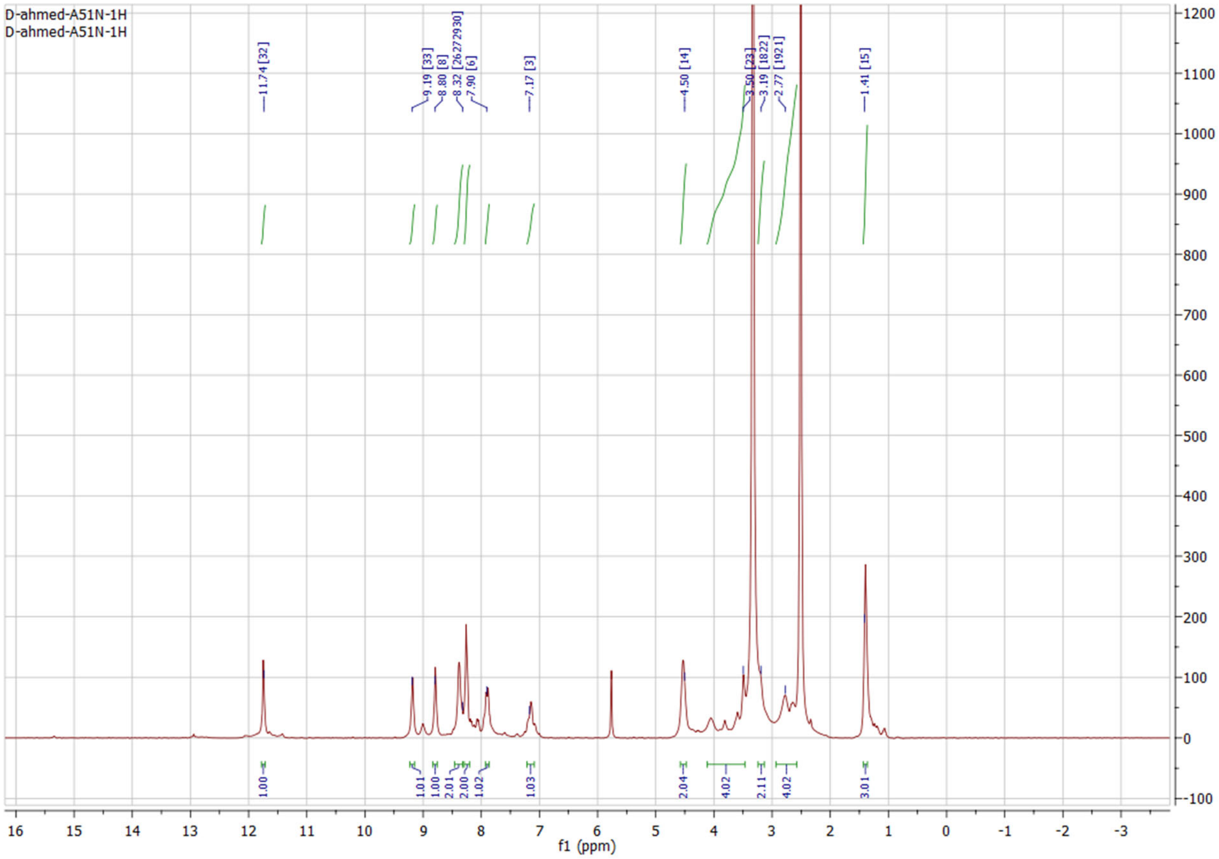

Figure S53: ^{1}H NMR of compound 13d.

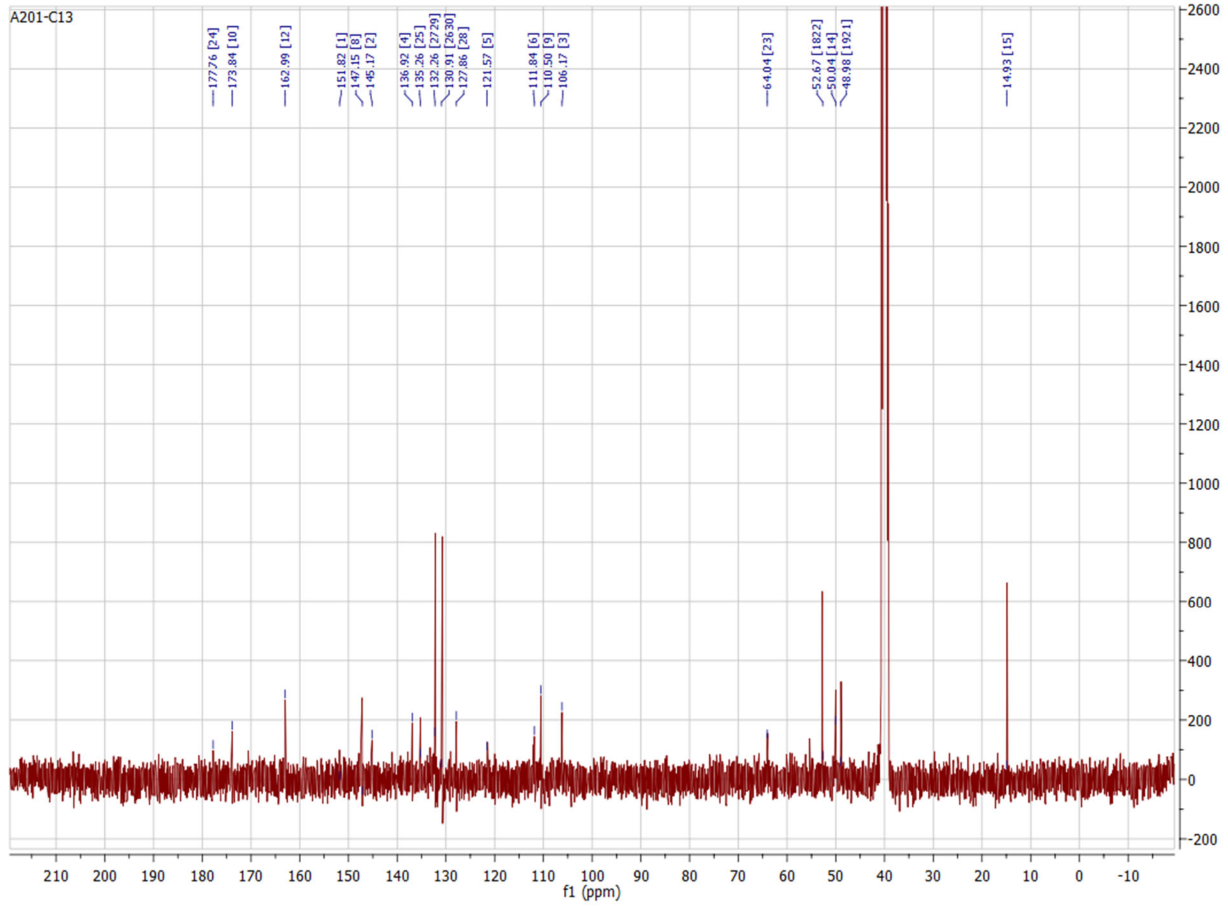

Figure S54: ^{13}C NMR of compound 13d.

16a

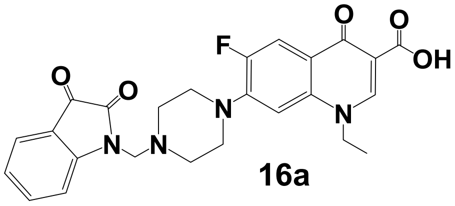

16a

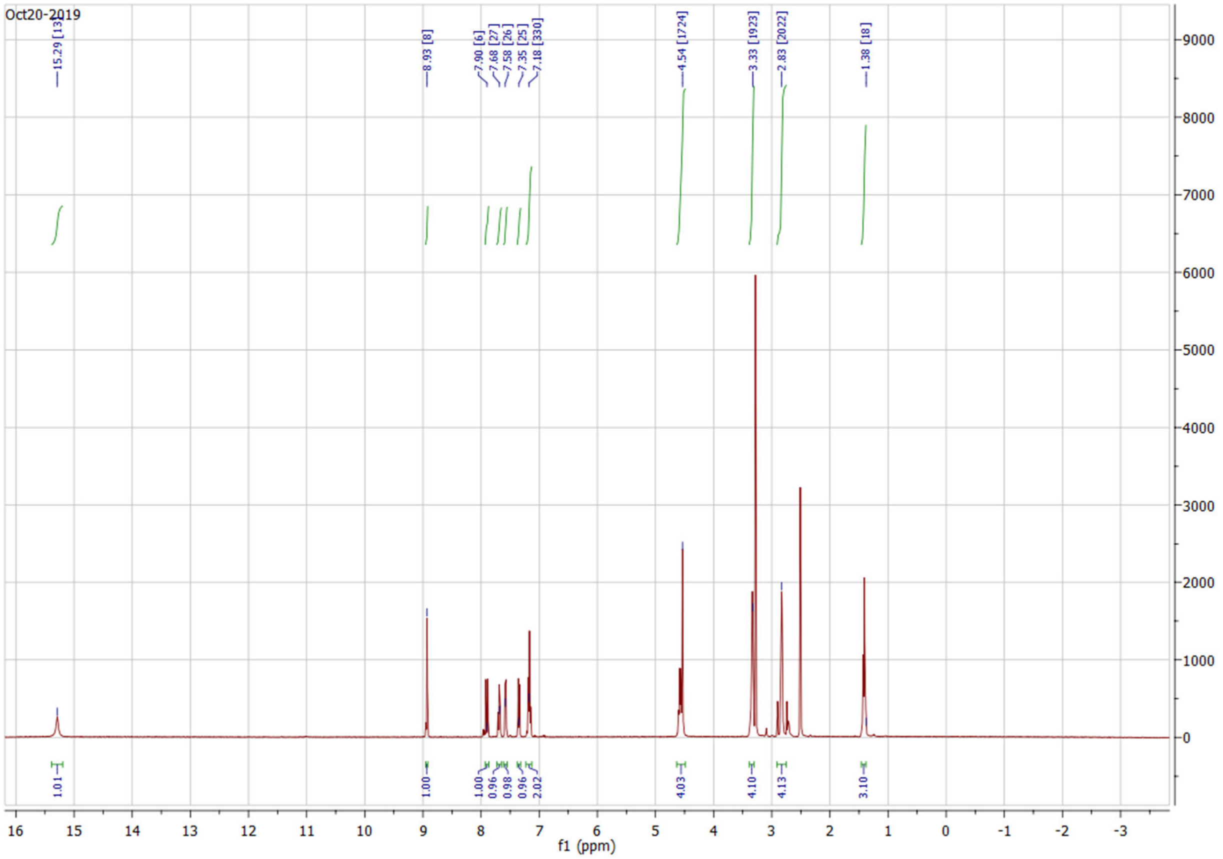

Figure S55: ^{1}H NMR of compound 16a.

16b

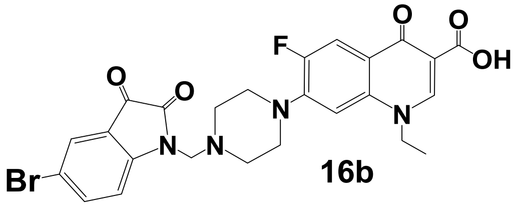

16b

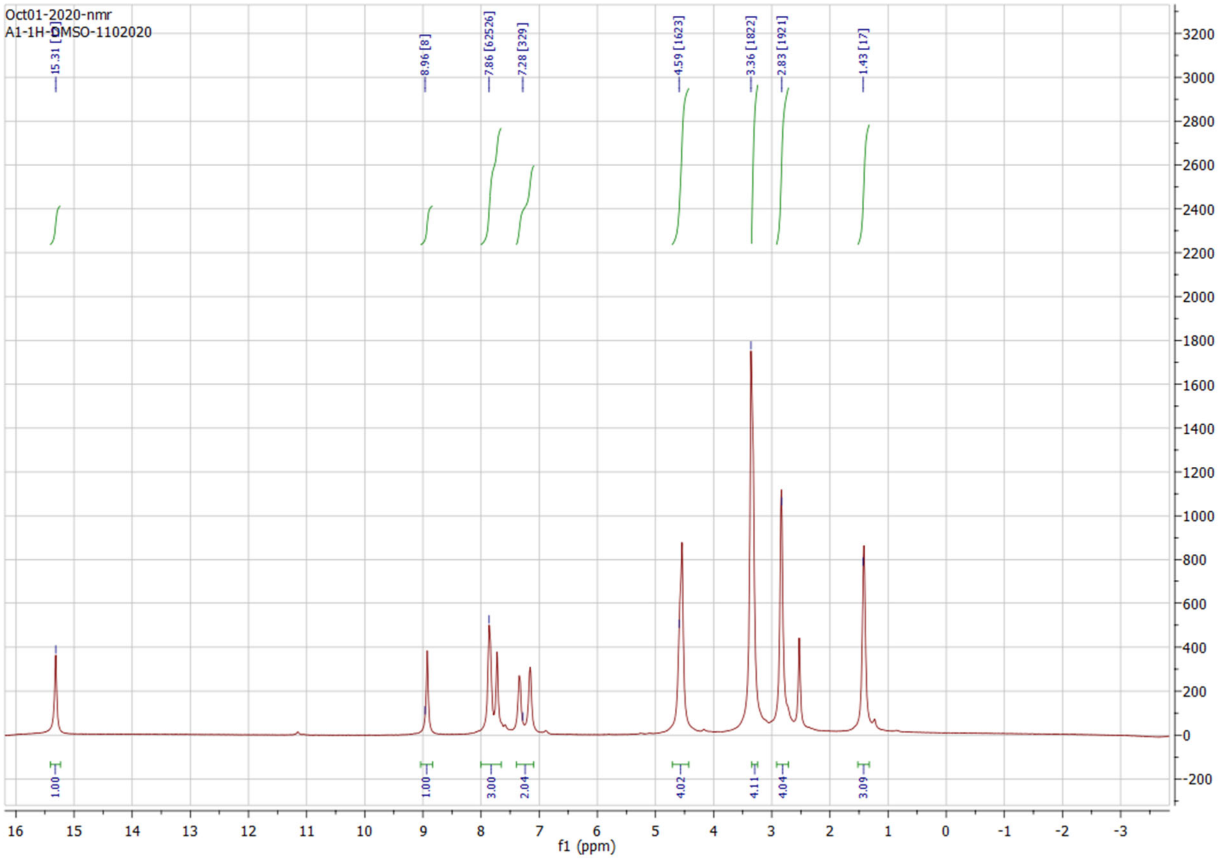

Figure S56: ^{1}H NMR of compound 16b.

16c

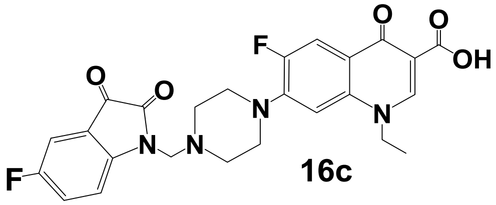

16c

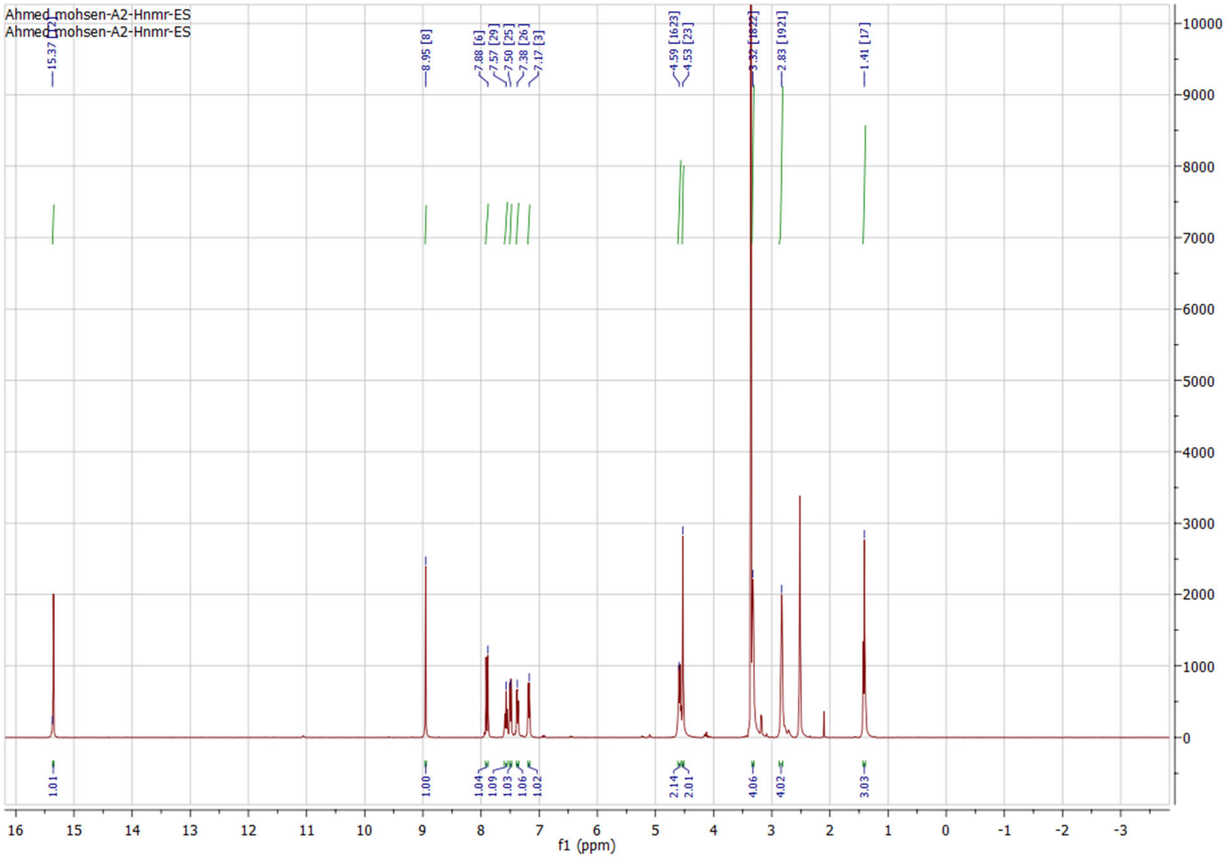

Figure S57: ^{1}H NMR of compound 16c.

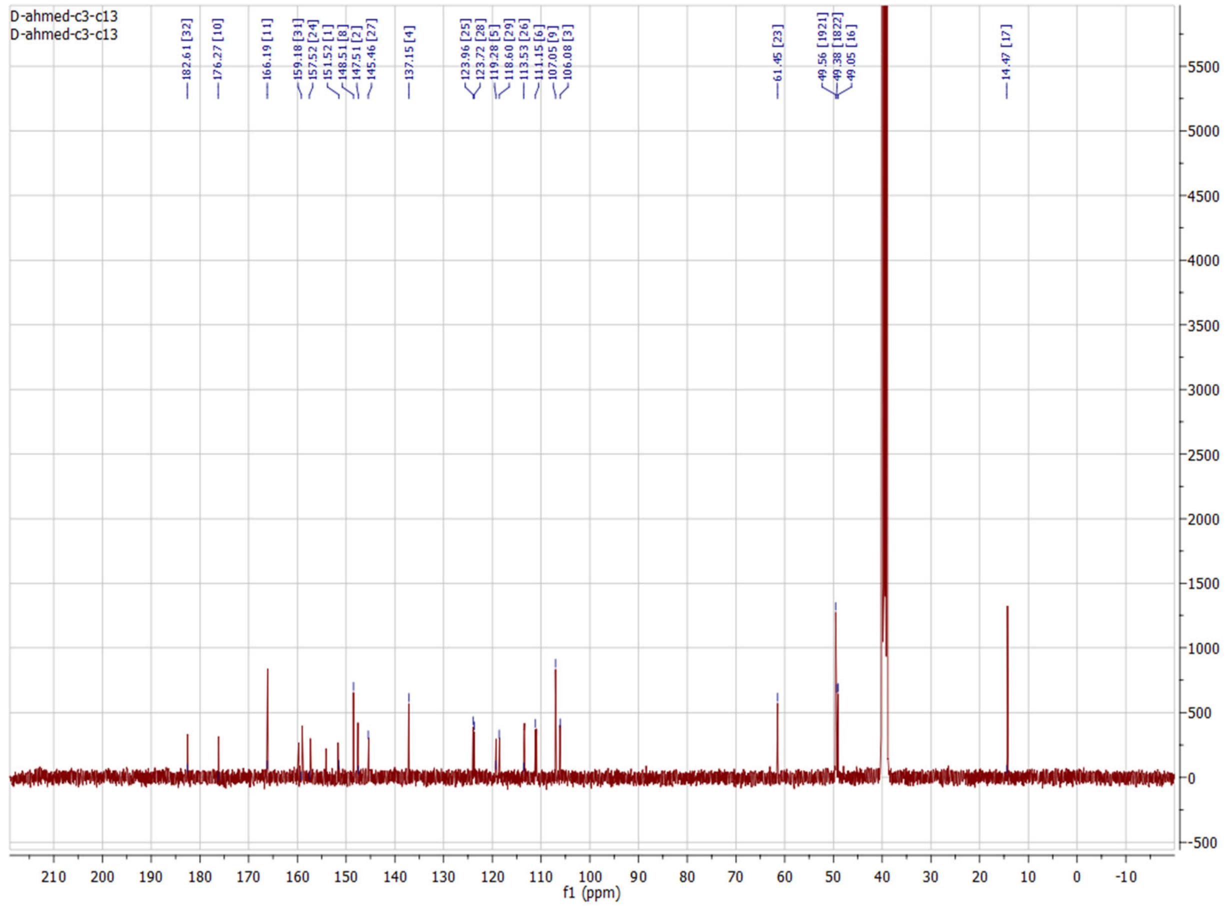

Figure S58: ^{13}C NMR of compound 16c.

16d

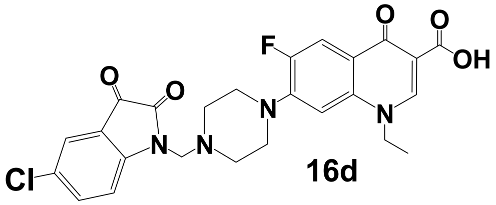

16d

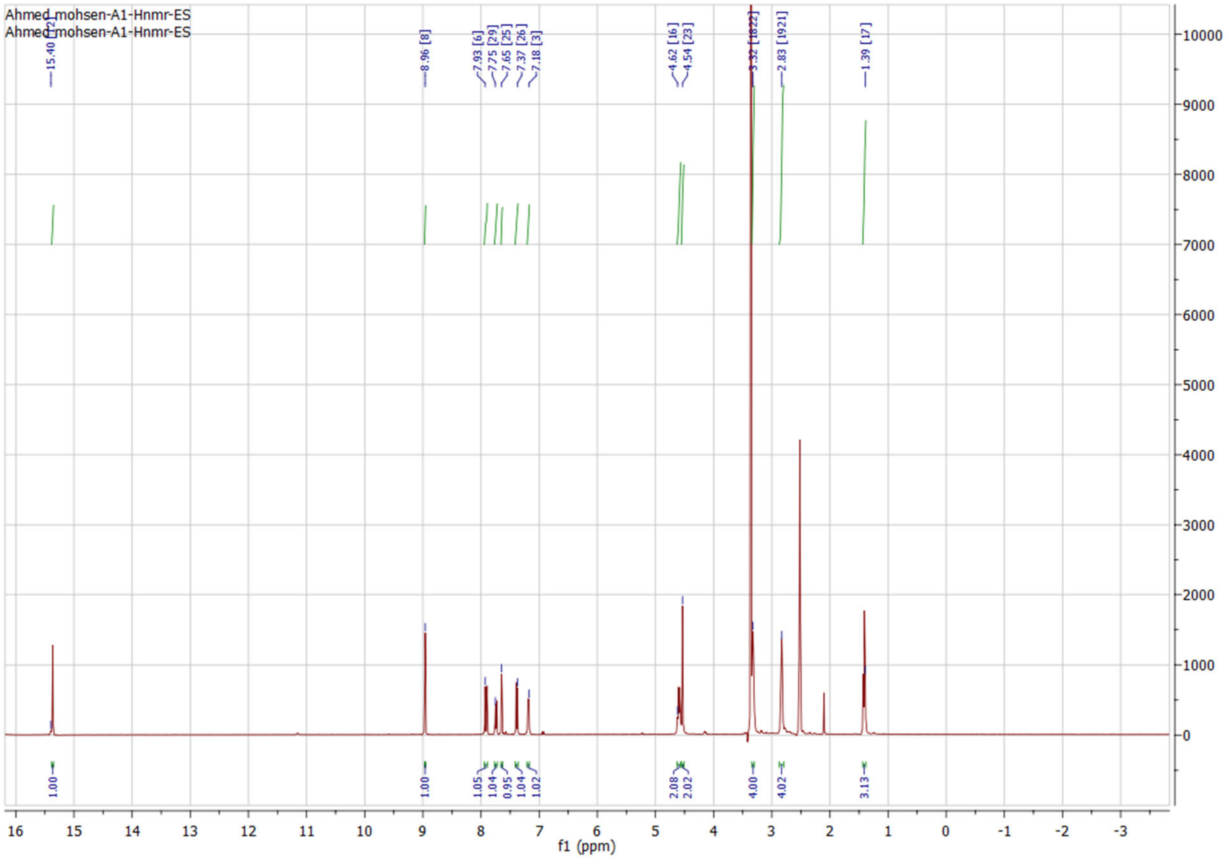

Figure S59: ^{1}H NMR of compound 16d.

16e

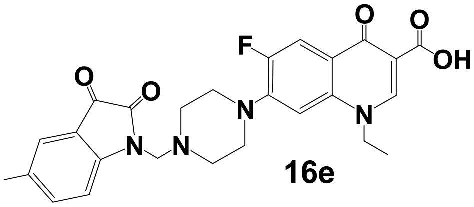
16e

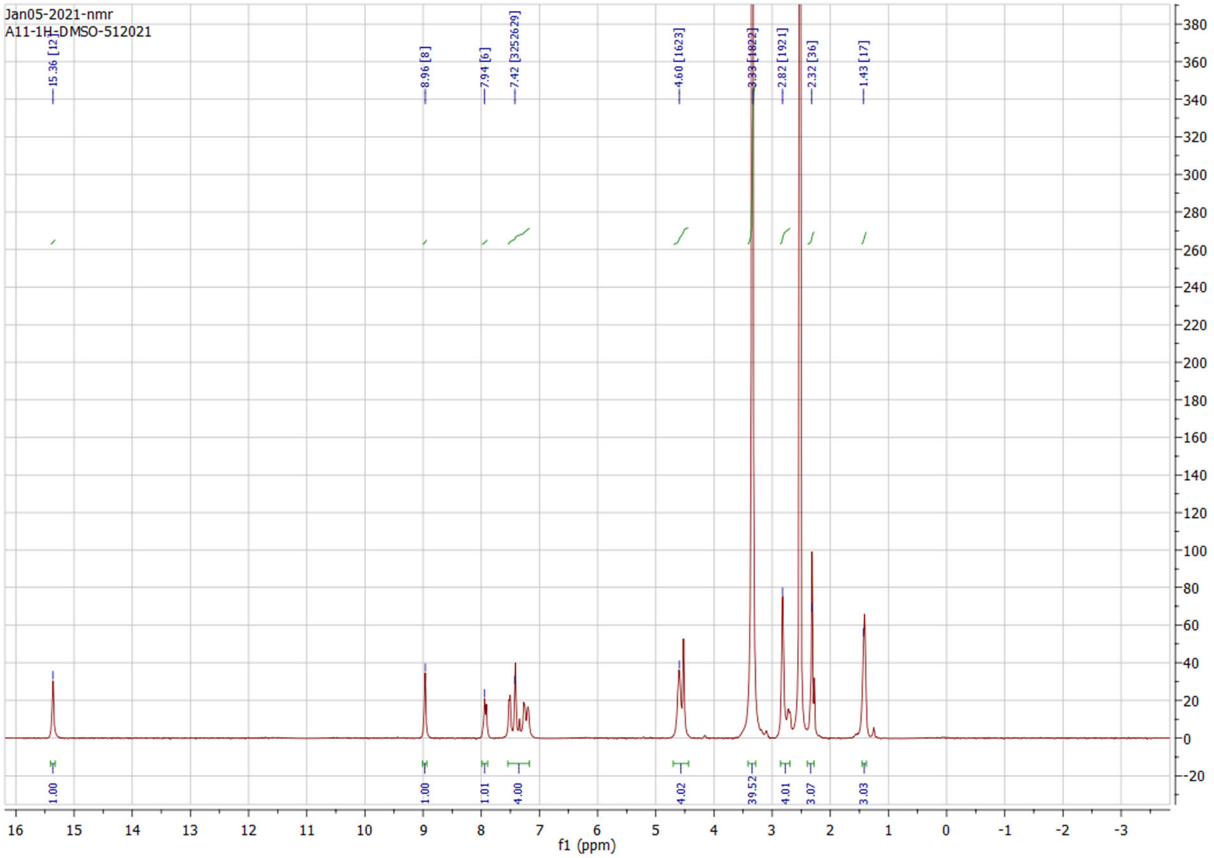

Figure S60: ^{1}H NMR of compound 16e.

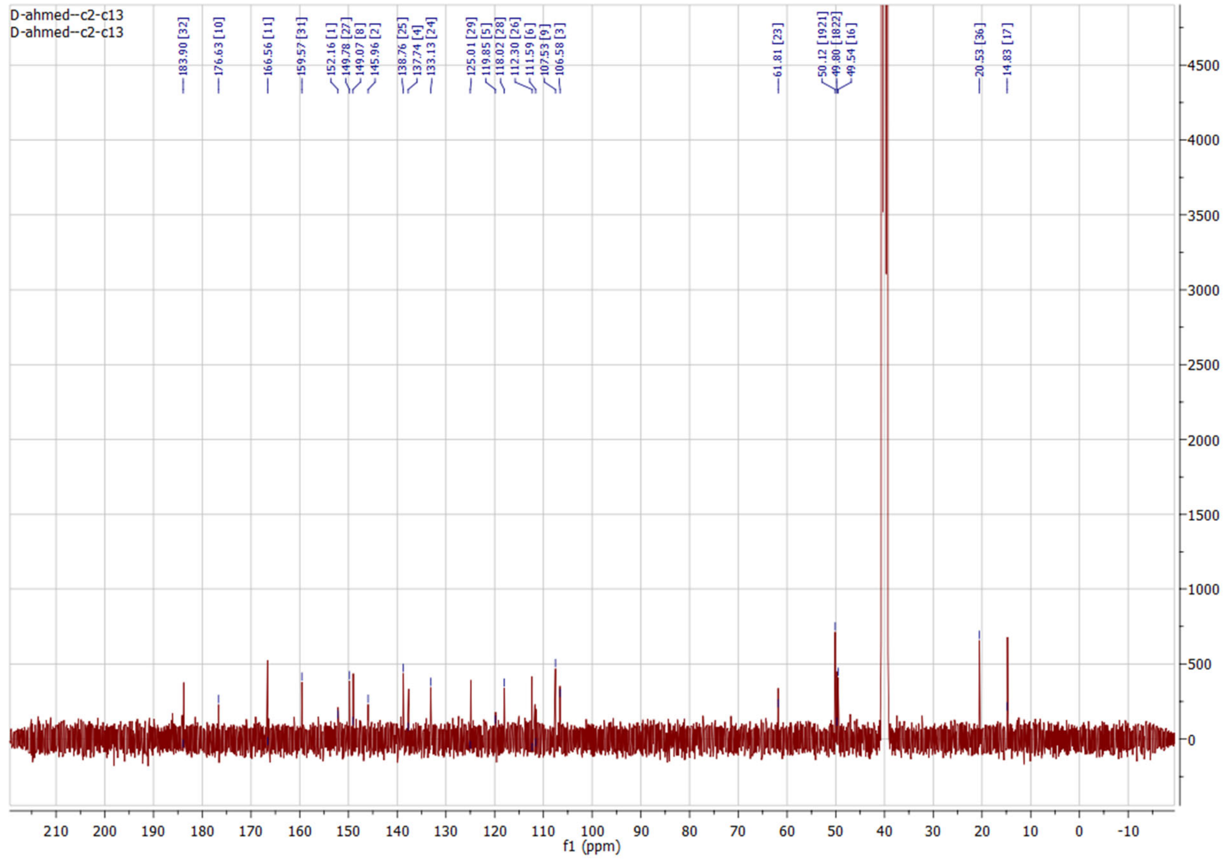

Figure S61: ^{13}C NMR of compound 16e.

16f

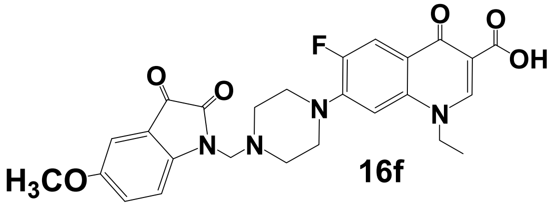

16f

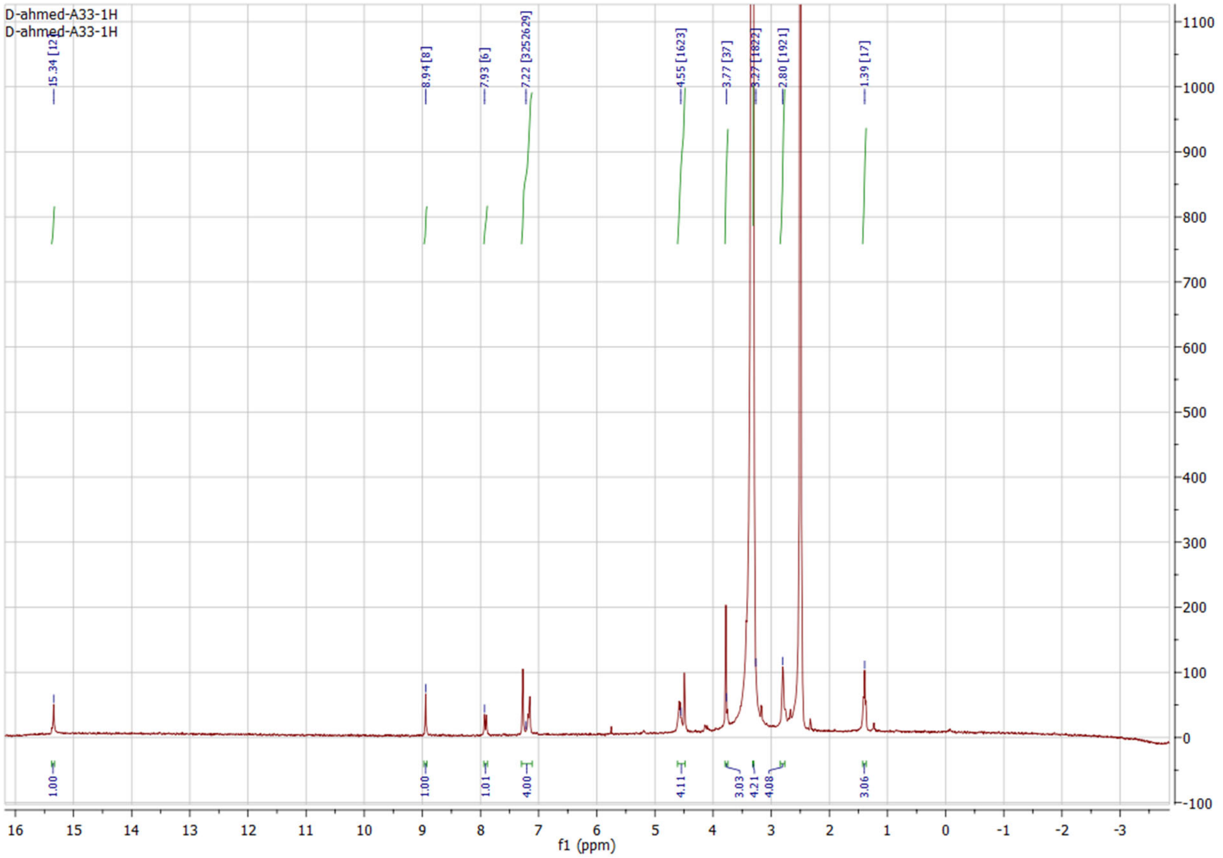

Figure S62: ^{1}H NMR of compound 16f.

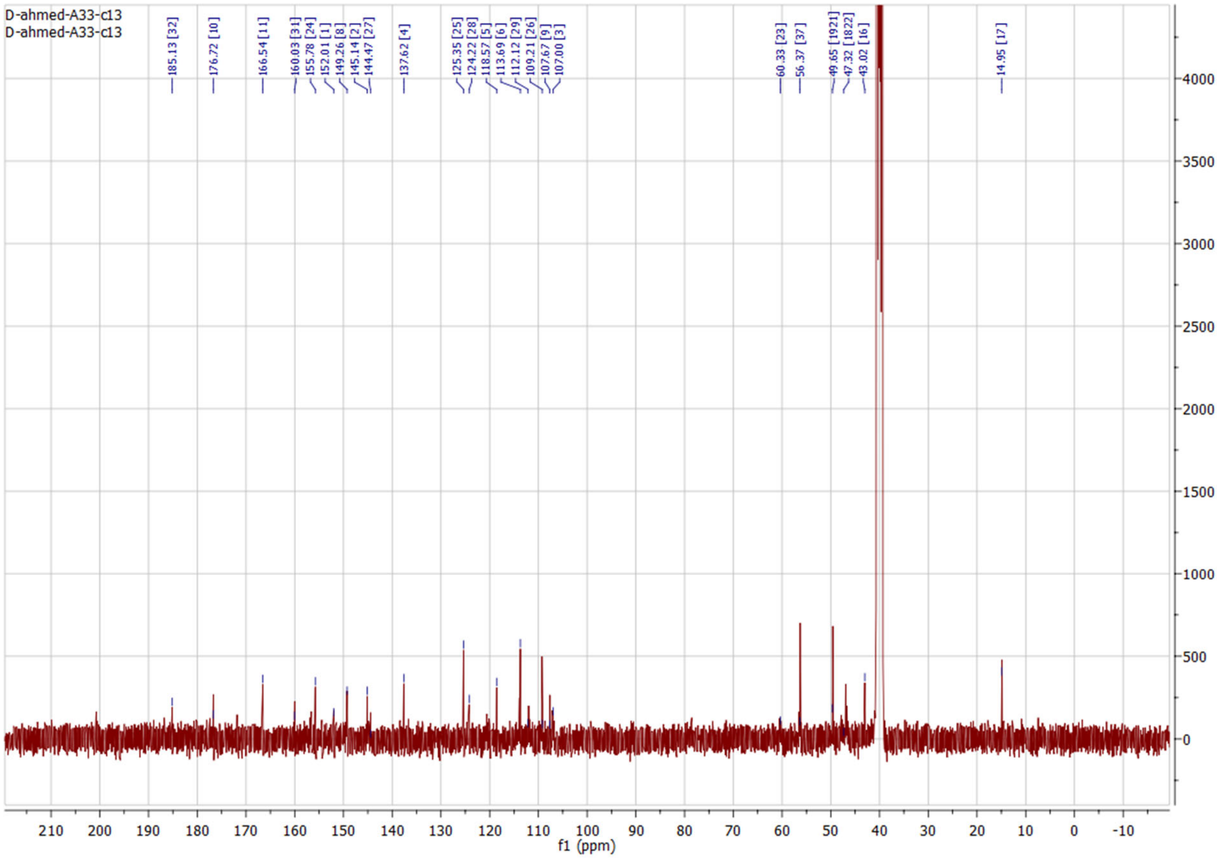

Figure S63: ^{13}C NMR of compound 16f.

17a

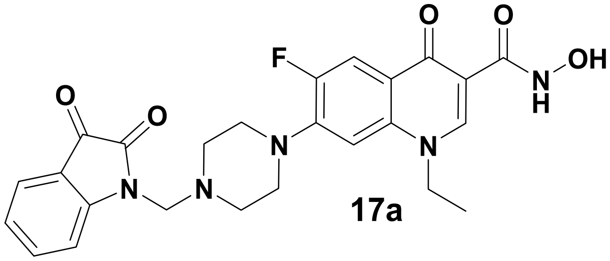
17a

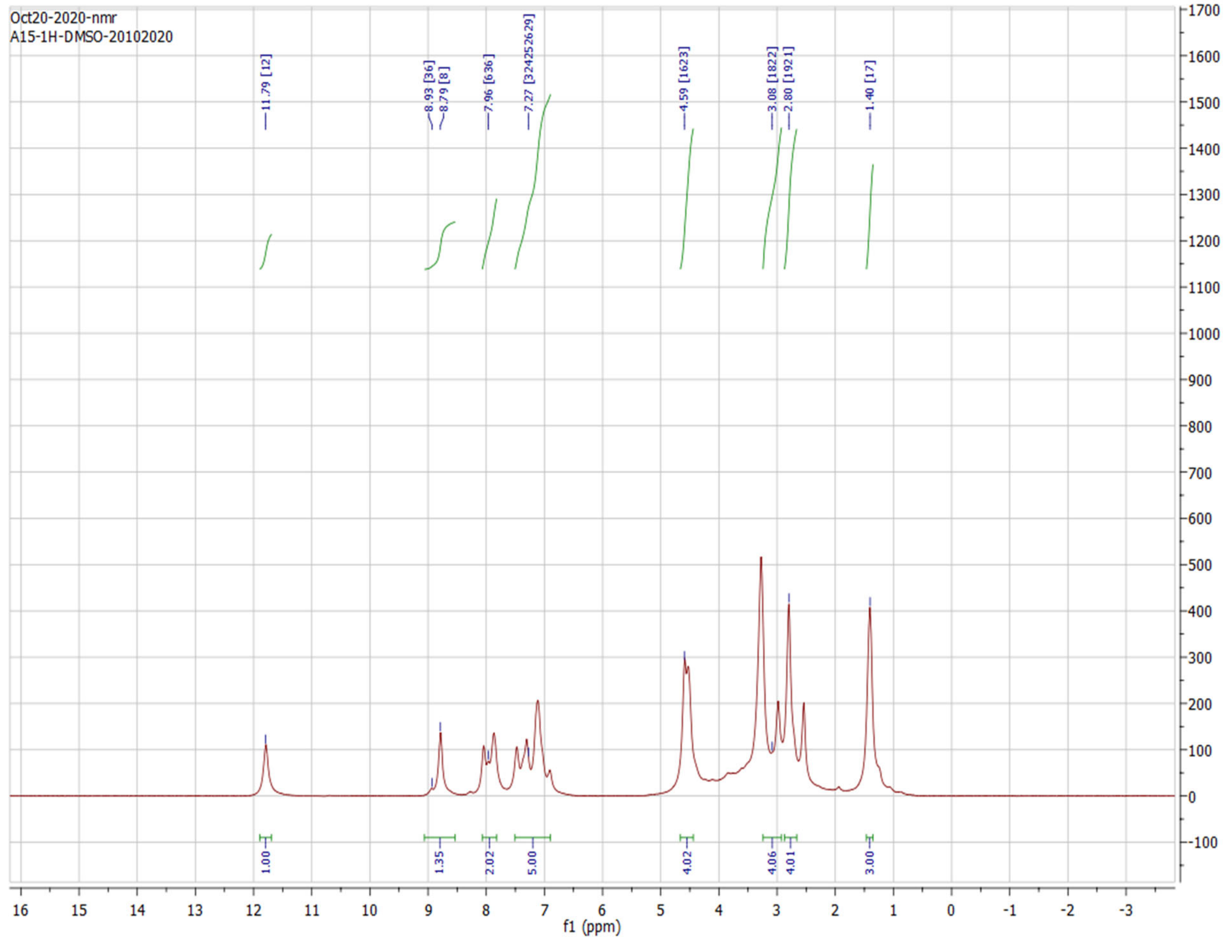

Figure S64: ^{1}H NMR of compound 17a.

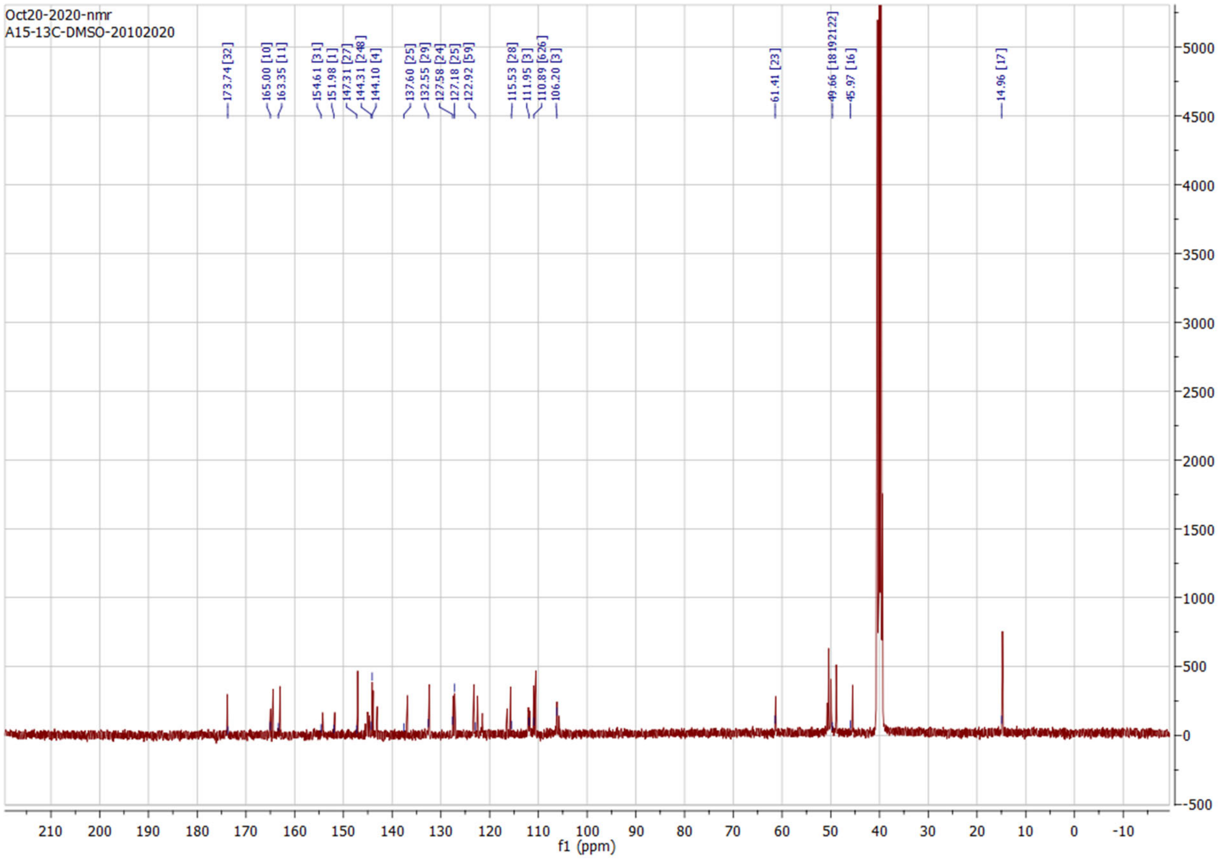

Figure S65: ^{13}C NMR of compound 17a.

17b

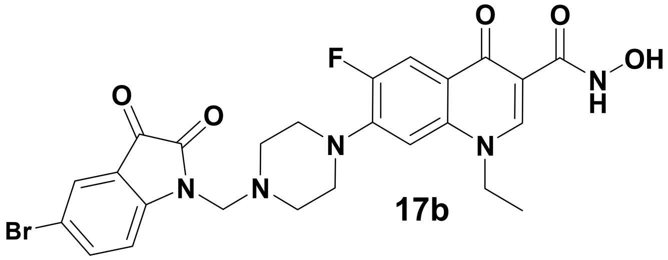

17b

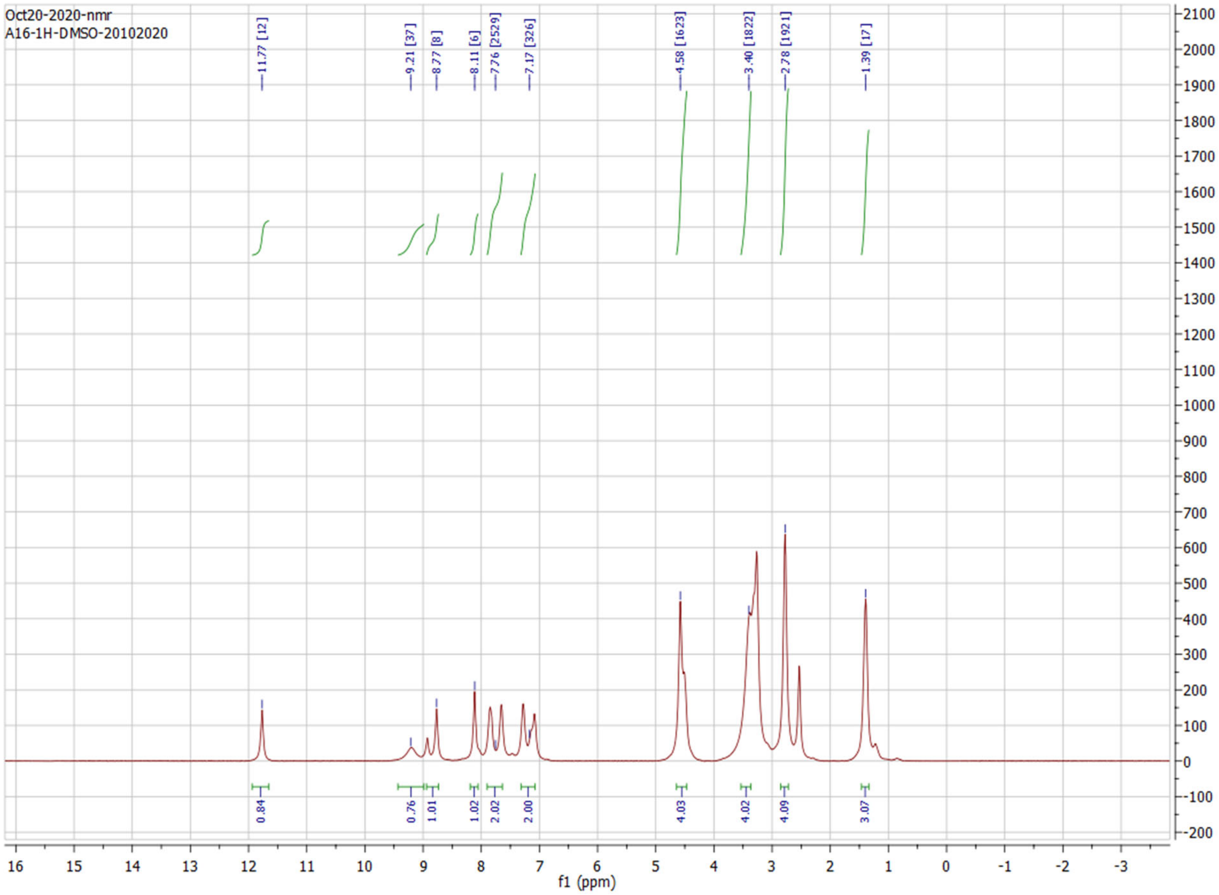

Figure S66: ^{1}H NMR of compound 17b.

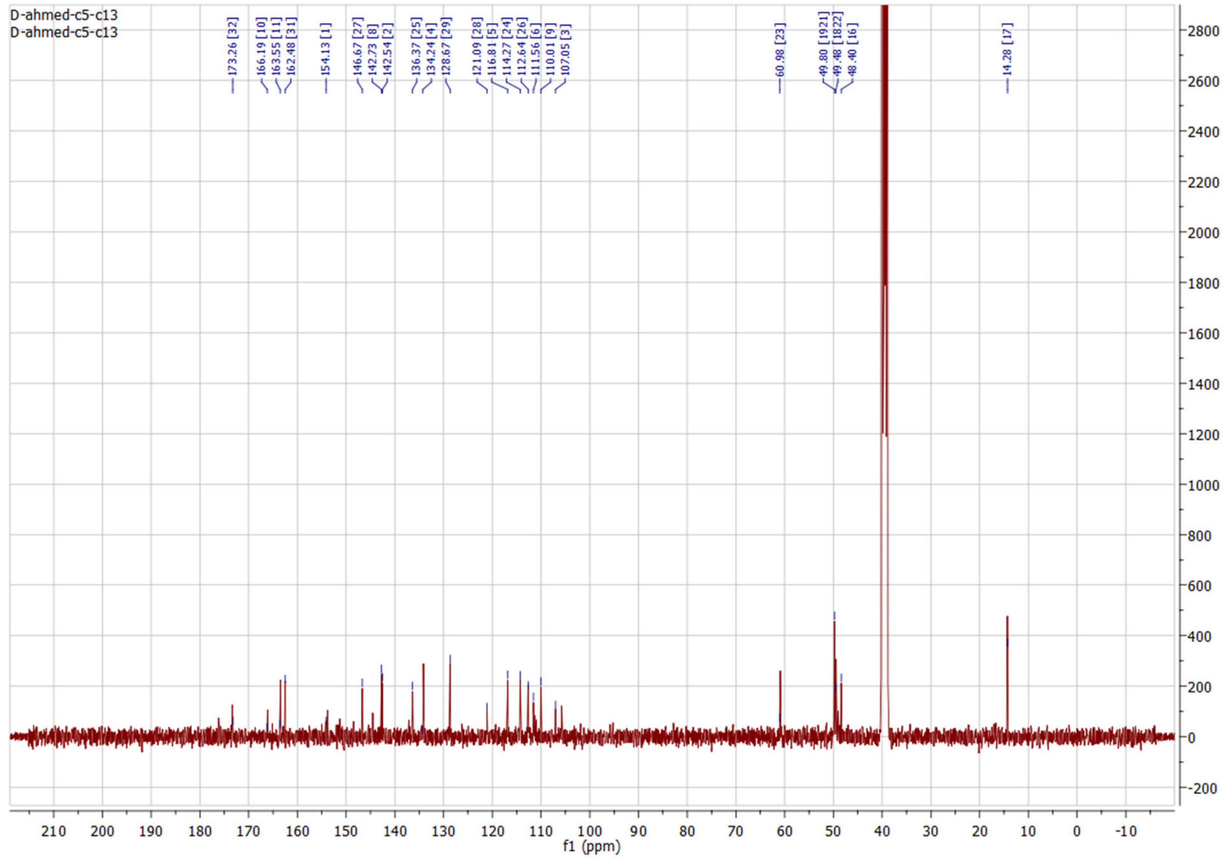

Figure S67: ^{13}C NMR of compound 17b.

17c

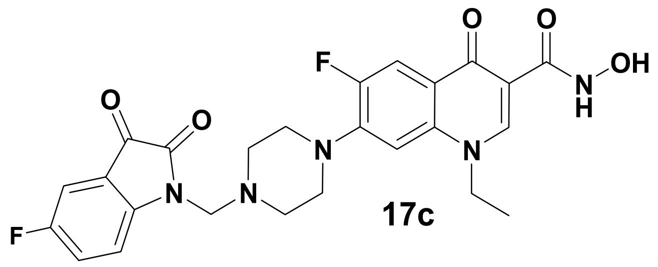

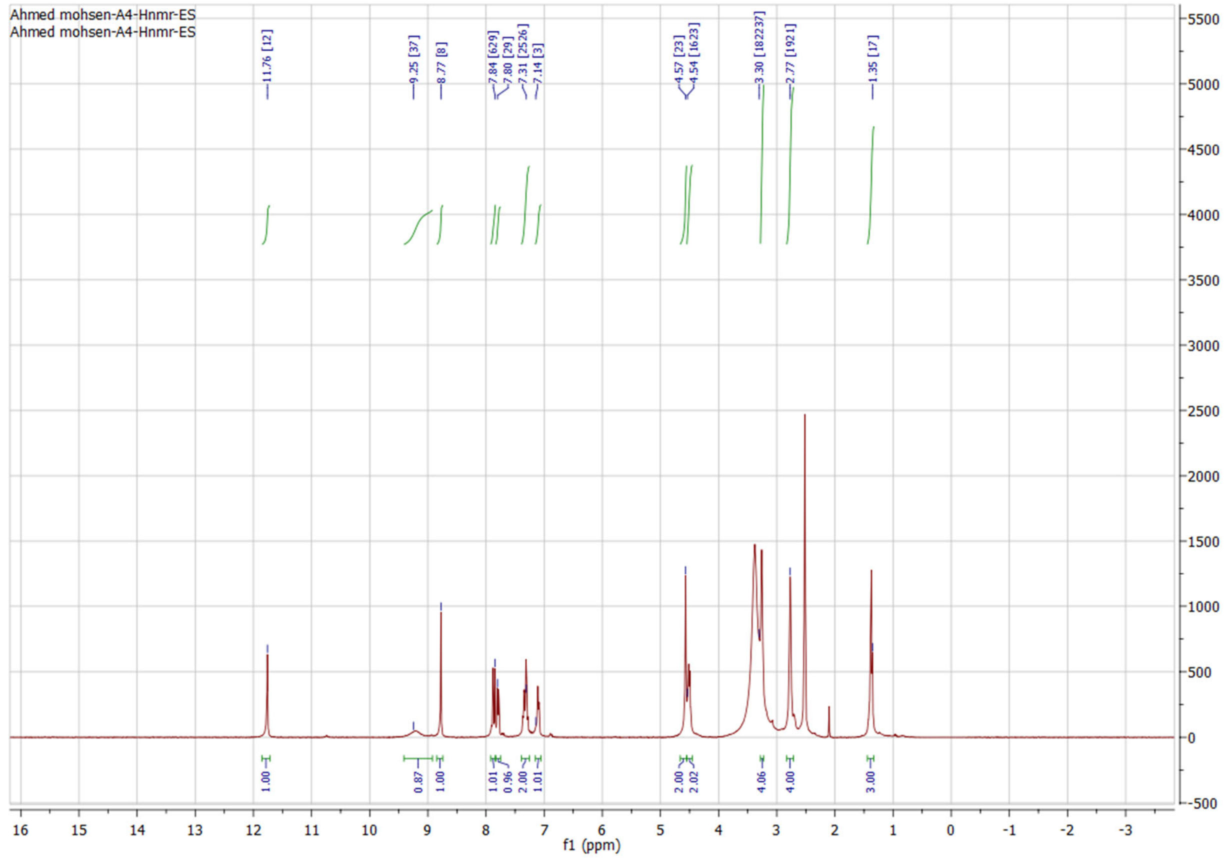

Figure S68: ^{1}H NMR of compound 17c.

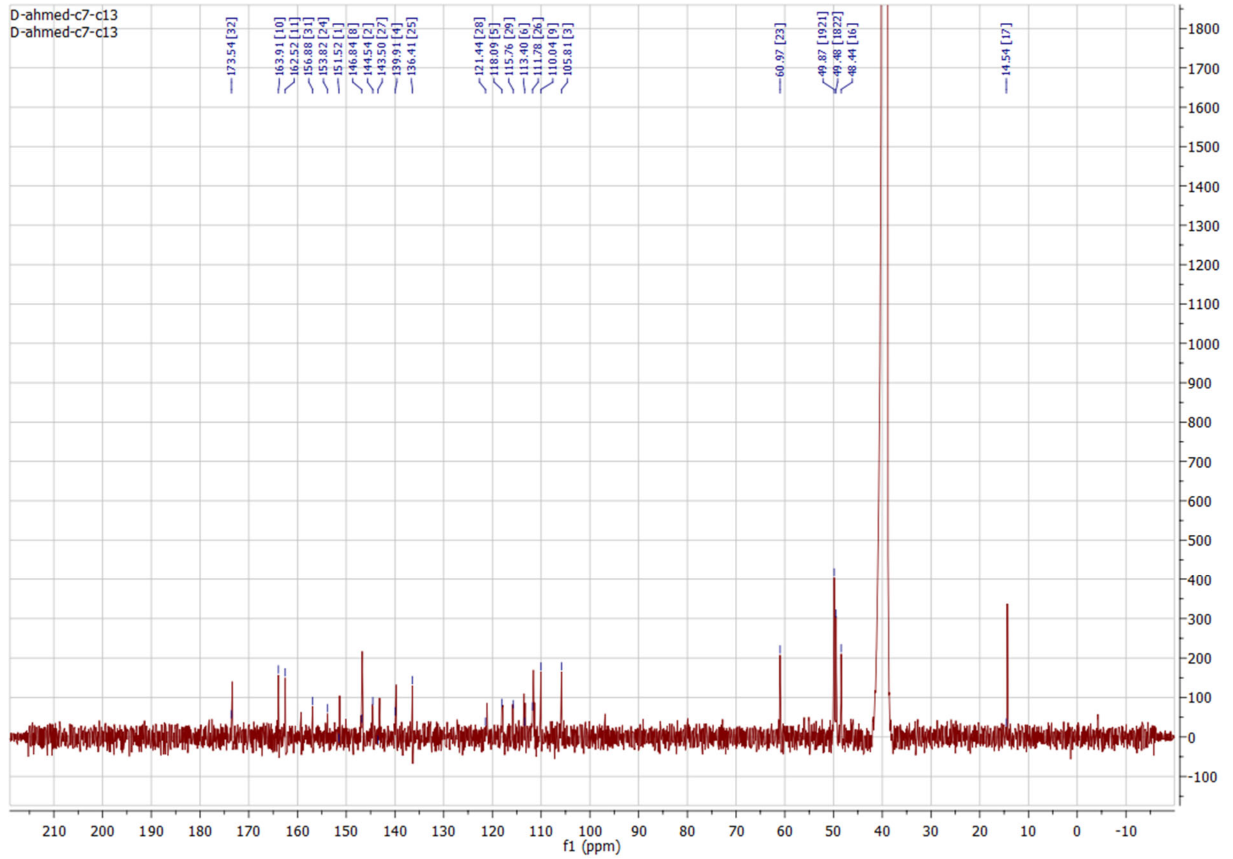

Figure S69: ^{13}C NMR of compound 17c.

17d

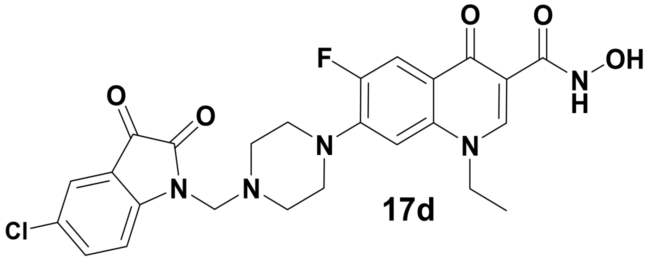

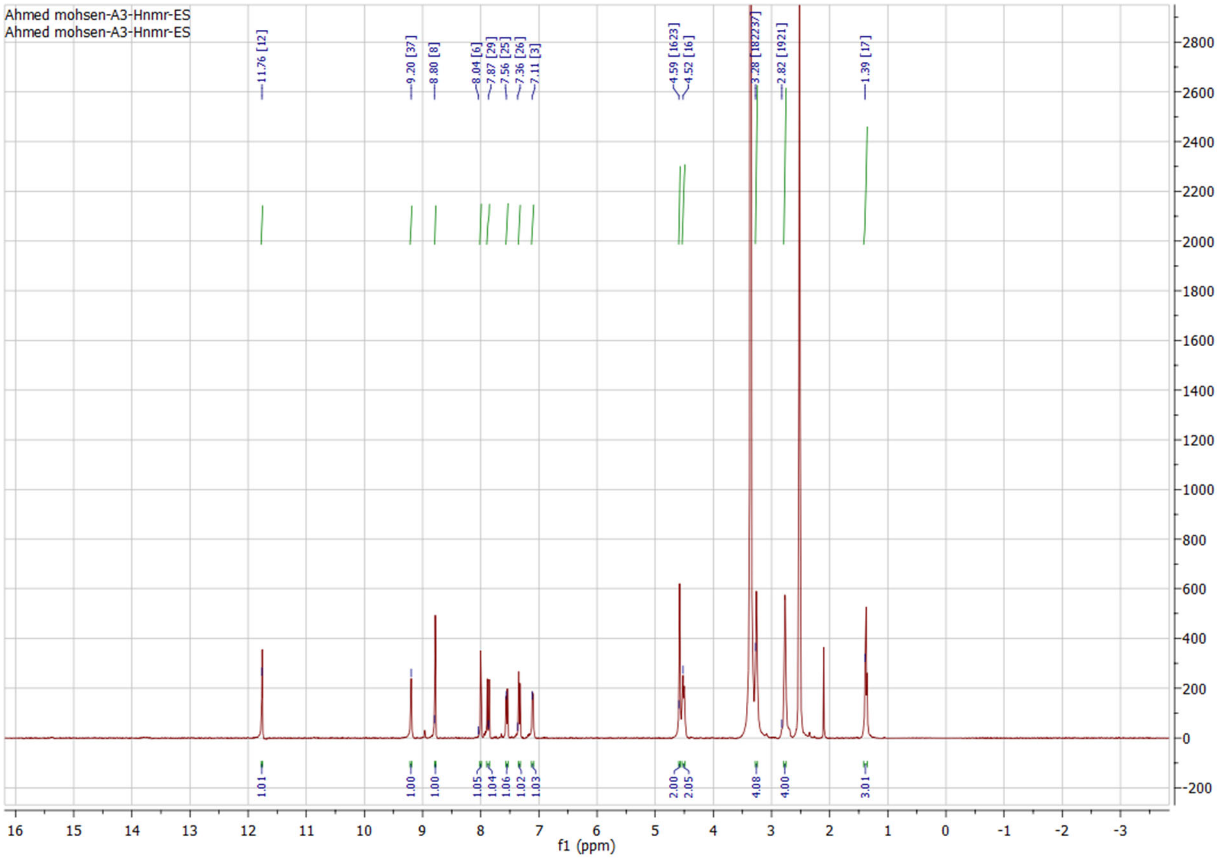

Figure S70: ^{1}H NMR of compound 17d.

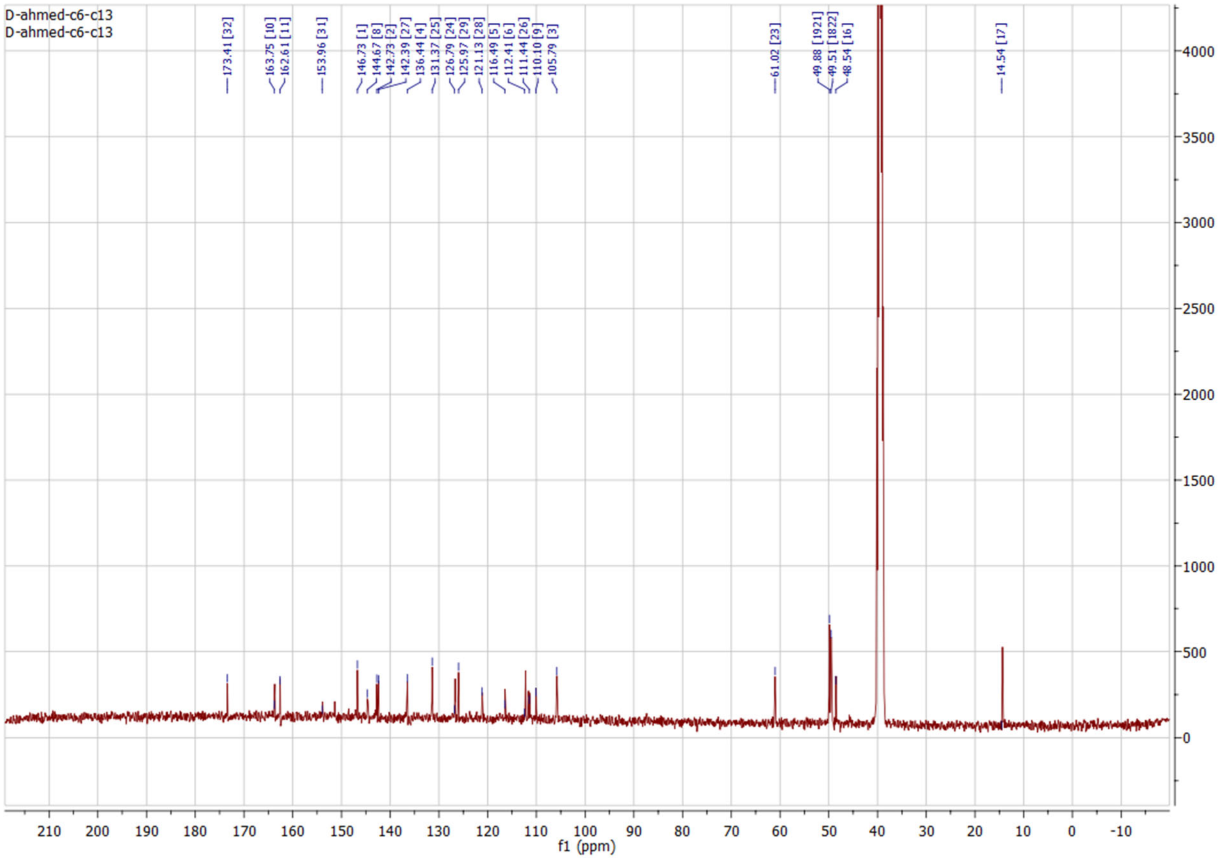

Figure S71: ^{13}C NMR of compound 17d.

17e

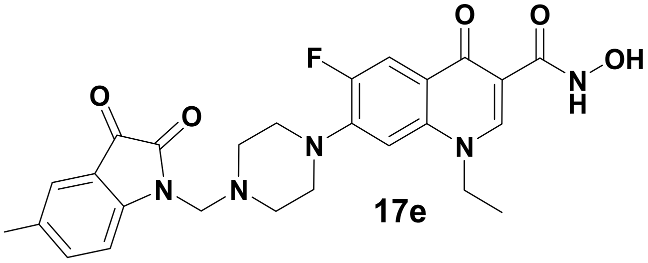

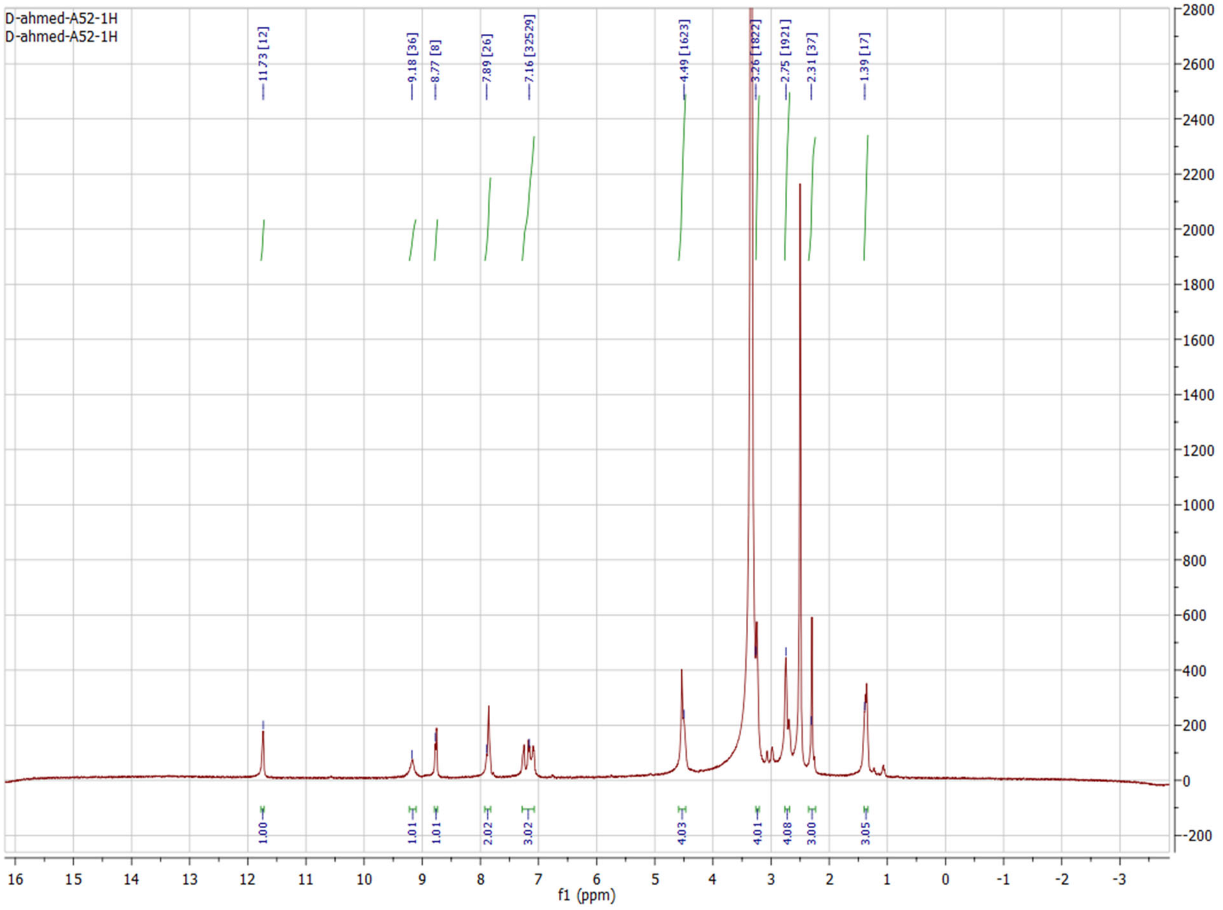

Figure S72: ^{1}H NMR of compound 17e.

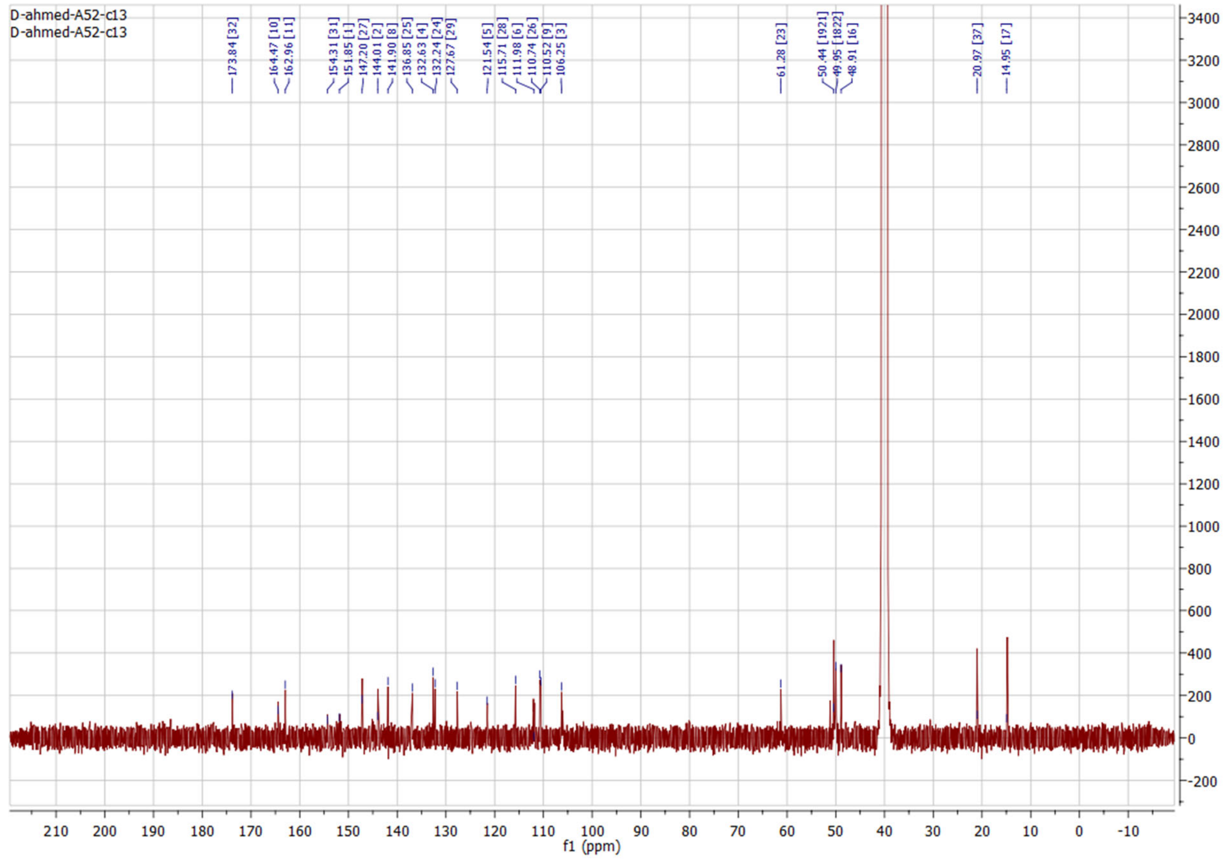

Figure S73: ^{13}C NMR of compound 17e.

17f

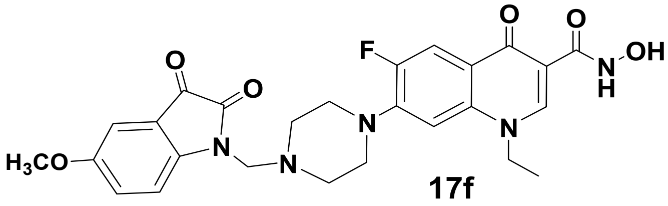

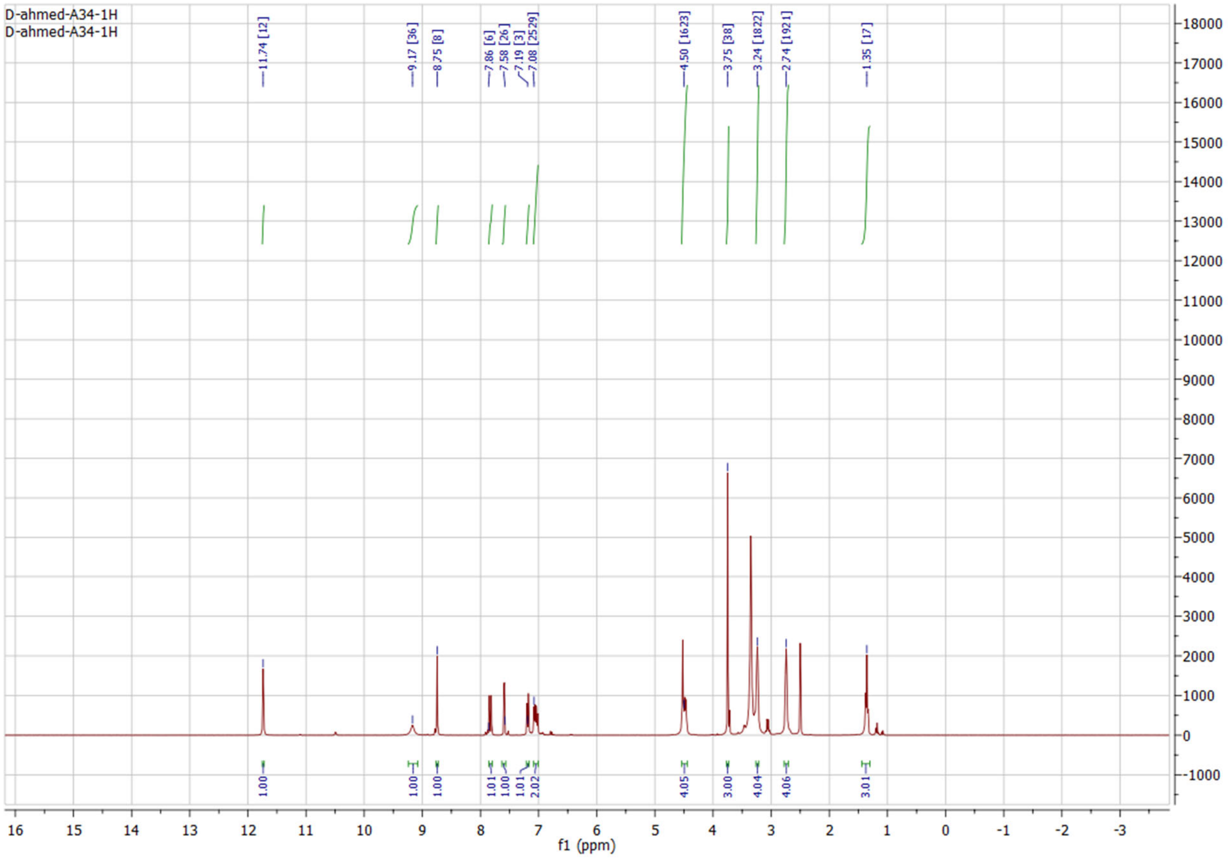

Figure S74: ^{1}H NMR of compound 17f.

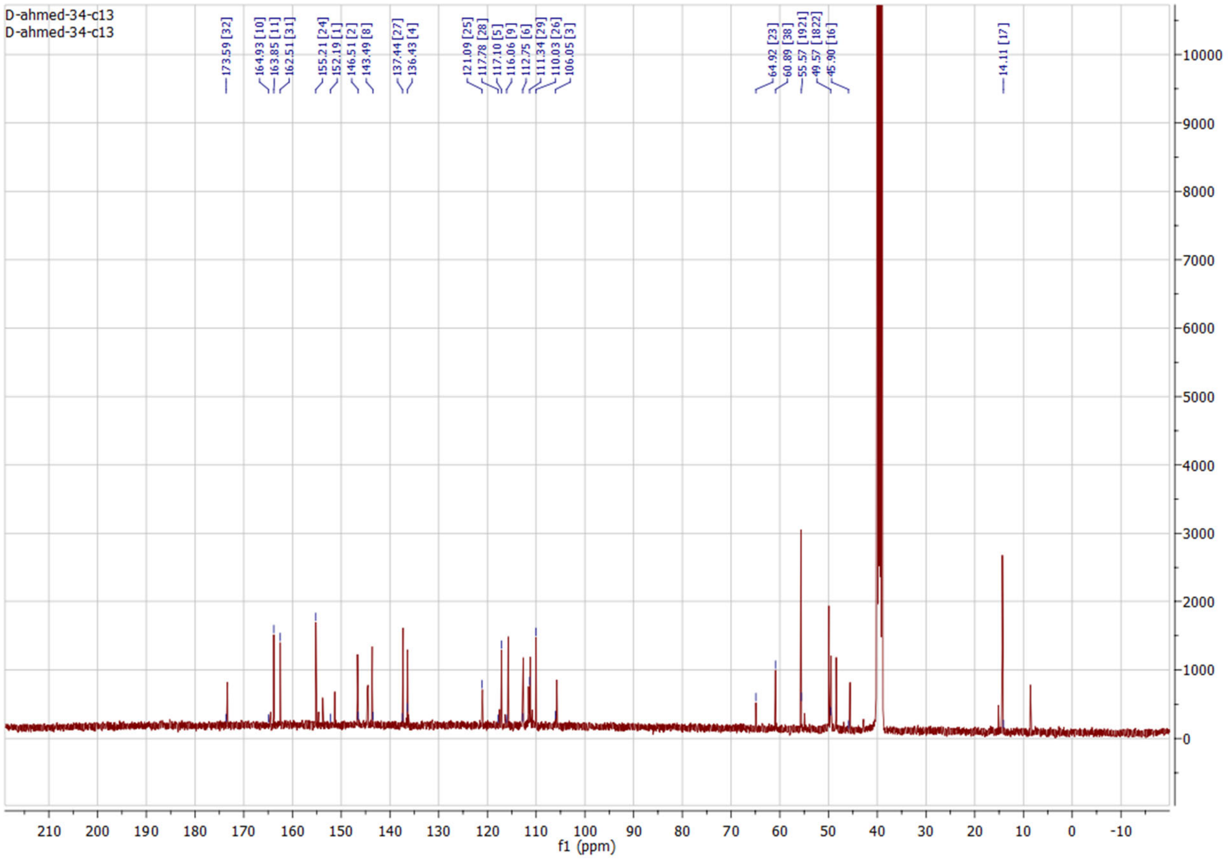

Figure S75: ^{13}C NMR of compound 17f.

19a

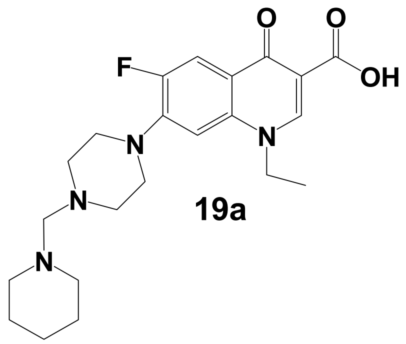

19a

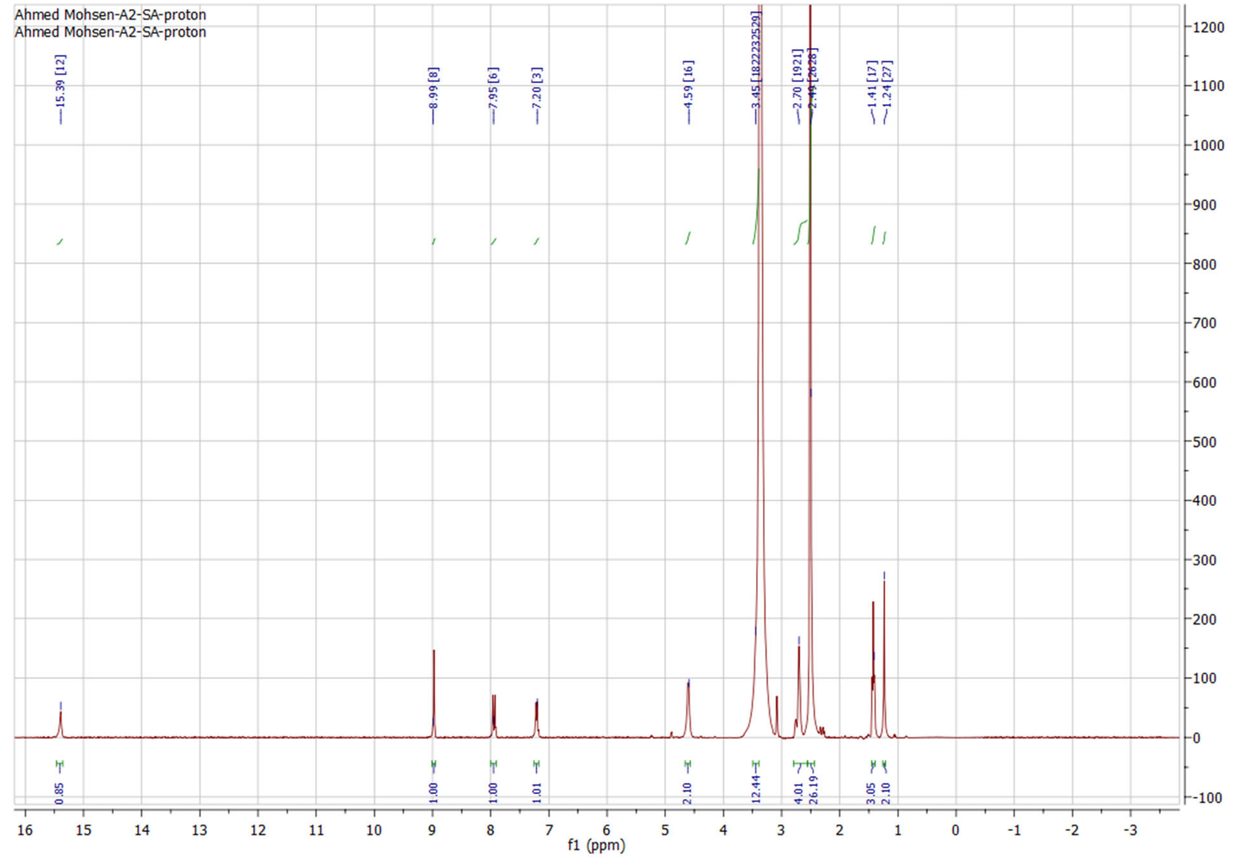

Figure S76: ^{1}H NMR of compound 19a.

19b

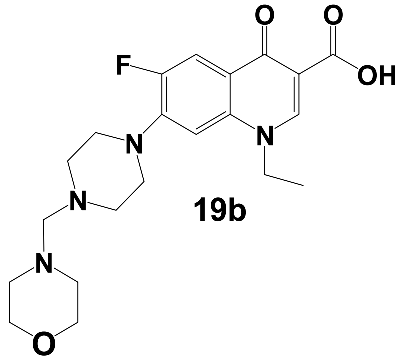

19b

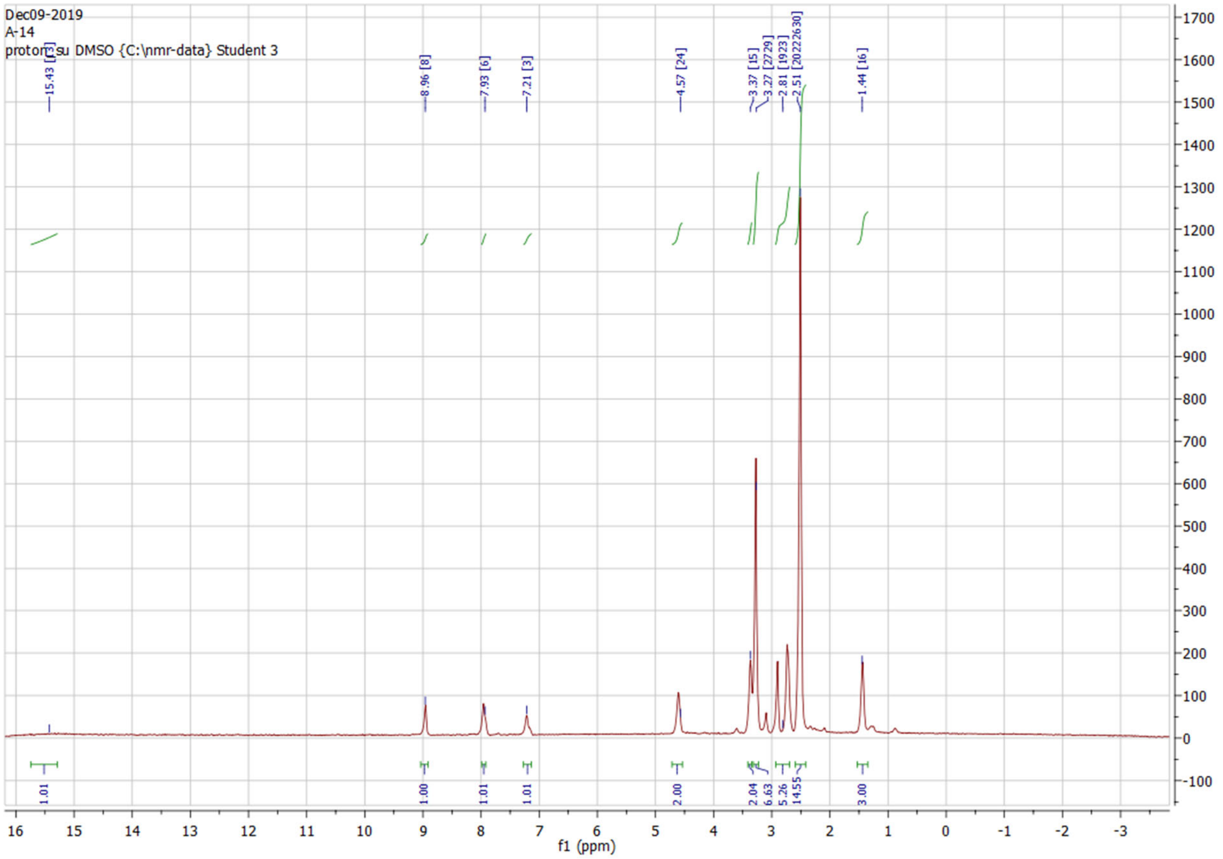

Figure S77: ^{1}H NMR of compound 19b.

20a

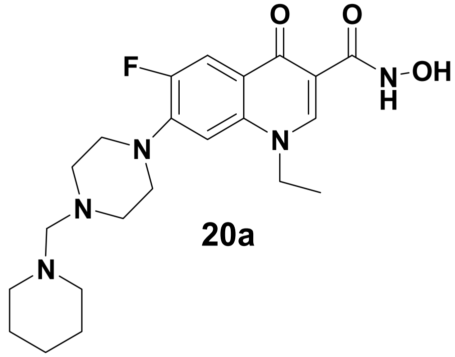

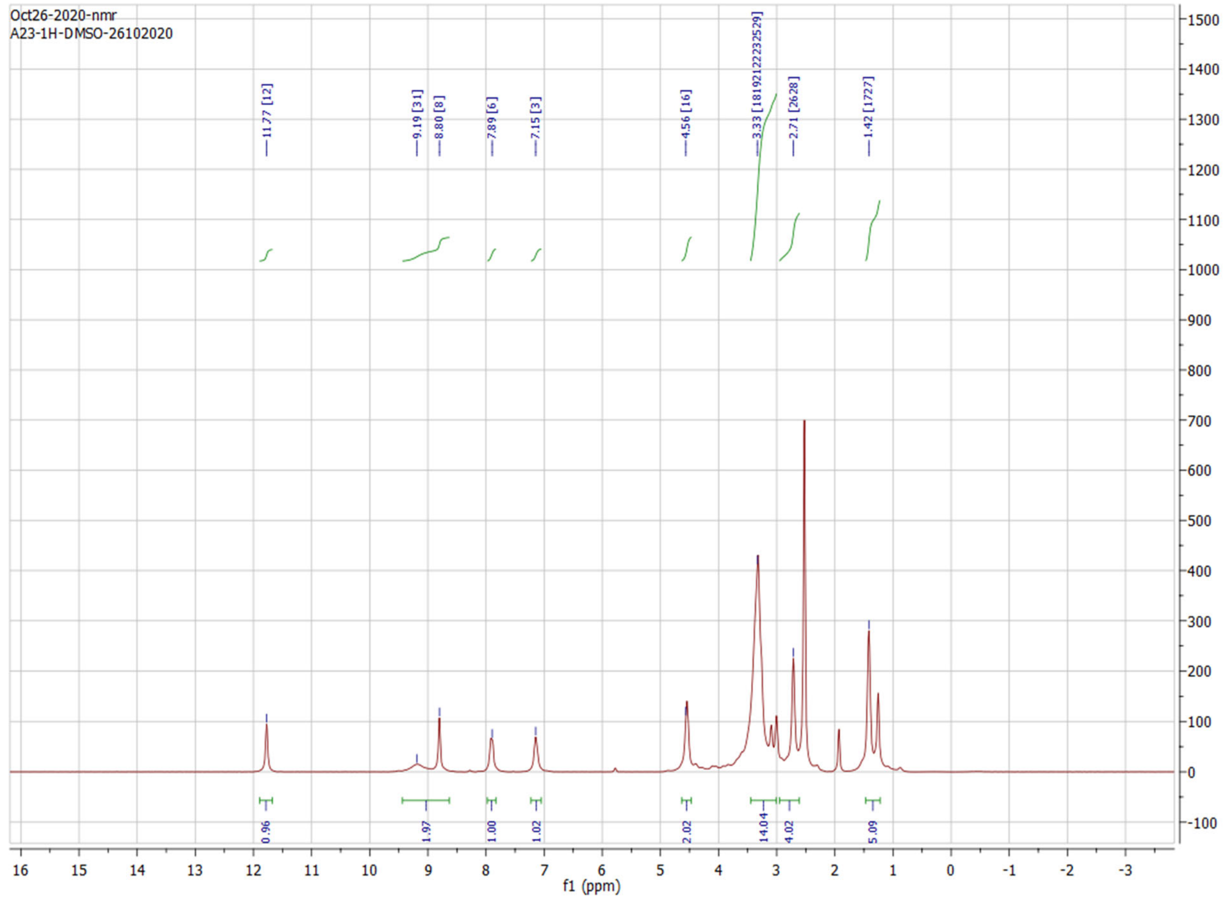

Figure S78: ^{1}H NMR of compound 20a.

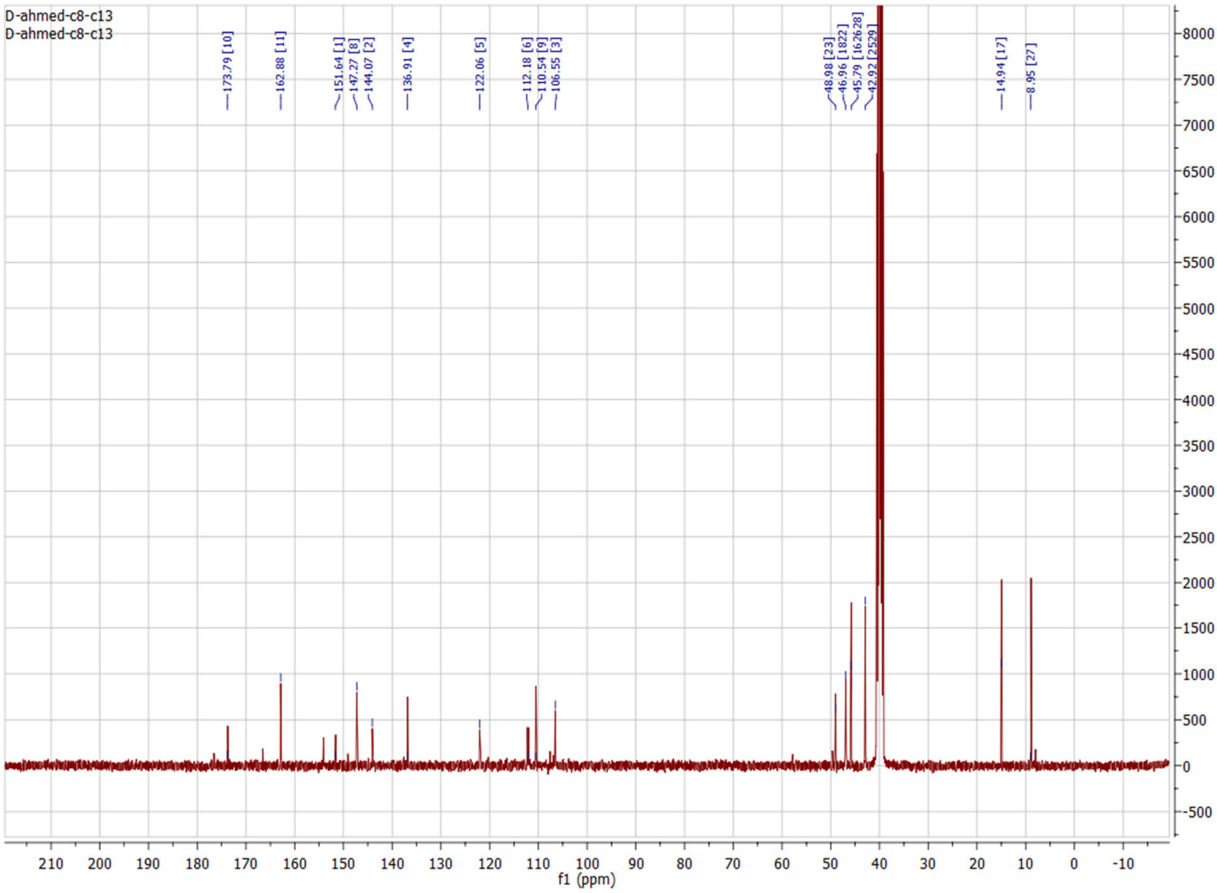

Figure S79: ^{13}C NMR of compound 20a.

20b

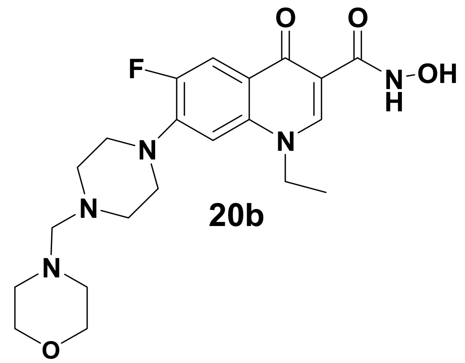

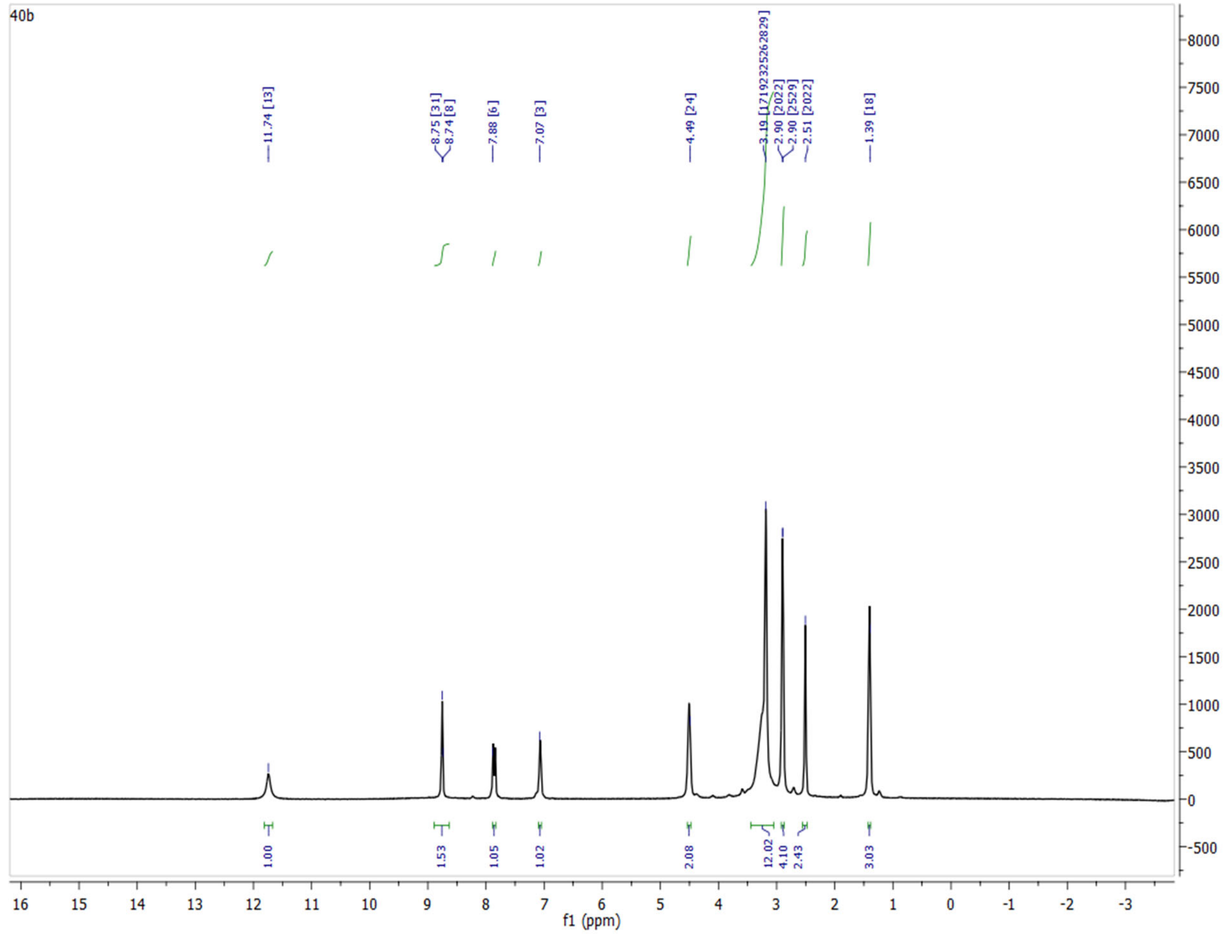

Figure S80: ^{1}H NMR of compound 20b.

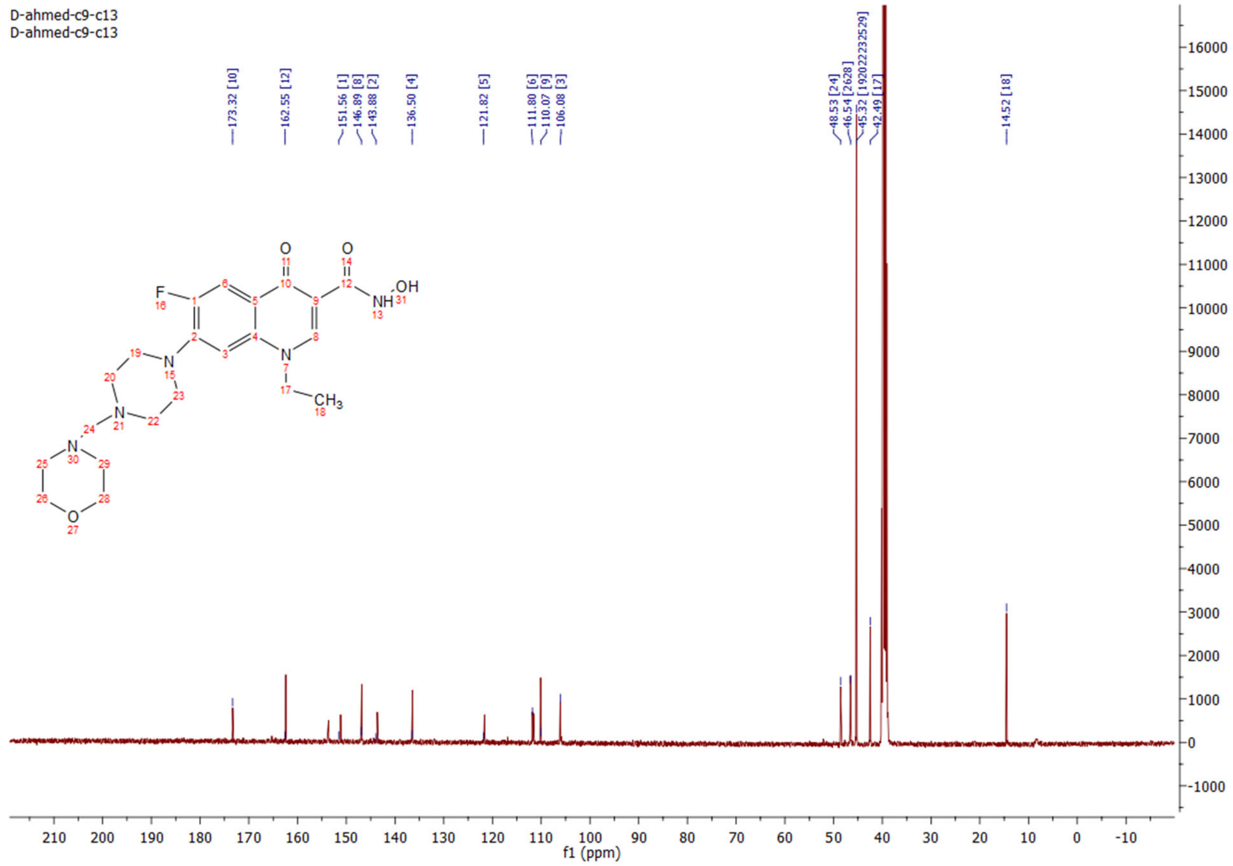

Figure S81: ^{13}C NMR of compound 20b.

22a

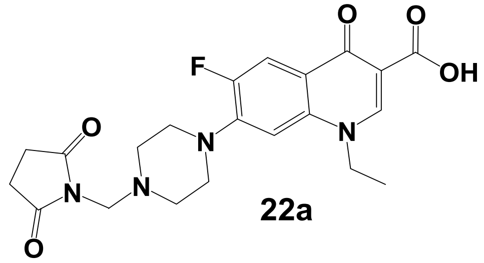

22a

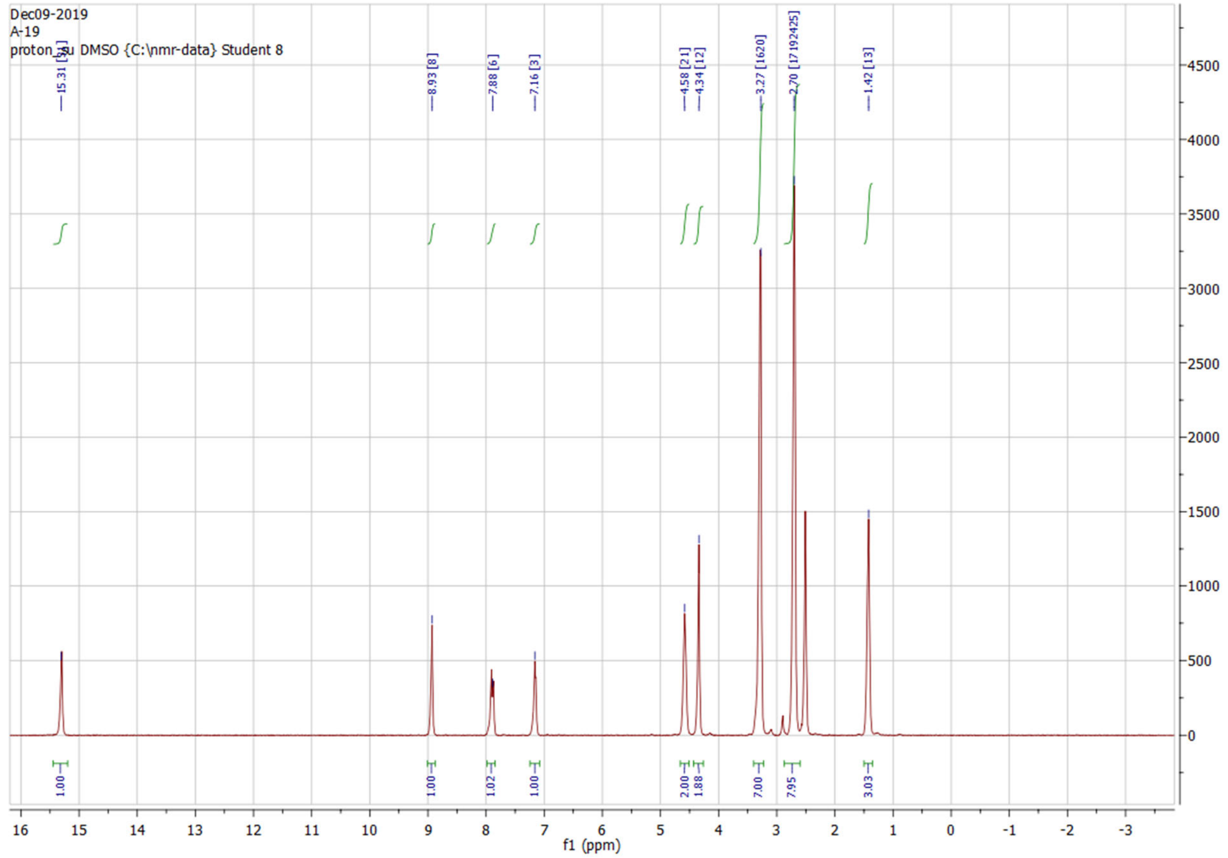

Figure S82: ^{1}H NMR of compound 22a.

22b

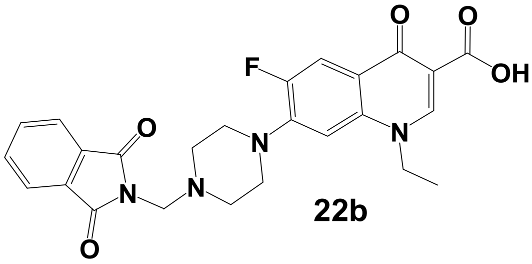

22b

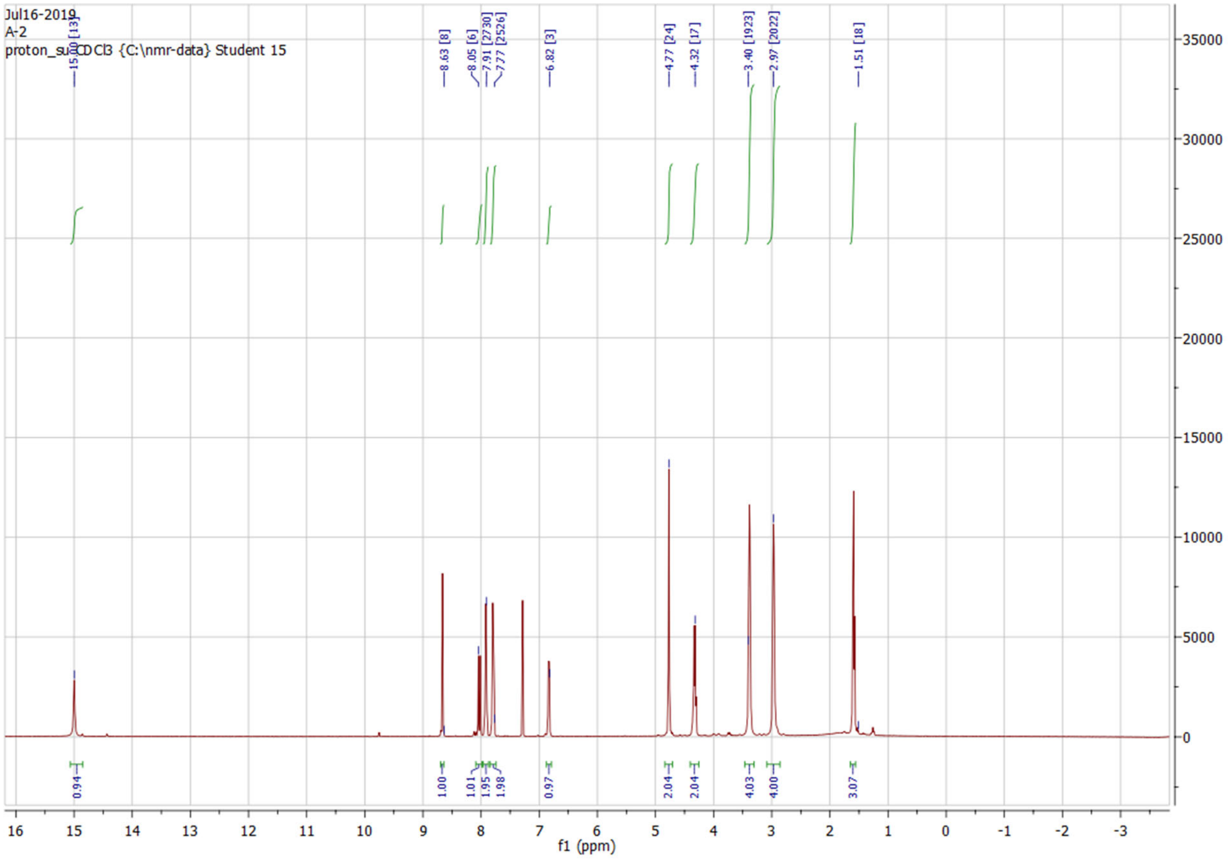

Figure S83: ^{1}H NMR of compound 22b.

23a

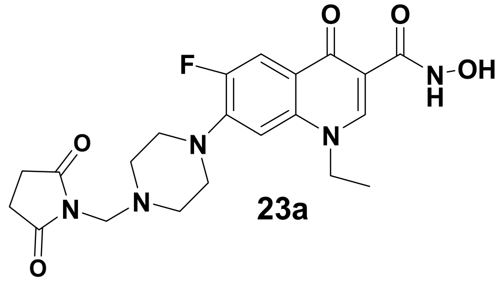

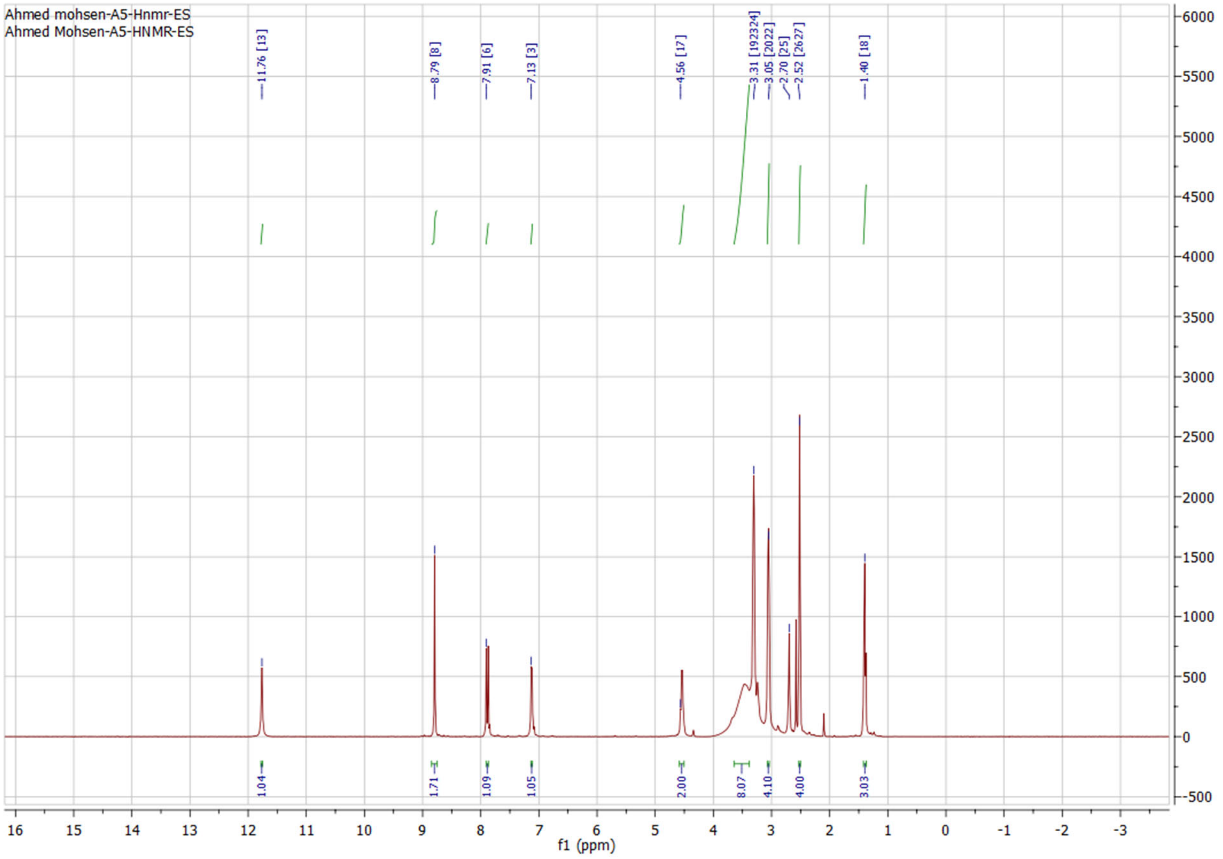

Figure S84: ^{1}H NMR of compound 23a.

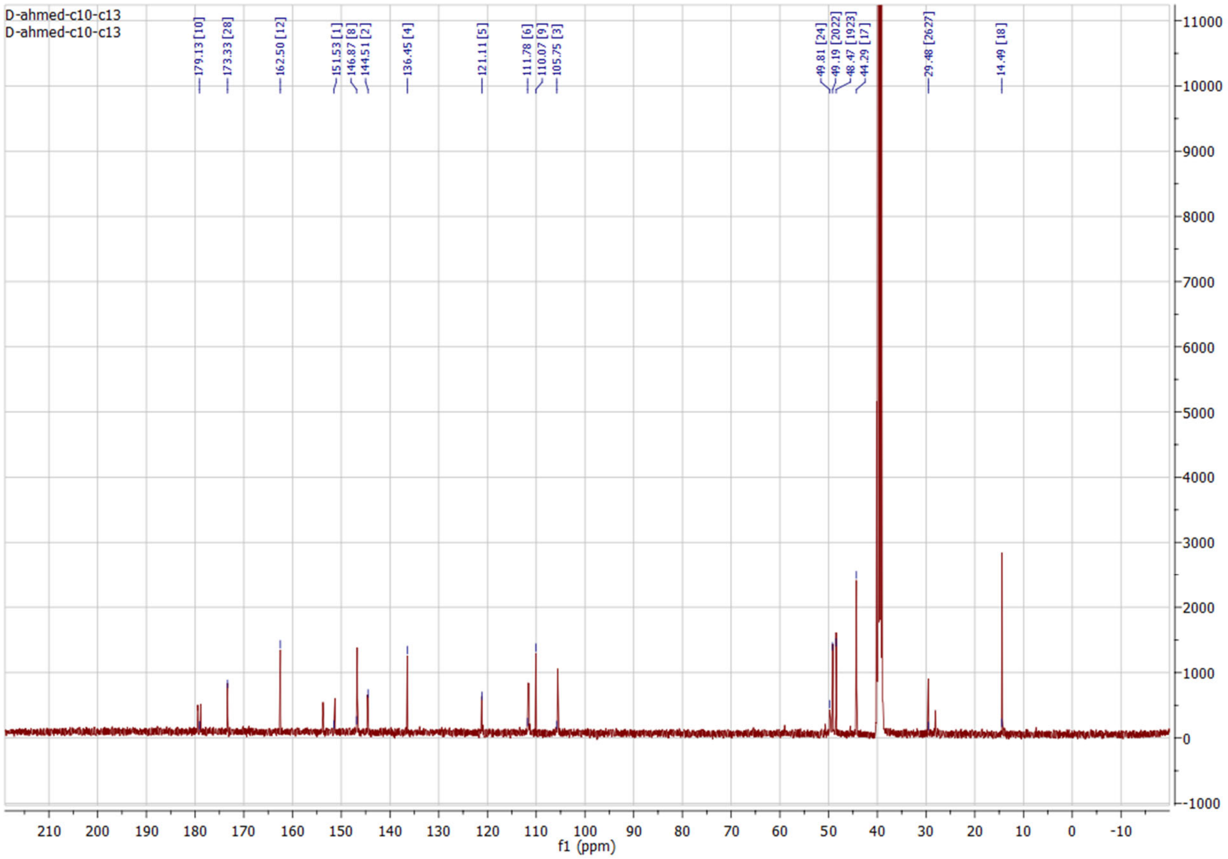

Figure S85: ^{13}C NMR of compound 23a.

23b

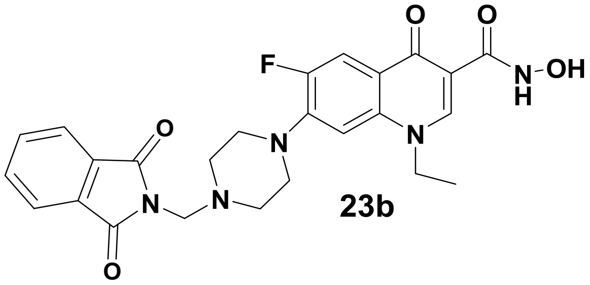

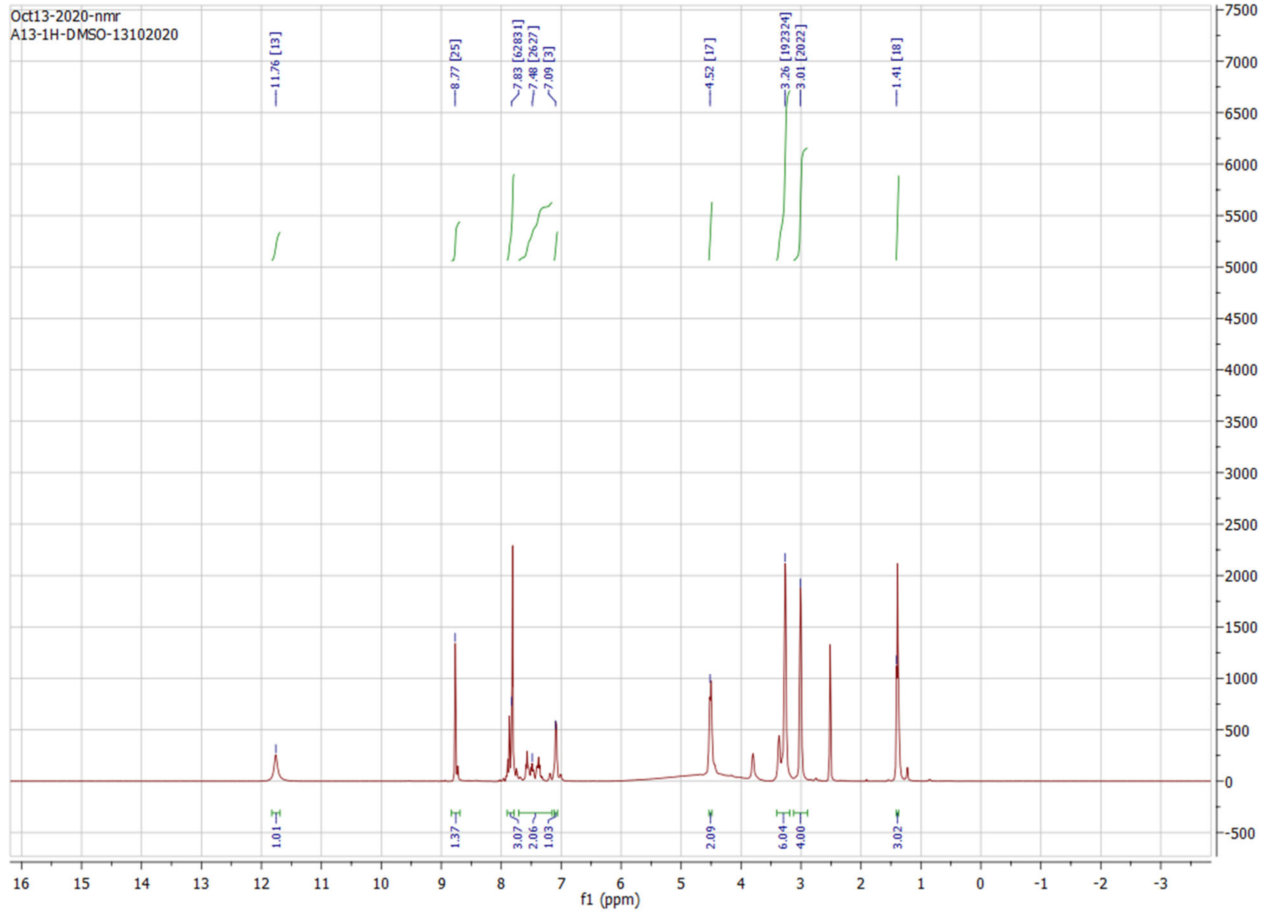

Figure S86: ^{1}H NMR of compound 23b.

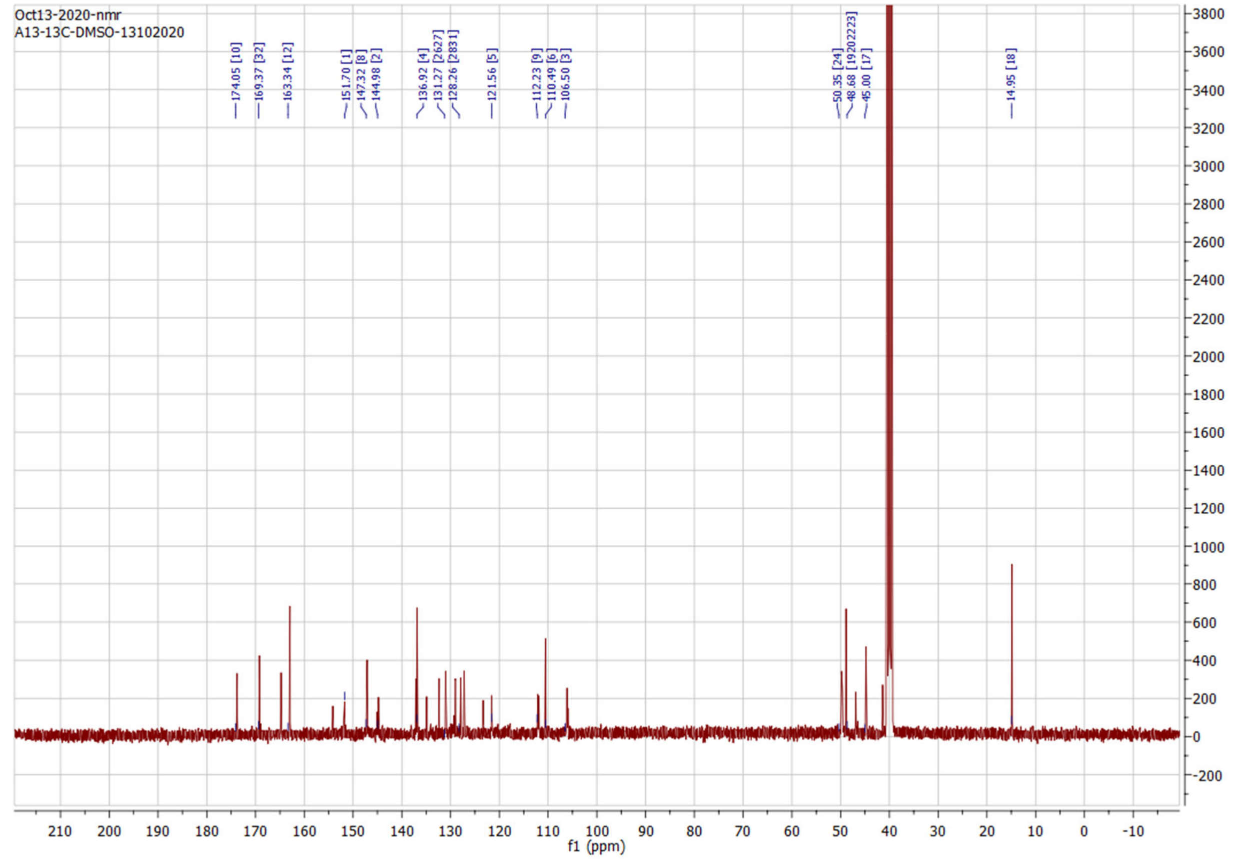

Figure S87: ^{13}C NMR of compound 23b.

25

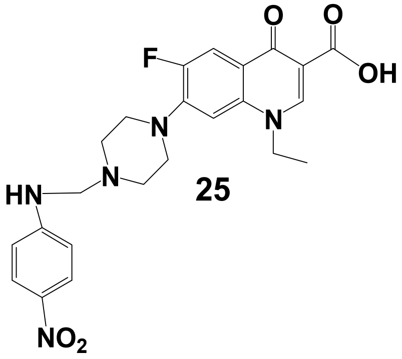

25

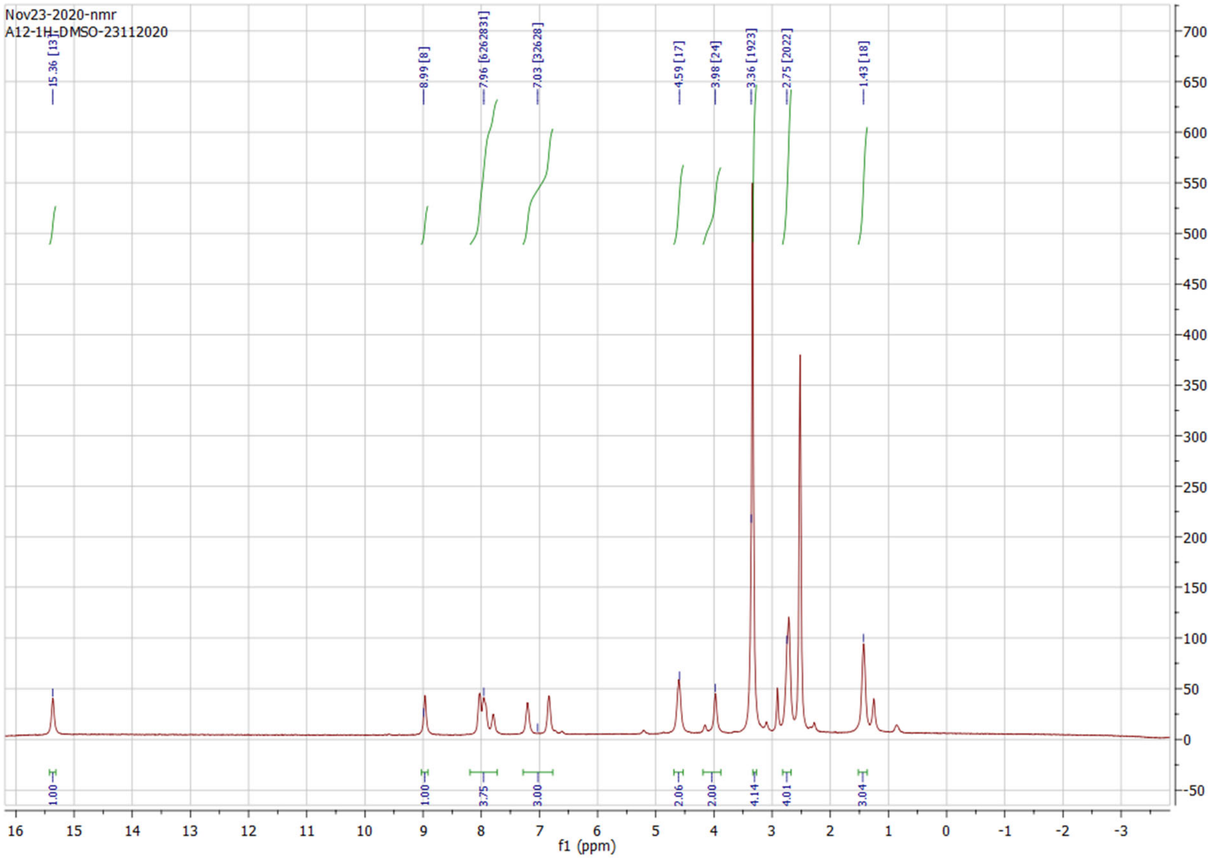

Figure S88: ^{1}H NMR of compound 25.

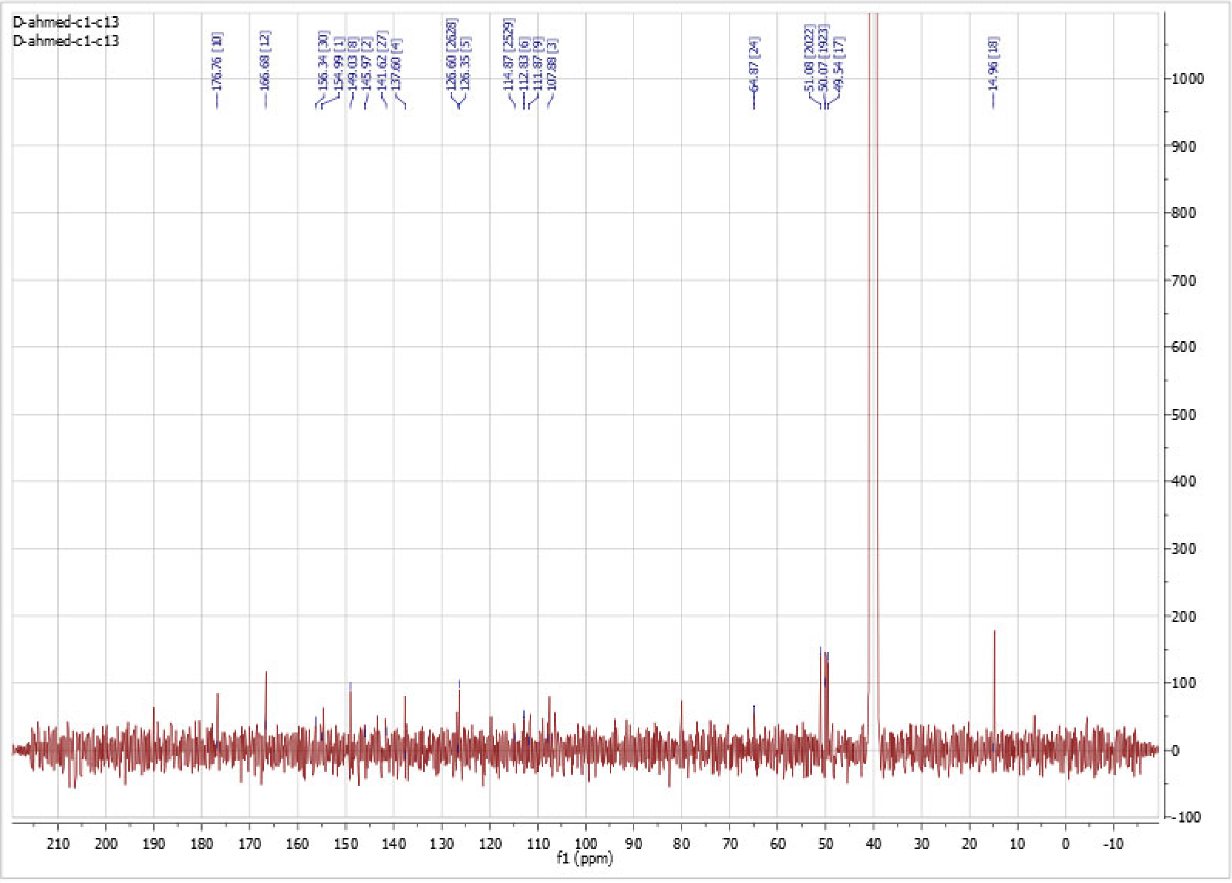

Figure S89: ^{13}C NMR of compound 25.

26

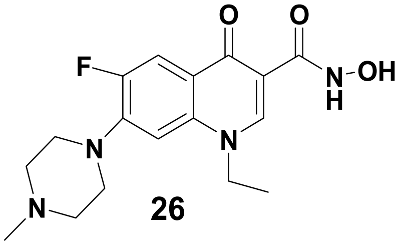

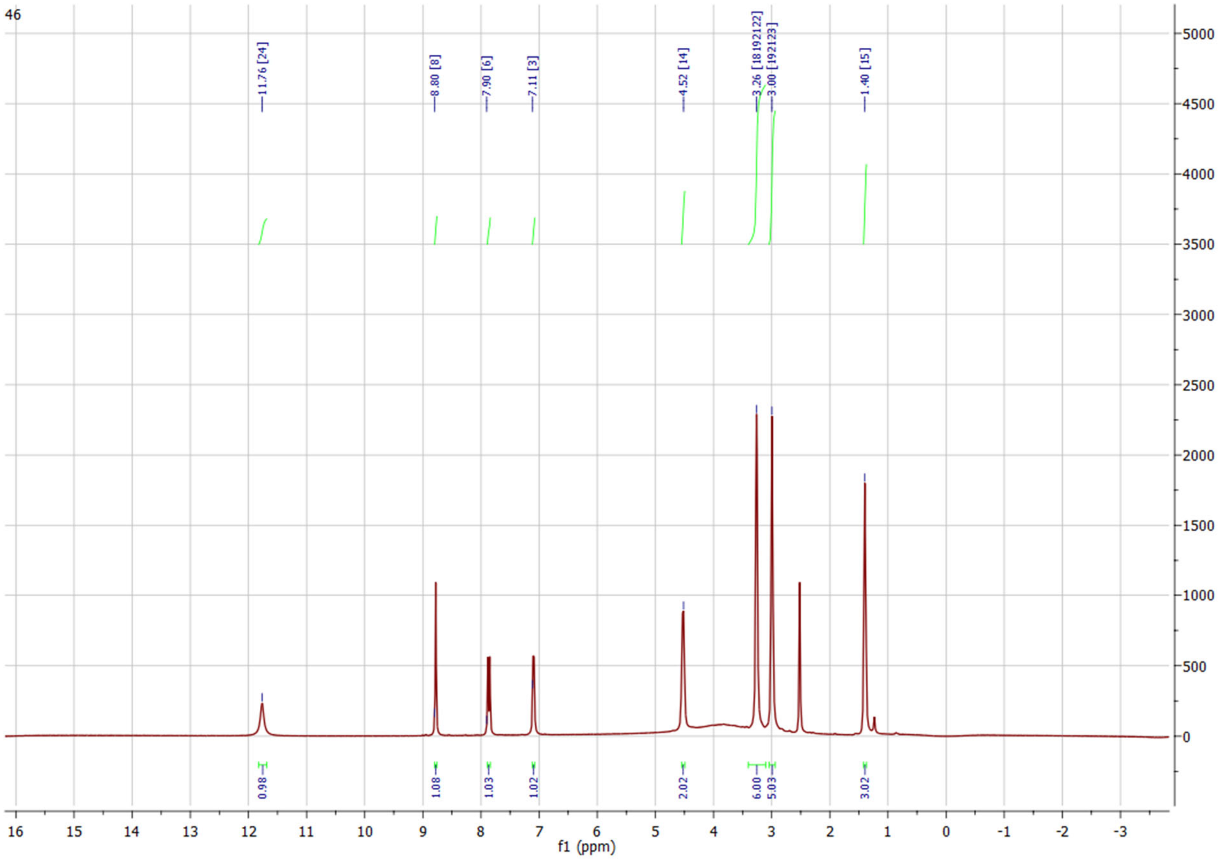

Figure S90: ^{1}H NMR of compound 26.

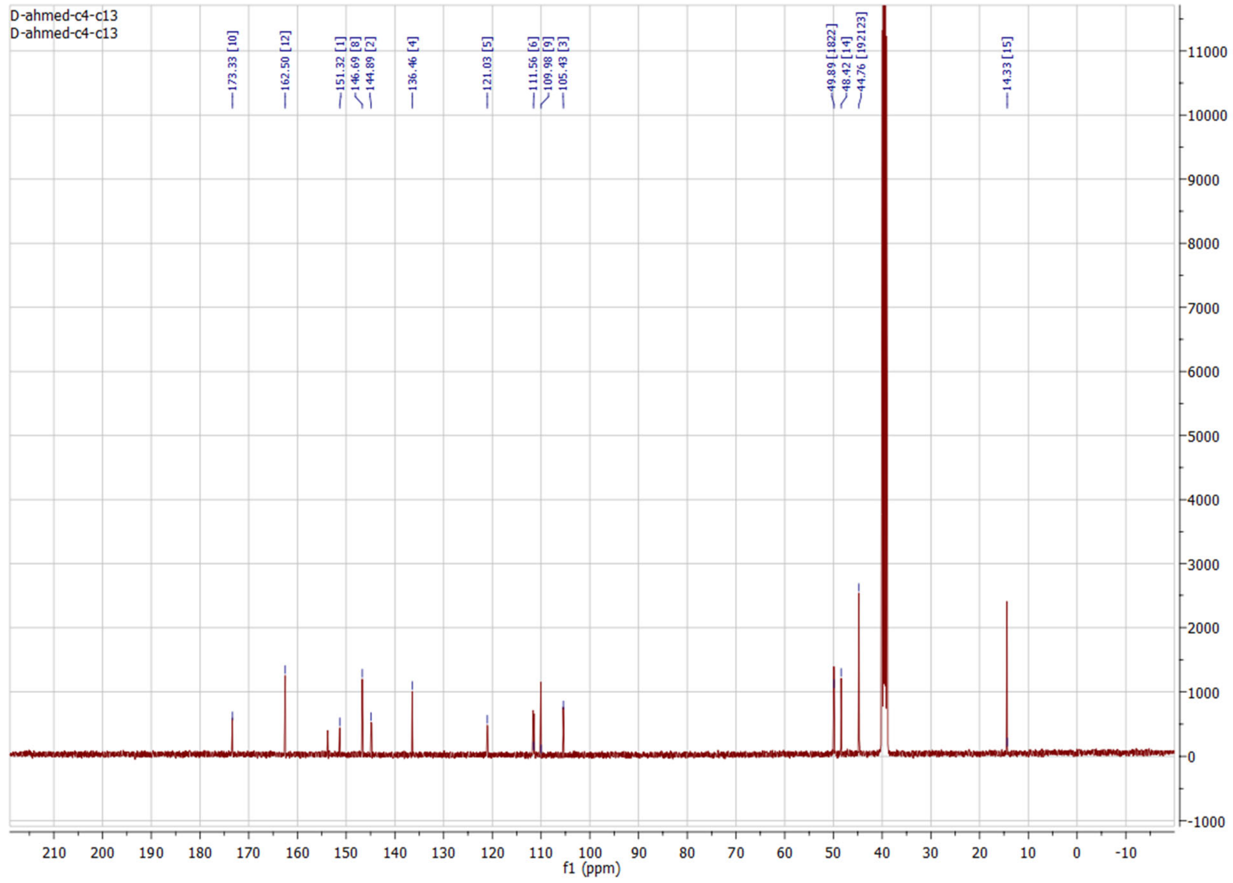

Figure S91: ^{13}C NMR of compound 26.

Al-Azhar University
The Regional Center for Mycology and Biotechnology

Requester Data:
Name: Dr. Ahmed Mohsen Kamal
Authority: Faculty of Pharmacy, Assuit University

Sample Data:
Eighteen samples had been submitted for elemental analysis.

Analysis Report:

Sample Code	C%	H%	N%
10f	62.47	5.38	9.70
12d	59.91	5.05	11.89
5a	57.63	5.88	15.11
5b	52.88	5.04	13.70
8a	62.86	5.40	13.02
8b	61.75	5.46	12.05
8c	58.68	4.87	12.01
8d	55.94	4.96	12.09
8e	56.76	5.28	11.71
11e	65.24	5.87	13.46
11f	60.47	5.41	12.48
11a	58.83	6.12	16.31
11b	59.92	6.56	15.53
11c	60.84	6.35	15.12
13a	63.52	5.73	12.64
13b	54.51	4.68	10.79
13c	64.25	5.95	12.37
13d	58.17	5.02	14.35

INVESTIGATOR DIRECTOR

Al-Azhar University
The Regional Center for Mycology and Biotechnology

Requester Data:
Name: Dr. Ahmed Mohsen Kamal
Authority: Faculty of Pharmacy, Assuit University

Sample Data:
Eleven samples had been submitted for elemental analysis.

Analysis Report:

Sample Code	C%	H%	N%
16a	62.95	5.01	11.97
16b	54.13	4.12	10.29
16c	60.71	4.59	11.46
16d	58.78	4.47	11.14
16e	63.29	5.32	11.50
16f	61.59	5.12	11.28
19a	63.70	7.19	13.62
19b	60.45	6.72	13.58
22a	58.73	5.53	13.18
22b	62.59	4.97	11.95
25	59.11	5.34	15.19

INVESTIGATOR DIRECTOR

11d	62.50	7.37	14.08

Al-Azhar University
The Regional Center for Mycology and Biotechnology

Requester Data:
Name: Dr. Ahmed Mohsen Kamal
Authority: Faculty of Pharmacy, Assuit University

Sample Data:
Eleven samples had been submitted for elemental analysis.

Analysis Report:

Sample Code	C%	H%	N%
26	58.72	6.05	15.91
17a	61.08	5.12	14.37
17b	52.29	4.31	12.48
17c	58.98	4.70	13.85
17d	57.14	4.51	13.44
17e	61.37	5.40	14.07
17f	59.88	5.13	13.52
20a	61.48	7.09	16.17
20b	58.40	6.73	16.42
23a	56.89	5.61	15.88
23b	60.73	5.12	14.37

INVESTIGATOR DIRECTOR

Figure S92: Elemental analysis

3. Prediction of ADME/Tox

Text S2: Prediction of physicochemical properties

Physicochemical properties are a complex balance of various structural features which determine whether a particular molecule is similar to the known drugs or not. Hydrophobicity, molecular size, flexibility, and presence of various pharmacophoric features are the main physical properties that influence the behavior of molecules in a living organism. Good bioavailability can be achieved with an appropriate balance between solubility and partitioning properties. In addition, topological polar surface area (TPSA) and number of rotatable bonds (nrotb) have been linked to drug bioavailability[11].

The compliance of the newly synthesized compounds to Lipinski's and Veber's rules of oral bioavailability was estimated using MOE 2020.01[12]. According to Lipinski's rule of five, a compound with a molecular mass under 500 Dalton (MW), a coefficient of partition between octanol and water (LogP(o/w)) lower than 5, no more than five hydrogen bond donors (lip_don) and no more than 10 hydrogen bond acceptors (lip_acc) could be a good drug candidate. Veber's rule states that a compound with 10 or fewer rotatable bonds (b_rotN) and a polar surface area (TPSA) no greater than 140 Å^2 should present good oral bioavailability[13]. The results (**Table S1**) showed that except for compound **17f** the tested derivatives are in accordance with Lipinski's and Veber's rules with high probability of reasonable oral absorption.

The molecular properties of the newly synthesized compounds were calculated using MOE software program and compared to the values of our reference drug norfloxacin (Table). TPSA was calculated based on the methodology published by Ertl *et al.*[14] as the surface areas that are occupied by oxygen and nitrogen atoms and by hydrogen atoms attached to them. It is considered a good descriptor for drug absorption, including intestinal absorption, bioavailability, and blood-brain barrier penetration. Molecules with TPSA values around 140 Å^2 or more are expected to exhibit poor intestinal absorption[11]. Results shown in Table S1 and indicate that most of the synthesized compounds have TPSA values < 140 Å2. Thus, they are expected to have good intestinal absorption. Molecules with more than 10 rotatable bonds may have problems with bioavailability[11]. Most of the compounds under investigation have

between three and ten rotatable bonds suggesting good bioavailability. LogP values are based on summation of fragment-based contributions and correction factors. It has been shown that for the compound to have a reasonable probability of being well-absorbed, LogP values must be in the range of −0.4 to 5[11]. All the tested compounds were found to have LogP values within the acceptable range and are thus expected to have reasonable oral absorption.

Table S1: Physicochemical parameters of norfloxacin derivatives.

Code	Lip_acc	Lip_don	Lip_druglike	LogP(O/W)	LogS	TPSA	Weight	b_rotN	Lip. violation
**Nor**	6	2	1	0.7250	-2.5094	72.879	319.33	3	0
**16a**	9	1	1	1.454	-4.690	101.47	478.479	5	0
**16b**	9	1	1	2.289	-5.7804	101.47	557.375	5	1
**16c**	9	1	1	1.644	-4.9850	101.47	496.47	5	0
**16d**	9	1	1	2.08299	-5.4243	101.47	512.924	5	1
**16e**	9	1	1	1.789	-5.1639	101.47	492.506	5	0
**16f**	10	1	1	1.447	-4.7404	110.69	508.505	6	1
**19a**	7	1	1	1.954	-2.9979	67.33	416.496	5	0
**19b**	8	1	1	0.5460	-2.5352	76.559	418.468	5	0
**22a**	9	1	1	0.10199	-2.7124	101.47	430.436	5	0
**22b**	9	1	1	1.88399	-4.690	101.47	478.479	5	0
**25**	10	1	1	2.421999	-4.9873	124.76	468.464	7	0
**26**	7	3	1	0.0460	-2.5955	84.910	334.350	4	0
**17a**	10	2	1	0.774999	-4.7761	113.5	493.494	6	0
**17b**	10	2	1	1.610	-5.8665	113.5	572.390	6	1
**17c**	10	2	1	0.964999	-5.0711	113.5	511.484	6	1
**17d**	10	2	1	1.404	-5.5104	113.5	527.94	6	1
**17e**	10	2	1	1.110	-5.250	113.5	507.522	6	1
**17f**	11	2	0	0.7680	-4.8265	122.73	523.520	7	2
**20a**	8	2	1	1.27499	-3.0840	79.360	431.51	6	0
**20b**	9	2	1	-0.1330	-2.6213	88.589	433.483	6	0
**23a**	10	2	1	-0.5770	-2.7985	113.5	445.450	6	0
**23b**	10	2	1	1.2050	-4.7761	113.5	493.494	6	0
**4a**	7	1	1	0.56599	-2.8164	81.160	361.372	4	0
**4b**	7	1	1	0.9940	-3.5743	81.160	395.817	5	0
**7a**	7	1	1	2.227999	-4.5769	81.160	423.444	5	0
**7b**	8	1	1	2.1840	-4.6273	90.389	453.47	6	0
**7c**	7	1	1	2.81999	-5.3112	81.160	457.888	5	0
**7d**	8	1	1	1.47800	-4.4503	98.230	459.497	5	0
**7e**	8	1	1	1.77600	-4.9243	98.230	473.524	5	0
**10e**	6	1	1	2.78099	-4.4082	64.089	409.460	5	0
**10f**	6	1	1	3.37299	-5.1425	64.089	443.905	5	0
**10a**	6	1	1	0.99299	-2.6403	64.089	333.363	3	0
**10b**	6	1	1	1.333999	-2.9675	64.089	347.389	4	0
**10c**	6	1	1	1.654999	-3.1366	64.089	359.401	5	0
**12a**	7	1	1	2.33599	-4.7396	81.160	437.470	6	0
**12b**	7	1	1	3.1340	-5.830	81.16	516.367	6	1
**12c**	7	1	1	2.6340	-5.2135	81.160	451.497	6	0
**12d**	10	1	1	2.27099	-5.5298	126.98	482.467	7	0
**10d**	6	1	1	2.8320	-4.1997	64.089	389.470	7	0
**5a**	8	2	1	-0.11299	-2.9025	93.190	376.388	5	0
**5b**	8	2	1	0.314999	-3.6604	93.190	410.832	6	0
**8a**	8	2	1	1.5490	-4.6630	93.190	438.458	6	0
**8b**	9	2	1	1.505	-4.7134	102.41	468.484	7	0
**8c**	8	2	1	2.1410	-5.3973	93.190	472.903	6	0
**8d**	9	2	1	0.7990	-4.5364	110.26	474.513	6	0
**8e**	9	2	1	1.097	-5.0104	110.26	488.539	6	0
**11e**	7	2	1	2.102	-4.4943	76.120	424.475	6	0

**11f**	7	2	1	2.6940	-5.2286	76.120	458.920	6	0
**11a**	7	2	1	0.3140	-2.7264	76.120	348.377	4	0
**11b**	7	2	1	0.654999	-3.0536	76.12	362.404	5	0
**11c**	7	2	1	0.9760	-3.2227	76.120	374.415	6	0
**13a**	8	2	1	1.65699	-4.8257	93.190	452.485	7	0
**13b**	8	2	1	2.45499	-5.9161	93.190	531.382	7	0
**13c**	8	2	1	1.9550	-5.2996	93.190	466.513	7	0
**13d**	11	2	1	1.5920	-5.6159	139.01	497.483	8	1
**11d**	7	2	1	2.150	-4.2859	76.12	404.485	8	0

Text S3: ADME/Tox prediction using pKCSM lab.

Absorption, distribution, metabolism, and excretion (ADME) as well as toxicity are crucial parameters to be considered in drug design. *in silico* predictions of these parameters can help selecting the most promising compounds without the need to large-scale experiments. pkCSM is a platform for the analysis and optimization of pharmacokinetic and toxicity properties implemented in a user-friendly, freely available web interface. It can assist in finding a balance between potency, safety, and pharmacokinetic properties. Predicted values for norfloxacin and its derivatives are displayed in **Table S2**.

1- Caco-2 permeability (log Papp in 10^{-6} cm/s)

Caco-2 permeability assay measures the rate of flux of a compound across polarized Caco-2 monolayers and can be used to predict *in vivo* absorption of drugs. The Caco-2 cell line is derived from a human colon carcinoma and resembles intestinal epithelial cells [15].

Values of log Papp and its indication [16]:

A. Log Papp ≤ 10^{-6} cm/s indicates low intestinal absorption (0-20%).
B. Log Papp 10^{-6}-10 x 10^{-6} cm/s indicates medium intestinal absorption (20-70%).
C. Log Papp > 10 x 10^{-6} cm/s indicates high intestinal absorption (70-100%).

2- Steady state volume of distribution (VDss)

Steady state volume of distribution (VDss) reflects the blood and tissue volume, into which a drug is distributed and the relative binding of a drug to proteins in these spaces[17].

A drug with a high VD has a propensity to leave the plasma and enter the extravascular compartments of the body, meaning that a higher dose of a drug is required to achieve a given plasma concentration (high VD -> more distribution to other tissues). Conversely, a drug with a low VD has a propensity to remain in the plasma meaning a lower dose of a drug is required to achieve a given plasma concentration (low VD -> less distribution to other tissue)[18].

3- Blood-brain barrier permeability (Log BB)

The most common parameter used to quantify penetration of a compound across the blood-brain barrier (BBB) is the ratio of the concentration of compound measured in the brain to the concentration of compound measured in the blood at steady state. This ratio is expressed as logBB (log[brain]/[blood]) and determines the total extent of brain exposure, at a steady state. Values of logBB can be used to determine if the compound is either BBB+ (crosses the BBB) or BBB− (does not cross the BBB)[19].

4- Metabolism

No metabolizing enzyme was predicted for norfloxacin and compound **26**, while all other synthesized compounds are predicted to be metabolized by the enzymes CYP3A4 and CYP2D6 enzymes.

5- Total body clearance

Clearance describes the volume of plasma, from which a drug would be totally removed per unit of time. Clearance is a measure of the body's ability to remove a drug by either metabolism or excretion. It is the parameter that determines total systemic exposure to a drug, which is simply the ratio of dose/clearance. Total body clearance is the sum of all processes, by which drugs are removed from the body or inactivated, primarily renal excretion and metabolism[20, 21]. The primary application of clearance is dose adjustment in patients. Low clearance indicates high systemic exposure and high clearance indicates low systemic exposure. Thus, adverse drug events, which can be related to overexposure, would be expected more often in patients with low clearance[21].

6- Toxicity

A. Oral rat acute toxicity (LD_{50})

Norfloxacin has a predicted (median lethal dose) LD_{50} equal to 2.139 mol/kg, while is lower than the LD_{50} of all newly synthesized compounds, indicating that the new compounds could be safer than norfloxacin.

B. Oral rat chronic toxicity

It is predicted that norfloxacin induces chronic toxicity with a dose equal to 1.153 mg/kg-bw/day, which is lower than that of most newly synthesized compounds.

C. Hepatotoxicity

Norfloxacin, like other fluoroquinolones, is associated with a low rate of serum enzyme elevations during therapy (1% to 3%). These abnormalities are generally mild, asymptomatic, and transient. Norfloxacin has also been linked to rare but occasionally severe and even fatal cases of acute liver injury[22, 23]. This hepatotoxicity is an important factor in the design of novel fluoroquinolones. Both norfloxacin and its derivatives were predicted to induce hepatotoxicity.

D. Ames toxicity

The Ames test is used to assess potential carcinogenic effects of chemicals by using a histidine-auxotrophic strain of *Salmonella typhimurium*. Reversal to a histidine-prototrophic phenotype is an indication of mutation rate[24]. Both norfloxacin and its derivatives are predicted to not be carcinogenic or mutagenic with the exception of **5a**, **5b**, **11c**, **11d**, **26**, **20a**, **20b**, and **23a**.

Table S2: ADME/TOX properties predicted by pKCSM.

Compound	Caco2 permeability (log Papp in 10^{-6} cm/s)	Steady state volume of distribution	Fraction unbound (human)	BBB permeability (log BB)	Total Clearance (log ml/min/kg)	Oral Rat Acute Toxicity (LD50)	Oral Rat Chronic Toxicity	Hepatotoxicity	AMES toxicity	Metabolizing enzyme
**Nor**	0.363	-0.201	0.478	-.0559	0.356	2.139	1.153	Yes	No	No predicted
**10f**	1.251	-0.007	0.144	-0.122	0.344	2.539	0.971	Yes	No	CYP2D6<br>CYP3A4
**12d**	0.507	-0.402	0.082	-0.954	0.12	2.314	1.558	Yes	No	CYP3A4
**5a**	0.21	-0.508	0.338	-1.193	0.531	2.721	1.189	Yes	Yes	CYP3A4
**5b**	0.228	-0.499	0.286	-3.481	0.268	2.892	1.309	Yes	Yes	CYP3A4
**8a**	0.673	-0.41	0.066	-1.162	0.381	2.828	1.312	Yes	No	CYP3A4
**8b**	0.598	-0.355	0.083	-1.377	0.524	2.88	1.089	Yes	No	CYP3A4
**8c**	0.574	-0.418	0.073	-1.346	-0.048	2.835	1.247	Yes	No	CYP3A4
**8d**	0.046	-0.696	0.079	-1.081	0.55	3.015	1.218	Yes	No	CYP3A4
**8e**	0.478	-0.653	0.104	-1.077	0.553	2.996	1.106	Yes	No	CYP3A4
**11e**	1.13	0.543	0.158	-1.039	0.596	2.795	1.275	Yes	No	CYP3A4
**11f**	1.224	0.53	0.167	-1.223	0.591	2.805	1.209	Yes	No	CYP3A4
**11a**	0.61	0.317	0.482	-1.035	0.591	2.654	1.232	Yes	Yes	CYP3A4
**11b**	0.661	0.383	0.462	-1.086	0.637	2.65	1.094	Yes	Yes	CYP3A4
**11c**	0.772	0.467	0.417	-1.085	0.674	2.603	1.144	Yes	No	CYP3A4
**13a**	0.646	0.297	0.172	-1.188	0.594	2.809	1.229	Yes	No	CYP3A4
**13b**	0.549	0.282	0.179	-1.395	0.562	2.814	1.144	Yes	No	CYP3A4
**13c**	0.554	0.333	0.196	-1.221	0.593	2.814	1.113	Yes	No	CYP3A4
**13d**	0.026	0.105	0.134	-1.411	0.367	2.732	1.83	Yes	No	CYP3A4
**11d**	1.086	0.593	0.314	-1.186	0.755	2.557	1.15	Yes	No	CYP3A4
**16c**	0.654	-0.062	0.316	-1.117	0.405	2.292	2.671	Yes	No	CYP3A4
**16e**	0.595	-0.028	0.304	-0.909	0.467	2.297	2.62	Yes	No	CYP3A4
**16f**	0.619	0	0.31	-2.993	0.48	2.254	2.66	Yes	No	CYP3A4
**25**	0.524	-.354	0.103	-1.348	0.2	2.389	2.062	Yes	No	CYP2D6<br>CYP3A4
**26**	0.543	0.203	0.473	-1.009	0.683	2.453	1.072	Yes	Yes	No predicted
**17a**	0.13	0.117	0.162	-1.286	0.689	2.805	0.918	Yes	No	CYP3A4
**17b**	0.634	0.136	0.168	-1.492	0.577	2.822	0.833	Yes	No	CYP3A4
**17c**	0.145	0.123	0.202	-1.51	0.603	2.84	1.541	Yes	No	CYP3A4
**17d**	0.631	0.124	0.171	-1.471	0.618	2.819	0.849	Yes	No	CYP3A4
**17e**	0.639	0.187	0.181	-1.318	0.665	2.822	0.803	Yes	No	CYP3A4
**17f**	0.155	0.157	0.189	-1.501	0.688	2.85	1.542	Yes	No	CYP3A4
**20a**	1.117	0.663	0.439	-1.253	0.742	2.572	0.912	Yes	Yes	CYP3A4
**20b**	0.719	0.418	0.508	-1.35	0.766	2.655	1.507	Yes	Yes	CYP3A4
**23a**	0.178	-0.227	0.447	-1.536	0.802	2.805	1.43	Yes	Yes	CYP3A4
**23b**	-.014	-0.111	0.178	-1.484	0.695	2.741	1.602	Yes	No	CYP3A4

Text S4: ADME prediction by SwissADME

Further investigation of ADME properties was done for the most promising compounds using SwissADME[25]. The predicted values are shown in **Table S2**. All of the studied compounds showed high GI absorption values, indicating good gastrointestinal absorption. The values were similar to norfloxacin, which is indeed marketed for oral administration. SwissADME also predicted good bioavailability scores, matching MOE-predicted physicochemical properties and Lipinski's rule of five (see above). The topological polar surface are (TPSA) is a value linked to drug bioavailability, a TPSA equal to or less than 140 $Å^2$ indicates a good oral bioavailability in rats[11]. TPSA values for most of the tested compounds ranged from 65.78 to 140.70 $Å^2$, suggesting good oral absorption. Most of the compounds also showed aqueous solubility values between -2.39 and -5.27, indicating good to moderate solubility in water. Lipophilicity was assessed using the logarithm of the n-octanol/water partition coefficient, which was predicted using the Consensus LogPo/w descriptor of SwissADME. LogPo/w is closely related to transport processes, including membrane permeability and penetration, which directly affects ability of the drug to reach its target site[26]. Most of the tested compounds had LogPo/w values ranging from 0.73 to 3.07, predicting good permeability and tissue penetration according to the general guide for good oral bioavailability (logP (0 < logP< 3))[27]. All investigated compounds show a good bioavailability score of 0.55.

Table S3: Physicochemical and pharmacokinetic properties predicted by SwissADME.

Compound	Consensus Log $P_{o/w}$	TPSA (Å²)	Log S (ESOL)	Water solubility class	GI absorption	BBB permeant	Bioavailability Score
**Nor**	0.98	74.57	-1.29	Very soluble	High	No	0.55
**10f**	3.07	65.78	-4.15	Moderately soluble	High	No	0.55
**5a**	0.89	94.88	-2.39	Soluble	High	No	0.55
**11e**	2.63	77.81	-4.54	Moderately soluble	High	Yes	0.55
**11f**	2.87	77.81	-5.14	Moderately soluble	High	Yes	0.55
**11a**	1.25	77.81	-3.17	Soluble	High	No	0.55
**11c**	1.88	77.81	-3.58	Soluble	High	No	0.55
**13a**	2.19	94.88	-4.57	Moderately soluble	High	No	0.55
**13d**	1.47	140.70	-4.65	Moderately soluble	High	No	0.55
**16c**	1.92	103.16	-3.64	Soluble	High	No	0.55
**16e**	1.98	103.16	-3.77	Soluble	High	No	0.55
**25**	1.69	123.63	-3.82	Soluble	High	No	0.55
**17a**	1.65	115.19	-4.46	Moderately soluble	High	No	0.55
**17b**	2.06	115.19	-5.27	Moderately soluble	High	No	0.55
**17d**	1.97	115.19	-4.95	Moderately soluble	High	No	0.55
**20b**	1.18	90.28	-3.36	Soluble	High	No	0.55
**23a**	0.73	115.19	-2.96	Soluble	High	No	0.55

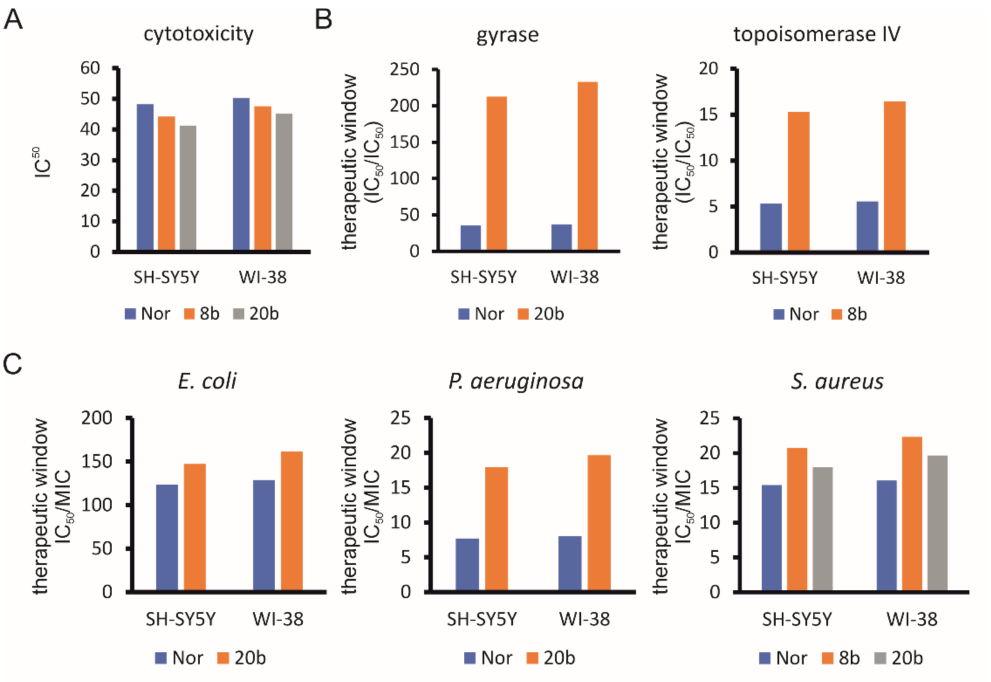

Figure S93: Cytotoxicity and therapeutic windows of compounds **8b** and **20b**. (A) Cytotoxicity against SH-Sy5y (human neuroblastoma) and WI38 (human fetal lung fibroblast) cells. Therapeutic windows were estimated by the ratio of the IC_{50} values of cytotoxicity and (B) IC_{50} values of gyrase and topoisomerase inhibition and (C) MIC values against *E. coli* W3110, *P. aeruginosa* PAO1, and *S. aureus* CCUG1800T.

4. Molecular Modeling

Text S5: Docking on *S. aureus* DNA gyrase

Docking studies of the most active compounds from each series were performed against *S. aureus* DNA gyrase based on the crystal structure of the enzyme in complex with moxifloxacin and DNA (PDB code 5cdq)[28]. The binding patterns and interaction modes of the designed molecules at the active site were then compared to that of moxifloxacin and norfloxacin. The docking protocol was validated by re-docking of the co-crystalized moxifloxacin at the active site of DNA gyrase (PDB ID: 5cdq) (**Figure S94**, re-docking rmsd = 0.6010 Å, binding score = -10.76 kcal mol^{-1}). The validated docking setup was then used to investigate the ligand-receptor interactions for norfloxacin (**Figure S95**, score = -9.54 kcal mol^{-1}). The main interactions of norfloxacin were two coordination bonds (2.44 and 2.34 Å) with the Mg^{2+} metal ion in the active center, H-bonding between the carbonyl of the carboxylic acid group and Ser B84 (2.26 Å), a π-hydrogen bond with DA E2013, and π-π stacking between the quinolone ring and DG D2009.

Compounds **8b**, **25**, **17a**, and **17b** were selected for docking on the target enzymes (**Figure S96-99**) as they had the highest activity against *S. aureus.* The binding modes of compounds **8b** and **17d** showed the lowest binding scores (-9.80 and -10.11 kcal mol^{-1}, respectively). In compounds **8b**, and **17d**, the oxygens of both the quinolone and hydroxamic carbonyl groups formed two coordination bonds with Mg^{2+} (2.45 and 2.53 Å, respectively). H-bonding between the hydroxamic carbonyl group with Ser B84 residue had an average distance of 2.23 Å. Interactions with nitrogenous bases DG D2009 and DA E2013 were mediated by π-π stacking and π-H bond with the quinolone and piperazine ring, respectively. The newly designed compounds also interacted with other amino acids as extra binding interactions were formed by the added structural moieties at the *N*-4 of piperazine ring of norfloxacin through H-bonding and π-cation, such as two H-bonds that formed between the 2' and 3' carbonyl groups of the isatin moiety of compound **17d** and Arg C458 and Lys C417 (2.25 and 2.45 Å, respectively). These non-covalent interactions might stabilize the **8b/17d**-enzyme-DNA complex, which may underlie the low binding scores and the good inhibitory activities of compounds (**Table S4**).

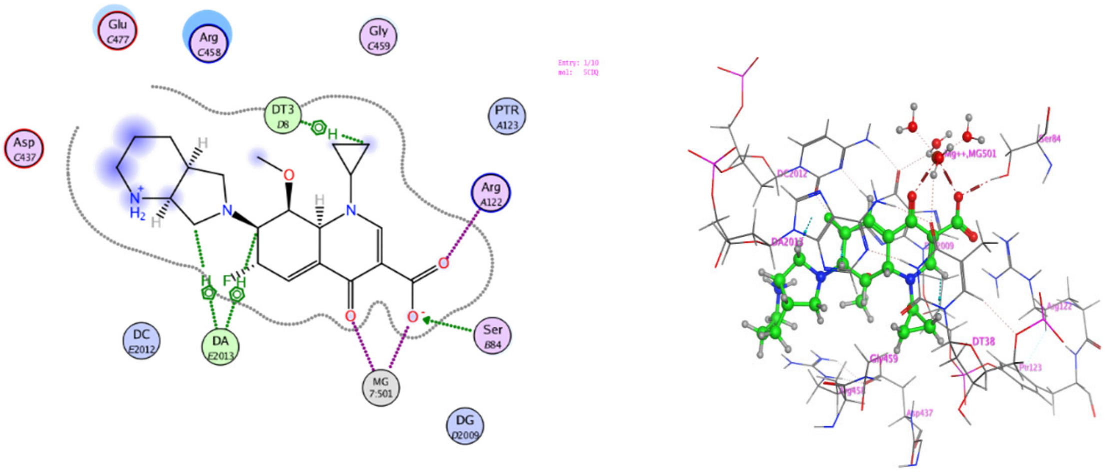

Figure S94: 2D and 3D interactions of co-crystallized ligand moxifloxacin with *S. aureus* gyrase.

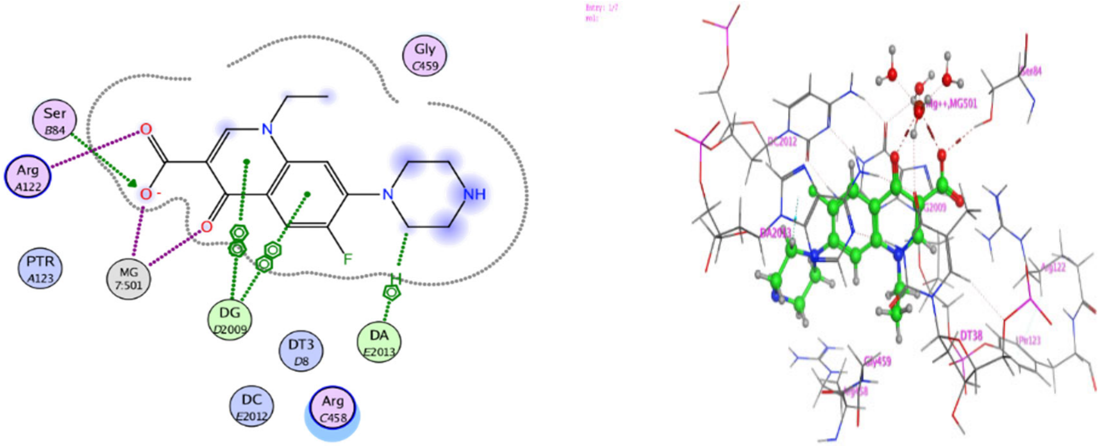

Figure S95: 2D and 3D interactions of norfloxacin with *S. aureus* gyrase.

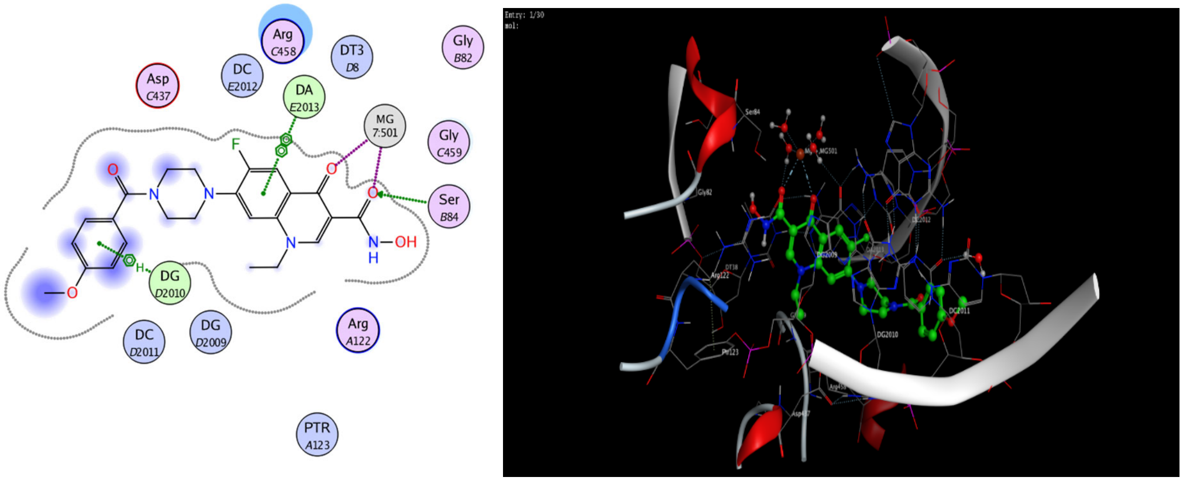

Figure S96: 2D and 3D interactions of compound **8b** with *S. aureus* gyrase.

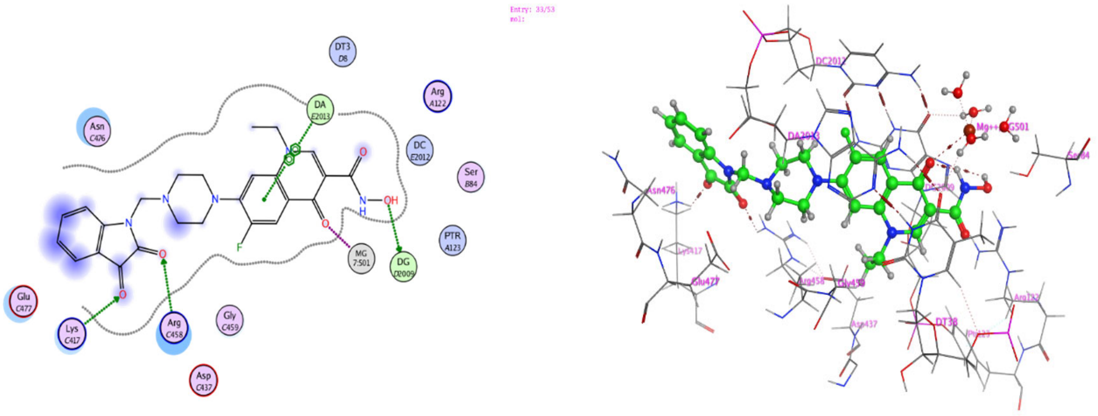

Figure S97: 2D and 3D interactions of compound **17a** with *S. aureus* gyrase.

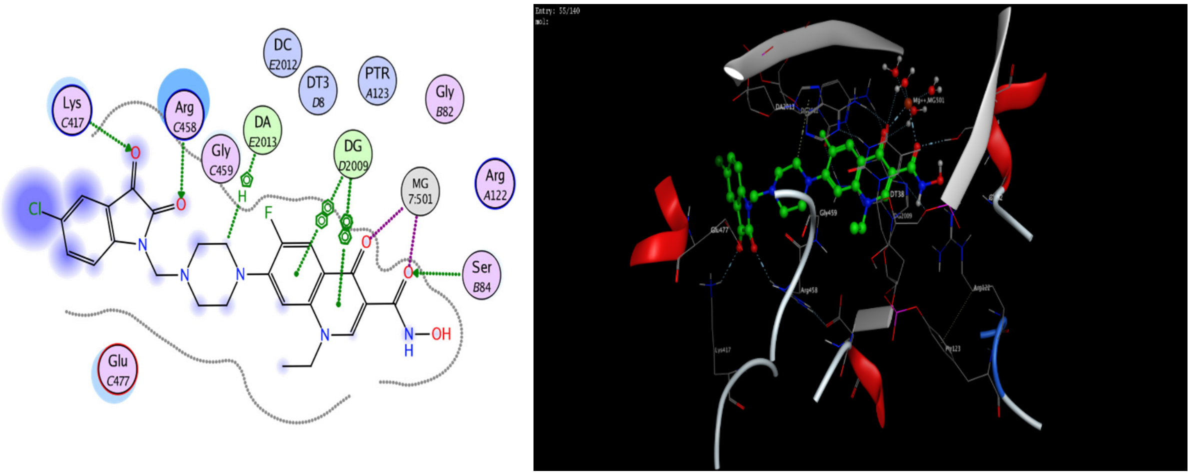

Figure S98: 2D and 3D interactions of compound **17d** with *S. aureus* gyrase.

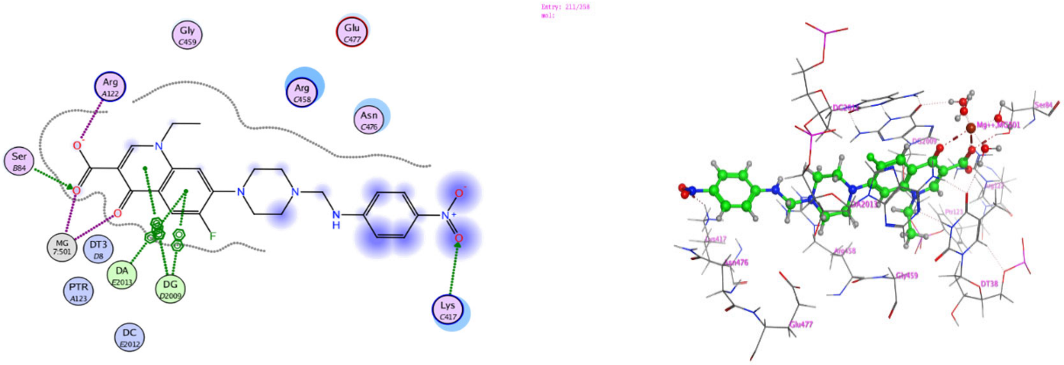

Figure S99: 2D and 3D interactions of compound **25** with *S. aureus* gyrase.

Table S4: Binding energy scores with *S. aureus* DNA gyrase.

Compound	Binding score ΔG (kcal mol^{-1})	Amino acids involved in interaction	MIC on *S. aureus* (μM)
**8b**	-9.80	Arg A122, Ser B84, Gly B117, Glu B88, Glu C477, Asn A269, Lys A276, Lys C417, DG D2009, DA E2013, DC D2012, DC C2012, Mg^{2+}	**2.13**
**25**	-9.71	Arg A122, Ser B84, Asn A269, Glu A88, Arg B272, Lys C417, DA E2013, DG D2009, DG B3, Mg^{2+}	**2.13**
**17a**	-10.03	Arg A122, Ser B84, Gly B117, Glu B88, Lys C417, Asn A269, Arg C58, DG D2009, DA E2013, DC D2012, DC C2012, Mg^{2+}	**2.02**
**17d**	-10.11	Arg A122, Ser B84, Gly B117, Glu B88, Asp A83, Asn A269, Arg C458, Lys C417, DG D2009, DA E2013, DC D2012, DC C2012, Mg^{2+}	**1.89**

Text S6: Docking on *A. baumannii* topoisomerase IV

Docking the most active compounds from each series was performed based on the crystal structure of *A. baumannii* topoisomerase IV in complex with moxifloxain and DNA (PDB code 2xkk)[29]. The docking protocol was validated by re-docking of the co-crystalized moxifloxacin at the active site (**Figure S100**, re-docking rmsd = 0.3718 Å, binding score = -10.62 kcal mol^{-1}). The validated docking setup was then used to investigate the ligand-receptor interactions for norfloxacin (**Figure S101**, score = -9.32 kcal mol^{-1}). The amino acid residues and nitrogenous bases involved in interactions with co-crystallized moxifloxacin at the active site were Arg A1123, DA D16, DT C19, DA C20, and Mg^{2+} [30]. The main types of interactions were coordination bonds with Mg^{2+} through the oxygen of the ketonic carbonyl and the oxygen of the carboxylic carbonyl groups (1.91 and 1.99 Å, respectively), H-bonding of the oxygen of the carboxylic carbonyl group with Arg A1123 (3.59 Å), and π-π stacking between the quinolone ring and DA D16, DT C19, and DA C20. For norfloxacin, the main interactions were a coordination bond with Mg^{2+} (2.41 Å), H-bonding between the carbonyl of the carboxylic acid group and Arg1123 (3.45 Å), a π-hydrogen bond with DA C20, and π-π stacking between the quinolone ring and DA D16.

Compounds **11a**, **11d**, **11f**, **19a**, **25**, **17b**, **20b**, and **23a** were selected for docking studies (**Figure S102-109**) as they had the highest activity against *E. coli.* The binding modes of compounds **11a**, **11f**, **25**, and **20b** showed the lowest binding scores (-10.44, -10.78, -11.89, and -11.74 kcal mol^{-1}, respectively). The oxygens of both quinolone and the hydroxamic carbonyl groups formed two coordination bonds with Mg^{2+} (2.41 and 2.45 Å, respectively). The oxygen of hydroxamic carbonyl formed a H-bond with Arg A1123 (1.96 Å). π-π Stacking and π-hydrogen interactions were mediated by the quinolone ring with DA D16 and DA C20, respectively. The new compounds also interacted with other amino acids as extra binding interactions were mediated by the hydroxamic acid group and the added structural moieties at the *N*-4 of piperazine ring of norfloxacin through H-bonds and π-cation bonds, such as two H-bonds that formed between the NH and OH groups of the hydroxamic moiety of compound **11a** with Glu B437 and Asp B440, respectively**.** Moreover, the morpholine and succinimide moieties of compounds **20b** and **23a** formed a π-H bond with the DT D15 nitrogenous base and one of the carbonyl groups of **23a** formed a H-bond with Asp A1083. The NH and phenyl ring of the p-nitrophenyl amino moiety of compound **25** formed a H-bond and π-H bond with Glu B437 and Gln B436, respectively. These

non-covalent interactions may stabilize the compounds-enzyme-DNA complex, explaining their low binding scores and the good inhibitory activity (**Table S5**).

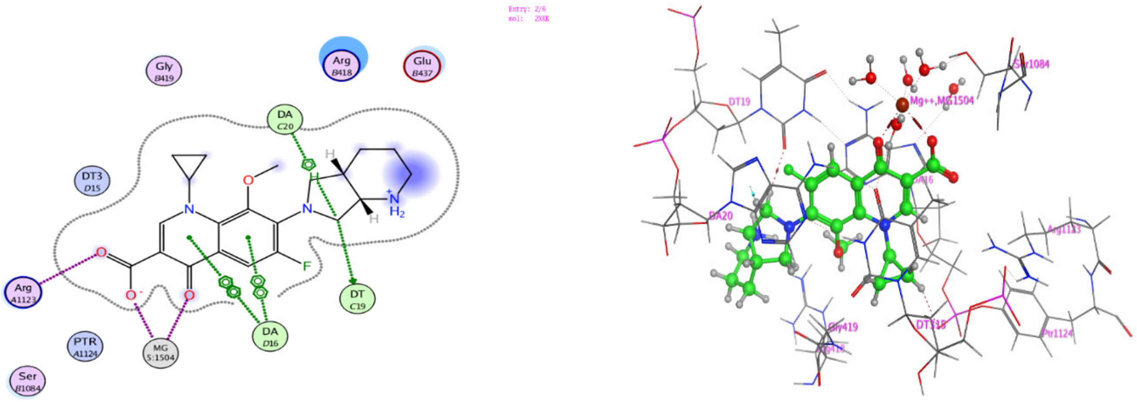

Figure S100: 2D and 3D interactions of co-crystallized moxifloxacin with *A. baumannii* topoisomerase IV.

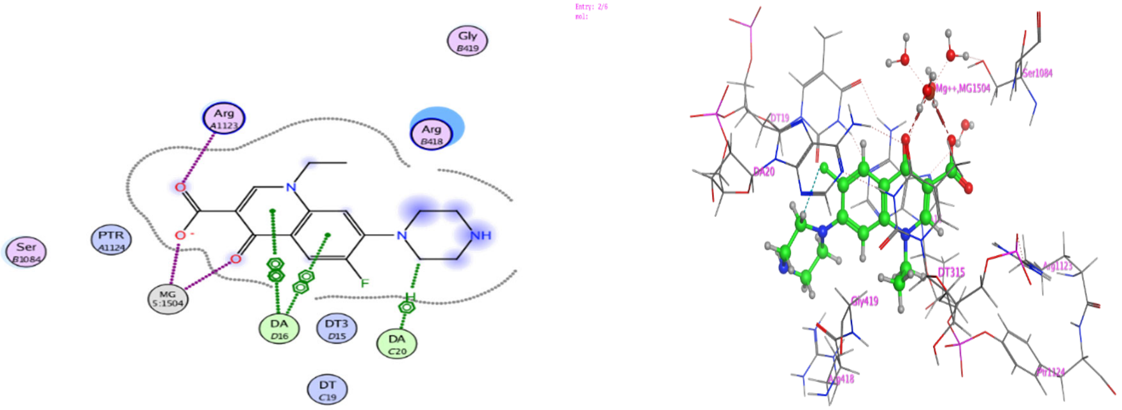

Figure S101: 2D and 3D interactions of norfloxacin with *A. baumannii* topoisomerase IV.

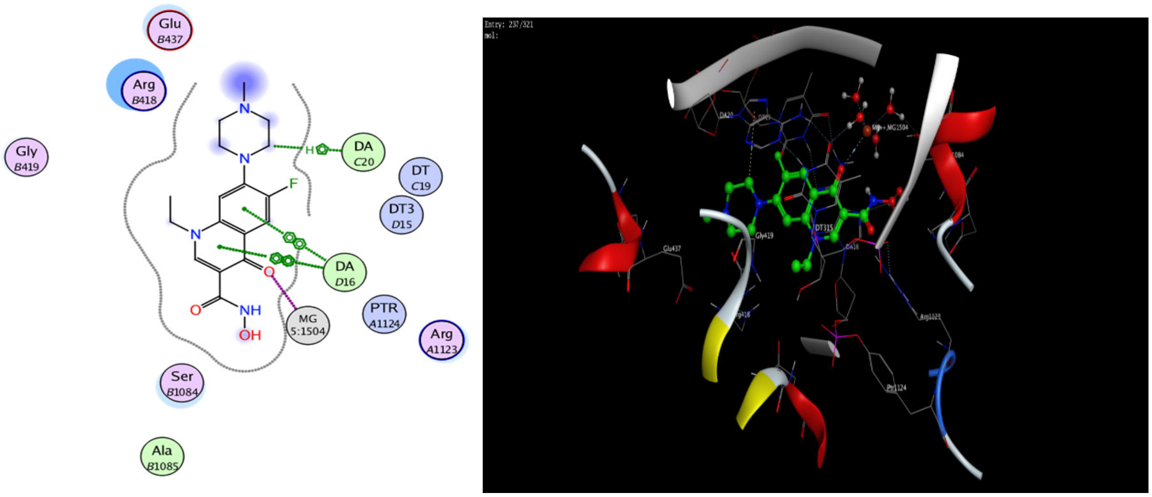

Figure S102: 2D and 3D interactions of compound **11a** with *A. baumannii* topoisomerase IV.

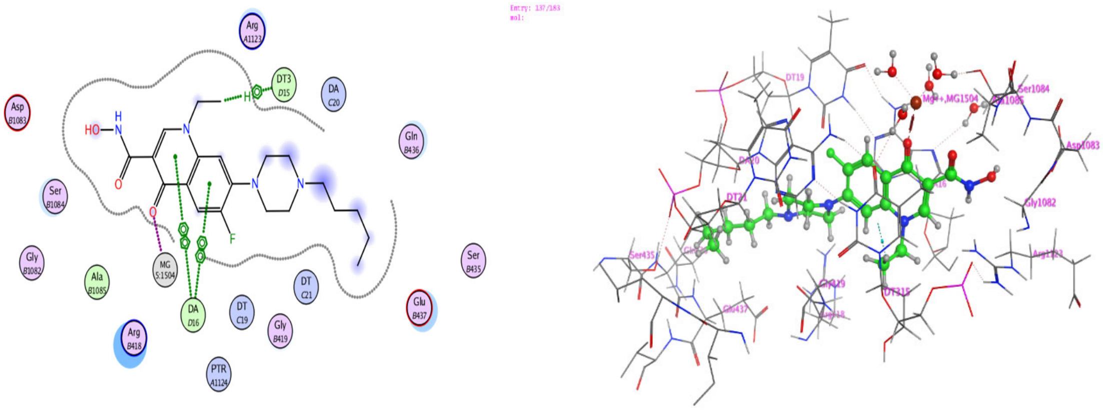

Figure S103: 2D and 3D interactions of compound **11d** with *A. baumannii* topoisomerase IV.

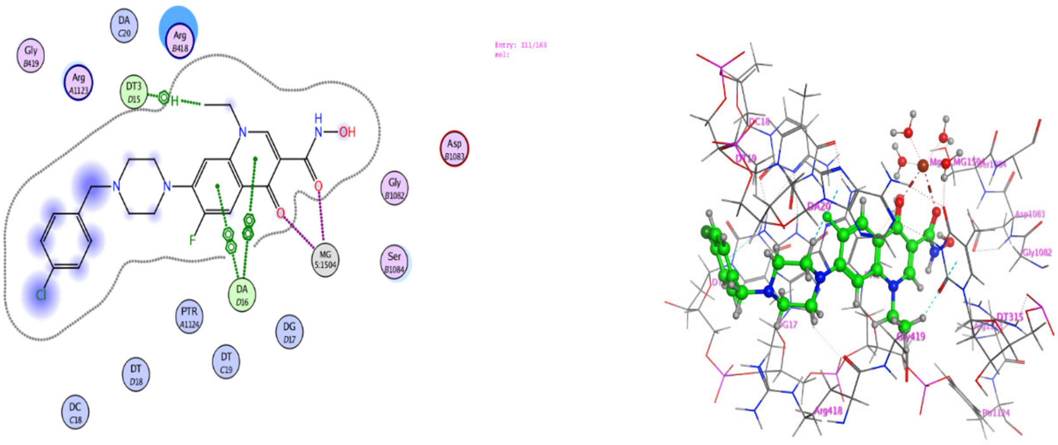

Figure S104: 2D and 3D interactions of compound **11f** with *A. baumannii* topoisomerase IV.

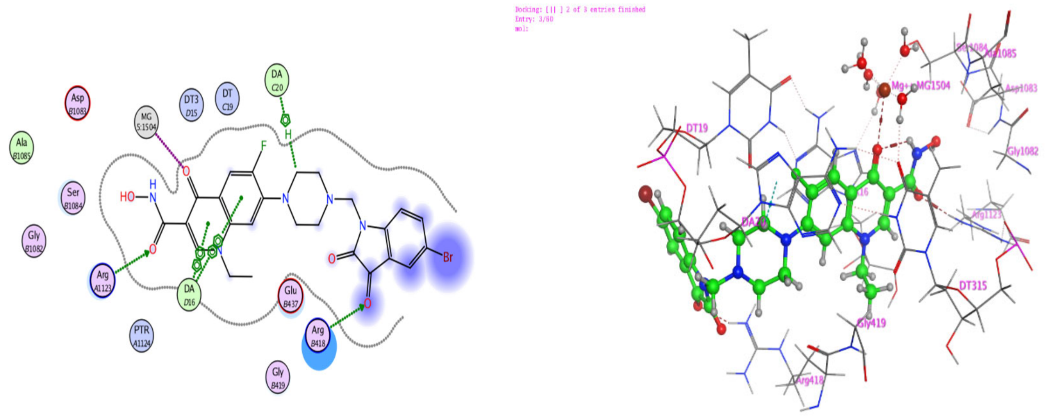

Figure S105: 2D and 3D interactions of compound **17b** with *A. baumannii* topoisomerase IV.

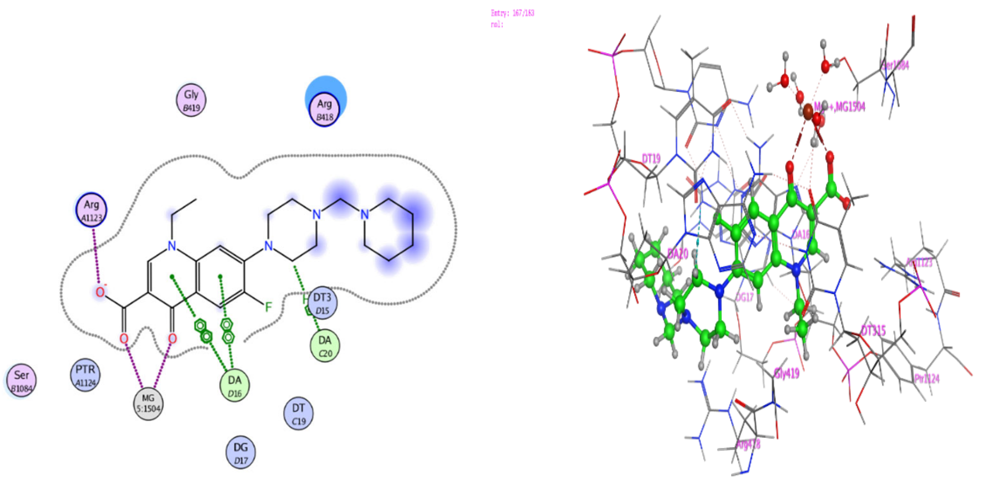

Figure S106: 2D and 3D interactions of compound **19a** with *A. baumannii* topoisomerase IV.

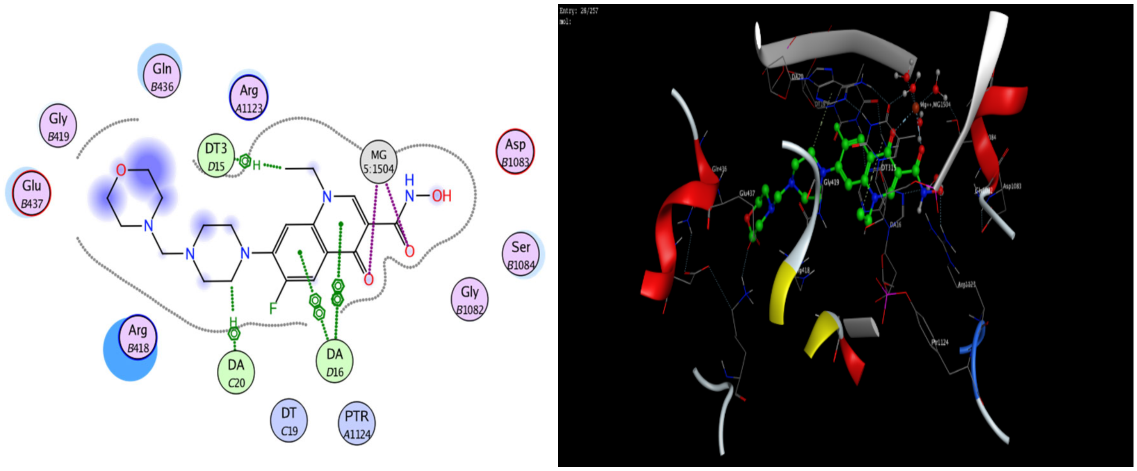

Figure S107: 2D and 3D interactions of compound **20b** with *A. baumannii* topoisomerase IV.

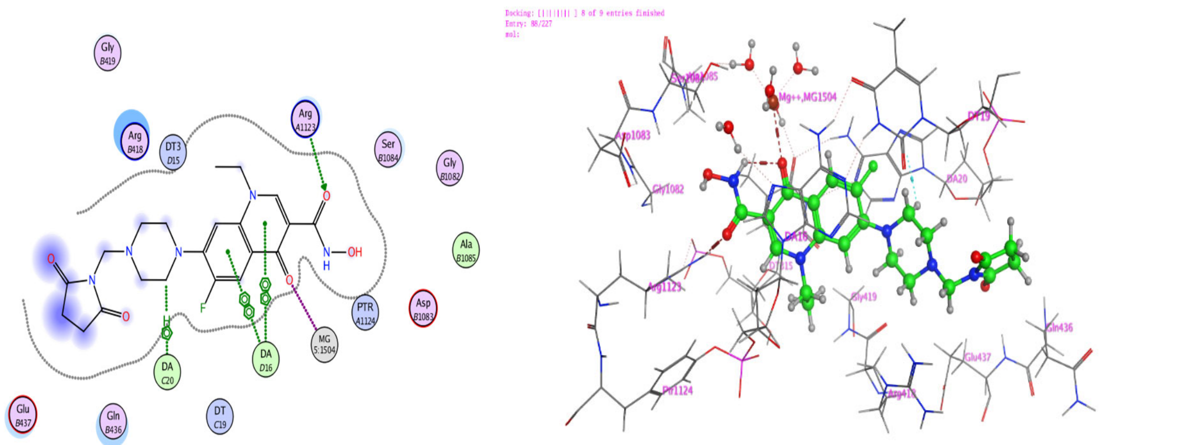

Figure S108: 2D and 3D interactions of compound **23a** with *A. baumannii* topoisomerase IV.

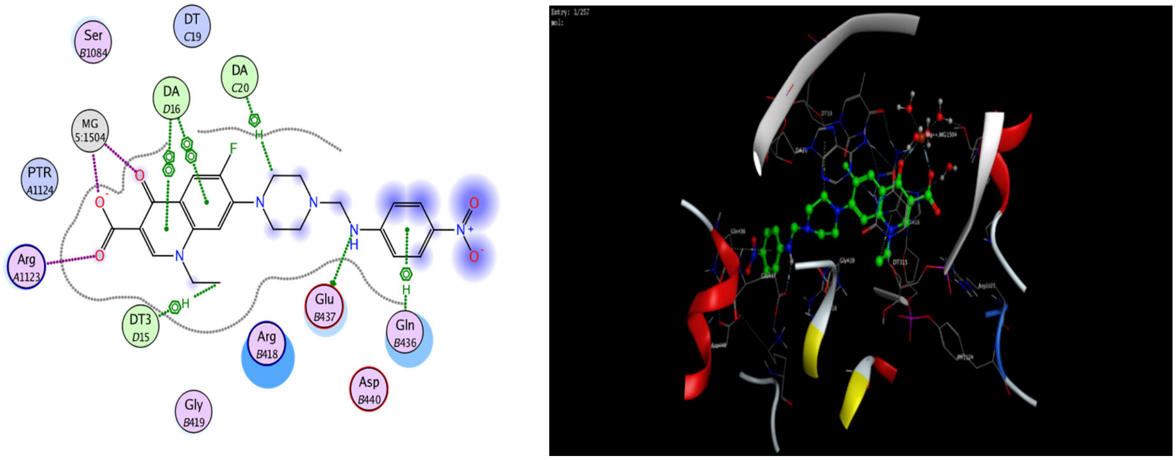

Figure S109: 2D and 3D interactions of compound **25** with *A. baumannii* topoisomerase IV.

Table S5: Binding scores with *A. baumannii* topoisomerase IV.

Compound	Binding score ΔG (kcal mol^{-1})	Amino acids involved in interaction	MIC on *E. coli* (μM)
**11a**	-10.44	Arg A1123, Asp A440, Arg B418, Glu A437, DA C20, DG C16, DT3 C15, Mg^{2+}	**0.18**
**11d**	-10.23	Arg A1123, Asp A1083, Arg B418, Glu B437, DA C20, DG C16, DT3 C15, Mg^{2+}	**0.3**
**11f**	-10.78	Arg A1123, Asp A1083, Arg B418 DA C20, DG C16, DT3 C15, Mg^{2+}	**1.08**
**19a**	-11.48	Arg A1123, Asp A1083, Arg B418 DA C20, DA D16, DT3 C15, Mg^{2+}	**0.3**
**25**	-11.89	Arg A1123, Asp A1083, Arg B418, Glu B437, Gln B436, DA C20, DA D16, DT3 D15, Mg^{2+}	**0.266**
**17b**	-11.23	Arg A1123, Asp A1083, Arg B418, Glu B1088, Ser B1118, Lys B377, DA C20, DA D16, DT3 D15, Mg^{2+}	**2.62**
**20b**	-11.74	Arg A1123, Asp A1083, Arg B418, Glu B437, Asp A440, DA C20, DA D16, DT3 D15, Mg^{2+}	**0.28**
**23a**	-11.45	Arg A1123, Asp A1083, Arg B418, Glu B437, DA C20, DA D16, DT3 D15, Mg^{2+}	**0.56**

Text 7: Docking on *M. smegmatis* NagA

Docking of the derivatives with the highest activity against *B. subtilis* was performed based on the crystal structure of *Mycobacterium smegmatis N*-acetyl-D-glucosamine-6-phosphate deacetylase (D267A mutant) in complex with *N*-acetyl-D-glucosamine-6-phosphate (PDB code 6fv4)[31]. The binding patterns and interaction modes of the designed molecules were then compared to that of *N*-acetyl-D-glucosamine-6-phosphate and norfloxacin at its active site. The docking protocol was validated by re-docking of co-crystalized *N*-acetyl-D-glucosamine-6-phosphate at the active site (**Figure S110**, re-docking rmsd = 1.5535 Å, binding score = -12.63 kcal mol-1). The validated docking setup was then used to investigate the ligand-receptor interactions of norfloxacin (score = -8.40 kcal mol^{-1}) (**Figure S111).** Docking of norfloxacin showed that the oxygen of the carboxylic acid group formed a coordination bond with Cd^{2+} (1.75 Å) and two H-bonds with Gly132 and Ala133 that were mediated by the ketonic carbonyl group (2.29 Å).

Compounds **11a**, **11c**, **11e**, **11f**, **16b**, **16c**, **25**, **20b**, and **43a** were selected for docking experiments (**Figure S112-120**) as they had the highest activity against *B. subtilis.* The binding modes of compounds **11e**, **11f**, **25**, and **20b** showed the lowest binding scores (-13.57, -14.61, -16.64, and -14.35 kcal mol^{-1}, respectively). All tested compounds interacted with the same amino acids as the co-crystallized ligand and norfloxacin. The common interactions in all docked compounds include a coordination bond with both Cd^{2+} and Zn^{2+} (2.43 and 2.56 Å, respectively), which was mediated by the carboxylate and hydroxamic acid groups and was shorter than that formed by the co-crystallized ligand, which only formed a coordination bond with Cd^{2+} (2.69 Å). Furthermore, three H-bonds with Gly132, Ala133, and His134 were formed by quinolone, the carboxylate, or hydroxamic carbonyl groups with average lengths of 2.26, 1.93, and 2.21 Å, respectively. Compounds **11e, 11f** and **20b** formed a H-bond with Ala302 through the hydroxamic NH and OH groups (2.30 Å). The compounds also interacted with other amino acids mediated by hydroxamic acid modifications at the carboxylic group and by the added structural moieties at the *N*-4 of piperazine ring through H-bonds and π-cation bonds, such as a H-bond between the nitro group of the p-nitroaniline moiety or oxygen atom of the morpholine ring of compounds **25** and **20b** with Ala246, respectively. Moreover, compound **20b** formed a π-H bond with Thr300 and Ala213 mediated by the quinolone core. These interactions explain the lower docking scores of new compounds compared to norfloxacin and could play a role in their higher activity (**Table S6**).

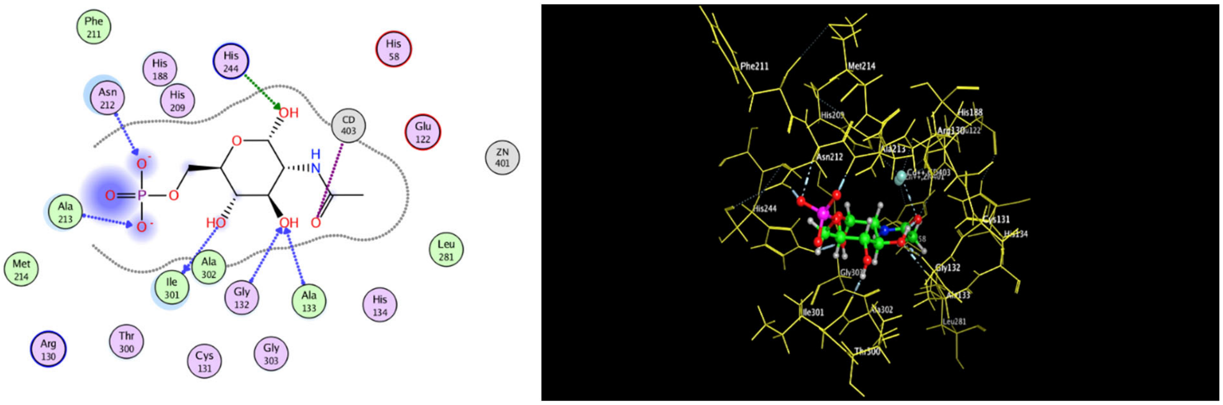

Figure S110: 2D and 3D interactions of co-crystallized ligand *N*-acetyl-D-glucosamine-6-phosphate with *M. smegmatis* NagA.

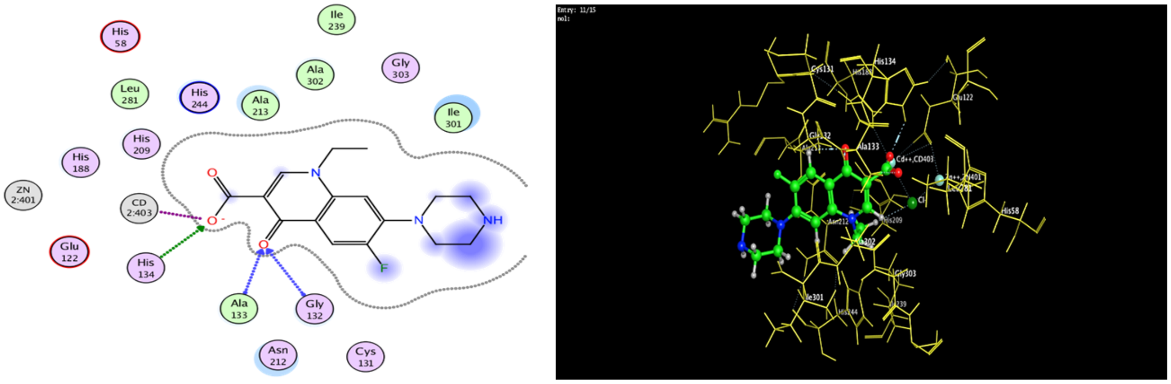

Figure S111: 2D and 3D interactions of norfloxacin with *M. smegmatis* NagA.

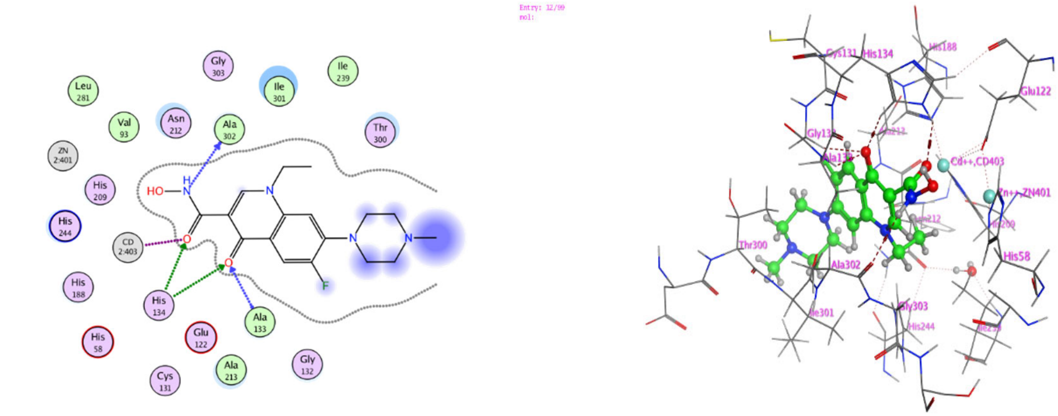

Figure S112: 2D and 3D interactions of compound **11a** with *M. smegmatis* NagA.

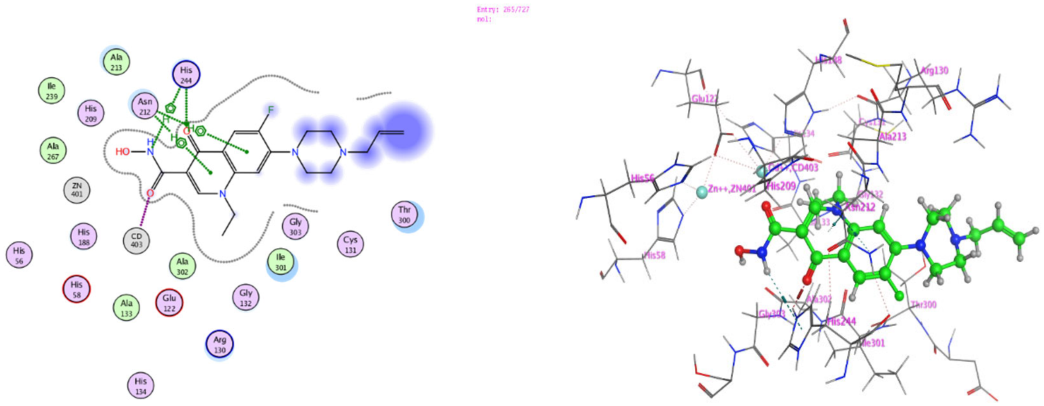

Figure S113: 2D and 3D interactions of compound **11c** with *M. smegmatis* NagA.

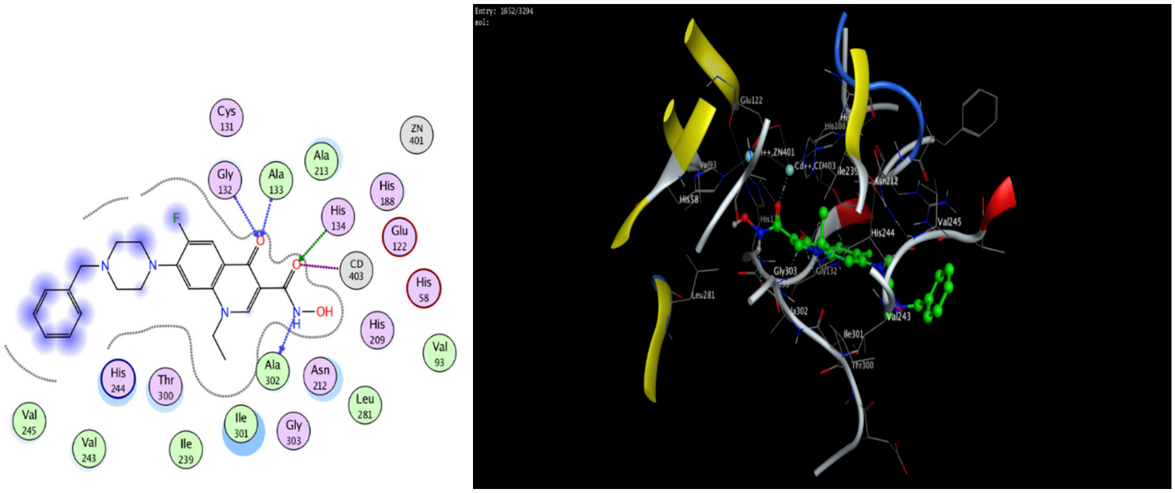

Figure S114: 2D and 3D interactions of compound **11e** with *M. smegmatis* NagA.

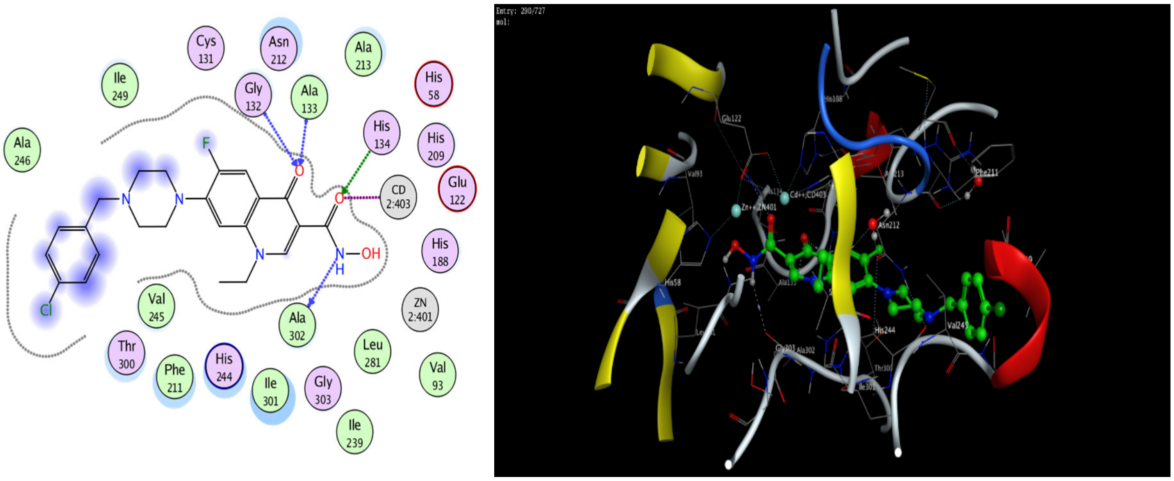

Figure S115: 2D and 3D interactions of compound **11f** with *M. smegmatis* NagA.

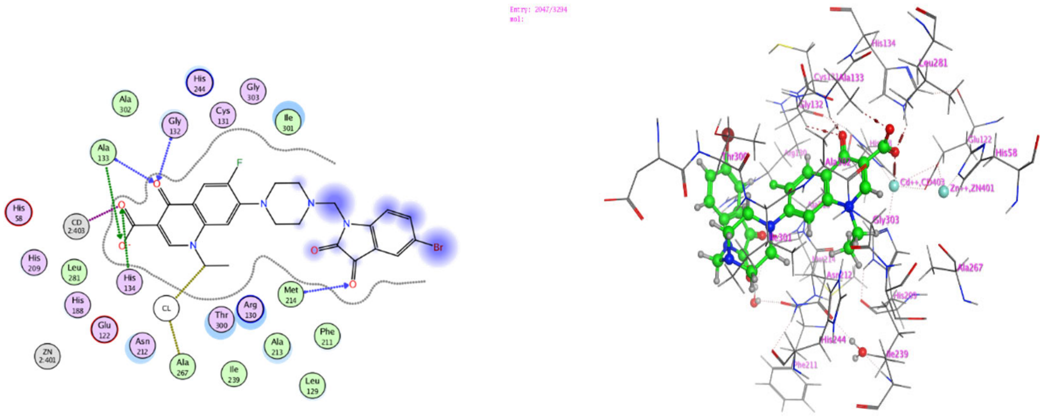

Figure S116: 2D and 3D interactions of compound **16b** with *M. smegmatis* NagA.

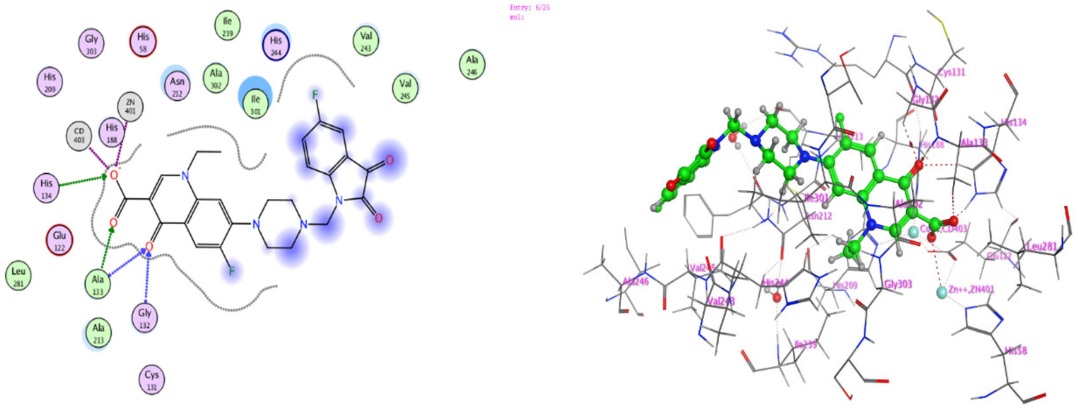

Figure S117: 2D and 3D interactions of compound **16c** with *M. smegmatis* NagA.

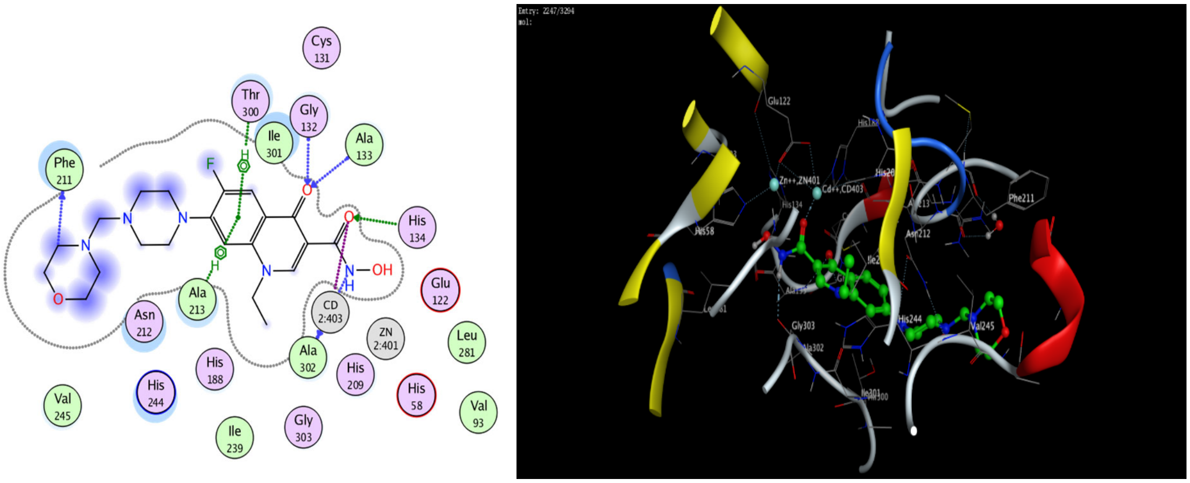

Figure S118: 2D and 3D interactions of compound **20b** with *M. smegmatis* NagA.

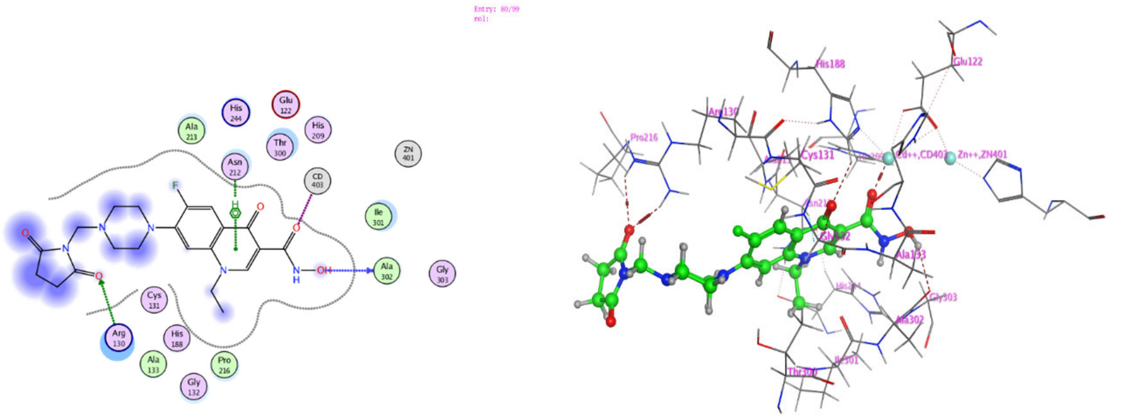

Figure S119: 2D and 3D interactions of compound **23a** with *M. smegmatis* NagA.

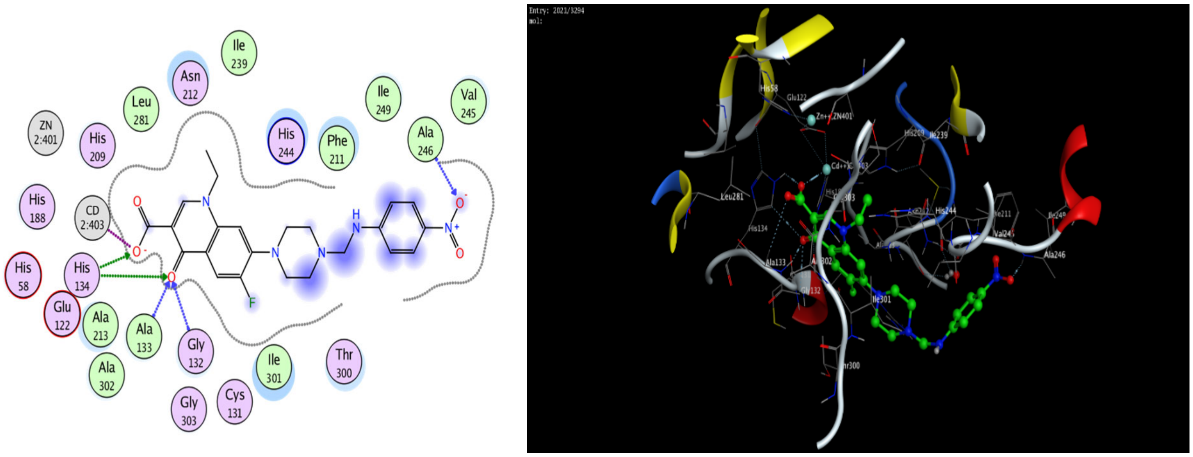

Figure S120: 2D and 3D interactions of compound **25** with *M. smegmatis* NagA.

Table S6: Binding energy scores with *M. smegmatis* NagA.

Compound	Binding score ΔG (kcal mol^{-1})	Amino acids involved in interaction	MIC on *B. subtilis* (µM)
**11a**	-12.29	Gly132, Ala133, His134, Arg130, His244, Asn212, Ala213, Ala302, Cd^{2+}	**2.87**
**11c**	-13.38	Gly132, Ala133, His134, Arg130, His244, Asn212, Ala213, Ala302, Cd^{2+}	**1.33**
**11e**	-13.57	Gly132, Ala133, His134, Arg130, His244, Asn212, Ala213, Ala302, Arg130, Cd^{2+}	**1.17**
**11f**	-14.61	Gly132, Ala133, His134, Arg130, His244, Ala213, Asn212, Cd^{2+}	**1.08**
**16b**	-13. 63	Gly132, Ala133, His134, Arg130, His244, Met214, Ala213, Ala267, Asn212, Zn^{2+}, Cd^{2+}	**7.17**
**16c**	-13. 86	Gly132, Ala133, His134, Arg130, His244, Asp299, Ala213, Ala264, Asn212, Zn^{2+}, Cd^{2+}	**16.11**
**25**	-16.64	Gly132, Ala133, His134, Arg130, His244, Asp299, Ala213, Ala264, Asn212, Cd^{2+}	**2.13**
**20b**	-14.35	Gly132, Ala133, His134, Arg130, His244, Asn212, Ala302, Leu129, Ala213, Zn^{2+}, Cd^{2+}	**6.92**
**23a**	-13.41	Gly132, Ala133, His134, Arg130, His244, Asn212, Ala302, Leu129, Ala213, Cd^{2+}, Zn^{2+}	**17.95**

Text S8: Docking on *P. aeruginosa* LpxC

Docking studies were performed based on the co-crystal structure of the *P. aeruginosa* LpxC-50432 complex (PDB code: 6mod)[32]. The binding patterns and interaction modes of the designed molecules was then compared to that of the co-crystallized ligand N-[(1S)-2-(hydroxyamino)-1-(3-methoxy-1,1-dioxo-1lambda~6~-thietan-3-yl)-2-oxoethyl]-4-(6-hydroxyhexa-1,3-diyn-1-yl) benzamide (JWV) and norfloxacin at its active site. The docking protocol was validated by re-docking of the co-crystalized JWV ligand at the active site of LpxC (**Figure S121**, re-docking rmsd = 0.4827 Å, binding score = -10.74 kcal mol^{-1}). The validated docking setup was then used to investigate the ligand-receptor interactions for norfloxacin (**Figure S122**, score = -6.21 kcal mol^{-1}). Studying the interaction between the LpxC enzyme and the co-crystallized ligand revealed that the amino acid residues involved in the binding are Thr190, Phe191, Lys238, Leu18, His78, Met62, Asp241, and the Mg^{2+} metal ion[33]. The main interactions were H-bonding between the hydroxyl group of the hydroxamic group with His78 (2.59 Å), a H-bind between the amino group of the hydroxamic group with Met62 (2.27 Å), H-bonding between the carbonyl group and Thr190 (1.73 Å), π-H bond between the phenyl ring with Leu18, and H-bonding between the oxygen of the sulphonyl group with Lys238 (1.92 Å). Additionally, a coordination bond between the carbonyl group and Mg^{2+} (2.16 Å) was observed. In case of norfloxacin, the carbonyl of the carboxylic acid formed a coordination bond with Mg^{2+} (2.73 Å), a H-bond between the oxygen of the carboxylic acid and Phe191 (2.51 Å), and a π-cation interaction between the phenyl ring of the quinolone and Lys238.

Compounds **11a**, **11b**, **11d**, **11f**, **19a**, **25**, **17b**, **20b**, and **23a** were selected for docking experiments (**Figures S123-131**) as they had the highest activity against *E. coli.* The binding modes of compounds **11b**, **25**, and **20b** showed the lowest binding scores (-8.94, -9.48, -9.58, and -9.94 kcal mol^{-1}, respectively). Compounds with carboxylic acid groups like compound **25** formed two coordination bonds with Mg^{2+} both oxygen atoms of the carboxylate group (2.12 and 2.08 Å) and a H-bond with Thr190 mediated by the oxygen of the carboxylate group (1.69 Å). In thw hydroxamic acid compounds **11a**, **11b**, **11f**, and **20b**, the oxygen of the hydroxamic carbonyl group formed a coordination bond with Mg^{2+} (2.47 Å) and a H-bond with Thr190 (1.97 Å). Furthermore, the NH of the hydroxamic group formed a H-bond with Met62 (2.45 Å). Besides these interactions, the new compounds interacted with other amino acids mediated by the hydroxamic acid group and the added structural moieties at the *N*-4 of piperazine ring through H-bonds and π-cation bonds,

such as H-bonds between the NH and OH of the hydroxamic group with His264 and Glu77 (1.66 and 2.29 Å, respectively). Compound **25** formed two H-bonds through its nitro group with Asp161 and Lys261, while compound **20b** formed a H-bond with Lys142 through the oxygen of the morpholine moiety. Moreover, one carbonyl group of the succinimide moiety of compound **23b** formed a H-bond with Arg195. These non-covalent interactions could explain the low binding scores and the good inhibitory activity of the new compounds (**Table S7**).

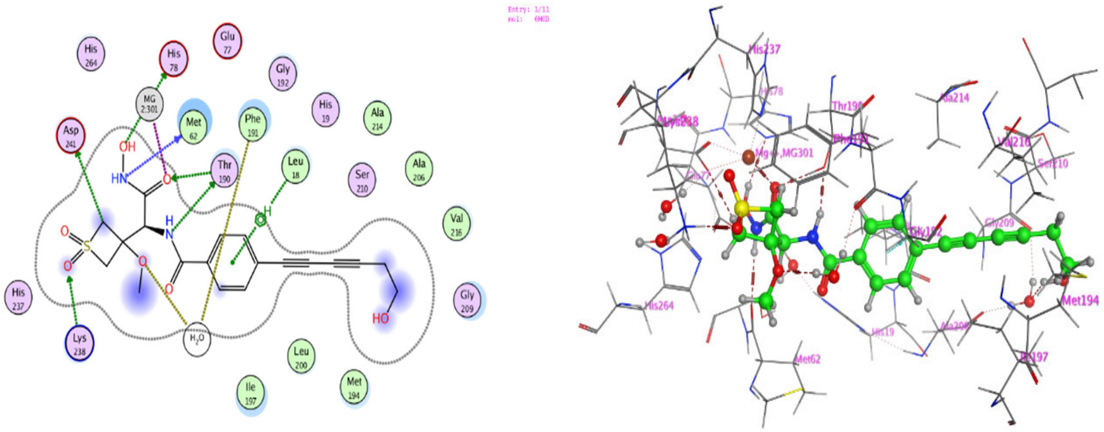

Figure S121: 2D and 3D interactions of co-crystallized ligand JWV with *P. aeruginosa* LpxC.

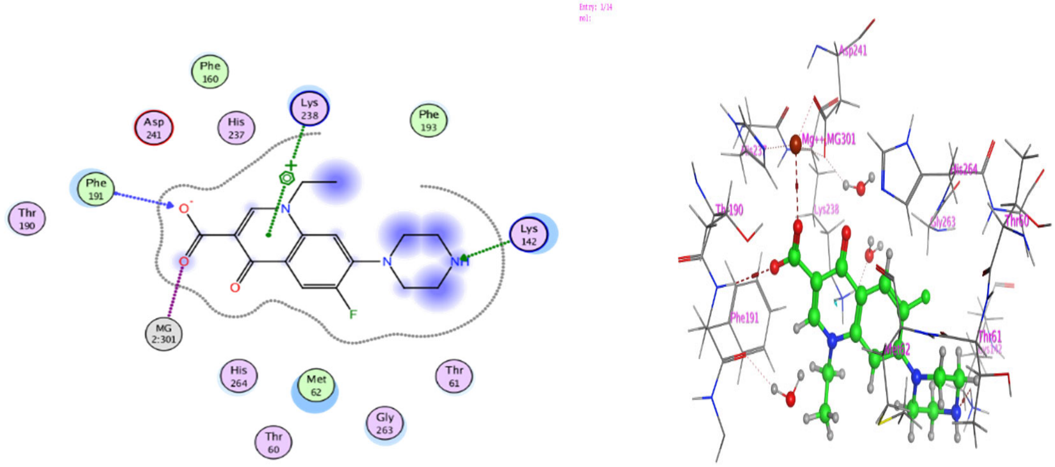

Figure S122: 2D and 3D interactions of norfloxacin with *P. aeruginosa* LpxC.

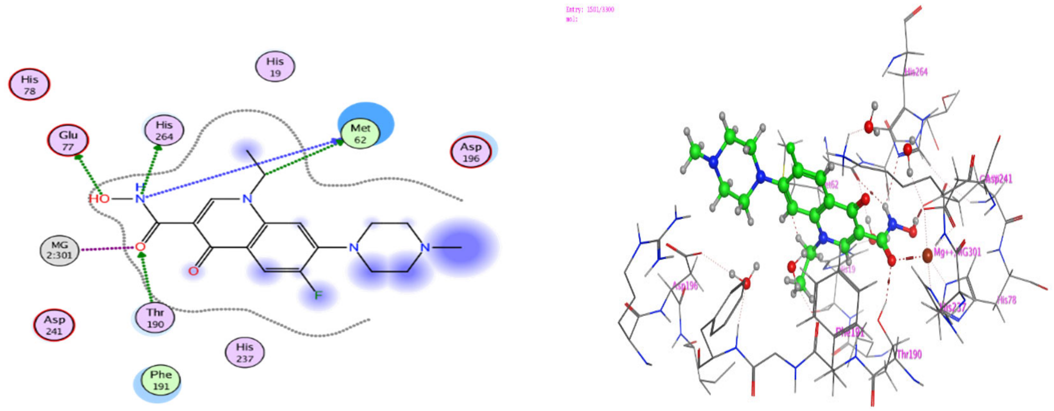

Figure S123: 2D and 3D interactions of compound **11a** with *P. aeruginosa* LpxC.

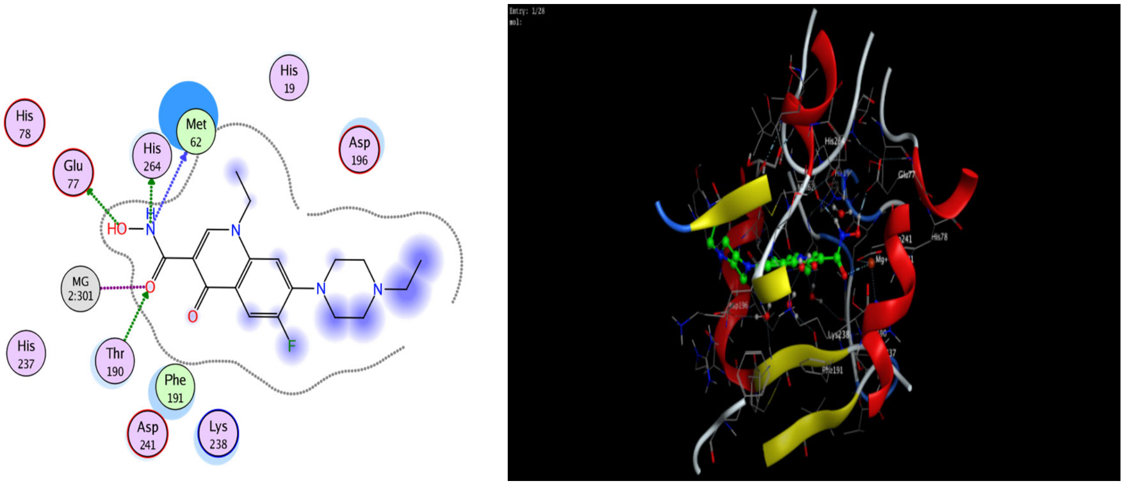

Figure S124: 2D and 3D interactions of compound **11b** with *P. aeruginosa* LpxC.

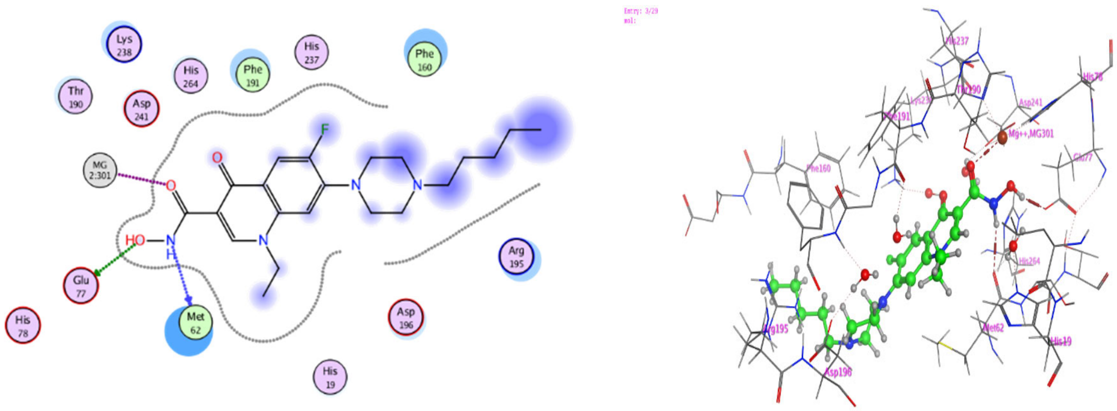

Figure S125: 2D and 3D interactions of compound **11d** with *P. aeruginosa* LpxC.

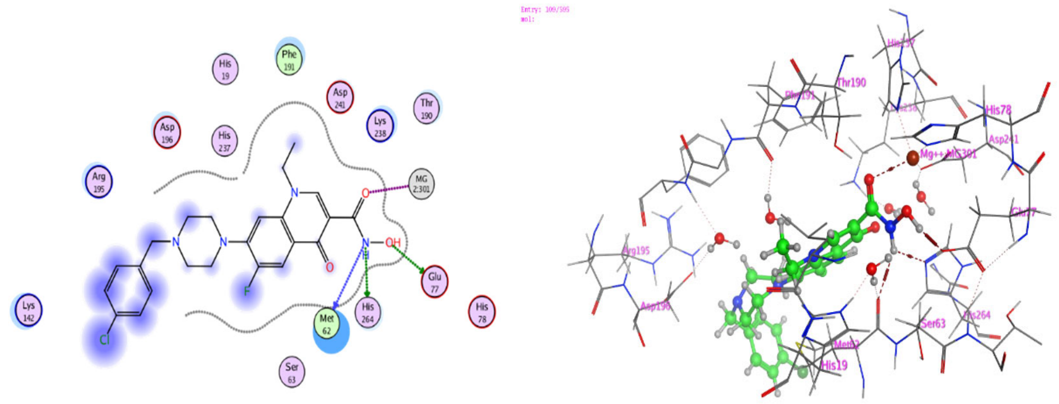

Figure S126: 2D and 3D interactions of compound **11f** with *P. aeruginosa* LpxC.

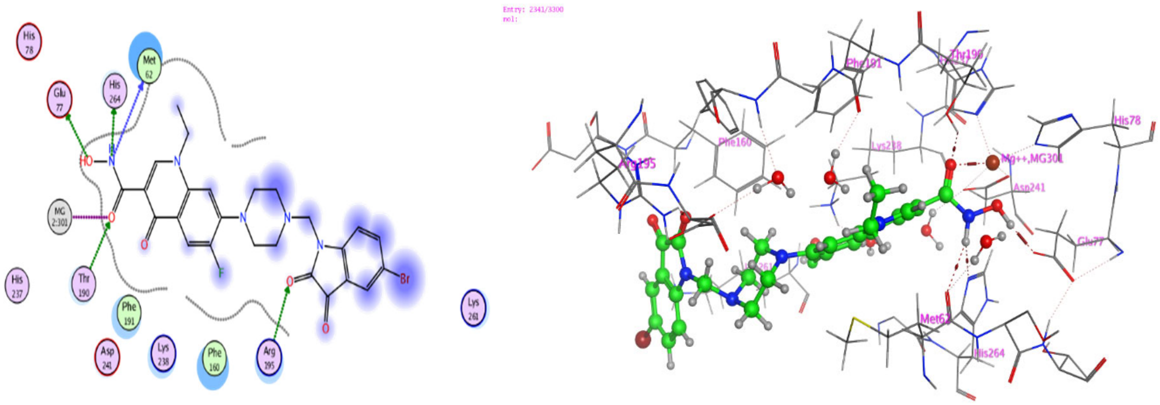

Figure S127: 2D and 3D interactions of compound **17b** with *P. aeruginosa* LpxC.

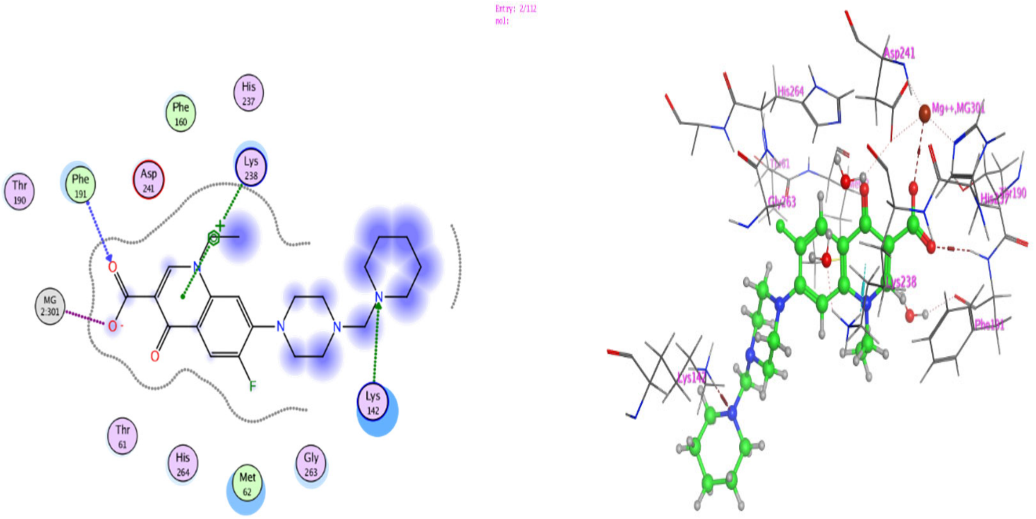

Figure S128: 2D and 3D interactions of compound **19a** with *P. aeruginosa* LpxC.

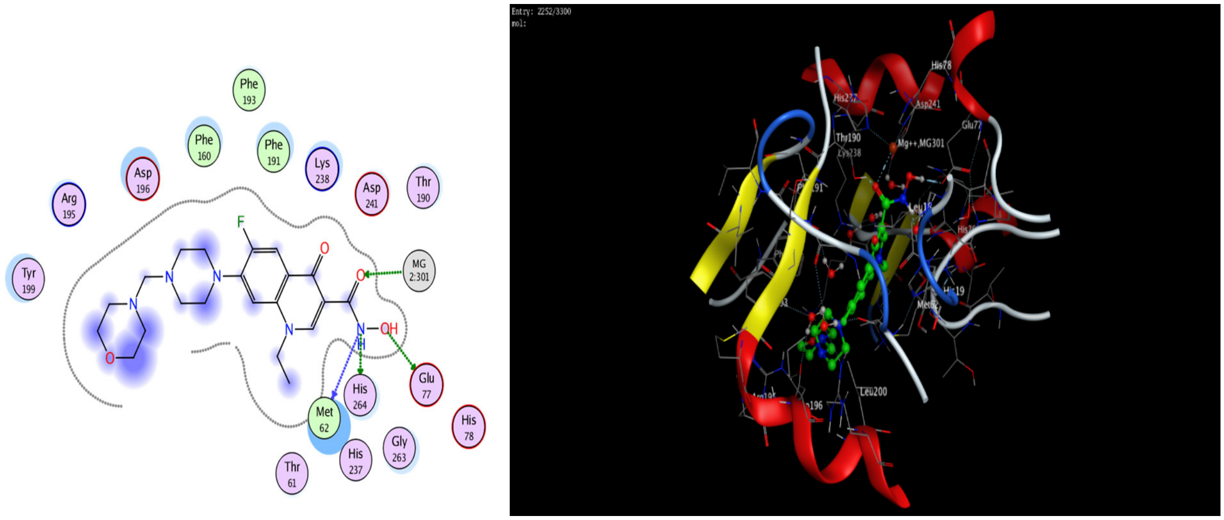

Figure S129: 2D and 3D interactions of compound **20b** with *P. aeruginosa* LpxC.

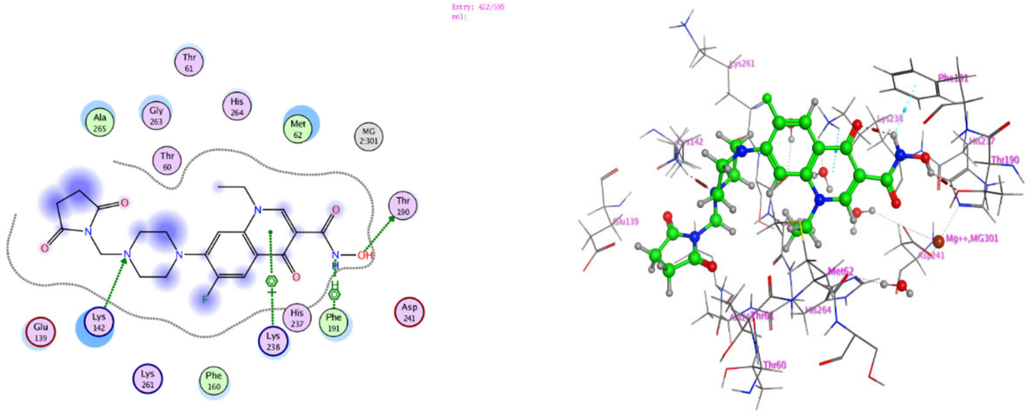

Figure S130: 2D and 3D interactions of compound **23a** with *P. aeruginosa* LpxC.

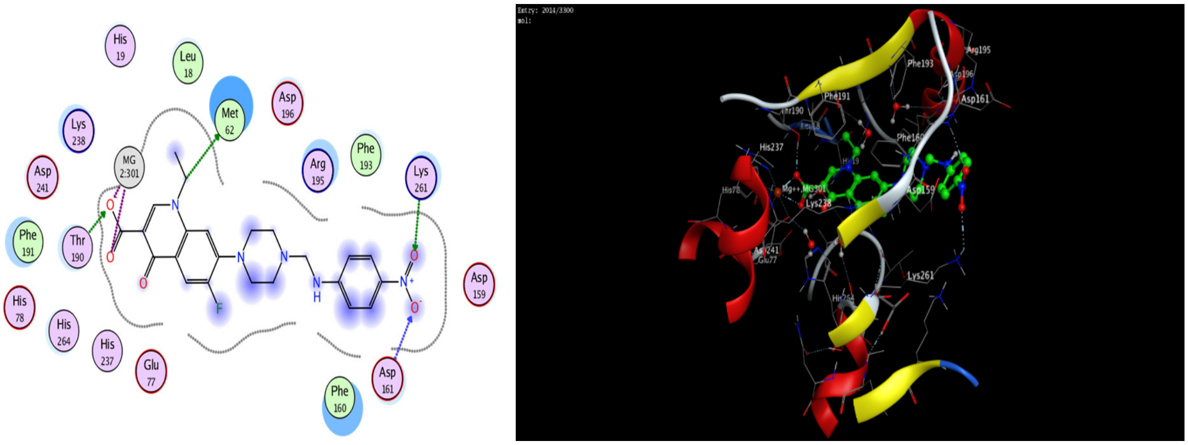

Figure S131: 2D and 3D interactions of compound **25** with *P. aeruginosa* LpxC.

Table S7: Binding scores with *P. aeruginosa* LpxC.

Compound	Binding score ΔG (kcal mol^{-1})	Amino acids involved in interaction	MIC on *E. coli* (μM)
**11a**	-8.41	Thr190, Phe191, Lys142, Lys238, Met62, Ala265, His264, Gly263, Glu77, Asp241, Mg^{2+}	**0.18**
**11b**	-8.94	Thr190, Phe191, Lys142, Lys238, Met62, Ala265, His264, Gly263, Glu77, Asp241, Mg^{2+}	**2.75**
**11f**	-9.48	Thr190, Phe191, Phe160, Lys238, Met62, Ala265, His264, Gly263, Glu77, Asp241, Leu18, Gly192, Mg^{2+}	**1.08**
**19a**	-9.23	Thr190, Phe191, Phe160, Lys238, Met62, Ala265, His264, Gly263, Leu18, Mg^{2+}	**0.3**
**25**	-9.58	Thr190, Phe191, Lys142, Lys238, Met62, Ala265, His264, Gly263, His19, Mg^{2+}	**0.266**
**17b**	-9.45	Thr190, Phe191, Phe160, Lys238, Met62, Ala265, His264, Gly263, Glu139, Asp241, Arg195, Mg^{2+}	**2.62**
**20b**	-9.94	Thr190, Phe191, Phe160, Lys238, Met62, Ala265, His264, Gly263, Glu139, Leu18, Asp241, Mg^{2+}	**0.28**
**23a**	-9.66	Thr190, Phe191, Lys142, Lys238, Met62, Ala265, His264, Gly263, Glu139, Asp241, Leu266, Mg^{2+}	**0.56**

Text S9: Ligand-based pharmacophore modelling

Ligand-based pharmacophore modeling was performed using MOE 2020.01, which was enabled by using 57 LpxC inhibitors and their corresponding $IC_{50\%}$ values. To achieve a significant pharmacophore model, the following criteria was maintained during selection of the training set compounds: all 40 compounds have an excellent range of experimental activities against LpxC enzyme, were minimized to the most stable conformation on the MOE interface, and the selected training set are of variable chemical structures. Common pharmacophoric features were obtained after performing flexible alignment for the training set. The process was completed after it reached its maximum iteration limit (see **Methods**). One hundred flexible alignments resulted in different scores, the best one being -85.84 kcal mol^{-1}. The best obtained model consisted of four pharmacophore features which have mutual distance constraints between each other (**Table S8-9**, **Figure S132**). The model was validated against the validation test set database and identified 13 hits of 17 entries with the required pharmacophoric features. All reported LpxC inhibitors used in this study share four common structural features, including hydrophobic regions essential for occupying the hydrophobic tunnel at active site of and metal ligating sites (hydrogen bond donor-acceptor, hydroxamic acid group) that is crucial for chelation of the metal ion at active site.

The set of target compounds consisting of our newly designed compounds was built and minimized to the least conformational energy. The pharmacophoric search of this test set on the validated pharmacophore query was performed resulting in 55 hits out of 55 total entries. All compounds possess a hydrophobic side chain in addition to the quinoline core and a metal ligator group represented by the carboxylic or hydroxamic groups, showing that they could potentially bind to LpxC. Compounds showing the best hits are overlapping with all features of the generated pharmacophore query with rmsd values of 0.7703 (**7b**), 0.7228 (**12b**), 0.5182 (**10e**), 0.5491 (**8a**), 0.5839 (**8b**), 0.7148 (**11b**), 0.7272 (**11c**), 0.7253 (**16b**), 0.6046 (**16f**), 0.6510 (**19a**), 0.6922 (**17b**), 0.5689 (**17f**), and 0.6031 (**23b**), respectively. **Figure S133** shows the overlay of compounds **8a**, **8b**, **11e**, **17b**, **17f**, and **23b** with the generated pharmacophore query.

Virtual ligand-based pharmacophore screening showed that norfloxacin had an rmsd value of 0.9506, while most of the new derivatives showed lower rmsd values, suggesting that their structural modifications increase the probability of an interaction with LpxC. This is illustrated by the alignment of compounds **8a** and **17f** with the reported LpxC inhibitor **CHIR-12** with alignment scores of -85.59 and -66.80 kcal mol^{-1}, respectively (**Figure S134**).

Another interesting finding was that rmsd values of most hydroxamic acid derivatives were lower than those of derivatives with carboxylic acid groups. In the case of Mannich base derivatives, all hydroxamic acids have lower rsmd values than their corresponding carboxylic acid variants except for **20a** (piperidine) and **23a** (succinimide). Similarly, hydroxamic acids of acyl, alkyl, and phenacyl derivatives showed lower rmsd values than their carboxylic acids with the exception of **13b** (p-Brphenacyl).

Table S8: Query features calculated from the aligned molecules.

Feature	Radius	Description
F1 Hyd | Aro	2.3 Å	Hydrophobic region | Aromatic ring center
F2 Aro | Hyd	2.9 Å	Aromatic ring center | Hydrophobic region
F3 ML | Acc | Don	1.5 Å	Metal ligator | H-bond acceptor | H-bond donor
F4 ML | Acc | Don	1.3 Å	Metal ligator | H-bond acceptor | H-bond donor

Table S9: Pharmacophore features with distance constraints (Å). Pharmacophore features have mutual distances between each other.

Feature	F1 Hyd | Aro	F2 Aro | Hyd	F3 ML | Acc | Don	F4 ML | Acc | Don
F1 Hyd | Aro	0 Å	4.38 Å	11.44 Å	10.70 Å
F2 Aro | Hyd	4.38 Å	0 Å	7.13 Å	6.72 Å
F3 ML | Acc | Don	11.44 Å	7.13 Å	0 Å	4.40 Å
F4 ML | Acc | Don	10.70 Å	6.72 Å	4.40 Å	0 Å

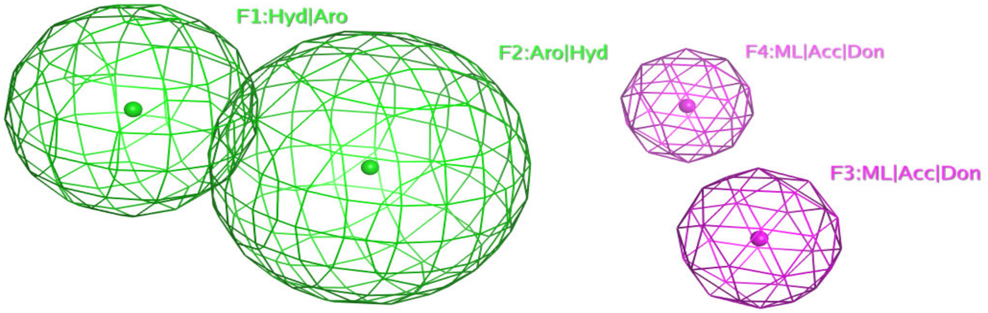

Figure S132: Query features calculated from the aligned molecules.

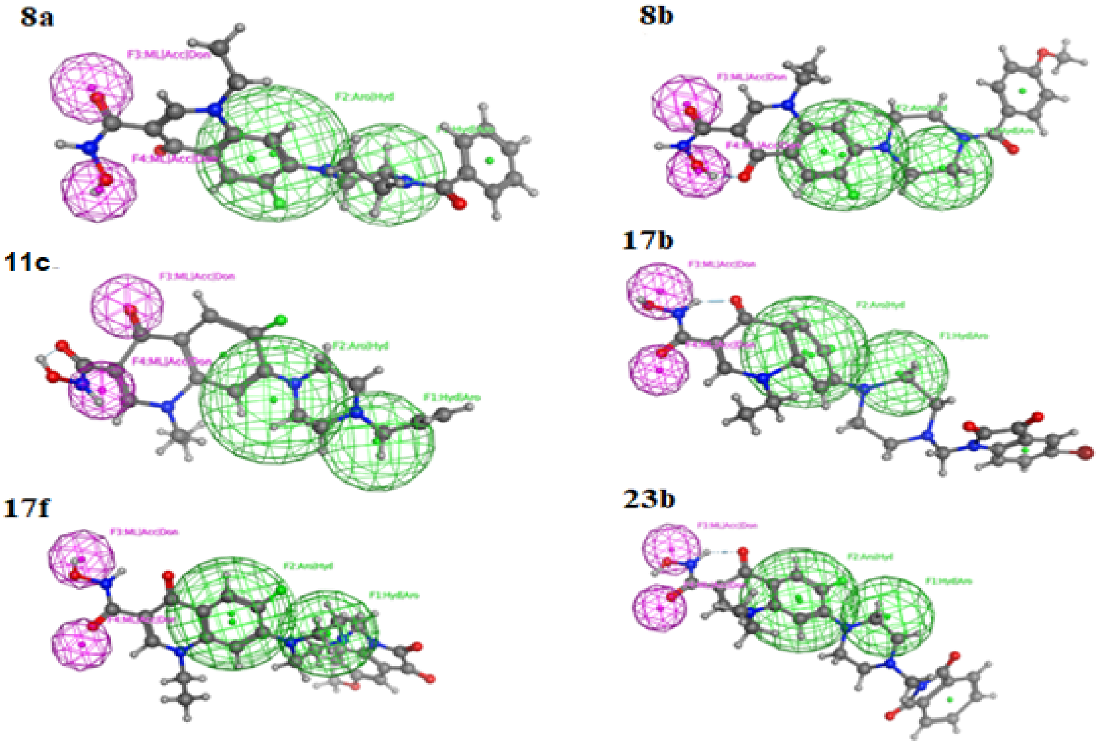

Figure S133: Overlaying of some target compounds with the generated pharmacophore query.

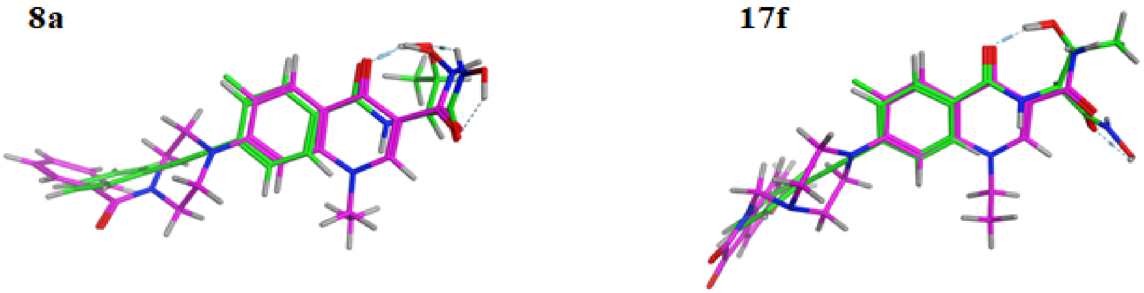

Figure S134: Alignment of compounds **8a** and **17f** (violet) and **CHIR-12** (green).

5. Mechanism of action

Text S10: Metal-chelating properties

The ability of compounds **11a**, **11b**, **11f (**series 1), **17a**, **20b**, and **23a (**series 2) to chelate metals such as Mg^{2+}, Zn^{2+}, and Cd^{2+} was studied by UV–vis spectrometry[34]. The absorption spectra of the compounds (30 μM), alone (in methanol) and in the presence of $MgCl_2$, $ZnCl_2$ and $CdCl_2$ (20 μM), were recorded at room temperature in a 1 cm quartz cell using a UV-Vis spectrophotometer. It should be mentioned that different concentrations of each compound were recorded and 30 μM was found to give reasonable absorbance obeying Lambert-Beer law in the best absorbance range of 0.1 to 0.9[35, 36].

When the compounds were mixed with each metal solution (20 μM), a spectral change was observed, which was attributed to complex formation between the compounds and metal (**Figure S135-136**). Metal binding led to a decrease in absorption (hypochromic shift) and a bathochromic shift with a maximum peak at 278-282 nm resulting from a charge transfer processes between the coordinated hydroxamic acid group of the tested compounds and metal.

Additionally, the ratio of ligand/metal ion in complex was determined by a molar ratio method[37, 38], where fixed concentrations of the compounds (30 μM) were mixed with ascending concentrations of each metal (15–35 μM). It was observed that the spectra showed no change in absorption intensity at 1:1 molar ratio, suggesting that the molar ratio of ligand/metal ion in the complex was 1:1 (**Figure S137**). This is in line with previous studies, in which quinolones could form metal complexes with 1:1, 1:2, and 1:3 metal/ligand ratios[39]. The results revealed that the investigated compounds had higher affinity for binding and chelation of zinc than magnesium and cadmium and higher affinity than norfloxacin, as concluded from higher hypochromic shift values (**Table S10**).

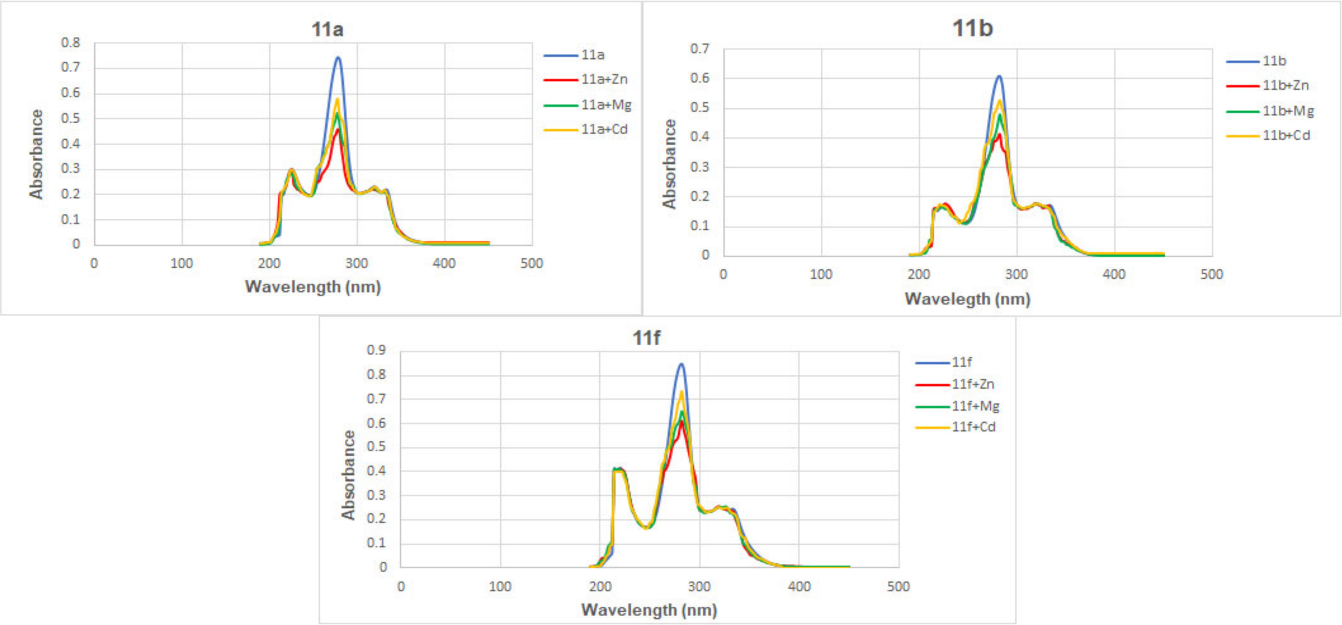

Figure: S135: UV–vis absorption spectra of series **1** compounds **11a, 11b** and **11f.**

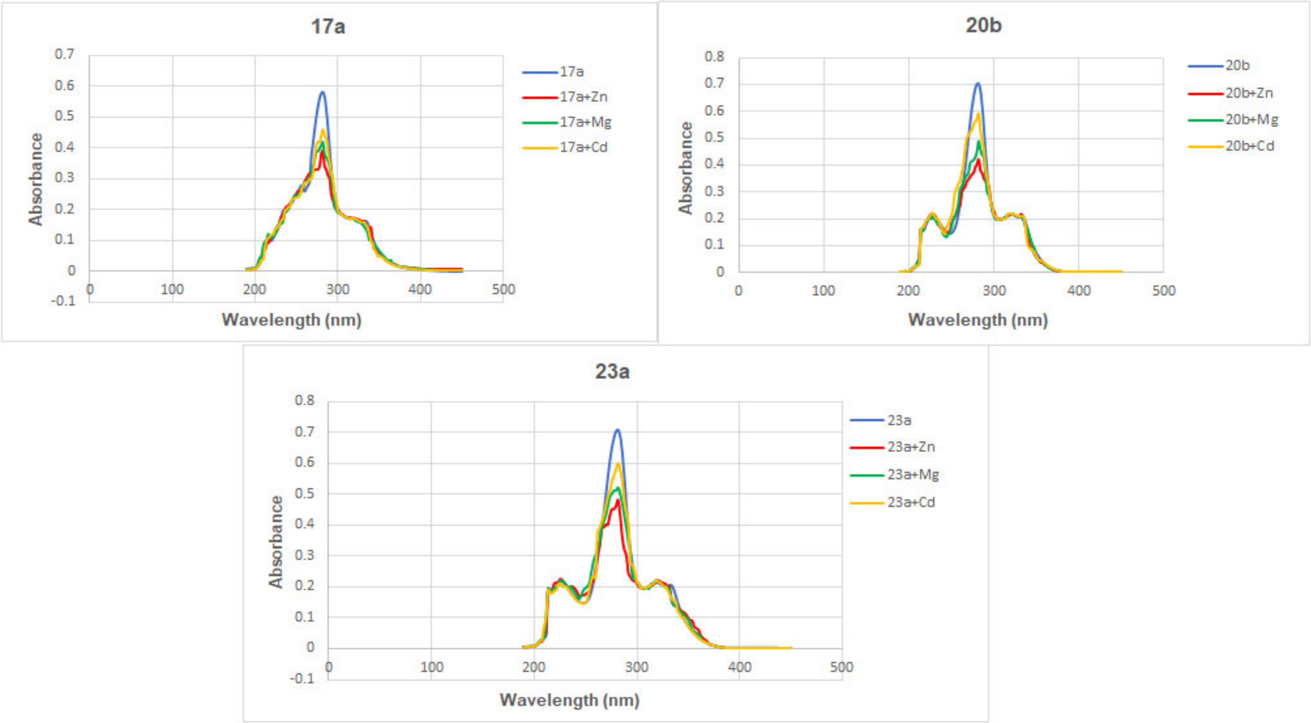

Figure: S136: UV–vis absorption spectra of series **2** compounds **17a, 20b** and **23a.**

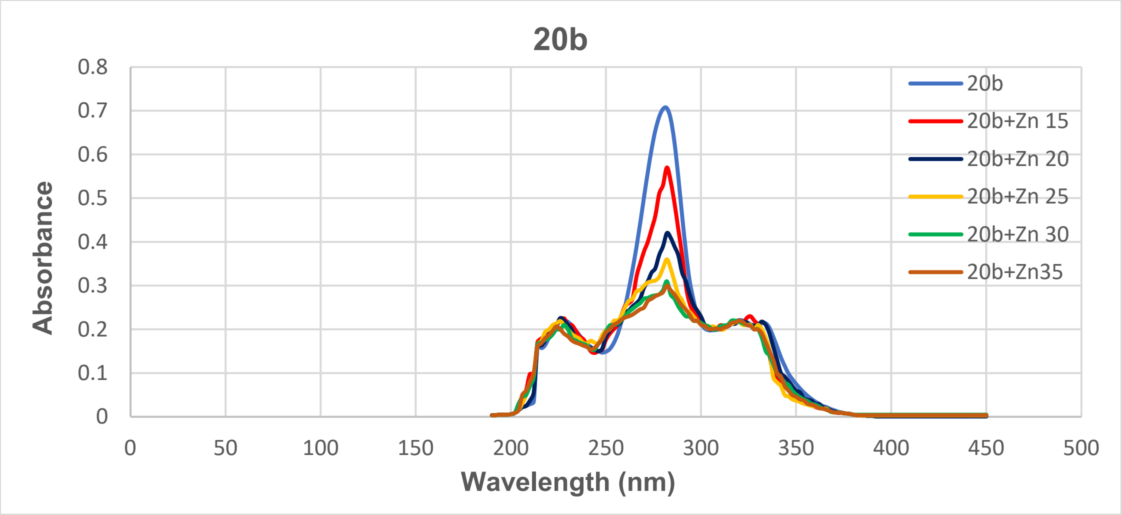

Figure S137: The molar ratio of ligand/metal in metal complex of compound **20b**.

Table S10: Absorbance of investigated compounds and their metal complexes.

**Code**	**Absorbance**			
	**Original**	**Zn^{2+} complex**	**Mg^{2+} complex**	**Cd^{2+} complex**
**Nor**	0.715	0.542	0.583	0.647
**11a**	0.743	0.46	0.523	0.581
**11b**	0.61	0.413	0.48	0.526
**11f**	0.85	0.61	0.653	0.734
**17a**	0.582	0.39	0.422	0.46
**20b**	0.706	0.425	0.49	0.591
**23a**	0.71	0.482	0.52	0.603

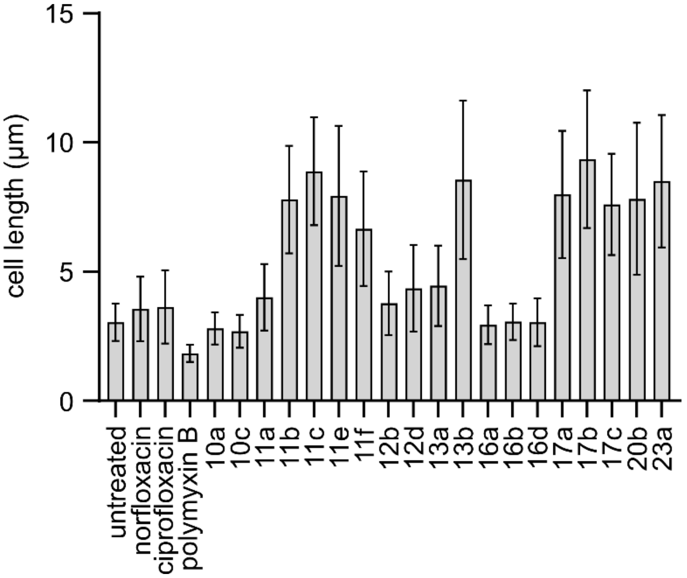

Figure S138: Cell length of *E. coli* W3110 measured from BCP images. A minimum of 50 cells were measured per sample. Error bars represent standard deviation of the mean of three biological replicates.

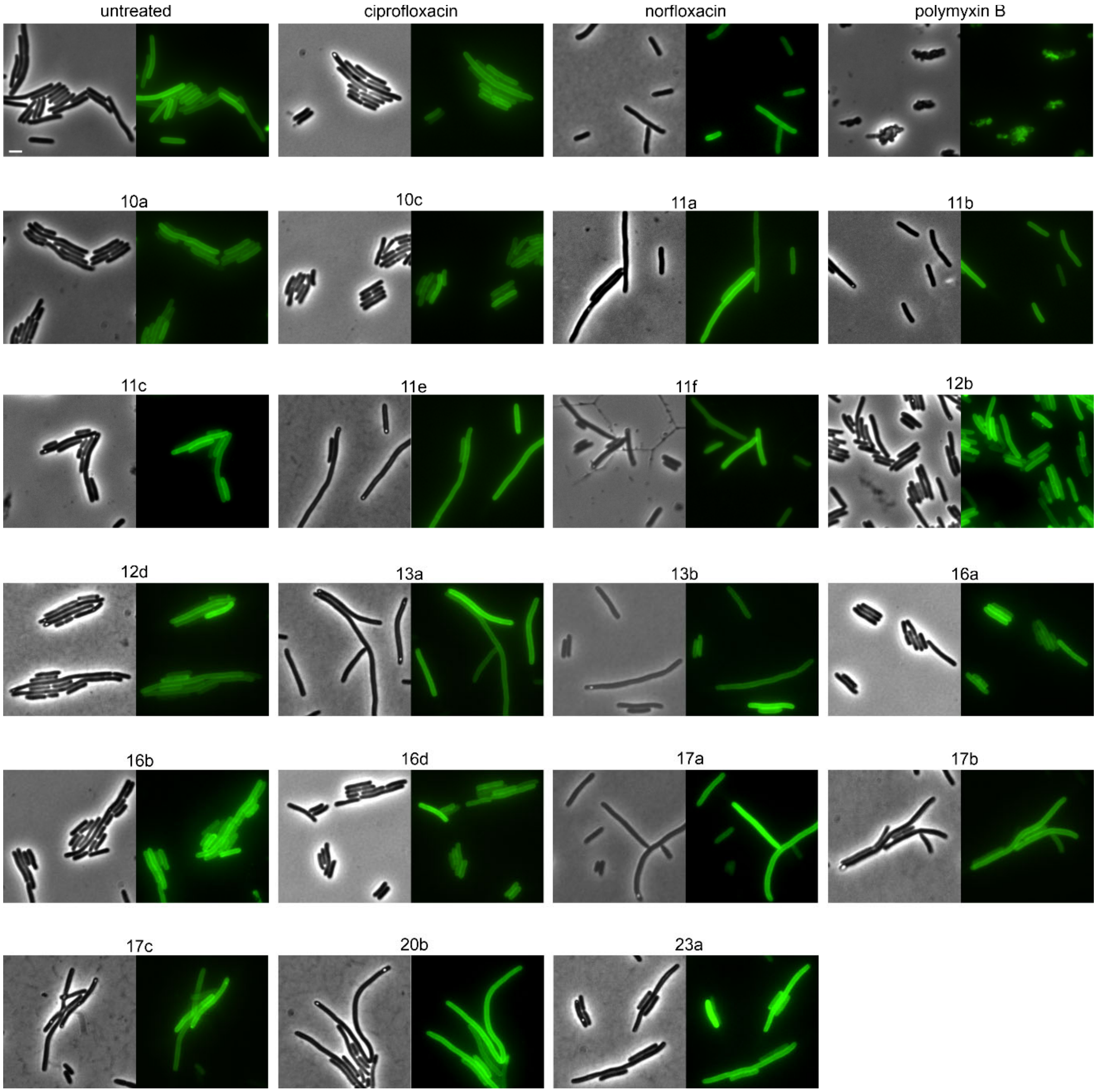

Figure S139: Fluorescence and phase contrast microscopy of *E. coli* BCB472. Cells were treated with 1xMIC of the respective compounds for 1 h prior to microscopy. Expression of NeonGreen-GlpT was induced with 10 µM IPTG for 1 h (concomitantly with antibiotic incubation). Scale bar 2 µm.

Table S11: Results summary of bacterial cytological profiling in *E. coli*. Phase contrast images indicate cell lysis. The fluorescent membrane dye FM4-64 and the GFP-tagged membrane protein GlpT report on membrane effects. The fluorescent DNA stain DAPI reports on DNA condensation. Cip = ciprofloxacin, Nor = norfloxacin, PolB = polymyxin B.

**Compound**	**concentration (µg/mL)**	**phase contrast**	**FM4-64**	**GlpT**	**DAPI**	**gyrase inhibition?**	**membrane damage?**
Untreated		dark	smooth	smooth	regular	no	No
Cip	0.37	dark	smooth	smooth	condensed	yes	no
Nor	0.39	dark	smooth	smooth	condensed	yes	no
PolB	0.83	light	patchy	dispersed	dispersed	no	yes
**10a**	5.99	dark	smooth	smooth	condensed	yes	no
**10c**	5.56	dark	smooth	smooth	condensed	yes	no
**11a**	0.18	dark	smooth	smooth	condensed	yes	no
**11b**	2.75	dark, elongated	smooth	smooth	condensed	yes	no
**11e**	2.35	dark, elongated	smooth	smooth	condensed	yes	no
**11f**	1.08	dark, elongated	smooth	smooth	condensed	yes	no
**12c**	70.87	dark, elongated	smooth	smooth	condensed	yes	no
**12b**	4.84	dark, elongated	smooth	smooth	condensed	yes	no
**12d**	5.18	dark, elongated	smooth	smooth	condensed	yes	no
**13a**	2.21	dark, elongated	smooth	smooth	condensed	yes	no
**13b**	1.88	dark, elongated	smooth	smooth	condensed	yes	no
**16a**	5.22	dark	smooth	smooth	condensed	yes	no
**16b**	4.48	dark	smooth	smooth	condensed	yes	no
**16d**	3.89	dark	smooth	smooth	condensed	yes	no
**17a**	3.03	dark, elongated	smooth	smooth	condensed	yes	no
**17b**	2.62	dark, elongated	smooth	smooth	condensed	yes	no
**17c**	2.93	dark, elongated	smooth	smooth	condensed	yes	no
**20b**	0.28	dark, elongated	smooth	smooth	condensed	yes	no
**23a**	0.56	dark, elongated	smooth	smooth	condensed	yes	no

Table S12: Results of checkerboard assays of norfloxacin derivatives combined with mupirocin. FICI values represent the average of at least two replicate experiments. FICI values were interpreted as follows: synergy, FICI of ≤0.5; additivity or partial synergy (indicates increase in inhibitory activity from the additive effect of both compounds combined), FICI of >0.5 to ≤1; no interaction (indifference), FICI of >1 to ≤4; antagonism, FICI of >4. Mup = muprocin, Cip = ciprofloxacin, Nor = norfloxacin, PolB = polymyxin B.

**Compound**	**MIC_C**	**$MIC_C^{checkerboard}$**	**FIC_C**	**$MIC_M^{checkerboard}$**	**FIC_M**	**FICI**	**Outcome**
Mup	64	-	-	-	-	-	
Cip	0.125	0.031	0.25	2	0.031	0.281	Synergistic
Nor	0.125	0.039	0.25	32	0.5	0.813	Additive
ACHN-975	0.5	0.125	0.25	3	0.047	0.297	Synergistic
PolBN	128	1	0.0078	1	0.016	0.023	Synergistic
**4a**	8	0.031	0.004	64	1	1.004	Additive
**10a**	2	0.008	0.004	64	1	1.004	Additive
**10c**	2	0.008	0.004	64	1	1.004	Additive
**10f**	32	0.125	0.004	64	1	1.004	Additive
**11a**	0.125	0.032	0.26	48	0.75	1.01	Additive
**11f**	0.5	0.501	1.002	48	0.75	1.752	Additive
**12b**	2.5	0.01	0.004	64	1	1.004	Additive
**12d**	2.5	2.505	1.002	48	0.75	1.752	Additive
**16a**	2.5	0.63	0.252	36	0.563	0.815	Additive
**16b**	2.5	0.313	0.125	64	1	**0.172**	**Synergistic**
**16d**	2	0.008	0.004	64	1	1.004	Additive
**17c**	1.5	0.006	0.004	64	1	1.004	Additive
**17e**	4	0.016	0.004	64	1	1.004	Additive
**19b**	8	4	0.5	3	0.047	**0.547**	**Synergistic**
**20b**	0.125	0.064	0.51	48	0.75	1.26	Additive
**25**	0.125	0.0005	0.0039	64	1	1.004	Additive

MIC_C: MIC of the test compound alone, $MIC_C^{checkerboard}$: MIC of the test compound in checkerboard assay, FIC_C: fractional inhibitory concentration of the test compound ($FIC_C=MIC_C^{checkerboard}/MIC_C$), MIC_M: MIC of mupirocin alone (64 µg/mL), $MIC_M^{checkerboard}$: MIC of mupirocin in checkerboard assay, FIC_M: fractional inhibitory concentration of the test compound ($FIC_M=MIC_M^{checkerboard}/MIC_M$), FICI: fractional inhibitory concentration index = FIC_C+FIC_M.

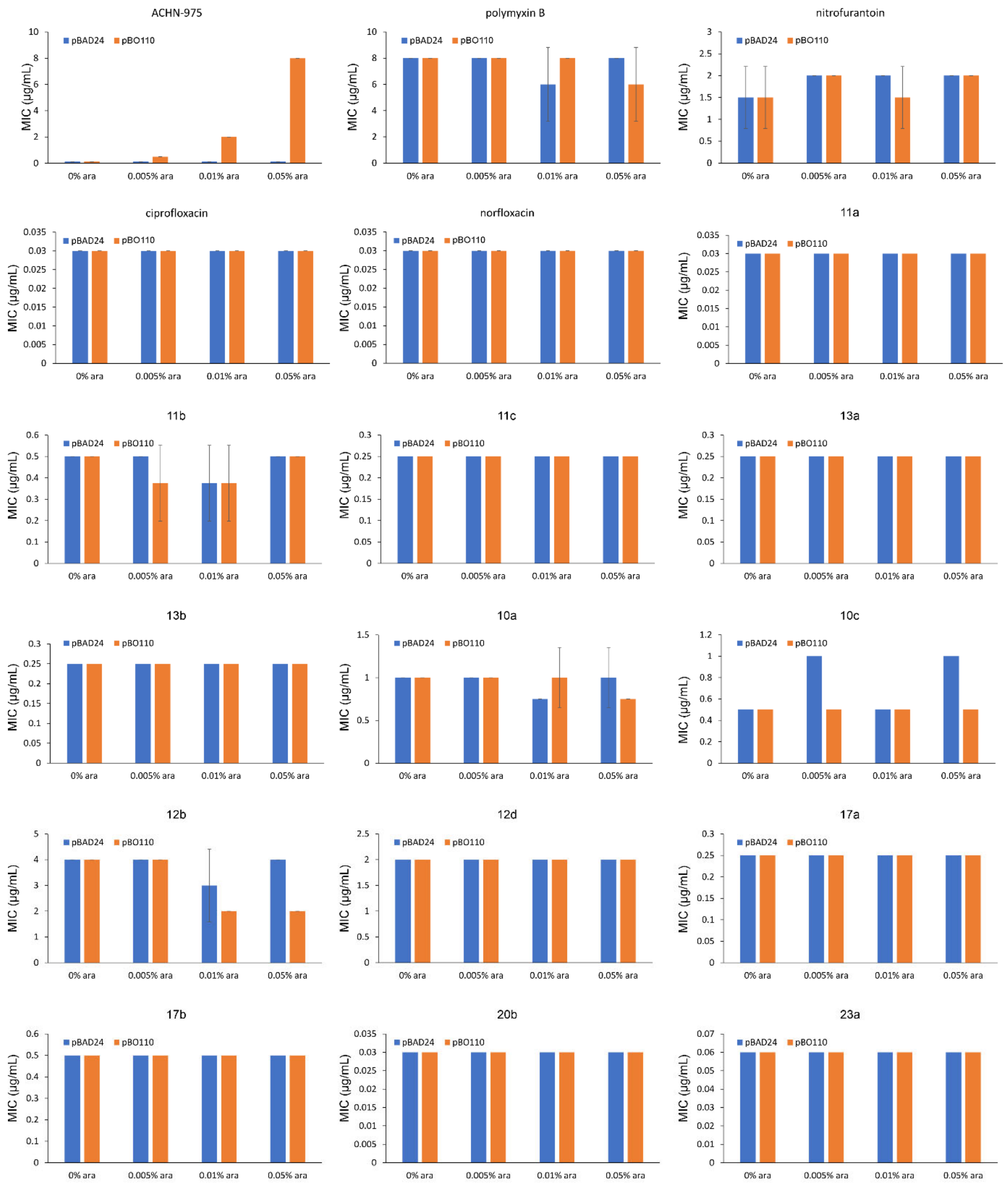

Figure S140: Effects on LpxC. *E. coli* BL21 DE03 carrying pBO110, expressing LpxC from the arabinose-inducible P_{BAD} promoter, was grown in presence of increasing arabinose concentrations. Overexpression of LpxC leads to accumulation of lipidA in the cell membrane, which is toxic for *E. coli*. Inhibition of LpxC activity mitigates this effect. As controls for the presence of sugar, parallel cultures were grown in the presence of glucose. *E. coli* BL21 DE03 carrying pBAD24 was included as empty vector control.

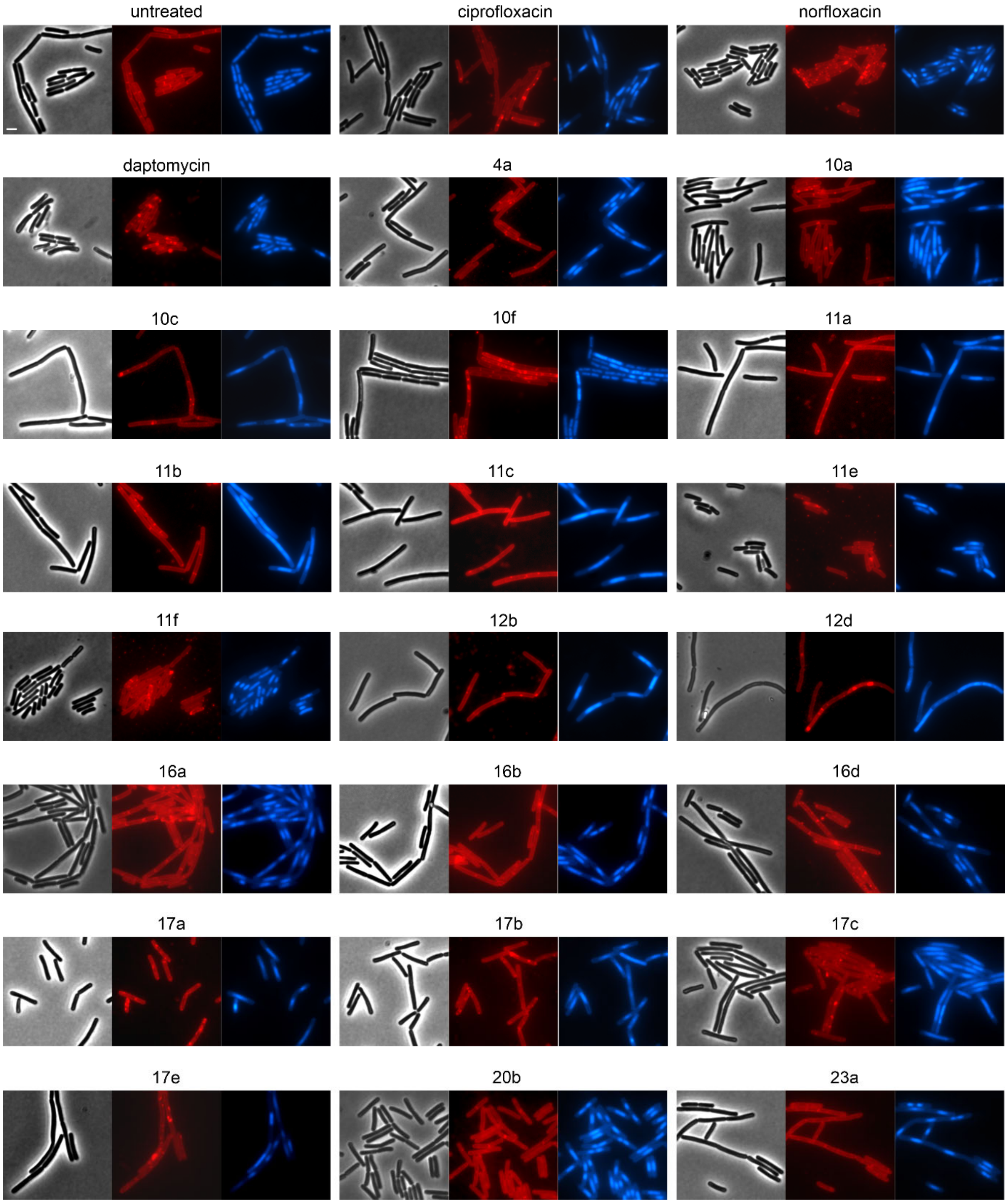

Figure S141: Bacterial cytological profiling of *B. subtilis*. Fluorescence and phase contrast microscopy of *B. subtilis* DSM402. Cells were treated with 1x MIC of the respective compounds for 1 h prior to staining with FM4-64 (membrane, red) and DAPI (nucleoid, blue). Scale bars 2 µm.

Table S13: Results summary of bacterial cytological profiling of *B. subtilis*. Phase contrast images indicate cell lysis. The fluorescent membrane dye FM4-64 reports on membrane effects. The fluorescent DNA stain DAPI reports on DNA condensation. Cip = ciprofloxacin, Nor = norfloxacin, Dap = daptomycin.

**Compound**	**concentration (µg/mL)**	**phase contrast**	**FM4-64**	**DAPI**	**membrane damage?**	**gyrase inhibition?**
untreated		dark	smooth	regular	no	no
Cip	3.01	dark, elongated	smooth/patchy	diffuse	heterogenous	yes
Nor	18.11	dark, elongated	patchy	diffuse	yes	yes
Dap	0.61	light	patchy	regular	yes	No
**4a**	72.43	dark, elongated	smooth	condensed	no	yes
**10a**	47.99	dark, elongated	patchy	condensed	yes	yes
**10c**	33.38	dark, elongated	patchy	condensed	yes	yes
**10f**	18.02	light, elongated	patchy	regular	yes	no
**11a**	2.87	dark, elongated	smooth/spotty	condensed	heterogenous	yes
**11b**	22.07	dark, elongated	smooth/spotty	condensed	heterogenous	yes
**11c**	1.33	dark, elongated	smooth/spotty	condensed	heterogenous	yes
**11e**	1.17	dark	smooth/patchy	regular	heterogenous	no
**11f**	1.08	dark, elongated	smooth/patchy	condensed	heterogenous	yes
**12b**	30.98	dark, elongated	patchy	condensed	yes	yes
**12d**	198.98	dark, elongated	patchy	condensed	yes	yes
**16a**	24.31	dark, elongated	patchy/smooth	condensed	heterogenous	yes
**16b**	55.90	dark	smooth	condensed	no	yes
**16d**	31.19	dark, elongated	spotty	condensed	yes	yes
**17a**	33.43	dark, elongated	patchy	condensed	yes	yes
**17b**	7.17	dark, elongated	patchy	condensed	yes	yes
**17c**	23.46	dark, elongated	patchy	condensed	yes	yes
**17e**	126.10	dark, elongated	patchy	condensed	yes	yes
**20b**	6.92	dark, elongated	smooth	condensed	no	yes
**23a**	17.95	dark, elongated	spotty	condensed	yes	yes

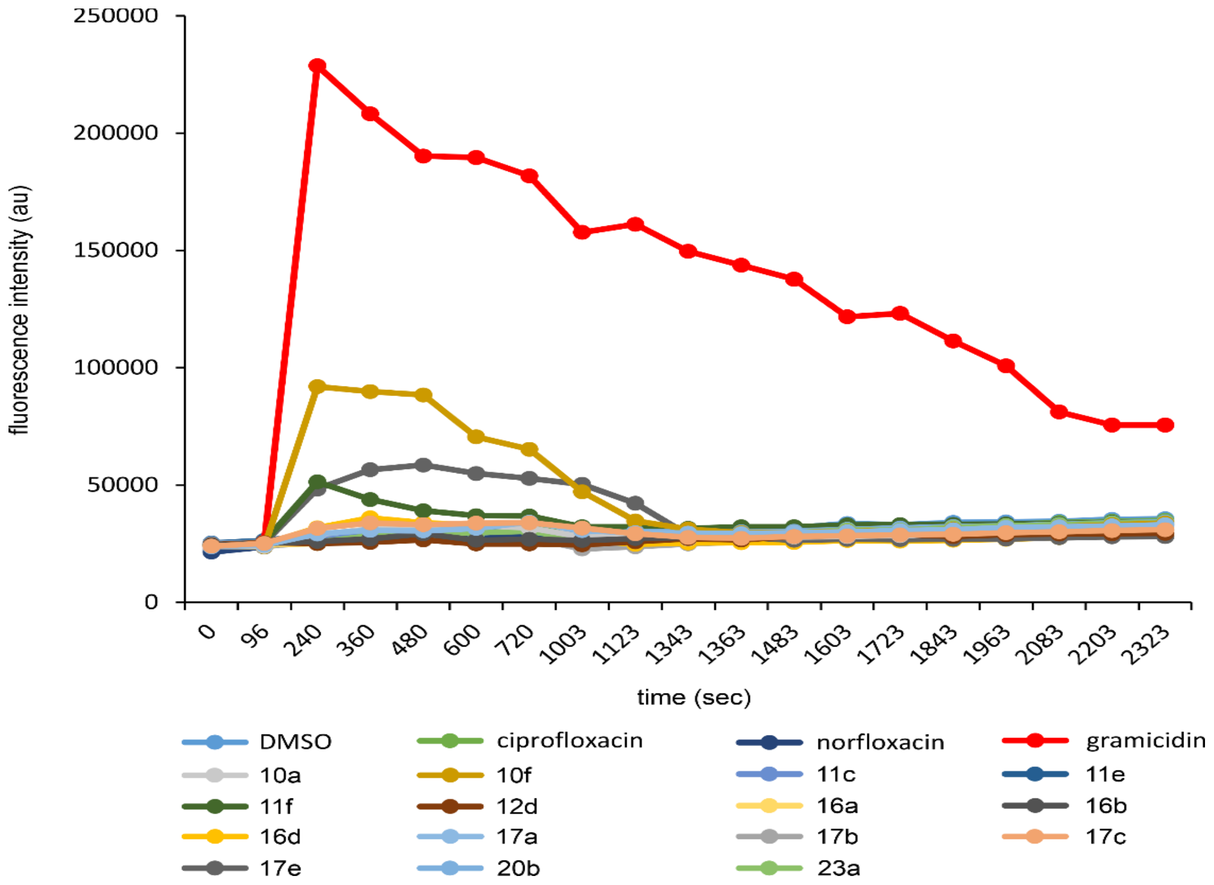

Figure S142: Effects of the membrane potential in *B. subtilis* DSM 402. Bacteria were grown until early log phase in Muller Hinton broth and stained with the self-quenching membrane potentiometric fluorescence probe DiSC(3)5. The dye binds to polarized membranes and self-quenches. Upon depolarization, the dye is released leading to de-quenching and an increased fluorescence signal. Gramicidin (1 µg/mL), which forms a transmembrane ion channel, was used as a positive control.

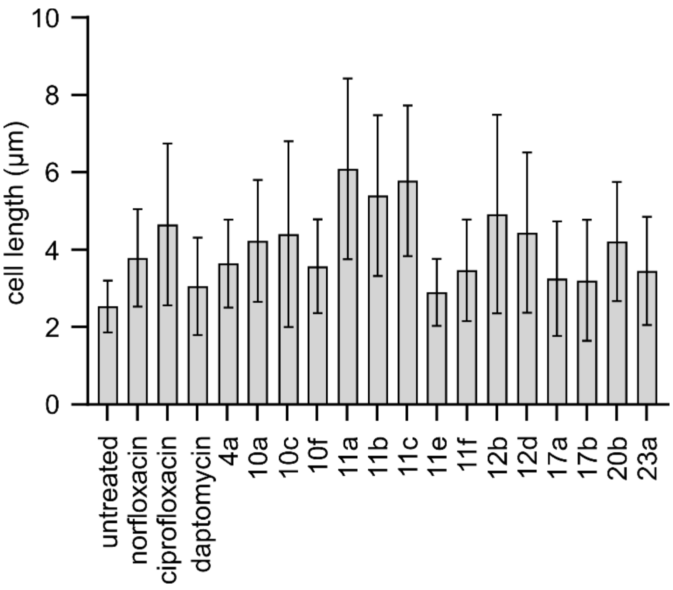

Figure S143: Cell length of *B. subtilis* DSM402 measured from BCP images. A minimum of 50 cells were measured per sample. Error bars represent standard deviation of the mean of three biological replicates.

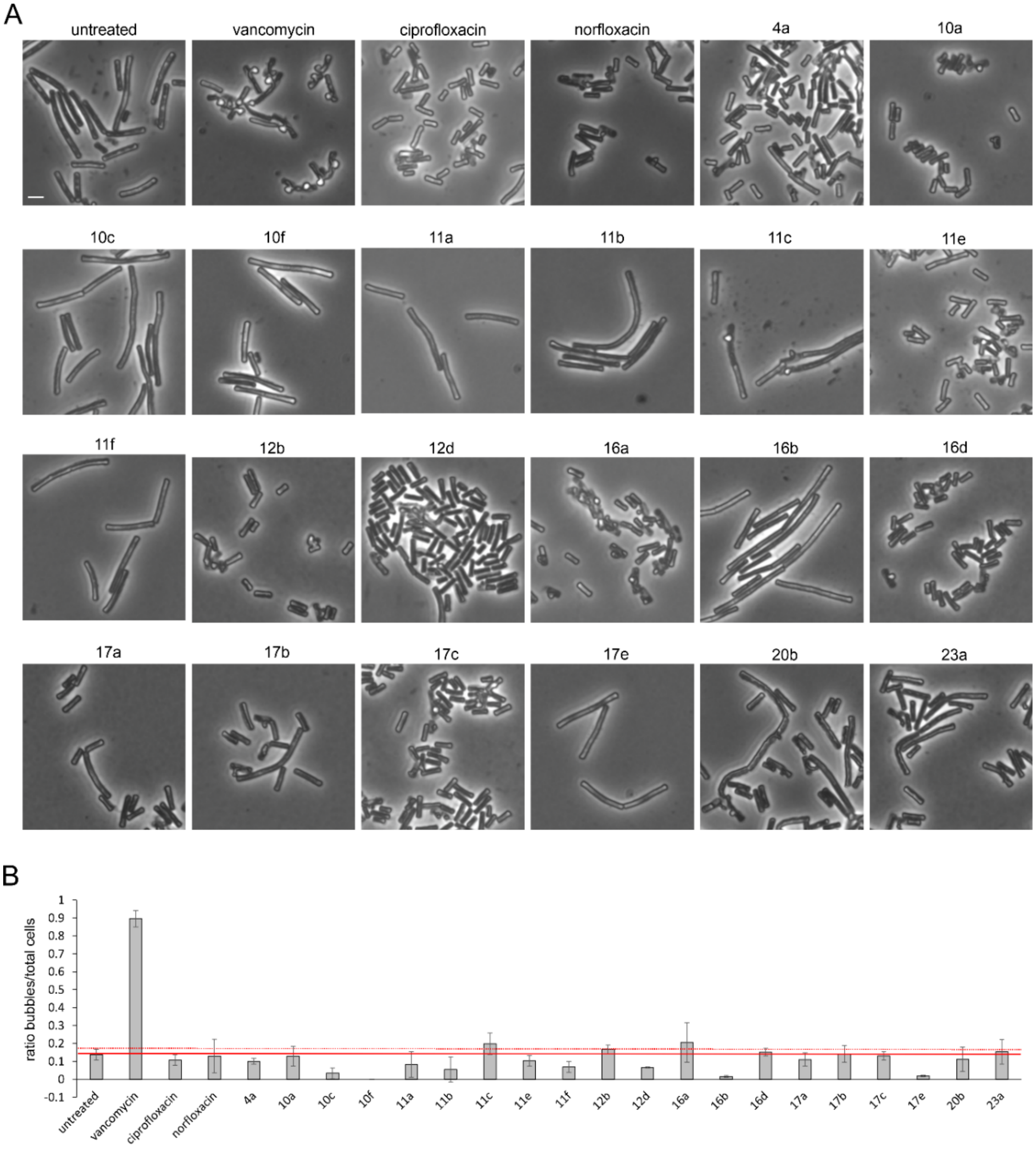

Figure S144: Effects on peptidoglycan synthesis. (A) Phase contrast microscopy of *B. subtilis* 168CA. Cells were treated with 1xMIC of the respective compounds for 10 min (fosfomycin, tunicamycin, vancomycin) or 1 h (all other compounds) prior to fixation in 1:3 acetic acid/methanol. (B) Quantification of microscopy images shown as ratio of bubbles per total number of cells. Error bars show standard deviation of three datasets. A minimum of 50 cells were examined per individual sample. Solid red line indicates the average, dotted red line the upper margin of standard deviation in the untreated control sample. Scale bar 2 µm.

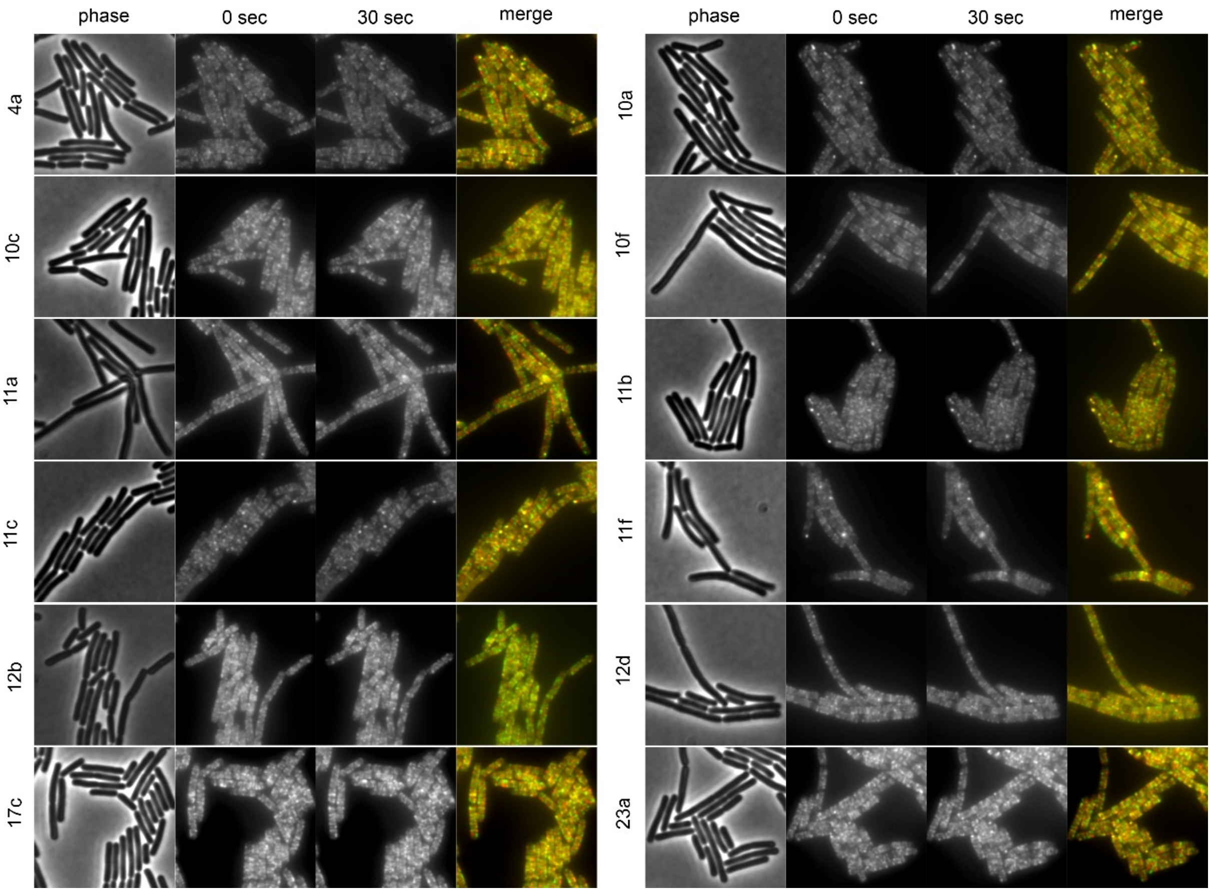

Figure S145: Fluorescence and phase contrast microscopy of *B. subtilis* MW10. Expression of MreB-msfGFP was induced with 0.3% xylose. Cells were treated with 1xMIC of the respective compounds for 10 min and 60 min prior to microscopy. Images were taken 30 sec apart to capture MreB mobility. Scale bar 2 µm.

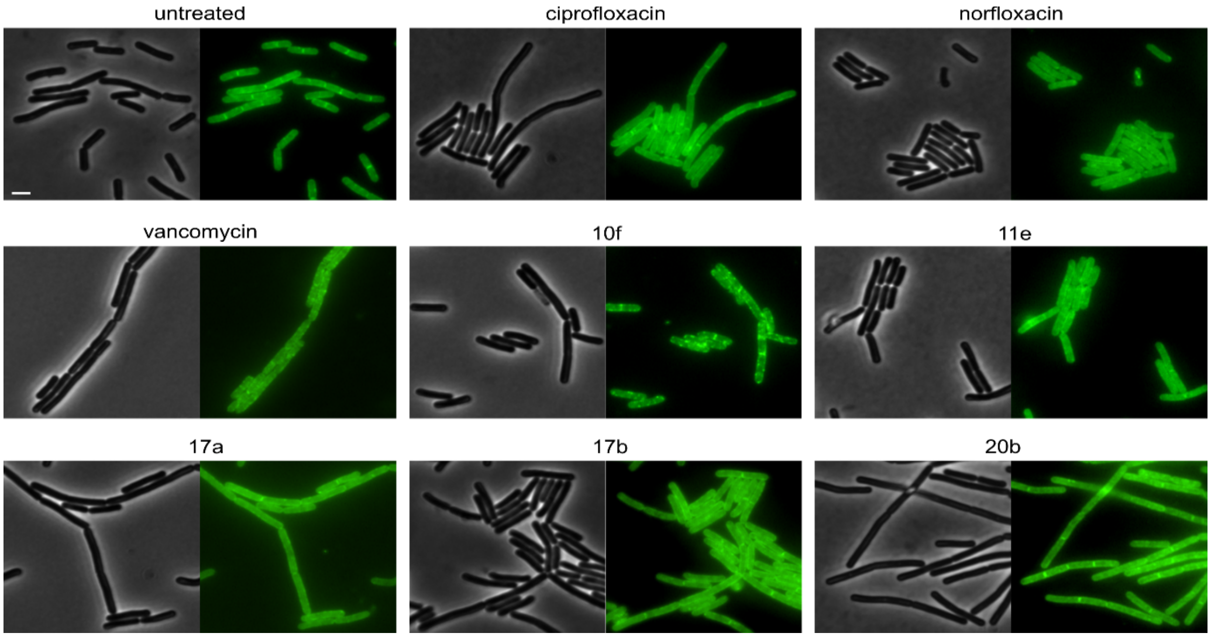

Figure S146: Fluorescence and phase contrast microscopy of *B. subtilis* TNVS284 (MraY-msfGFP). Expression of MraY-msfGFP was induced with 0.1% xylose. Cells were treated with 1xMIC of the respective compounds for 30 min (vancomycin) or 1 h (all other compounds) prior to microscopy. Scale bar 2 µm.

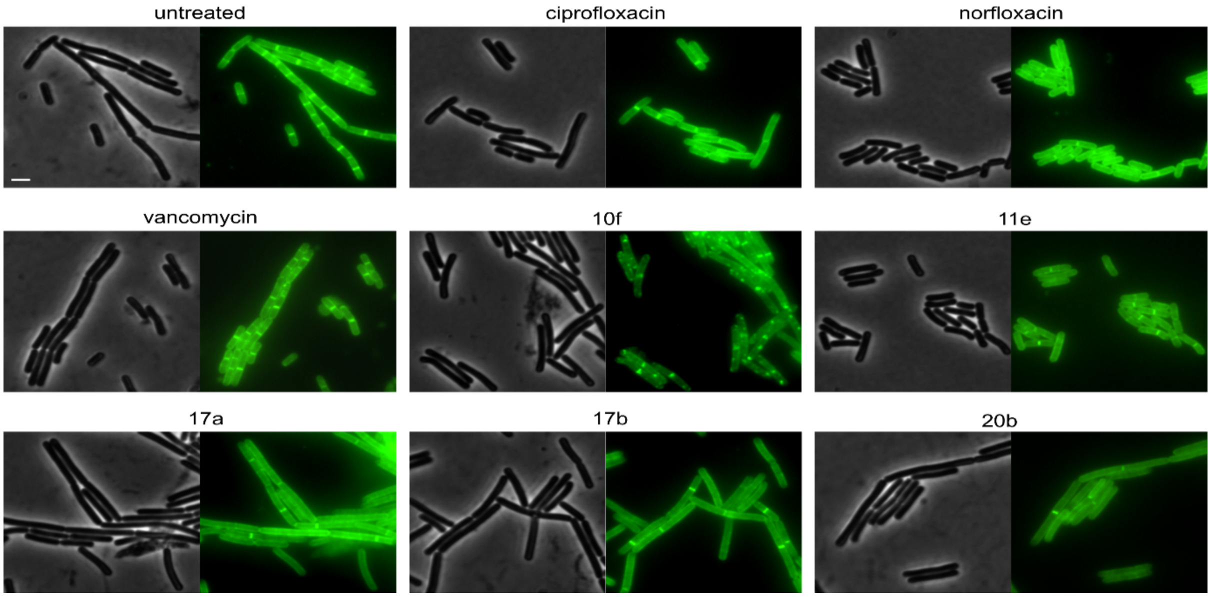

Figure 147: Fluorescence and phase contrast microscopy of *B. subtilis* EKB46 (msfGFP-PbpB). Expression of msfGFP-PbpB was induced with 0.1% xylose. Cells were treated with 1xMIC of the respective compounds for 30 min (vancomycin) or 1 h (all other compounds) prior to microscopy. Scale bar 2 µm.

6. Methods

Text 11: Chemistry

Materials and instruments

All reagents and solvents were of commercially available reagent grade quality and were used without further purification. IR spectra were recorded on a Nicolet® iS5 FT-IR Spectrometer. ^{1}H NMR and ^{13}C NMR spectra were recorded on a Bruker® Advance III (400 MHz). Chemical shifts are reported in δ parts per million (ppm) using TMS as an internal standard and coupling constants (J) are expressed in hertz (Hz). Abbreviations indicating multiplicity were used as follows: s = singlet, bs = broad singlet, d = doublet, t = triplet, q = quartet and m = multiplet. Melting points were measured on a Stuart® SMP10 melting point apparatus.

Synthesis of *N*-substituted piperazinylnorfloxacin hydroxamic acid derivatives.

Synthesis of *N*-acyl norfloxacin derivatives (4a-b, 7a-e)[6].

To stirred solution of 3g (9.39 mmol) norfloxacin in 30 mL anhydrous tetrahydrofuran, 1.9 mL (14.08 mmol) of triethylamine was added, then the reaction mixture was stirred in an ice bath for 5 minutes prior to dropwise addition of 14.08 mmol of acyl chloride or benzene sulphonyl chloride and further stirring for 10 minutes in the ice bath. The mixture was then heated at reflux temperature until the reaction completion as monitored by TLC using DCM/methanol with ratio of 0.3:9.7 as a mobile phase. When the reaction was completed, the reaction mixture was cooled, and the precipitate was filtered, dried, and recrystallized from DMF/water.

7-(4-acetylpiperazin-1-yl)-1-ethyl-6-fluoro-1,4-dihydro-4-oxoquinoline-3-carboxylic acid 4a. Yield= 2.7 g (79%); white powder, mp:297-299 ºC (reported mp: 297-302 ºC)[40]. ^{1}H NMR 400 MHz (DMSO-d_6) δ (ppm): 15.78 (br. s, 1H, COOH), 9.4 (s, 1H, H-2), 8.4 (d, $J_{H\text{-}F}$ = 12.9 Hz, 1H, H-5), 7.63 (d, $J_{H\text{-}F}$ = 6.6 Hz, 1H, H-8), 5.02 (q, J = 4.8, 9.5 Hz, 2H, CH_3-$\underline{CH_2}$), 4.1 (br. m, 4H, 4 H of piperazine near quinolone ring), 3.72 (br. m, 4H, 4H of piperazine near acetyl), 2.5 (s, 3H, CH_3 of acetyl group), 1.85 (t, J = 5.1 Hz, 3H, CH_2-$\underline{CH_3}$).

7-(4-(2-chloroacetyl)piperazin-1-yl)-1-ethyl-6-fluoro-1,4-dihydro-4-oxoquinoline-3-carboxylic acid 4b.

Yield= 3.27 g (88%); beige powder, mp:252-254 ºC (reported mp:252 ºC)[6, 41]. ^{1}H NMR 400 MHz ($CDCl_3$) δ (ppm): 15.29 (br. s, 1H, COOH), 8.6 (s, 1H, H-2), 8.1 (d, $J_{H\text{-}F}$ = 12.9 Hz, 1H, H-5), 6.8 (d, $J_{H\text{-}F}$ = 6.6 Hz, 1H, H-8), 4.65 (q, J = 4.8, 9.5 Hz, 2H, CH_3-$\underline{CH_2}$), 4.05 (s, 2H, -CH_2Cl), 3.7-3.3 (br. m, 8H, 8H of piperazine ring), 1.5 (t, J = 5.1 Hz, 3H, CH_2-$\underline{CH_3}$).

7-(4-benzoyl)piperazin-1-yl)-1-ethyl-6-fluoro-1,4-dihydro-4-oxoquinoline-3-carboxylic acid 7a.

Yield= 3.5 g (88%); white powder, mp:266-268 ºC (reported mp: 263-264 ºC)[42]. ^{1}H NMR 400 MHz ($CDCl_3$) δ (ppm): 15 (s, 1H, COOH), 8.65 (s, 1H, H-2), 8.03 (d, $J_{H\text{-}F}$=12.9 Hz,1H, H-5), 7.45 (m, 5H, Ar-H), 6.86 (d, $J_{\text{H-F}}$ = 6.6 Hz ,1H, H-8), 4.33 (q, J = 7.2 Hz ,2H, CH_3-$\underline{CH_2}$), 3.88 (br. m, 4H, 4H of piperazine near quinolone ring), 3.34 (br. m, 4H, 4H of piperazine near carbonyl of benzoyl), 1.58 (t, J = 5.1 Hz, 3H, CH_2-$\underline{CH_3}$).

7-(4-(4-methoxybenzoyl)piperazin-1-yl)-1-ethyl-6-fluoro-1,4-dihydro-4-oxoquinoline-3-carboxylic acid 7b.

Yield= 3.45 g (81%); white powder, mp:247-249 ºC (no reported mp)[43]. ^{1}H NMR 400 MHz (DMSO-d_6) δ (ppm): 15.32 (s, 1H, COOH), 8.96 (s, 1H, H-2), 7.95 (d, $J_{H\text{-}F}$ =12.9 Hz,1H, H-5), 7.44 (d, J=8.4 Hz, 2H, Ar-H), 7.01 (d, J=8.4 Hz, 2H, Ar-H), 7.22 (d, $J_{\text{H–F}}$ = 6.6 Hz ,1H, H-8), 4.59 (q, J = 7.2 Hz ,2H, CH_3-$\underline{CH_2}$), 3.8 (s, 3H, OCH_3), 3.72 (br. m, 4H, 4H of piperazine near quinolone ring), 3.36 (br. m, 4H, 4H of piperazine near carbonyl of p-Methoxybenzoyl) ,1.41 (t, J = 5.1 Hz, 3H, CH_2-$\underline{CH_3}$).

7-(4-(4-chlorobenzoyl)piperazin-1-yl)-1-ethyl-6-fluoro-1,4-dihydro-4-oxoquinoline-3-carboxylic acid 7c.

Yield= 3.2 g (74%); white powder, mp:244-246 ºC (reported mp:242-243 ºC)[42]. ^{1}H NMR 400 MHz (DMSO-d_6) δ (ppm): 15.31 (br. s, 1H, COOH), 8.96 (s, 1H, H-2), 7.95 (d, $J_{H\text{-}F}$ = 12.9 Hz, 1H, H-5), 7.57-7.49 (m, 4H, Ar-H), 7.22 (d, $J_{H\text{-}F}$ = 6.6 Hz, 1H, H-8), 4.59 (q, J = 4.8, 9.5 Hz, 2H, CH_3-$\underline{CH_2}$), 3.88-3.36 (br. m, 8H, 8H of piperazine ring), 1.42 (t, J = 5.1 Hz, 3H, CH_2-$\underline{CH_3}$).

7-(4-phenylsulphonylpiperazin-1-yl)-1-ethyl-6-fluoro-1,4-dihydro-4-oxoquinoline-3-carboxylic acid 7d.

Yield= 3.85 g (89%); white powder, mp:290-292 ºC (no reported mp)[44]. ^{1}H NMR 400 MHz (DMSO-d_6) δ (ppm): 15.2 (s, 1H, COOH), 8.94 (s, 1H, H-2), 7.89 (d, $J_{H\text{-}F}$ =12.9 Hz,1H, H-5),

7.66-7.82 (m, 5H, Ar-H), 7.19 (d, $J_{H\text{-}F}$ = 6.6 Hz ,1H, H-8), 4.57 (q, J = 7.2 Hz, 2H, CH_3-$\underline{CH_2}$), 3.4 (br. m, 4H, 4H of piperazine near quinolone ring), 3.1 (br. m, 4H, 4H of piperazine near sulphonyl group) ,1.37 (t, J = 5.1 Hz, 3H, CH_2-$\underline{CH_3}$). **^{13}C NMR** 100 MHz (DMSO-d6): 176.3, 166.2, 151.8, 148.8, 144.8, 137.1, 134.8, 133.5, 129.4, 127.8, 120.1, 111.4, 107.4, 106.7, 67.0, 49.2, 45.9, 14.8.

7-(4-(4-tolylsulphonyl)piperazin-1-yl)-1-ethyl-6-fluoro-1,4-dihydro-4-oxoquinoline-3-carboxylic acid 7e.

Yield= 4 g (90%); white powder, mp:277-279 °C (no reported mp)[44]. ^{1}H NMR 400 MHz (DMSO-d_6) δ (ppm): 15.24 (s, 1H, COOH), 8.93 (s, 1H, H-2), 7.88 (d, $J_{H\text{-}F}$ =12.9 Hz,1H, H-5), 7.67 (d, J= 8 Hz , 2H, Ar-H), 7.48 (d, J= 8 Hz ,2H, Ar-H), 7.18 (d, $J_{H\text{-}F}$ = 6.6 Hz ,1H, H-8), 4.56 (q, J = 7.2 Hz, 2H, CH_3-$\underline{CH_2}$), 3.39 (br. m, 4H, 4H of piperazine near quinolone ring), 3.07 (br. m, 4H, 4H of piperazine near sulphonyl group) , 2.41 (s, 3H, 3H of CH_3 of P-tolyl group), 1.37 (t, J = 5.1 Hz, 3H, CH_2-$\underline{CH_3}$).

Synthesis of phenacyl bromide derivatives (2a-d) [45].

To 3 g of acetophenone derivative (acetophenone, P-bromo acetophenon, P-methyl acetophenon and P-nitro acetophenon) one equivalent of *N*-bromosuccinimide and 0.3 equivalents of P-toluenesulphonic acid were added in acetonitrile and reflux was done for 4 hours. When the reaction was completed, the reaction mixture was added to ice water and the formed precipitate was filtered, washed with water, and dried. Compounds were confirmed by their melting points as reported (2a [46], 2c [46, 47], 2b [48], 2d [49]).

2-bromo-1-phenylethanone(2a)

Yield= 2.93 g (59%); white powder, mp:79-81 °C (reported mp: 80-82 °C) [46].

2-bromo-1-(4-bromophenyl) ethanone (2b)

Yield= 3 g (72%); white powder, mp:106-108 °C (reported mp: 107-110 °C) [47, 48].

2-bromo-1-p-tolylethanone (2c)

Yield= 2.96g (62%); white powder, mp:82-84 °C (reported mp: 84-86 °C) [46].

2-bromo-1-(4-nitrophenyl) ethanone (2d)

Yield= 3.32 g (75%); white powder, mp:91-93 °C (reported mp: 91-92 °C) [49].

Synthesis of *N*-alkyl and phenacyl norfloxacin derivatives [50].

To a solution of norfloxacin (3g, 9.39 mmol) in acetonitrile (20 mL), triethylamine (1.9 ml, 14.08 mmol) was added while stirring for 10 minutes. The respective alkyl or phenacyl bromide (11.26 mmol) and potassium iodide (0.083 g, 0.5 mmol) were added, and the mixture was heated to 60-80 °C for 6-12 hours. When the reaction was completed (reaction was monitored by TLC using DCM/methanol with a ratio of 0.3:9.7 as a mobile phase), the reaction mixture was cooled to room temperature and the precipitated product was filtered and recrystallized from DMF/water.

1-ethyl-6-fluoro-1,4-dihydro-7-(4-methylpiperazin-1-yl)-4-oxoquinoline-3-carboxylic acid 10a.

Yield= 2.65 g (84%); white powder, mp:270-272 ºC (reported mp: 272-274 ºC) [51]. 1H NMR 400 MHz (DMSO-d6) δ (ppm): 15.25 (br. s, 1H, COOH), 8.95 (s, 1H, H-2), 7.92 (d, $J_{H\text{-}F}$ = 12.9 Hz, 1H, H-5), 7.17 (d, $J_{H\text{-}F}$ = 6.6 Hz, 1H, H-8), 4.6 (q, *J* = 4.8, 9.5 Hz, 2H, CH_3-<u>CH_2</u>), 3.23-2.89 (m, 4H, 4H of piperazine near quinolone ring), 2.53 (m, 4H, 4H of piperazine near methyl group), 2.26 (s, 3H, *N*-CH3), 1.42 (t, *J* = 5.1 Hz, 3H, CH_2-<u>CH_3</u>).

1-ethyl-6-fluoro-1,4-dihydro-7-(4-ethylpiperazin-1-yl)-4-oxoquinoline-3-carboxylic acid 10b.

Yield= 2.1 g (64%); white powder, mp:251-253 ºC (reported mp:251-253 ºC) [51]. ^{1}H NMR 400 MHz (DMSO-d6) δ (ppm): 15.35 (br. s, 1H, COOH), 8.95 (s, 1H, H-2), 7.92 (d, $J_{H\text{-}F}$ = 12.9 Hz, H-5), 7.17 (d, $J_{H\text{-}F}$ = 6.6 Hz, 1H, H-8), 4.58 (q, *J* = 4.8, 9.5 Hz, 2H, CH_3-<u>CH_2</u>), 3.35 (m, 4H, 4H of piperazine near quinolone ring), 2.57 (m, 4H, 4H of piperazine near ethyl group), 2.41 (q, *J* = 6.8 Hz, 2H, 2H, CH_2 of N-ethyl group), 1.41 (t, *J* = 5.1 Hz, 3H, CH_2-<u>CH_3</u>), 1.04 (t, *J* = 6.8 Hz, 3H, CH_3 of N-ethyl group).

1-ethyl-6-fluoro-1,4-dihydro-7-(4-allylpiperazin-1-yl)-4-oxoquinoline-3-carboxylic acid 10c.

Yield= 2.5 g (74%); white powder, mp:240-242 ºC (reported mp:237-238 ºC) [50]. ^{1}H NMR 400 MHz (DMSO-d6) δ (ppm): 15.4 (br. s, 1H, COOH), 8.94 (s, 1H, H-2), 7.92 (d, $J_{\text{H-F}}$ = 12.9 Hz, 1H, H-5), 7.17 (d, $J_{H\text{-}F}$ = 6.6 Hz, 1H, H-8), 5.85 (m, 1H, <u>CH</u>=CH_2), 5.19 (m, 2H, <u>CH_2</u>=CH), 4.58 (q, *J* = 4.8, 9.5 Hz, 2H, CH_3-<u>CH_2</u>), 3.44 (m, 4H, 4H of piperazine near quinolone ring), 3.03 (d, *J* = 6 Hz, 2H, CH_2 of allylic carbon), 2.57 (m, 4H, 4H of piperazine near ethyl group), 1.41 (t, *J* = 5.1 Hz, 3H, CH_2-<u>CH_3</u>).

1-ethyl-6-fluoro-1,4-dihydro-7-(4-pentylpiperazin-1-yl)-4-oxoquinoline-3-carboxylic acid 10d.

Yield= 2.61 g (71%); white powder, mp:216-218 ºC (no reported mp) [52]. ^{1}H NMR 400 MHz (DMSO-d$_6$) δ (ppm): 15.25 (br. s, 1H, COOH), 8.93 (s, 1H, H-2), 7.91 (d, $J_{H\text{-}F}$= 12.9 Hz, 1H, H-5), 7.16 (d, $J_{H\text{-}F}$= 6.6 Hz, 1H, H-8), 4.58 (q, J = 4.8, 9.5 Hz, 2H, CH$_3$-$\underline{CH_2}$), 3.43 (m, 4H, 4H of piperazine near quinolone ring), 2.88 (m, 4H, 4H of piperazine near ethyl group), 1.55-1.32 (m, 7H, first 4H of amyl chain near piperazine ring and 3H of CH$_2$-$\underline{CH_3}$), 1.03-0.83 (m, 7H, rest 7H of amyl chain).

1-ethyl-6-fluoro-1,4-dihydro-7-(4-benzylpiperazin-1-yl)-4-oxoquinoline-3-carboxylic acid 10e.

Yield= 2.7 g (70%); white powder, mp:216-218 ºC(reported mp: 214 ºC) [51]. ^{1}H NMR 400 MHz (DMSO-d$_6$) δ (ppm): 15.29 (br. s, 1H, COOH), 8.94 (s, 1H, H-2), 7.89 (d, $J_{H\text{-}F}$= 12.9 Hz, 1H, H-5), 7.36-7.25 (m, 5H, Ar-H), 7.16 (d, $J_{H\text{-}F}$= 6.6 Hz, 1H, H-8), 4.56 (q, J = 4.8, 9.5 Hz, 2H, CH$_3$-$\underline{CH_2}$), 3.57 (s, 2H, -CH$_2$ of benzyl), 3.31 (br. m, 4H, 4H of piperazine near quinolone ring), 2.57 (br. m, 4H, 4H of piperazine near benzyl moiety), 1.41 (t, J = 5.1 Hz, 3H, CH$_2$-$\underline{CH_3}$).

1-ethyl-6-fluoro-1,4-dihydro-7-(4-(4-chlorobenzyl)piperazin-1-yl)-4-oxoquinoline-3-carboxylic acid 10f.

Yield= 2.65 g (61%); white powder, mp:248-250 ºC. ^{1}H NMR 400 MHz (DMSO-d$_6$) δ (ppm): 15.15 (br. s, 1H, COOH), 8.87 (s, 1H, H-2), 7.83 (d, $J_{H\text{-}F}$= 12.9 Hz, 1H, H-5), 7.34-7.28 (m, 4H, Ar-H), 7.09 (d, $J_{H\text{-}F}$= 6.6 Hz, 1H, H-8), 4.5 (q, J = 4.8, 9.5 Hz, 2H, CH$_3$-$\underline{CH_2}$), 3.48 (s, 2H, -CH2 of p-Clbenzyl), 3.34 (br. m, 4H, 4H of piperazine near quinolone ring), 2.5 (br. m, 4H, 4H of piperazine near p-Clbenzyl moiety), 1.32 (t, J = 5.1 Hz, 3H, CH$_2$-$\underline{CH_3}$). ^{13}C NMR 100 MHz (DMSO-d$_6$): 176.6, 166.2, 154.0, 151.5, 148.8, 145.5, 137.4, 131.5, 130.8, 128.1, 119.4, 111.4, 107.4, 106.0, 60.9, 52.1, 49.6, 49.1, 14.3. Anal. Calcd for $C_{23}H_{23}ClFN_3O_3$: C, 62.23; H, 5.22; N, 9.47. Found: C, 62.47; H, 5.38; N, 9.70.

1-ethyl-6-fluoro-1,4-dihydro-7-(4-phenacylpiperazin-1-yl)-4-oxoquinoline-3-carboxylic acid 12a.

Yield= 3.6 g (87%); white powder, mp:220-222 ºC (reported mp:208-210 ºC) [50, 53]. ^{1}H NMR 400 MHz (DMSO-d$_6$) δ (ppm): 15.35 (br. s, 1H, COOH), 8.94 (s, 1H, H-2), 8.03 (d, J= 8 Hz, 2H, H-2' and H-6', Ar-H), 7.92 (d, $J_{H\text{-}F}$= 12.9 Hz, 1H, H-5), 7.61 (m, 3H, H-3',4' and 5', Ar-H), 7.19 (d, $J_{H\text{-}F}$= 6.6 Hz, 1H, H-8), 4.59 (q, J = 4.8, 9.5 Hz, 2H, CH$_3$-$\underline{CH_2}$), 3.97 (s, 2H, CH$_2$ of phenacyl), 3.24 (br. m, 4H, 4H of piperazine near quinolone ring), 2.76 (br. m, 4H), 1.41 (t, J = 5.1 Hz, 3H, CH$_2$-$\underline{CH_3}$).

1-ethyl-6-fluoro-1,4-dihydro-7-(4-(4-bromophenacyl)piperazin-1-yl)-4-oxoquinoline-3-carboxylic acid 12b.

Yield= 3.93 g (81%); white powder, mp:251-253 °C (reported mp:249-251 °C) [50, 53]. ^{1}H NMR 400 MHz (DMSO-d$_6$) δ (ppm): 15.37 (br. s, 1H, COOH), 8.96 (s, 1H, H-2), 7.96 (m, 3H, H-3',5', Ar-H and 1H of H-5), 7.74 (d, *J*= 8.4 Hz, 2H, 2H of H-2' and 6', Ar-H), 7.18 (d, $J_{H\text{-}F}$ = 6.6 Hz, 1H, H-8), 4.6 (q, *J* = 4.8, 9.5 Hz, 2H, CH_3-<u>CH_2</u>), 3.94 (s, 2H, CH_2 of phenacyl), 3.41 (br. m, 4H, 4H of piperazine near quinolone ring), 3.32 (br. m, 4H, 4H of piperazine near p-Bromophenacyl moiety), 1.41 (t, *J* = 5.1 Hz, 3H, CH_2-<u>CH_3</u>).

1-ethyl-6-fluoro-1,4-dihydro-7-(4-(4-methylphenacyl)piperazin-1-yl)-4-oxoquinoline-3-carboxylic acid 12c.

Yield= 3.48 g (82%); white powder, mp:210-212 °C (reported mp:206-208 °C) [50]. ^{1}H NMR 400 MHz (DMSO-d$_6$) δ (ppm): 15.35 (br. s, 1H, COOH), 8.95 (s, 1H, H-2), 7.92 (m, 3H, 2H of H-2',6', Ar-H and 1H of H-5), 7.33 (d, *J*= 8.4 Hz, 2H of H-3',5', Ar-H), 7.19 (d, $J_{H\text{-}F}$ = 6.6 Hz, 1H, H-8), 4.59 (q, *J* = 4.8, 9.5 Hz, 2H, CH_3-<u>CH_2</u>), 3.92 (s, 2H, CH_2 of phenacyl), 3.34 (br. m, 4H, 4H of piperazine near quinolone ring), 2.74 (br. m, 4H, 4H of piperazine near p-Methylphenacyl moiety), 2.38 (s, 3H, CH_3 of P-me phenacyl), 1.41 (t, *J* = 5.1 Hz, 3H, CH_2-<u>CH_3</u>).

1-ethyl-6-fluoro-1,4-dihydro-7-(4-(4-nitrophenacyl)piperazin-1-yl)-4-oxoquinoline-3-carboxylic acid 12d.

Yield= 3.64 g (80%); yellow powder, mp:242-244 °C. ^{1}H NMR 400 MHz (DMSO-d$_6$) δ (ppm): 15.38 (br. s, 1H, COOH), 8.96 (s, 1H, H-2), 8.35 (d, *J* = 8.7 Hz, 2H, 2H of H-3' and 5' , Ar-H), 8.25 (d, *J* = 8.7 Hz, 2H, 2H of H-2' and 6', Ar-H), 7.94 (d, $J_{H\text{-}F}$ = 12.9 Hz, 1H, H-5), 7.21 (d, $J_{H\text{-}F}$ = 6.6 Hz, 1H, H-8), 4.6 (q, *J* = 4.8, 9.5 Hz, 2H, CH_3-<u>CH_2</u>), 4.04 (s, 2H, CH_2 of phenacyl), 3.48 (br. m, 4H, 4H of piperazine near quinolone ring), 3.29 (br. m, 4H, 4H of piperazine near p-Nitrophenacyl moiety), 1.41 (t, *J* = 5.1 Hz, 3H, CH_2-<u>CH_3</u>). ^{13}C NMR 100 MHz (DMSO-d$_6$): 185.0, 176.3, 166.2, 151.5, 148.5, 145.5, 137.1, 131.5, 130.8, 128.1, 119.4, 111.4, 107.4, 106.0, 60.9, 52.1, 49.5, 49.1, 14.8. Anal. Calcd for $C_{24}H_{23}FN_4O_6$: C, 59.75; H, 4.81; N, 11.61. Found: C, 59.91; H, 5.05; N, 11.89.

Synthesis of *N*-substituted piperazinylnorfloxacin hydroxamic acid derivatives.

To a cooled stirred suspension of *N*-substituted piperazinyl norfloxacin derivative (1g) in dichloromethane (30 mL), triethylamine (2 equivalents) and ethyl chloroformate (1.5 equivalents)

were added. The reaction mixture was stirred for 1 hour in an ice bath. Then, hydroxylamine hydrochloride (2 equivalents) was added and stirring continued at room temperature for 6-8 hours. The reaction progress was monitored by TLC ($CHCl_3/CH_3OH$: 9.7/0.3). After total consumption of the reactants, the organic layer was washed with saturated brine solution (2 x 25 mL) and distilled water (2 x 25 mL), dried over anhydrous Na_2SO_4, filtered and evaporated under reduced pressure. The obtained solid was recrystallized from methanol to afford the hydroxamic acid derivatives.

7-(4-acetylpiperazin-1-yl)-1-ethyl-6-fluoro-1,4-dihydro-*N*-hydroxy-4-oxoquinoline-3-carboxamide 5a.

Yield= 0.55 g (53%); beige powder, mp:244-246 °C (no reported mp) [54]. ^{1}H NMR 400 MHz (DMSO-d_6) δ (ppm): 11.74 (br. s, 1H, NH of hydroxamic), 9.15 (br. s, 1H, OH of hydroxamic), 8.78 (s, 1H, H-2), 7.89 (d, $J_{H\text{-}F}$ = 12.9 Hz, 1H, H-5), 7.12 (d, $J_{H\text{-}F}$ = 6.6 Hz, 1H, H-8), 4.51 (q, J = 4.8, 9.5 Hz, 2H, CH_3-$\underline{CH_2}$), 3.64 (br. m, 4H, 4 H of piperazine near quinolone ring), 3.25 (br. m, 4H, 4H of piperazine near acetyl), 2.06 (s, 3H, CH_3 of acetyl group), 1.39 (t, J = 5.1 Hz, 3H, CH_2-$\underline{CH_3}$). ^{13}C NMR (DMSO-d_6): 173.4, 168.6, 162.5, 151.3, 146.9, 144.5, 136.5, 121.4, 111.8, 110.1, 106.1, 49.7, 48.5, 45.4, 21.1, 14.5. Anal. Calcd for $C_{18}H_{21}FN_4O_4$: C, 57.44; H, 5.62; N, 14.89. Found: C, 57.63; H, 5.88; N, 15.11.

7-(4-(2-chloroacetyl)piperazin-1-yl)-1-ethyl-6-fluoro-1,4-dihydro-*N*-hydroxy-4-oxoquinoline-3-carboxamide 5b.

Yield= 0.73 g (70%); beige powder, mp:270-272 °C. IR (KBr): 3430(NH str), 3190(OH str), 3049(aromatic C-H str), 2849(aliphatic C-H str), 1683(hydroxamic C=O str), 1665(carbamidic C=O str), 1632(quinolone C=O str), 1241(C-O) cm^{-1}. ^{1}H NMR 400 MHz (DMSO-d_6) δ (ppm): 11.74 (br. s, 1H, NH of hydroxamic), 9.19 (br. s, 1H, OH of hydroxamic), 8.77 (s, 1H, H-2), 7.88 (d, $J_{H\text{-}F}$ = 12.9 Hz, 1H, H-5), 7.12 (d, $J_{H\text{-}F}$ = 6.6 Hz, 1H, H-8), 4.51 (q, J = 4.8, 9.5 Hz, 2H, CH_3-$\underline{CH_2}$), 3.85-3.64 (br. m, 6H, 2H of -CH_2Cl and 4 H of piperazine near quinolone ring), 3.27 (br. m, 4H, 4H of piperazine near chloroacetyl), 1.39 (t, J = 5.1 Hz, 3H, CH_2-$\underline{CH_3}$). ^{13}C NMR 100 MHz (DMSO-d_6): 173.3, 166.9, 162.5, 152.5, 147.2, 146.8, 144.5, 136.5, 121.4, 111.8, 110.1, 106.4, 49.4, 48.5, 45.6, 41.0, 14.1. Anal. Calcd for $C_{18}H_{20}ClFN_4O_4$: C, 52.62; H, 4.91; N, 13.64. Found: C, 52.88; H, 5.04; N, 13.70.

7-(4-benzoyl)piperazin-1-yl)-1-ethyl-6-fluoro-1,4-dihydro-*N*-hydroxy-4-oxoquinoline-3-carboxamide 8a.

Yield= 0.82 g (79%); white powder, mp:253-255 ºC. IR (KBr): 3438(NH str), 3192(OH str), 3046(aromatic C-H str), 2987(aliphatic C-H str), 1678(hydroxamic C=O str), 1644(carbamidic C=O str), 1607(quinolone C=O str), 1249(C-O), 1029(C-N) cm^{-1}. ^1H NMR 400 MHz (DMSO-d_6) δ (ppm): 11.75 (s, 1H, NH of hydroxamic),9.19 (s, 1H, OH of hydroxamic), 8.79 (s, 1H, H-2), 7.9 (d, J_{H-F}=12.9 Hz,1H, H-5), 7.45-7.51 (m, 5H, Ar-H), 7.15 (d, J_{H-F}= 6.6 Hz ,1H, H-8),4.52 (q, *J* = 7.2 Hz, 2H, CH_3-$\underline{CH_2}$), 3.7 (br. m, 4H, 4H of piperazine near quinolone ring), 3.3 (br. m, 4H, 4H of piperazine near carbonyl of benzoyl), 1.39 (t, *J* = 5.1 Hz, 3H, CH_2-$\underline{CH_3}$). ^{13}C NMR 100 MHz (DMSO-d_6): 173.8, 169.7, 163.0, 152.0, 147.3, 145.3, 136.9, 135.9, 130.6, 129.3, 128.0, 121.9, 112.2, 110.5, 106.5, 50.3, 49.6, 47.7, 14.9. Anal. Calcd for $C_{23}H_{23}FN_4O_4$: C, 63.00; H, 5.29; N, 12.78. Found: C, 62.86; H, 5.40; N, 13.02. LRMS for $[C_{24}H_{23}FN_4O_4]^+$ $[M]^+$ calculated: 438.17 found: 438.13.

7-(4-(4-methoxybenzoyl)piperazin-1-yl)-1-ethyl-6-fluoro-1,4-dihydro-*N*-hydroxy-4-oxoquinoline-3-carboxamide 8b.

Yield= .78 g (75%); white powder, mp:197-199 ºC. IR (KBr): 3427(NH str), 3186(OH str), 3042(aromatic C-H str), 2983(aliphatic C-H str), 1683(hydroxamic C=O str), 1637(carbamidic C=O str), 1601(quinolone C=O str), 1242(C-O), 1023(C-N) cm^{-1}. ^1H NMR 400 MHz (DMSO-d_6) δ (ppm): 11.73 (s, 1H, NH of hydroxamic), 9.19 (s,1H, OH of hydroxamic), 8.77 (s, 1H, H-2), 7.89 (d, J_{H-F}=12.9 Hz,1H, H-5), 7.43 (d, *J*=8.4 Hz, 2H, 2H of H-2' and 6', Ar-H), 7.14 (d, J_{H-F}= 6.6 Hz ,1H, H-8), 7.01 (d, *J*=8.8 Hz, 2H, 2H of H-3' and 5', Ar-H), 4.51 (q, *J* = 7.2 Hz ,2H, CH_3-$\underline{CH_2}$), 3.8 (s, 3H, OCH_3), 3.7 (br. m, 4H, 4H of piperazine near quinolone ring), 3.29 (br. m, 4H, 4H of piperazine near carbonyl of p-Methoxybenzoyl), 1.38 (t, *J* = 5.1 Hz, 3H, CH_2-$\underline{CH_3}$). ^{13}C NMR 100 MHz (DMSO-d_6): 173.6, 169.2, 162.9, 160.6, 151.5, 146.9, 144.5, 136.5, 129.5, 127.4, 121.4, 114.1, 111.8, 110.4, 106.4, 55.6, 49.9, 48.5, 45.5, 14.4. Anal. Calcd for $C_{24}H_{25}FN_4O_5$: C, 61.53; H, 5.38; N, 11.96. Found: C, 61.75; H, 5.46; N, 12.05.

7-(4-(4-chlorobenzoyl)piperazin-1-yl)-1-ethyl-6-fluoro-1,4-dihydro-*N*-hydroxy-4-oxoquinoline-3-carboxamide 8c.

Yield= .76 g (74%); white powder, mp:225-227 ºC. IR (KBr): 3432(NH str), 3196(OH str), 3054(aromatic C-H str), 2991(aliphatic C-H str), 1685(hydroxamic C=O str), 1661(carbamidic C=O str), 1634(quinolone C=O str), 1245(C-O), 1126(C-N) cm^{-1}. ^1H NMR 400 MHz (DMSO-d_6)

δ (ppm): 11.73 (s, 1H, NH of hydroxamic), 9.18 (s, 1H, OH of hydroxamic), 8.78 (s, 1H, H-2), 7.89 (d, $J_{H\text{-}F}$ =12.9 Hz, 1H, H-5), 7.57-7.48 (m, 4H, Ar-H), 7.14 (d, $J_{H\text{–}F}$ = 6.6 Hz ,1H, H-8) ,4.51 (q, *J* = 7.2 Hz ,2H, CH_3-<u>CH_2</u>), 3.68 (br. m, 4H, 4H of piperazine near quinolone ring), 3.28 (br. m, 4H, 4H of piperazine near carbonyl of p-Clbenzoyl), 1.38 (t, *J* = 5.1 Hz, 3H, CH_2-<u>CH_3</u>). ^{13}C NMR 100 MHz (DMSO-d_6): 168.7, 166.4, 163.0, 154.3, 147.6, 145.0, 140.6, 136.9, 135.3, 129.9, 128.9, 121.5, 110.9, 106.9, 104.9, 50.1, 48.9, 46.5, 14.9. Anal. Calcd for $C_{23}H_{22}ClFN_4O_4$: C, 58.42; H, 4.69; N, 11.85. Found: C, 58.68; H, 4.87; N, 12.01.

7-(4-phenylsulphonylpiperazin-1-yl)-1-ethyl-6-fluoro-1,4-dihydro-*N*-hydroxy-4-oxoquinoline-3-carboxamide 8d.

Yield= .85 g (82%); white powder, mp:282-284 ºC. ^{1}H NMR 400 MHz (DMSO-d_6) δ (ppm): 11.7 (br. s, 1H, NH of hydroxamic), 9.16 (br. s, 1H, OH of hydroxamic), 8.77 (s, 1H, H-2), 7.86 (d, $J_{H\text{-}F}$=12.9 Hz,1H, H-5), 7.67-7.82 (m, 5H, Ar-H), 7.13 (d, $J_{H\text{–}F}$ = 6.6 Hz, 1H, H-8), 4.5 (q, *J* = 7.2 Hz, 2H, CH_3-<u>CH_2</u>), 3.35 (br. m, 4H, 4H of piperazine near quinolone ring), 3.09 (br. m, 4H, 4H of piperazine near sulphonyl group) ,1.35 (t, *J* = 5.1 Hz, 3H, CH_2-<u>CH_3</u>). ^{13}C NMR 100 MHz (DMSO-d_6): 173.8, 162.8, 151.8, 147.3, 144.8, 137.0, 136.1, 130.2, 128.7, 127.5, 121.6, 111.9, 110.6, 106.6, 50.2, 48.9, 47.4, 14.9. Anal. Calcd for $C_{22}H_{23}FN_4O_5S$: C, 55.69; H, 4.89; N, 11.81; S, 6.76. Found: C, 55.94; H, 4.96; N, 12.09; S, 6.89.

7-(4-(4-tolylsulphonyl)piperazin-1-yl)-1-ethyl-6-fluoro-1,4-dihydro-*N*-hydroxy-4-oxoquinoline-3-carboxamide 8e.

Yield= .82 g (79%); white powder, mp:271-273 ºC. ^{1}H NMR 400 MHz (DMSO-d_6) δ (ppm): 11.7 (s, 1H, NH of hydroxamic), 9.16 (br. s, 1H, OH of hydroxamic), 8.76 (s, 1H, H-2), 7.85 (d, $J_{H\text{-}F}$ =12.9 Hz,1H, H-5), 7.67 (d, *J*= 8 Hz, 2H, H-2' and 6', Ar-H Ar-H), 7.48 (d, *J*= 8 Hz, 2H, H-3' and 5', Ar-H), 7.11 (d, $J_{H\text{–}F}$ = 6.6 Hz, 1H, H-8), 4.49 (q, *J* = 7.2 Hz ,2H, CH_3-<u>CH_2</u>), 3.34 (br. m, 4H, 4H of piperazine near quinolone ring), 3.06 (br. m, 4H, 4H of piperazine near sulphonyl group), 2.41 (s, 3H, CH_3 of P-tolyl group), 1.35 (t, *J* = 5.1 Hz, 3H, CH_2-<u>CH_3</u>). ^{13}C NMR 100 MHz (DMSO-d_6): 173.3, 162.5, 153.5, 151.2, 146.8, 143.8, 136.2, 131.8, 130.1, 128.1, 121.4, 112.1, 110.0, 106.4, 54.9, 49.2, 45.5, 21.1, 14.5. Anal. Calcd for $C_{23}H_{25}FN_4O_5S$: C, 56.55; H, 5.16; N, 11.47; S, 6.56. Found: C, 56.76; H, 5.28; N, 11.71; S, 6.78.

1-ethyl-6-fluoro-1,4-dihydro-*N*-hydroxy-7-(4-methylpiperazin-1-yl)-4-oxoquinoline-3-carboxamide 11a.

Yield= 0.72 g (69%); white powder, mp:150-152 ºC (no reported mp) [54]. ^{1}H NMR 400 MHz (DMSO-d$_6$) δ (ppm): 11.77 (br. s, 1H, NH of hydroxamic), 9.19 (br. s, 1H, OH of hydroxamic), 8.8 (s, 1H, H-2), 7.91 (d, $J_{H\text{-}F}$ = 12.9 Hz, 1H, H-5), 7.15 (d, $J_{H\text{-}F}$ = 6.6 Hz, 1H, H-8), 4.54 (q, *J* = 4.8, 9.5 Hz, 2H, CH$_3$-<u>CH$_2$</u>), 3.3-2.93 (m, 8H, 8H of piperazine ring), 2.71 (s, 3H, *N*-CH$_3$), 1.42 (t, *J* = 5.1 Hz, 3H, CH$_2$-<u>CH$_3$</u>). ^{13}C NMR (DMSO-d$_6$): 173.8, 163.0, 152.3, 147.3, 142.0, 136.9, 122.2, 112.2, 110.5, 106.9, 52.4, 48.9, 47.0, 42.7, 14.9. Anal. Calcd for $C_{17}H_{21}FN_4O_3$: C, 58.61; H, 6.08; N, 16.08. Found: C, 58.83; H, 6.12; N, 16.31.

1-ethyl-6-fluoro-1,4-dihydro-*N*-hydroxy-7-(4-ethylpiperazin-1-yl)-4-oxoquinoline-3-carboxamide 11b.

Yield= .66 g (63%); white powder, mp:244-246 ºC. ^{1}H NMR 400 MHz (DMSO-d$_6$) δ (ppm): 11.74 (br. s, 1H, NH of hydroxamic), 9.18 (br. s, 1H, OH of hydroxamic), 8.76 (s, 1H, H-2), 7.87 (d, $J_{H\text{-}F}$ = 12.9 Hz, 1H, H-5), 7.09 (d, $J_{H\text{-}F}$ = 6.6 Hz, 1H, H-8), 4.5 (q, *J* = 4.8, 9.5 Hz, 2H, CH$_3$-<u>CH$_2$</u>), 3.27 (m, 4H, 4H of piperazine near quinolone ring), 2.59 (m, 4H, 4H of piperazine near ethyl group), 2.42 (q, *J* = 6.8 Hz, 2H, CH$_2$ of *N*-ethyl group), 1.38 (t, *J* = 5.1 Hz, 3H, CH$_2$-<u>CH$_3$</u>), 1.04 (t, *J* = 6.8 Hz, 3H, CH$_3$ of *N*-ethyl group). ^{13}C NMR 100 MHz (DMSO-d$_6$): 173.5, 163.0, 154.2, 151.6, 146.9, 136.5, 121.2, 111.8, 110.5, 105.8, 52.1, 51.6, 49.5, 48.5, 14.4, 11.9. Anal. Calcd for $C_{18}H_{23}FN_4O_3$: C, 59.66; H, 6.40; N, 15.46. Found: C, 59.92; H, 6.56; N, 15.53.

1-ethyl-6-fluoro-1,4-dihydro-*N*-hydroxy-7-(4-allylpiperazin-1-yl)-4-oxoquinoline-3-carboxamide 11c.

Yield= .74 g (71%); white powder, mp:216-218 ºC. ^{1}H NMR 400 MHz (DMSO-d$_6$) δ (ppm): 11.75 (br. s, 1H, NH of hydroxamic), 9.17 (br. s, 1H, OH of hydroxamic), 8.77 (s, 1H, H-2), 7.88 (d, $J_{H\text{-}F}$ = 12.9 Hz, 1H, H-5), 7.1 (d, $J_{H\text{-}F}$ = 6.6 Hz, 1H, H-8), 5.86 (m, 1H, <u>CH</u>=CH$_2$), 5.21 (m, 2H, <u>CH$_2$</u>=CH), 4.52 (q, *J* = 4.8, 9.5 Hz, 2H, CH$_3$-<u>CH$_2$</u>), 3.28 (m, 4H, 4H of piperazine near quinolone ring), 3.04 (d, *J* = 6 Hz, 2H, CH$_2$ of allylic carbon), 2.58 (m, 4H, 4H of piperazine near ethyl group), 1.39 (t, *J* = 5.1 Hz, 3H, CH$_2$-<u>CH$_3$</u>). ^{13}C NMR 100 MHz (DMSO-d$_6$): 173.6, 162.5, 154.1, 151.5, 146.8, 144.8, 136.5, 121.4, 118.1, 111.4, 110.0, 106.7, 60.6, 52.2, 49.9, 48.5, 14.4. Anal. Calcd for $C_{19}H_{23}FN_4O_3$: C, 60.95; H, 6.19; N, 14.96. Found: C, 60.84; H, 6.35; N, 15.12.

1-ethyl-6-fluoro-1,4-dihydro-*N*-hydroxy-7-(4-pentylpiperazin-1-yl)-4-oxoquinoline-3-carboxamide 11d.

Yield= .64 g (62%); white powder, mp: 151-153 ºC. ^{1}H NMR 400 MHz (DMSO-d$_6$) δ (ppm): 11.72 (br. s, 1H, NH of hydroxamic), 9.21 (br. s, 1H, OH of hydroxamic), 8.79 (s, 1H, H-2), 7.92

(d, J_{H-F} = 12.9 Hz, 1H, H-5), 7.19 (d, J_{H-F} = 6.6 Hz, 1H, H-8), 4.56 (q, J = 4.8, 9.5 Hz, 2H, CH_3-$\underline{CH_2}$), 3.75-3.12 (m, 8H, 8H of piperazine ring), 1.72-1.58 (m, 4H, first 4H of amyl chain near piperazine ring), 1.39 (t, J = 5.1 Hz, 3H, CH_2-$\underline{CH_3}$), 0.94-0.90 (m, 7H, rest 7H of amyl chain). ^{13}C NMR 100 MHz (DMSO-d_6): 173.8, 162.9, 151.7, 147.4, 143.6, 136.9, 122.1, 112.4, 110.6, 106.6, 54.6, 50.9, 48.1, 31.9, 26.3, 22.7, 15.0. Anal. Calcd for $C_{21}H_{29}FN_4O_3$: C, 62.36; H, 7.23; N, 13.85. Found: C, 62.50; H, 7.37; N, 14.08.

1-ethyl-6-fluoro-1,4-dihydro-*N*-hydroxy-7-(4-benzylpiperazin-1-yl)-4-oxoquinoline-3-carboxamide 11e.

Yield= .87 g (84%); white powder, mp:233-235 ºC. IR (KBr): 3423(NH str), 3186(OH str), 3059(aromatic C-H str), 2987(aliphatic C-H str), 1686(hydroxamic C=O str), 1657(carbamidic C=O str), 1632(quinolone C=O str), 1265(C-O), 1116(C-N) cm^{-1}. ^{1}H NMR 400 MHz (DMSO-d_6) δ (ppm): 11.75 (br. s, 1H, NH of hydroxamic), 9.17 (br. s, 1H, OH of hydroxamic), 8.77 (s, 1H, H-2), 7.89 (d, J_{H-F} = 12.9 Hz, 1H, H-5), 7.25-7.36 (m, 5H, Ar-H), 7.12 (d, J_{H-F} = 6.6 Hz, 1H, H-8), 4.49 (q, J = 4.8, 9.5 Hz, 2H, CH_3-$\underline{CH_2}$), 3.56 (s, 2H, -CH_2 of benzyl), 3.27 (br. m, 4H, 4H of piperazine near quinolone ring), 2.59 (br. m, 4H, 4H of piperazine near benzyl moiety), 1.39 (t, J = 5.1 Hz, 3H, CH_2-$\underline{CH_3}$). ^{13}C NMR 100 MHz (DMSO-d_6): 173.6, 162.5, 151.5, 146.8, 138.1, 136.4, 129.1, 128.1, 127.1, 121.1, 111.4, 110.0, 105.7, 61.5, 52.6, 49.6, 48.9, 14.5. Anal. Calcd for $C_{23}H_{25}FN_4O_3$: C, 65.08; H, 5.94; N, 13.20. Found: C, 65.24; H, 5.78; N, 13.46.

1-ethyl-6-fluoro-1,4-dihydro-*N*-hydroxy-7-(4-(4-chlorobenzyl)piperazin-1-yl)-4-oxoquinoline-3-carboxamide 11f.

Yield= .75 g (73%); white powder, mp:220-222 ºC. IR (KBr): 3430(NH str), 3189(OH str), 3069(aromatic C-H str), 2983(aliphatic C-H str), 1690(hydroxamic C=O str), 1654(carbamidic C=O str), 1622(quinolone C=O str), 1275(C-O), 1106(C-N) cm^{-1}. ^{1}H NMR 400 MHz (DMSO-d_6) δ (ppm): 11.75 (br. s, 1H, NH of hydroxamic), 9.17 (br. s, 1H, OH of hydroxamic), 8.77 (s, 1H, H-2), 7.87 (d, J_{H-F} = 12.9 Hz, 1H, H-5), 7.36-7.43 (m, 4H, Ar-H), 7.1 (d, J_{H-F} = 6.6 Hz, 1H, H-8), 4.51 (q, J = 4.8, 9.5 Hz, 2H, CH_3-$\underline{CH_2}$), 3.56 (s, 2H, -CH_2), 3.27 (br. m, 4H, 4H of piperazine near quinolone ring), 2.58 (br. m, 4H, 4H of piperazine near p-Clbenzyl moiety), 1.38 (t, J = 5.1 Hz, 3H, CH_2-$\underline{CH_3}$). ^{13}C NMR 100 MHz (DMSO-d_6): 173.3, 162.6, 151.6, 146.9, 144.9, 137.9, 136.5, 131.9, 130.9, 128.5, 121.2, 111.4, 110.5, 105.8, 61.0, 52.2, 49.9, 48.5, 14.5. Anal. Calcd for $C_{23}H_{24}ClFN_4O_3$: C, 60.20; H, 5.27; N, 12.21. Found: C, 60.47; H, 5.41; N, 12.48.

1-ethyl-6-fluoro-1,4-dihydro-*N*-hydroxy-7-(4-phenacylpiperazin-1-yl)-4-oxoquinoline-3-carboxamide 13a.

Yield= .73 g (71%); white powder, mp: 223-225 ºC. IR (KBr): 3420(NH str), 3165(OH str), 3055(aromatic C-H str), 2929(aliphatic C-H str), 1700(ketonic C=O str), 1681(hydroxamic C=O str), 1643(quinolone C=O str), 1257(C-O) cm^{-1}. ^{1}H NMR 400 MHz (DMSO-d_6) δ (ppm): 11.74 (br. s, 1H, NH of hydroxamic), 9.18 (br. s, 1H, OH of hydroxamic), 8.78 (s, 1H, H-2), 8.01 (d, *J*= 8 Hz, 2H, H-2' and 6', Ar-H) 7.9 (d, $J_{H\text{-}F}$ = 12.9 Hz, 1H, H-5), 7.63 (m, 3H, 3H, H-3',4' and 5', Ar-H), 7.15 (d, $J_{H\text{-}F}$ = 6.6 Hz, 1H, H-8), 4.53 (q, *J* = 4.8, 9.5 Hz, 2H, CH_3-$\underline{CH_2}$), 3.48 (br. m, 6H, 4H of piperazine near quinolone ring and 2H of CH_2 of phenacyl), 2.9 (br. m, 4H, 4H of piperazine near phenacyl moiety), 1.39 (t, *J* = 5.1 Hz, 3H, CH_2-$\underline{CH_3}$). ^{13}C NMR 100 MHz (DMSO-d_6): 173.5, 162.5, 146.8, 136.5, 133.5, 129.4, 128.8, 128.5, 128.1, 121.1, 111.8, 110.5, 105,8, 89.1, 52.6, 48.5, 42.9, 14.5. Anal. Calcd for $C_{24}H_{25}FN_4O_4$: C, 63.71; H, 5.57; N, 12.38. Found: C, 63.52; H, 5.73; N, 12.46.

1-ethyl-6-fluoro-1,4-dihydro-*N*-hydroxy-7-(4-(4-bromophenacyl)piperazin-1-yl)-4-oxoquinoline-3-carboxamide 13b.

Yield= .8 g (78%); white powder, mp:233-235 ºC. ^{1}H NMR 400 MHz (DMSO-d_6) δ (ppm): 11.75 (br. s, 1H, NH of hydroxamic), 9.19 (br. s, 1H, OH of hydroxamic), 8.77 (s, 1H, H-2), 7.96 (d, *J* = 8.6 Hz, 2H, H-2' and 6', Ar-H), 7.88 (d, $J_{H\text{-}F}$ = 12.9 Hz, 1H, H-5), 7.75 (d, *J* = 8.6 Hz, 2H, H-3' and 5', Ar-H), 7.12 (d, $J_{H\text{-}F}$ = 6.6 Hz, 1H, H-8), 4.52 (q, J = 4.8, 9.5 Hz, 2H, CH_3-$\underline{CH_2}$), 3.95 (s, 2H, CH_2 of phenacyl), 3.28 (m, 4H, 4H of piperazine near quinolone ring), 2.75 (m, 4H, 4H of piperazine near p-Bromophenacyl moiety), 1.39 (t, *J* = 5.1 Hz, 3H, CH_2-$\underline{CH_3}$). ^{13}C NMR 100 MHz (DMSO-d_6): 177.7, 173.9, 163.0, 151.5, 147.3, 145.3, 136.9, 135.3, 132.3, 130.6, 127.9, 111.9, 110.9, 106.2, 64.0, 52.8, 50.0, 48.7, 15.2. Anal. Calcd for $C_{24}H_{24}BrFN_4O_4$: C, 54.25; H, 4.55; N, 10.54. Found: C, 54.51; H, 4.68; N, 10.79.

1-ethyl-6-fluoro-1,4-dihydro-*N*-hydroxy-7-(4-(4-methylphenacyl)piperazin-1-yl)-4-oxoquinoline-3-carboxamide 13c.

Yield= .69 g (67%); white powder, mp:166-168 ºC. IR (KBr): 3426(NH str), 3172(OH str), 3061(aromatic C-H str), 2925(aliphatic C-H str), 1712(ketonic C=O str), 1683(hydroxamic C=O str), 1647(quinolone C=O str), 1253(C-O) cm^{-1}. ^{1}H NMR 400 MHz (DMSO-d_6) δ (ppm): 11.72 (br. s, 1H, NH of hydroxamic), 10.11 (br. s, 1H, OH of hydroxamic), 8.8 (s, 1H, H-2), 7.95-7.9 ppm (m, 3H, 2H of H-2' and 6', Ar-H and 1H of H-5), 7.44 (d, *J* = 8 Hz, 2H, H-3' and 5', Ar-H),

7.22 (d, $J_{H\text{-}F}$ = 6.6 Hz, 1H, H-8), 5.17 (s, 2H, CH_2 of p-Methylphenacyl), 4.56 (q, J = 4.8, 9.5 Hz, 2H, CH_3-$\underline{CH_2}$), 3.77-3.50 (br. m, 8H, 8H of piperazine ring), 2.42 (s, 3H, CH_3 of P-me phenacyl), 1.41 (t, J = 5.1 Hz, 3H, CH_2-$\underline{CH_3}$). ^{13}C NMR 100 MHz (DMSO-d_6): 191.4, 173.9, 162.9, 153.3, 147.4, 146.2, 143.4, 136.9, 130.1, 129.8, 128.8, 126.8, 112.3, 110.9, 106.9, 66.7, 52.4, 49.0, 43.0, 21.6, 14.9. Anal. Calcd for $C_{25}H_{27}FN_4O_4$: C, 64.37; H, 5.83; N, 12.01. Found: C, 64.25; H, 5.95; N, 12.37.

1-ethyl-6-fluoro-1,4-dihydro-*N*-hydroxy-7-(4-(4-nitrophenacyl)piperazin-1-yl)-4-oxoquinoline-3-carboxamide 13d.

Yield= .72 g (70%); yellow powder, mp: 224-226 ºC. IR (KBr): 3418(NH str), 3177(OH str), 3064(aromatic C-H str), 2921(aliphatic C-H str), 1715(ketonic C=O str), 1685(hydroxamic C=O str), 1646(quinolone C=O str), 1472(N=O str), 1259(C-O) cm^{-1}. ^{1}H NMR 400 MHz (DMSO-d_6) δ (ppm): 11.74 (s, 1H, NH of hydroxamic), 9.18 (s, 1H, OH of hydroxamic), 8.79 (s, 1H, H-2), 8.38 (d, J = 7.8 Hz, 2H, H-3' and 5', Ar-H), 8.25 (d, J = 7.8 Hz, 2H, H-2' and 6', Ar-H), 7.89 (d, $J_{H\text{-}F}$ = 12.9 Hz, 1H, H-5), 7.14 (d, $J_{H\text{-}F}$ = 6.6 Hz, 1H, H-8), 4.52 (q, J = 4.8, 9.5 Hz, 2H, CH_3-$\underline{CH_2}$), 4.13-3.14 (m, 6H, 2H of CH_2 of phenacyl and 4H of piperazine ring near quinolone ring), 2.95-2.56 (br. m, 4H, 4H of piperazine ring near p-Nitrophenacyl group), 1.39 (t, J = 5.1 Hz, 3H, CH_2-$\underline{CH_3}$). ^{13}C NMR 100 MHz (DMSO-d_6): 177.8, 173.8, 163.0, 151.8, 147.2, 145.2, 136.9, 135.3, 132.3, 130.9, 127.9, 111.8, 110.5, 106.2, 64.0, 52.7, 50.0, 49.0, 14.9. Anal. Calcd for $C_{24}H_{24}FN_5O_6$: C, 57.94; H, 4.86; N, 14.08. Found: C, 58.17; H, 5.02; N, 14.35.

Synthesis of hydroxamic acid of different norfloxacin mannich bases.

Synthesis of Indoline-2,3-dione Derivatives (isatin derivatives) [55, 56].

To a solution of 50 mL water, chloral hydrate (4.45 g, 0.027 mol), anhydrous sodium sulfate (65 g, 0.20mol), substituted aniline (0.025 mol), hydroxylammonium chloride (5.45 g, 0.079 mol), and concentrated hydrochloric acid (22 mL) were added, respectively. Subsequently, the resulting suspension was heated to 90 °C for 30 min and cooled to room temperature, the product filtered with a suction pump, and dried in air. The crude was added portion-wise to a 250 mL three mouth flask containing concentrated sulfuric acid (15 mL) at 65°C and then heated up to 80 °C for 1 hour. The reaction solution was cooled to room temperature, poured onto ice water, and stirred vigorously for 90 minutes. The final products were filtered with suction, followed by washing with cold water, and recrystallization from ethanol.

5-fluroindoline-2,3-dione (15c)

Yield= 1.08 g (65%); yellow color, mp: 225-227 ºC (reported mp: 226-228 ºC)[57]

5-chloroindoline-2,3-dione (15d)

Yield= 1.25 g (69%); orange color, mp: 256-259 ºC (reported mp: 256-258 ºC)[57]

5-methylindoline-2,3-dione (15e)

Yield= 1.13 g (70%); red color, mp: 175-177 ºC (reported mp: 179-181 ºC)[58]

5-methoxyindoline-2,3-dione (15f)

Yield= 1.21 g (68%); red color, mp: 195-197 ºC (reported mp: 194-196 ºC)[58]

Synthesis of norfloxacin Mannich derivatives [59].

Equimolar mixtures of norfloxacin (3 g, 9.39 mmol) and the respective indoline-2,3-dione or amine (9.39 mmol) in ethanol (30 mL) were treated with 2 mL of formalin (37%) and heated at reflux overnight (9.7:0.3 chloroform/methanol was used as a mobile phase in TLC monitoring). After cooling, the precipitated product was filtered, washed with water, and dried. Recrystallization from DMF/water mixture afforded the desired Mannich bases.

1-ethyl-6-fluoro-1,4-dihydro-4-oxo-7-(4-((2,3-dioxoindolin-1-yl)methyl)piperazin-1-yl)quinoline-3-carboxylic acid 16a.

Yield= 3.85 g (87%); yellow powder, mp:218-220 ºC (reported mp:164 ºC) [4]. ^{1}H NMR 400 MHz (DMSO-d$_6$) δ (ppm): 15.29 (br. s, 1H, COOH), 8.93 (s, 1H, H-2), 7.9 (d, J_{H-F} = 12.9 Hz, 1H, H-5),7.68 (m, 1H, H-4’ of isatin), 7.58 (d, *J*= 8 Hz, 1H, H-5’ isatin), 7.35 (d, J= 8 Hz, 1H, H-6’ isatin), 7.18 (m, 2H, H-8 and H-7’ of isatin), 4.54 (m, 4H, 2H of -NCH$_2$N and 2H of CH$_3$-$\underline{CH_2}$), 3.35-3.31 (m, 4H, 4H of piperazine near quinolone ring), 2.85-2.81 (m, 4H, 4H of piperazine near isatin moiety), 1.38 (t, *J* = 5.1 Hz, 3H, CH$_2$-$\underline{CH_3}$). ^{13}C NMR 100 MHz (DMSO-d$_6$) 183.5, 176.8, 166.6, 159.5, 154.6, 151.9, 148.9, 145.8, 138.4, 137.7, 124.7, 123.8, 119.8, 118.0, 112.5, 111.7, 107.5, 106.5, 62.1, 50.2, 49.2, 14.7. Anal. Calcd for $C_{25}H_{23}FN_4O_5$: C, 62.76; H, 4.85; N, 11.71. Found: C, 62.95; H, 5.01; N, 11.97

7-(4-((5-bromo-2,3-dioxoindolin-1-yl)methyl)piperazin-1-yl)-1-ethyl-6-fluoro-1,4-dihydro-4-oxoquinoline-3-carboxylic acid 16b.

Yield= 4.2 g (74%); orange powder, mp:213-215 ºC (reported mp:139 ºC) [4]. ^{1}H NMR 400 MHz (DMSO-d$_6$) δ (ppm): 15.32 (br. s, 1H, COOH), 8.92 (s, 1H, H-2), 7.78 (m, 3H, 1H of H-5 and 2H of H-4’,6’ of 5-Brisatin), 7.25 (m, 2H, 1H of H-8 and 1H of H-7’ of 5-Brisatin), 4.54 (m, 4H, 2H of -NCH$_2$N and 2H of CH$_3$-$\underline{CH_2}$), 3.35-3.25 (m, 4H, 4H of piperazine near quinolone ring), 2.9-

2.72 (m, 4H, 4H of piperazine near 5-Brisatin moiety), 1.42 (t, *J* = 5.1 Hz, 3H, CH_2-<u>CH_3</u>). ^{13}C NMR 100 MHz (DMSO-d_6) 182.3, 176.6, 166.5, 159.1, 150.8, 148.9, 145.9, 140.1, 137.7, 126.9, 119.9, 115.6, 114.7, 111.5, 107.7, 106.5, 62.4, 50.0, 49.7, 14.9. **Anal. Calcd for $C_{25}H_{22}BrFN_4O_5$: C, 53.87; H, 3.98; N, 10.05. Found: C, 54.13; H, 4.12; N, 10.29.**

1-ethyl-6-fluoro-7-(4-((5-fluoro-2,3-dioxoindolin-1-yl)methyl)piperazin-1-yl)-1,4-dihydro-4-oxoquinoline-3-carboxylic acid 16c.

Yield= 4.1 g (89%); orange powder, mp:248-250 ºC. IR (KBr): 3451(OH str), 3049(aromatic C-H str), 2852(aliphatic C-H str), 1716(carboxylic C=O str), 1748(carbamidic C=O str), 1619(quinolone C=O str), 1250(C-O) cm^{-1}. ^{1}H NMR 400 MHz (DMSO-d_6) δ (ppm): 15.35 (br. s, 1H, COOH), 8.95 (s, 1H, H-2), 7.9 (d, J_{H-F} = 12.9 Hz, 1H, H-5), 7.57 (t. d, *J*= 12 $_{H-F}$, 9, 2.8 Hz, 1H, H-4' of 5-Fisatin), 7.49 (d. d, *J* = 7.2, 2.8 Hz, 1H, H-6' of 5-Fisatin), 7.37 (d. d, *J*= 8.4, 3.6 Hz, 1H, H-7' of 5-Fisatin), 7.17 (d, J_{H-F} = 6.6 Hz, 1H, H-8), 4.59 (q, *J*= 7.2, 2H, CH_3-<u>CH_2</u>), 4.53 (s, 2H, ,-NCH_2N), 3.33:3.31 (m, 4H, 4H of piperazine near quinolone ring), 2.84-2.81 (m, 4H, 4H of piperazine near 5-Fisatin moiety), 1.4 (t, *J* = 5.1 Hz, 3H, CH_2-<u>CH_3</u>). ^{13}C NMR (100 MHz, DMSO-d_6): 182.6, 176.3, 166.2, 159.2, 157.5, 151.5, 148.5, 147.5, 145.5, 137.2, 124.0, 123.7, 119.4, 118.6, 113.5, 111.5, 107.1, 106.1, 61.5, 49.6, 49.4, 49.1, 14.74. Anal. Calcd for $C_{25}H_{22}F_2N_4O_5$: C, 60.48; H, 4.47; N, 11.29. Found: C, 60.71; H, 4.59; N, 11.46.

7-(4-((5-chloro-2,3-dioxoindolin-1-yl)methyl)piperazin-1-yl)-1-ethyl-6-fluoro-1,4-dihydro-4-oxoquinoline-3-carboxylic acid 16d.

Yield= 4.15 g (87%); orange powder, mp:246-248 ºC (reported mp:120 ºC) [4]. ^{1}H NMR 400 MHz (DMSO-d_6) δ (ppm): 15.36 (br. s, 1H, COOH), 8.96 (s, 1H, H-2), 7.91 (d, J_{H-F} = 12.9 Hz, 1H, H-5), 7.74 (d. d, *J*= 8.6, 2.5 Hz, 1H, 1H of H-4' of 5-Clisatin), 7.46 (d, *J*= 7.2, 1H, 1H of H-6' of 5-Clisatin), 7.38 (d, *J*= 8.4, 1H, H-7' of 5-Clisatin), 7.18 (d, J_{H-F} = 6.6 Hz, 1H, H-8), 4.59 (q, *J*= 7.2, 2H, CH_3-<u>CH_2</u>), 4.53 (s, 2H, ,-NCH_2N), 3.34-3.3 (m, 4H, 4H of piperazine near quinolone ring), 2.84-2.81 (m, 4H, 4H of piperazine near 5-Clisatin moiety), 1.41 (t, *J* = 5.1 Hz, 3H, CH_2-<u>CH_3</u>). Anal. Calcd for $C_{25}H_{22}ClFN_4O_5$: C, 58.54; H, 4.32; N, 10.92. Found: C, 58.78; H, 4.47; N, 11.14.

7-(4-((5-methyl-2,3-dioxoindolin-1-yl)methyl)piperazin-1-yl)-1-ethyl-6-fluoro-1,4-dihydro-4-oxoquinoline-3-carboxylic acid 16e.

Yield= 3.95 g (85%); orange powder, mp:247-249 ºC. ^{1}H NMR 400 MHz (DMSO-d_6) δ (ppm): 15.36 (br. s, 1H, COOH), 8.96 (s, 1H, H-2), 7.92 (d, J_{H-F} = 12.9 Hz, 1H, H-5), 7.54-7.18 (m, 4H, 3H of H-4', 6', 7' of 5-Meisatin and 1H of H-8), 4.56 (m,4H, 2H, CH_3-<u>CH_2</u> and 2H of -NCH_2N),

3.4-3.3 (m, 4H, 4H piperazine near quinolone ring), 2.85-2.78 (m, 4H, 4H of piperazine near 5-Meisatin moiety), 2.31 (s, 3H of CH_3 5-Meisatin), 1.41 (t, J = 5.1 Hz, 3H, CH_2-$\underline{CH_3}$). ^{13}C NMR 100 MHz (DMSO-d_6): 183.9, 176.6, 166.6, 159.6, 152.2, 149.8, 149.1, 146.0, 138.8, 137.7, 133.1, 125.0, 119.9, 118.0, 112.3, 111.6, 107.5, 106.6, 61.8, 50.1, 49.8, 49.5, 20.5, 14.8. Anal. Calcd for $C_{26}H_{25}FN_4O_5$: C, 63.41; H, 5.12; N, 11.38. Found: C, 63.29; H, 5.32; N, 11.50.

7-(4-((5-methoxy-2,3-dioxoindolin-1-yl)methyl)piperazin-1-yl)-1-ethyl-6-fluoro-1,4-dihydro-4-oxoquinoline-3-carboxylic acid 16f.

Yield= 3.7 g (77%); red powder, mp:251-253 ºC. ^{1}H NMR 400 MHz (DMSO-d_6) δ (ppm): 15.34 (br. s, 1H, COOH), 8.94 (s, 1H, H-2), 7.91 (d, $J_{H\text{-}F}$ = 12.9 Hz, 1H, H-5), 7.2 (m, 4H, 1H of H-8 and 3H of H-4', 6', 7' of 5-Methoxyisatin), 4.53 (m, 4H, 2H, CH_3-$\underline{CH_2}$ and 2H,-NCH_2N), 3.78 (s, 3H, 3H of OCH_3), 3.31 (m, 4H, 4H of piperazine near quinolone ring), 2.81-2.78 (m, 4H, 4H of piperazine near 5-Methoxyisatin), 1.39 (t, J = 5.1 Hz, 3H, CH_2-$\underline{CH_3}$). ^{13}C NMR 100 MHz (DMSO-d_6): 185.1, 176.7, 166.5, 160.0, 152.0, 149.3, 145.1, 144.5, 137.6, 125.4, 124.2, 118.6, 113.7, 112.1, 109.2, 107.7, 107.0, 60.3, 56.4, 49.7, 47.3, 43.0, 15.0. Anal. Calcd for $C_{26}H_{25}FN_4O_6$: C, 61.41; H, 4.96; N, 11.02. Found: C, 61.59; H, 5.12; N, 11.28.

1-ethyl-6-fluoro-1,4-dihydro-4-oxo-7-(4-((piperidin-1-yl)methyl)piperazin-1-yl)quinoline-3-carboxylic acid 19a.

Yield= 3.15 g (80%); white powder, mp:278-280 ºC (reported mp:>300 ºC) [60]. IR (KBr): 3414(OH str), 3023(aromatic C-H str), 2916(aliphatic C-H str), 1719(carboxylic C=O str), 1638(quinolone C=O str), 1263(C-O) cm^{-1}. ^{1}H NMR 400 MHz (DMSO-d_6) δ (ppm): 15.39 (br. s, 1H, COOH), 8.97 (s, 1H, H-2), 7.94 (d, $J_{H\text{-}F}$ = 12.9 Hz, 1H, H-5), 7.21 (d, $J_{H\text{-}F}$ = 6.6 Hz, 1H, H-8), 4.6 (q, J = 4.8, 9.5 Hz, 2H, CH_3-$\underline{CH_2}$), 3.46-3.4 (m, 10H, 2H of -NCH_2N, 4H of piperazine near quinolone ring and 4H of piperidine ring near nitrogen overlapped with H_2O peak), 2.77-2.68 (m, 4H, 4H of piperazine near piperidine ring), 2.53-2.48 (m, 4H, 4H of piperidine in middle of ring), 1.42 (t, J = 5.1 Hz, 3H, CH_2-$\underline{CH_3}$), 1.23 (t, J = 7.2 Hz, 2H, 2H of piperidine). Anal. Calcd for C22H29FN4O3: C, 63.44; H, 7.02; N, 13.45. Found: C, 63.70; H, 7.19; N, 13.62.

1-ethyl-6-fluoro-1,4-dihydro-7-(4-(morpholinomethyl)piperazin-1-yl)-4-oxoquinoline-3-carboxylic acid 19b.

Yield= 3.25 g (83%); white powder, mp:290-292 ºC (reported mp:287-288 ºC) [59]. IR (KBr): 3424(OH str), 3028(aromatic C-H str), 2931(aliphatic C-H str), 1712(carboxylic C=O str), 1632(quinolone C=O str), 1265(C-O), 1018(C-N) cm^{-1}. ^{1}H NMR 400 MHz (DMSO-d_6) δ (ppm):

15.43 (br. s, 1H, COOH), 8.95 (s, 1H, H-2), 7.95 (d, J_{H-F} = 12.9 Hz, 1H, H-5), 7.21 (d, J_{H-F} = 6.6 Hz, 1H, H-8), 4.57 (q, J = 4.8, 9.5 Hz, 2H, CH_3-<u>CH_2</u>), 3.37 (s, 2H, -NCH_2N), 3.3-3.25 (br. m, 4H, 4H of morpholine near oxygen), 2.92-2.71 (br. m, 4H, 4H of piperazine near quinolone ring), 2.53-2.48 (br. m, 8H, 4H of morpholine near nitrogen and 4H of piperazine near morpholine), 1.44 (t, J = 5.1 Hz, 3H, CH_2-<u>CH_3</u>). Anal. Calcd for $C_{21}H_{27}FN_4O_4$: C, 60.27; H, 6.50; N, 13.39. Found: C, 60.45; H, 6.72; N, 13.58.

1-ethyl-6-fluoro-1,4-dihydro-4-oxo-7-(4-((2,5-dioxopyrrolidin-1-yl)methyl)piperazin-1-yl)quinoline-3-carboxylic acid 22a.

Yield= 3.55 g (88%); white powder, mp:259-261 ºC (reported mp:266-267 ºC) [59]. ^{1}H NMR 400 MHz (DMSO-d_6) δ (ppm): 15.31 (s, 1H, COOH), 8.93 (s, 1H, H-2), 7.88 (d, J_{H-F} = 12.9 Hz, 1H, H-5), 7.16 (d, J_{H-F} = 6.6 Hz, 1H, H-8), 4.58 (q, J = 4.8, 9.5 Hz, 2H, CH_3-<u>CH_2</u>), 4.34 (br. s, 2H, -NCH_2N), 3.34-3.26 (m, 4H, 4H of piperazine near quinolone ring), 2.74-2.67 (m, 8H, 4H of succinimide and 4H of piperazine near succinimide), 1.42 (t, J = 5.1 Hz, 3H, CH_2-<u>CH_3</u>). ^{13}C NMR 100 MHz (DMSO-d_6) 179.2, 176.2, 166.5, 148.5, 137.7, 111.4, 107.4, 106.4, 106.2, 59.5, 50.3, 28.5, 14.7. Anal. Calcd for $C_{21}H_{23}FN_4O_5$: C, 58.60; H, 5.39; N, 13.02. Found: C, 58.73; H, 5.53; N, 13.18.

1-ethyl-6-fluoro-1,4-dihydro-4-oxo-7-(4-((1,3-dioxoisoindolin-2-yl)methyl)piperazin-1-yl)quinoline-3-carboxylic acid 22b.

Yield= 4.05 g (90%); white powder, mp:248-250 ºC (reported mp:256-257 ºC) [59]. IR (KBr): 3489(OH str), 3041(aromatic C-H str), 2956(aliphatic C-H str), 1771(imidic C=O str), 1708(carboxylic C=O str), 1624(quinolone C=O str), 1254(C-O) cm^{-1}. ^{1}H NMR 400 MHz ($CDCl_3$) δ (ppm): 15 (s, 1H, COOH), 8.63 (s, 1H, H-2), 8.06 (d, J_{H-F} = 12.9 Hz, 1H, H-5), 7.91 (d, J= 8 Hz, 2H, H-3' and 6' of phthalimide), 7.77 (d, J= 8 Hz, 2H, H-1', 2'H of phthalimide), 6.82 (d, J_{H-F} = 6.6 Hz, 1H, H-8), 4.77 (s, 2H), 4.32 (q, J = 4.8, 9.5 Hz, 2H, CH_3-<u>CH_2</u>), 3.41-3.36 (m, 4H, 4H of piperazine near quinolone ring), 2.99- 2.94 (m, 4H, 4H of piperazine near phthalimide), 1.5 (t, J = 5.1 Hz, 3H, CH_2-<u>CH_3</u>). Anal. Calcd for $C_{25}H_{23}FN_4O_5$: C, 62.76; H, 4.85; N, 11.71. Found: C, 62.59; H, 4.97; N, 11.95.

7-(4-((4-nitrophenylamino)methyl)piperazin-1-yl)-1-ethyl-6-fluoro-1,4-dihydro-4-oxoquinoline-3-carboxylic acid 25.

Yield= 3.45 g (78%); yellow powder, mp:246-248 ºC. ^{1}H NMR 400 MHz (DMSO-d_6) δ (ppm): 15.36 (br. s, 1H, COOH), 8.97 (s, 1H, H-2), 8.19-7.88 (m, 3H, 1H of H-5, 2H of H-3', 5', Ar-H),

7.79 (s, 1H, NH of P-Nitroaniline), 7.03 (m, 3H, 1H of H-8 and 2H of H-2', 6', Ar-H), 4.6 (q, *J* = 4.8, 9.5 Hz, 2H, CH_3-<u>CH_2</u>), 3.98 (s, 2H, -NCH_2N), 3.38-3.3 (m, 4H, 4H of piperazine near quinolone ring), 2.76-2.68 (m, 4H, 4H of piperazine near p-Nitroaniline), 1.42 (t, *J* = 5.1 Hz, 3H, CH_2-<u>CH_3</u>). ^{13}C NMR 100 MHz (DMSO-d_6): 176.8, 166.7, 156.3, 154.6, 149.0, 146.0, 141.6, 137.6, 126.6, 126.4, 114.9, 112.8, 111.9, 107.9, 64.9, 51.1, 51.1, 50.1, 49.5, 15.0. Anal. Calcd for $C_{23}H_{24}FN_5O_5$: C, 58.84; H, 5.15; N, 14.92. Found: C, 59.11; H, 5.34; N, 15.19.

Synthesis of the corresponding hydroxamic derivatives.

To a stirred solution of the respective norfloxacin Mannich derivatives (0.5 g) in dichloromethane (20 mL) in an ice bath, triethylamine (2 equivalents) and ethyl chloroformate (1.5 equivalents) were added and stirring continued in an ice bath for 1 hour. The mixture was then treated with hydroxylamine hydrochloride (2 equivalents) and stirring at room temperature was continued for 6-8 hours. Progress of the reaction was observed by TLC monitoring (9.7:0.3 chloroform/methanol). The organic layer was washed with saturated brine solution (2 x 25 mL) and distilled water (2 x 25 mL), and dried over sodium sulfate anhydrous. Evaporation under vacuum of the organic layer afforded crude product that was recrystallized from methanol.

1-Ethyl-6-fluoro-1,4-dihydro-*N*-hydroxy-7-(4-methylpiperazin-1-yl)-4-oxoquinoline-3-carboxamide 26.

Yield= 0.33 g (63%); greenish yellow powder, mp:206-208 ºC. IR (KBr): 3417(NH str), 3146(OH str), 3046(aromatic C-H str), 2819(aliphatic C-H str), 1683(hydroxamic C=O str), 1620(quinolone C=O str), 1257(C-O) cm^{-1}. ^{1}H NMR (DMSO-d_6) δ (ppm): 11.77 (br. s, 1H, NH of hydroxamic), 8.8 (s, 1H, H-2), 7.9 (d, J_{H-F} = 12.9 Hz, 1H, H-5), 7.11 (d, J_{H-F} = 6.6 Hz, 1H, H-8), 4.53 (q, *J* = 4.8, 9.5 Hz, 2H, CH_3-<u>CH_2</u>), 3.26-2.99 (m, 8H, 8H of piperazine ring), 2.99 (s, 3H of *N*-CH_3), 1.4 (t, *J* = 5.1 Hz, 3H, CH_2-<u>CH_3</u>). ^{13}CNMR (DMSO-d_6) δ (ppm): 173.3, 162.5, 151.3, 146.7, 144.9, 136.5, 121.0, 112.0, 110.0, 105.4, 49.9, 48.4, 44.3, 14.3. Anal. Calcd for $C_{17}H_{21}FN_4O_3$: C, 58.61; H, 6.08; N, 16.08. Found: C, 58.72; H, 6.05; N, 15.91.

1-ethyl-6-fluoro-1,4-dihydro-*N*-hydroxy-4-oxo-7-(4-((2,3-dioxoindolin-1-yl)methyl)piperazin-1-yl)quinoline-3-carboxamide 17a.

Yield=0.39 g (76%); yellow powder, mp:188-190 ºC. IR (KBr): 3431(NH str), 3166(OH str), 3051(aromatic C-H str), 2837(aliphatic C-H str), 1724(ketonic C=O str), 1658(carbamidic C=O

str), 1628(quinolone C=O str), 1258(C-O), 1017(C-N) cm^{-1}. ^{1}H NMR 400 MHz (DMSO-d_6) δ (ppm): 11.79 (br. s, 1H, NH of hydroxamic), 8.96-7 (m, 8H, 1H of H-2, 1H of OH hydroxamic, 1H of H-5, 4H of H-4', 5', 6' 7'H of isatin and 1H of H-8), 4.59 (m, 4H, 2H of -NCH_2N and 2H of CH_3-$\underline{CH_2}$), 3.35-2.7 (m, 8H, 8H of piperazine ring), 1.4 (t, J = 5.1 Hz, 3H, CH_2-$\underline{CH_3}$). ^{13}C NMR 100 MHz (DMSO-d_6): 173.7, 165.0, 163.4, 154.6, 152.0, 147.3, 144.3, 144.1, 137.6, 132.6, 127.6, 127.2, 122.9, 115.5, 112.0, 110.9, 106.2, 61.4, 49.7, 46.0, 15.0. Anal. Calcd for $C_{25}H_{24}FN_5O_5$: C, 60.85; H, 4.90; N, 14.19. Found: C, 61.08; H, 5.12; N, 14.37. LRMS for [C24H23FN4O4] $^{+}$ $[M]^{+}$ calculated: 493.18 found: 493.12.

7-(4-((5-bromo-2,3-dioxoindolin-1-yl)methyl)piperazin-1-yl)-1-ethyl-6-fluoro-1,4-dihydro-*N*-hydroxy-4-oxoquinoline-3-carboxamide 17b.

Yield= 0.38 g (74%); yellow powder, mp:191-193 °C. IR (KBr): 3435(Nh str), 3135(OH str), 3063(aromatic C-H str), 2979(aliphatic C-H str), 1721(ketonic C=O str), 1662(carbamidic C=O str), 1627(quinolone C=O str), 1257(C-O) cm^{-1}. ^{1}H NMR 400 MHz (DMSO-d_6) δ (ppm): 11.77 (br. s, 1H, NH of hydroxamic), 9.2 (br. s, 1H, OH of hydroxamic), 8.77 (s, 1H, H-2), 8.11 (d, J_{H-F} = 12.9 Hz, 1H, H-5), 7.76 (m, 2H, H-4' and 6' of 5-Brisatin), 7.17 (m, 2H, 1H of H-8 and 1H of H-7' of 5-Brisatin), 4.58 (m, 4H, 2H of -NCH_2N and 2H of CH_3-$\underline{CH_2}$), 3.5-2.75 (m, 8H, 8H of piperazine ring), 1.39 (t, J = 5.1 Hz, 3H, CH_2-$\underline{CH_3}$). ^{13}C NMR 100 MHz (DMSO-d_6): 173.3, 166.2, 163.6, 162.5, 154.1, 142.7, 142.5, 136.4, 134.2, 128.7, 121.1, 116.8, 114.3, 112.6, 111.6, 110.0, 107.1, 61.0, 49.8, 49.5, 48.4, 14.3. Anal. Calcd for $C_{25}H_{24}FN_5O_5$: C, 52.46; H, 4.05; N, 12.24. Found: C, 52.29; H, 4.31; N, 12.48.

1-ethyl-6-fluoro-7-(4-((5-fluoro-2,3-dioxoindolin-1-yl)methyl)piperazin-1-yl)-1,4-dihydro-*N*-hydroxy-4-oxoquinoline-3-carboxamide 17c.

Yield= 0.41 g (80%); yellow powder, mp:212-214 °C. ^{1}H NMR 400 MHz (DMSO-d_6) δ (ppm): 11.76 (br. s, 1H, NH of hydroxamic), 9.21 (br. s, 1H, OH of hydroxamic), 8.77 (s, 1H, H-2), 7.84 (d, J_{H-F} = 12.9 Hz, 1H, H-5), 7.8 (d. d, J= 8,2.4 Hz, 1H, H-4' of 5-Fisatin), 7.31 (m, 2H, H-6' and 7' of 5-Fisatin), 7.14 (d, J_{H-F} = 6.6 Hz, 1H, H-8), 4.57 (s, 2H, -NCH_2N), 4.54 (q, J = 4.8, 9.5 Hz, 2H, CH_3-$\underline{CH_2}$), 3.27-3.24 (m, 4H, 4H of piperazine near quinolone ring), 2.79-2.74 (m, 4H, 4H of piperazine near 5-Fisatin), 1.35 (t, J = 5.1 Hz, 3H, CH_2-$\underline{CH_3}$). ^{13}C NMR 100 MHz (DMSO-d_6): 173.5, 163.9, 162.5, 156.9, 153.8, 151.5, 146.8, 144.5, 143.5, 139.9, 136.4, 121.4, 118.1, 115.8,

113.4, 111.8, 110.0, 105.8, 61.0, 49.9, 49.5, 48.4, 14.5. Anal. Calcd for $C_{25}H_{23}F_2N_5O_5$: C, 58.71; H, 4.53; N, 13.69. Found: C, 58.98; H, 4.70; N, 13.85.

7-(4-((5-chloro-2,3-dioxoindolin-1-yl)methyl)piperazin-1-yl)-1-ethyl-6-fluoro-1,4-dihydro-*N*-hydroxy-4-oxoquinoline-3-carboxamide 17d.

Yield= 0.4 g (78%); yellow powder, mp:198-200 ºC. ^{1}H NMR 400 MHz (DMSO-d_6) δ (ppm): 11.76 (br. s, 1H, NH of hydroxamic), 9.21 (br. s, 1H, OH of hydroxamic), 8.8 (s, 1H, H-2), 8.04 (d, J_{H-F}= 12.9 Hz, 1H, H-5), 7.87 (d, *J*=10 Hz,1H, H-4' of 5-Clisatin), 7.56 (d. d, *J*= 8, 2.4 Hz,1H, H-6' of 5-Clisatin), 7.36 (d, J= 8.4 Hz, 1H, H-7' of 5-Clisatin), 7.11 (d, J_{H-F}= 6.6 Hz, 1H, H-8), 4.59 (s, 2H, -NCH_2N), 4.52 (q, *J* = 4.8, 9.5 Hz, 2H, CH_3-$\underline{CH_2}$), 3.27-3.24 (m, 4H, 4H of piperazine near quinolone ring), 2.77-2.75 (m, 4H, 4H of piperazine ring near 5-Clisatin), 1.39 (t, *J* = 5.1 Hz, 3H, CH_2-$\underline{CH_3}$). ^{13}C NMR 100 MHz (DMSO-d_6): 173.4, 163.6, 162.6, 154.0, 146.7, 144.7, 142.7, 142.4, 136.4, 131.4, 126.8, 126.0, 121.1, 116.5, 112.4, 111.4, 110.1, 105.8, 61.0, 49.9, 49.5, 48.5, 14.5. Anal. Calcd for $C_{25}H_{23}ClFN_5O_5$: C, 56.88; H, 4.39; N, 13.27. Found: C, 57.14; H, 4.51; N, 13.44.

7-(4-((5-methyl-2,3-dioxoindolin-1-yl)methyl)piperazin-1-yl)-1-ethyl-6-fluoro-1,4-dihydro-*N*-hydroxy-4-oxoquinoline-3-carboxamide 17e.

Yield= 0.35 g (68%); yellow powder, mp: 200-202 ºC. ^{1}H NMR (400 MHz (DMSO-d_6) δ (ppm): 11.73 (br. s, 1H, NH of hydroxamic), 9.18 (br. s, 1H, OH of hydroxamic), 8.77 (s, 1H, H-2), 7.86 (m, 2H, 1H of H-5 and 1H of H-7' of 5-Meisatin), 7.16 (m, 3H, 2H of H-4',6' of 5-Meisatin and 1H of H-8), 4.52 (m, 4H, 2H of CH_3-$\underline{CH_2}$ and 2H of -NCH_2N), 3.26-3.21 (m, 4H, 4H of piperazine near quinolone ring), 2.76-2.68 (m, 4H, 4H of piperazine near 5-Meisatin), 2.3 (s, 3H, CH_3 of 5-Meisatin), 1.38 (t, *J* = 5.1 Hz, 3H, CH_2-$\underline{CH_3}$). ^{13}C NMR 100 MHz (DMSO-d_6): 173.8, 164.5, 163.0, 154.3, 151.9, 147.2, 144.0, 141.9, 136.9, 132.6, 132.2, 127.7, 121.5, 115.7, 112.0, 110.7, 110.5, 106.3, 61.3, 50.4, 50.0, 48.9, 21.0, 15.0. Anal. Calcd for $C_{26}H_{26}FN_5O_5$: C, 61.53; H, 5.16; N, 13.80. Found: C, 61.37; H, 5.40; N, 14.07.

7-(4-((5-methoxy-2,3-dioxoindolin-1-yl)methyl)piperazin-1-yl)-1-ethyl-6-fluoro-1,4-dihydro-*N*-hydroxy-4-oxoquinoline-3-carboxamide 17f.

Yield= 0.37 g (71%); yellow powder, mp: 193-195 ºC. ^{1}H NMR 400 MHz (DMSO-d_6) δ (ppm): 11.74 (br. s, 1H, NH of hydroxamic), 9.17 (br. s, 1H, OH of hydroxamic), 8.75 (s, 1H, H-2), 7.83 (d, J_{H-F}= 12.9 Hz, 1H, H-5), 7.59 (d, *J*= 2.4 Hz, 1H, 1H of H-7' of 5-Methoxyisatin), 7.18 (d, J_{H-}

$_F$= 6.6 Hz,1H, H-8), 7.06 (m, 2H, 2H of H-4' and 6' of 5-Methoxyisatin), 4.5 (m, 4H, 2H of CH_3-$\underline{CH_2}$ and 2H of -NCH_2N), 3.74 (s, 3H, OCH_3 group), 3.25-3.22 (m, 4H, 4H of piperazine near quinolone ring), 2.76-2.72 (m, 4H, 4H of piperazine near 5-Methoxyisatin), 1.35 (t, J = 5.1 Hz, 3H, CH_2-$\underline{CH_3}$). ^{13}C NMR 100 MHz (DMSO-d_6): 173.6, 164.9, 163.9, 162.5, 155.2, 152.2, 146.5, 143.5, 137.4, 136.4, 121.1, 117.8, 117.1, 116.1, 112.8, 111.3, 110.0, 106.1, 64.9, 60.9, 55.6, 49.6, 45.9, 14.1. Anal. Calcd for $C_{26}H_{26}FN_5O_6$: C, 59.65; H, 5.01; N, 13.38. Found: C, 59.88; H, 5.13; N, 13.52.

1-ethyl-6-fluoro-1,4-dihydro-*N*-hydroxy-4-oxo-7-(4-((piperidin-1-yl)methyl)piperazin-1-yl)quinoline-3-carboxamide 20a.

Yield= 0.31 g (60%); white powder, mp:216-218 °C. IR (KBr): 3433(NH str), 3192(OH str), 3049(aromatic C-H str), 2954(aliphatic C-H str),1686(hydroxamic C=O str), 1625(quinolone C=O str), 1261(C-O) cm^{-1}. ^{1}H NMR 400 MHz (DMSO-d_6) δ (ppm): 11.77 (br. s, 1H, NH of hydroxamic), 9.19 (br. s, 1H, OH of hydroxamic), 8.8 (s, 1H, H-2), 7.9 (d, J_{H-F}= 12.9 Hz, 1H, H-5), 7.15 (d, J_{H-F}= 6.6 Hz, 1H, H-8), 4.55 (q, J = 4.8, 9.5 Hz, 2H, CH_3-$\underline{CH_2}$), 3.45-3.01 (m, 14H, 2H of -NCH_2N and 8H of piperazine and 4H of piperidine ring), 2.95-2.63 (m, 4H of piperidine ring), 1.35 (m, 5H, 3H of CH_2-$\underline{CH_3}$ and 2H of piperidine). ^{13}C NMR 100 MHz (DMSO-d_6): 173.8, 162.9, 151.6, 147.3, 144.1, 136.9, 122.1, 112.2, 110.5, 106.6, 49.0, 47.0, 45.8, 42.9, 14.9, 9.0. Anal. Calcd for $C_{22}H_{30}FN_5O_3$: C, 61.24; H, 7.01; N, 16.23. Found: C, 61.48; H, 7.09; N, 16.17.

1-ethyl-6-fluoro-1,4-dihydro-*N*-hydroxy-7-(4-(morpholinomethyl)piperazin-1-yl)-4-oxoquinoline-3-carboxamide 20b.

Yield= 0.34 g (66%); buff powder, mp:237-239 °C. IR (KBr): 3434(NH str), 3182(OH str), 3079(aromatic C-H str), 2914(aliphatic C-H str), 1680(hydroxamic C=O str), 1617(quinolone C=O str), 1261(C-O), 1185(C-N) cm^{-1}. ^{1}H NMR 400 MHz (DMSO-d_6) δ (ppm): 11.74 (br. s, 1H, NH of hydroxamic), 8.75 (br. s, 2H, OH of hydroxamic and 1H of H-2), 7.86 (d, J_{H-F}= 12.9 Hz, 1H, H-5), 7.06 (d, J_{H-F}= 6.6 Hz, 1H, H-8), 4.51 (s, 2H, -NCH_2N), 2.5-3.4 (m, 18H, 2H of CH_3-$\underline{CH_2}$ and 16H of morpholine and piperazine ring), 1.4 (t, J = 5.1 Hz, 3H, CH_2-$\underline{CH_3}$). ^{13}C NMR 100 MHz (DMSO-d_6): 173.1, 162.6, 151.6, 146.9, 143.9, 136.5, 121.8, 111.8, 110.1, 106.1, 48.5, 46.5, 45.3, 42.5, 14.5. Anal. Calcd for $C_{21}H_{28}FN_5O_4$: C, 58.19; H, 6.51; N, 16.16. Found: C, 58.40; H, 6.73; N, 16.42.

1-ethyl-6-fluoro-1,4-dihydro-*N*-hydroxy-4-oxo-7-(4-((2,5-dioxopyrrolidin-1-yl)methyl)piperazin-1-yl)quinoline-3-carboxamide 23a.

Yield= 0.37 g (71%); pale yellow powder, mp:178-180 °C. IR (KBr): 3429(NH str), 3189(OH str), 3056(aromatic C-H str), 2945(aliphatic C-H str), 1746(imidic C=O str), 1701(hydroxamic C=O str), 1624(quinolone C=O str), 1259(C-O), 1160(C-N) cm^{-1}. ^{1}H NMR 400 MHz (DMSO-d_6) δ (ppm): 11.76 (br. s, 1H, NH of hydroxamic), 8.79 (br. s, 2H, OH of hydroxamic and 1H of H-2), 7.91 (d, J_{H-F} = 12.9 Hz, 1H, H-5), 7.13 (d, J_{H-F} = 6.6 Hz, 1H, H-8), 4.56 (q, J = 4.8, 9.5 Hz, 2H, CH_3-$\underline{CH_2}$), 3.5-3.22 (m, 6H, 4H of piperazine near quinolone ring and 2H of -NCH_2N), 3.05 (m, 4H, 4H of piperazine near succinimide), 2.52 (s, 4H, 4H of succinimide), 1.4 (t, J = 5.1 Hz, 3H, CH_2-$\underline{CH_3}$). ^{13}C NMR 100 MHz (DMSO-d_6): 179.1, 173.3, 162.5, 151.5, 146.9, 144.5, 136.5, 121.1, 111.8, 110.1, 105.8, 49.8, 49.2, 48.5, 44.3, 29.5, 14.5. Anal. Calcd for $C_{21}H_{24}FN_5O_5$: C, 56.62; H, 5.43; N, 15.72. Found: C, 56.89; H, 5.61; N, 15.88.

1-ethyl-6-fluoro-1,4-dihydro-*N*-hydroxy-4-oxo-7-(4-((1,3-dioxoisoindolin-2-yl)methyl)piperazin-1-yl)quinoline-3-carboxamide 23b.

Yield= 0.38 g (74%); pale orange powder, mp:166-168 °C. ^{1}H NMR 400 MHz (DMSO-d_6) δ (ppm): 11.75 (s, 1H, NH of hydroxamic), 8.77 (br. s, 2H, OH of hydroxamic and 1H of H-2), 7.85-7.08 (m, 6H, 1H of H-5, 4H of phthalimide and 1H of H-8), 4.5 (q, J = 4.8, 9.5 Hz, 2H, CH_3-$\underline{CH_2}$), 3.37-2.99 (m, 10H, 2H of -NCH_2N and 8H of piperazine ring), 1.39 (t, J = 5.1 Hz, 3H, CH_2-$\underline{CH_3}$). ^{13}C NMR 100 MHz (DMSO-d_6) 174.1, 169.4, 163.3, 151.7, 147.3, 145.0, 136.9, 131.3, 128.3, 121.6, 112.2, 110.5, 106.5, 50.4, 48.7, 45.1, 15.0. Anal. Calcd for $C_{25}H_{24}FN_5O_5$: C, 60.85; H, 4.90; N, 14.19. Found: C, 60.73; H, 5.12; N, 14.37.

Text S12: Molecular modeling

Optimization of target compounds

The target ligands for modelling compounds were built using the builder interface of the MOE software package 2020.01 and subjected to conformational search. Conformers were subjected to energy minimization until a RMSD gradient of 0.01 kcal mol^{-1} and RMS distance of 0.1 Å with MMFF94X force-field and the partial charges were automatically calculated. The obtained database was then saved as MDB file to be used in the physicochemical properties and docking calculations.

Calculation of physicochemical properties

Calculation of the physicochemical properties of the compounds, including AM1_dipole (AM1), water accessible surface area (ASA), Lipinski acceptor count (lip_acc), Lipinski donor count (lip_don), Lipinski druglike test (lip_druglike), log octanol/water partition coefficient (logP(o/w)), log solubility in water (logS), topological polar surface area (TPSA), van der waals surface area (VSA), molecular weight (weight), and number of rotatable bonds (nrotb), were calculated on MOE 2020.1 using the calculate descriptors command.

Molecular docking

The crystal structure of moxifloxacin with *S. aureus* DNA gyrase and DNA (PDB code 5cdq)[28], the crystal structure of moxifloxacin, DNA, and *A. baumannii* tomoisomerase IV (PDB code 2xkk)[29], structure of co-crystal of *P. aeruginosa* LpxC-50432 complex (PDB code: 6mod)[32], and the crystal structure of N-acetyl-D-glucosamine-6-phosphate deacetylase D267A mutant from *M. smegmatis* in complex with N-acetyl-D-glucosamine-6-phosphate (PDB code 6fv4)[31] were obtained from the protein data bank (PDB). Docking was run on the binding site of the co-crystallized ligand. Since the crystal structure contains a ligand molecule, the program automatically identifies the binding site, and the tested ligands were docked onto it. Docking of the conformations database of the target ligands was done using MOE-DOCK software wizard. The following parameters were adjusted: 1. receptor and solvent as receptor, 2. co-crystalized ligand atoms as active site, database containing test ligands as ligand, London dG as initial scoring function, GBVI/WSA dG as final scoring function, and MMFF94x force field was used for calculating the energy parameters of the ligand – cleavage complex model. To compare between

the conformers London dG was used as scoring function. The 2D and 3D ligand interactions for each compound were saved as picture files and color coding was chosen according to **Figure S148**.

Ligand-based pharmacophore modelling

The ligand-based pharmacophore query was determined from a collection of 40 active ligands in MOE 2020.01 using the following steps: 1. flexible alignment, 2. pharmacophore consensus, 3. feature selection and pharmacophore saving, 4. model validation, 5. pharmacophore search. The training set (40 compounds, **Table S14**), the validation test set (17 compounds, **Table S15**) and the target compound set (56 compounds) were built using the MOE builder interface and subjected to conformational search. Conformers were subjected to energy minimization as in the mentioned docking experiments. The obtained databases were then saved as MDB file to be used in the flexible alignment, validation, and pharmacophore search. Flexible alignment was adjusted to iteration limit = 200, failure limit = 30, and energy cutoff = 20.

in silico ADME/Tox profile of the new compounds

Two ADME/Tox web tools were used in the predictive study: pkCSM-pharmacokinetics (http://biosig.unimelb.edu.au/pkcsm/prediction)[61] and SwissADME (http://www.swissadme.ch/)[25]. The molecular structures of the new compounds and norfloxacin were built in ChemDraw Ultra 8.0, copied as SMILES (simplified molecular-input line-entry specification) nomenclature, and pasted into the web tools. The most important ADME/Tox properties provided from the web tools were selected to represent the ADME/Tox profile.

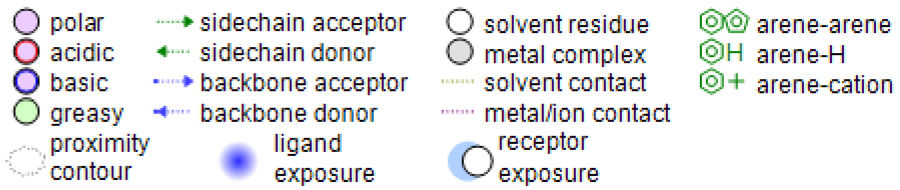

Figure S148: Color scheme for the 2D representations of the interactions between the docked ligands and the active site of the enzyme.

Table S14: Training set compounds.

Code	$IC_{50\%}$ (nM)	Code	$IC_{50\%}$ (nM)
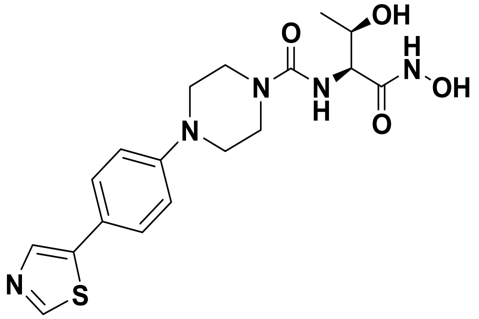 **27**	12	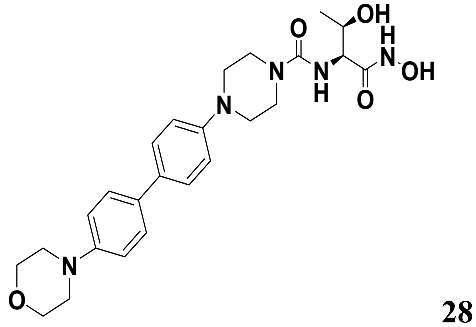 **28**	16.3
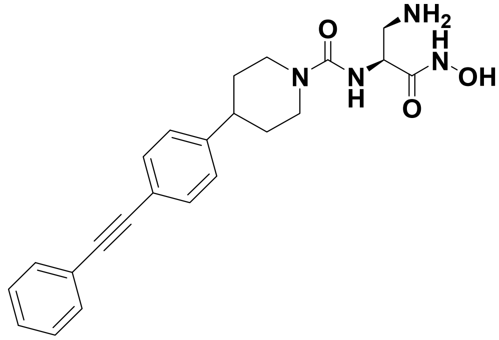 **29**	163.1	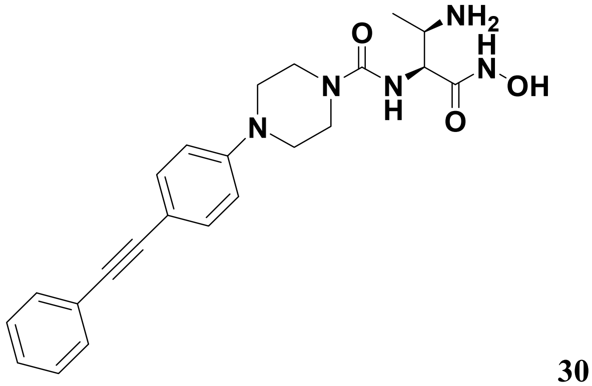 **30**	0.6
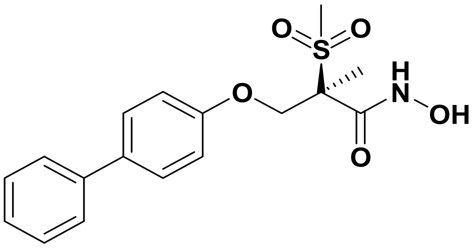 **31**	31.4	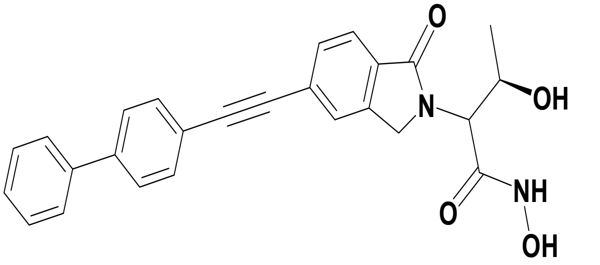 **32**	13
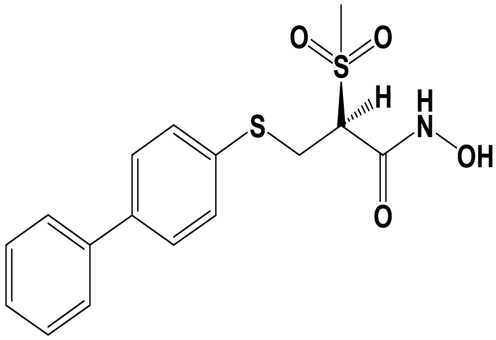 **33**	9.04	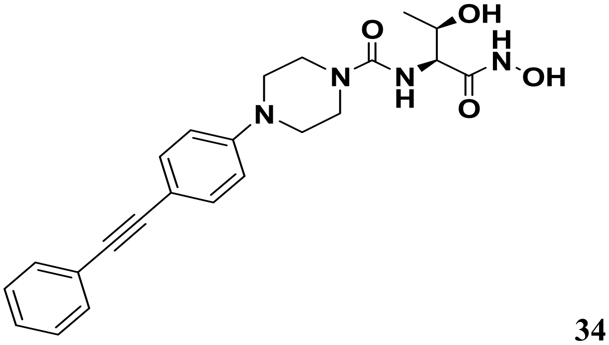 **34**	2.4

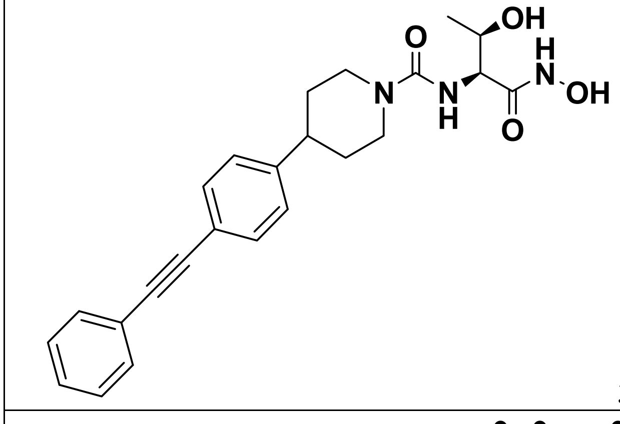 35	13.8	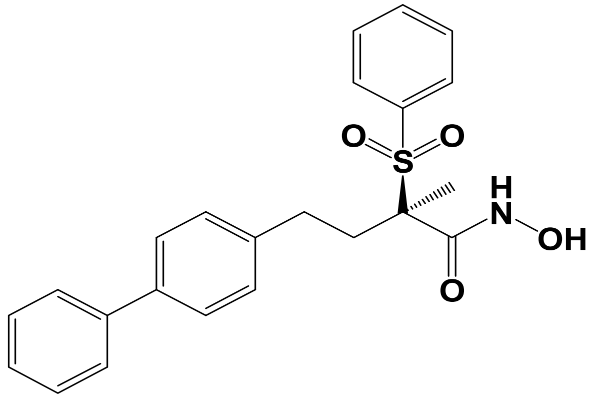 36	67.9
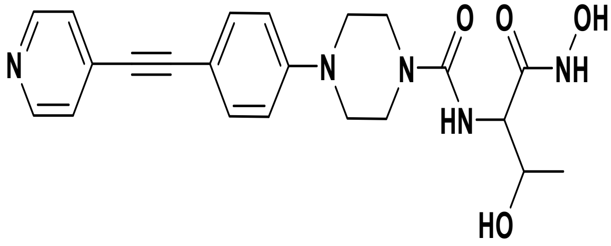 37	4	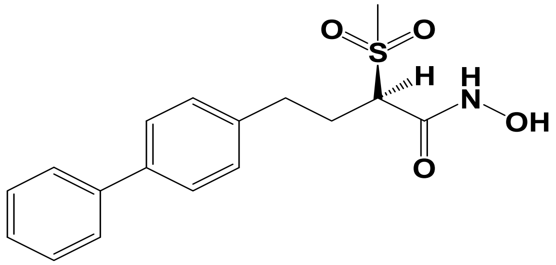 38	13.8
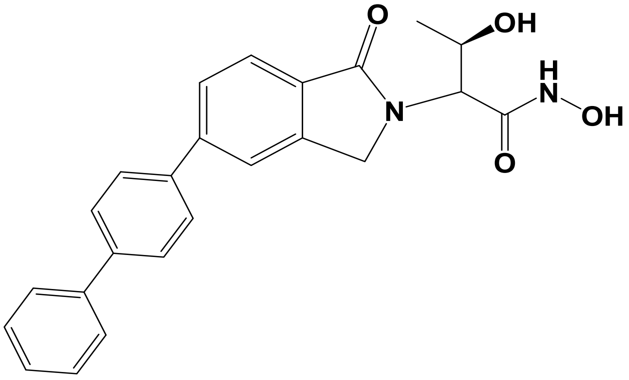 39	50	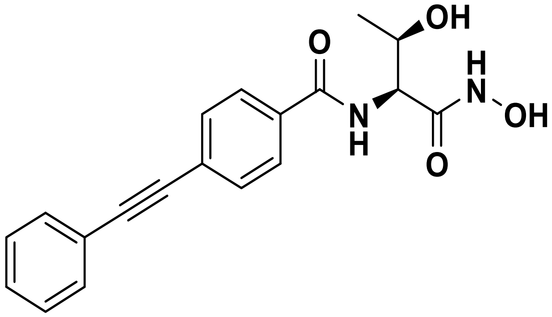 **CHIR-12** **40**	2
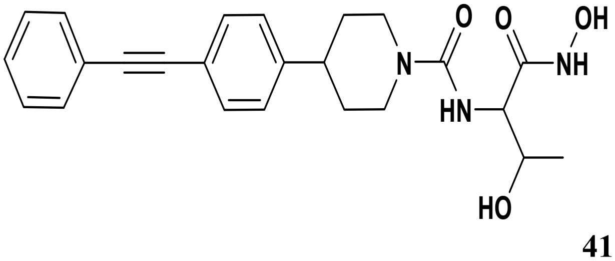 41	2.1	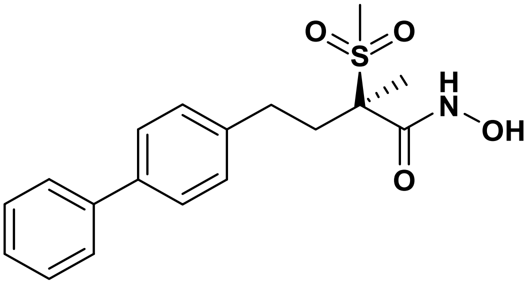 42	1.37

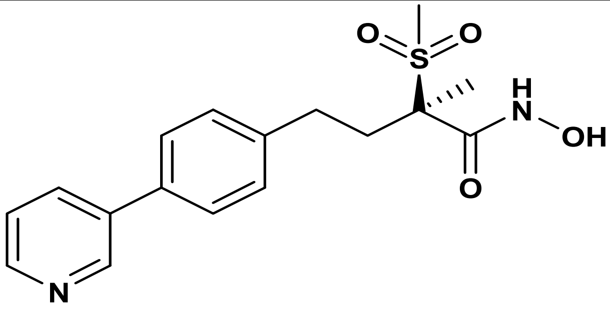 43	29.7	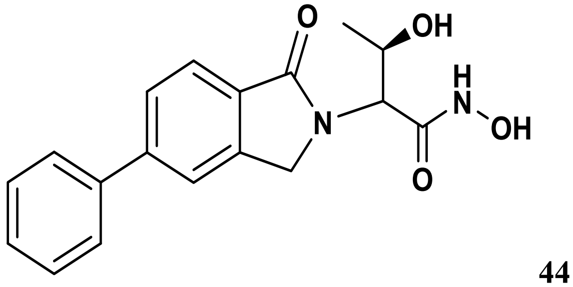 44	41
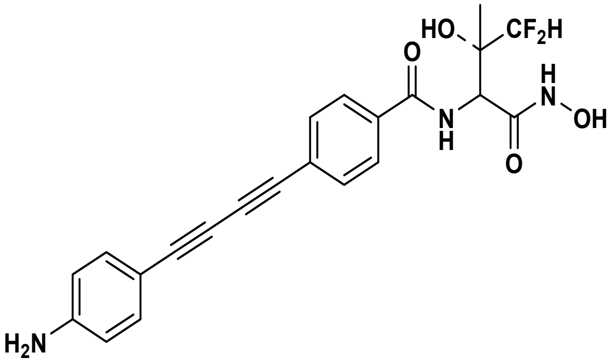 LPC-058 45	0.0035	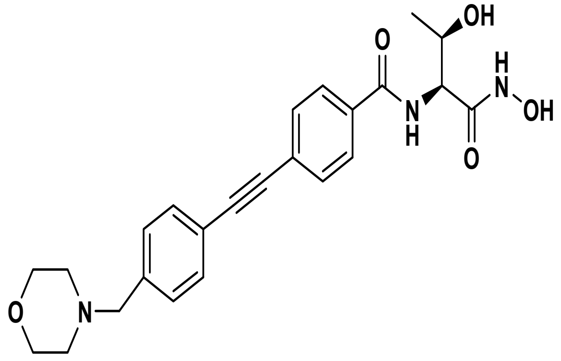 CHIR-90 46	2
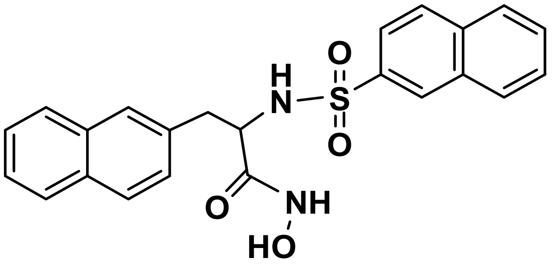 BB-78485 47	20	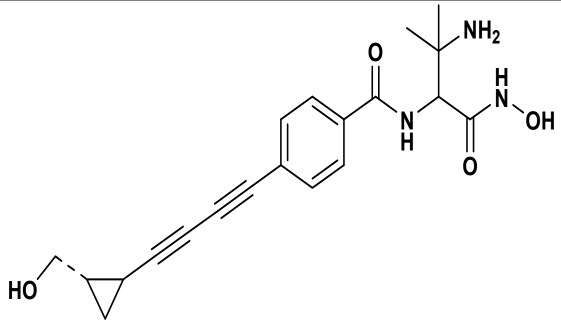 ACHN-975 48	0.02
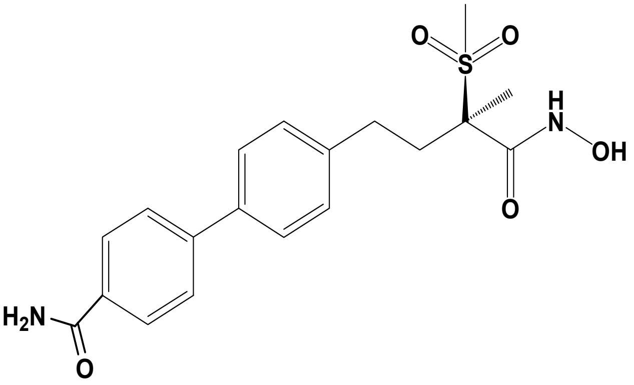 49	247	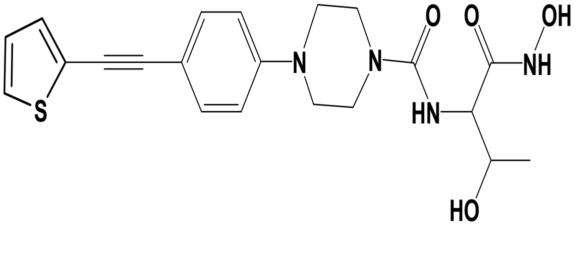 50	21

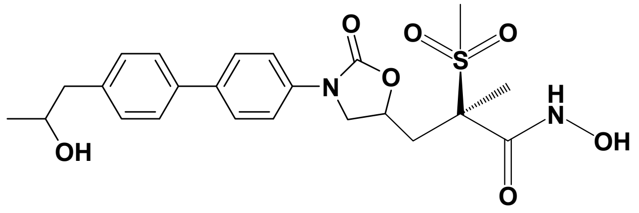 **51**	0.12	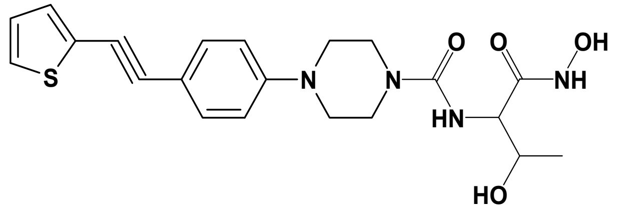 **52**	136
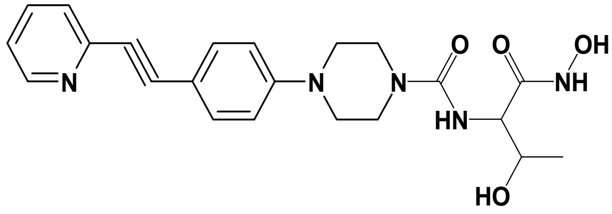 **53**	10	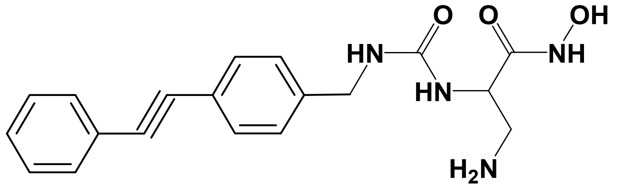 **54**	163.1
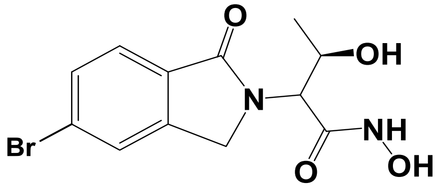 **55**	61	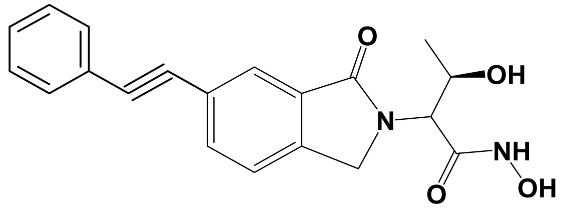 **56**	34
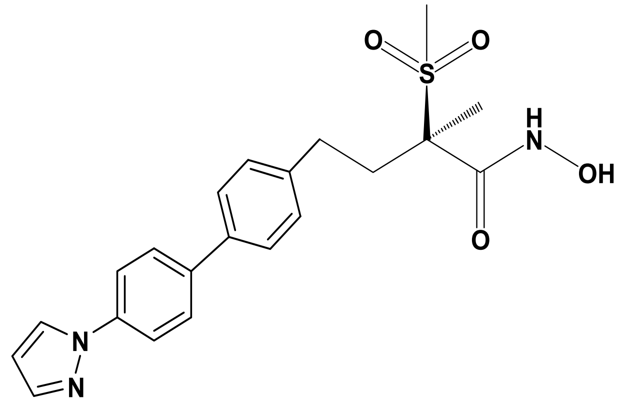 **57**	22.1	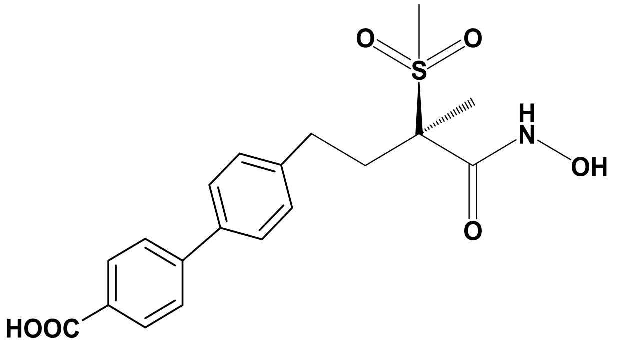 **58**	73.6
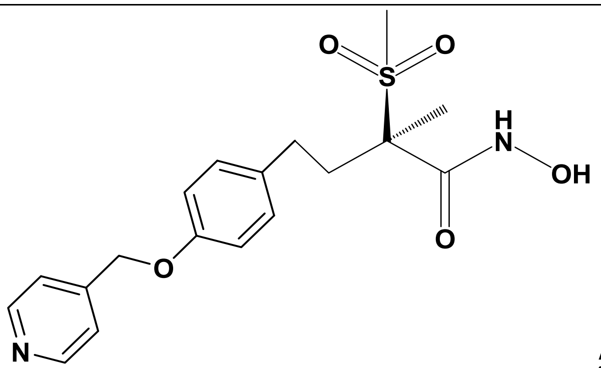 **59**	21.6	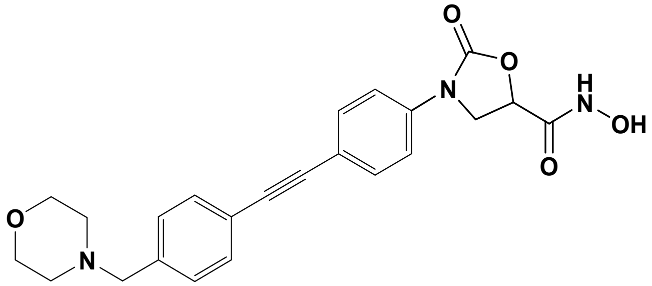 **60**	130

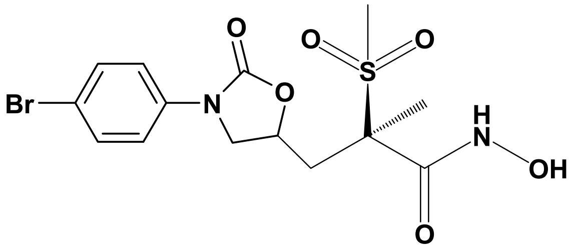 **61**	0.21	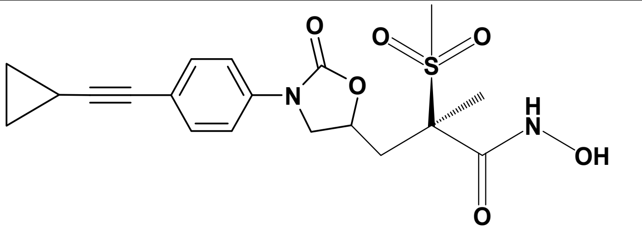 **62**	0.65
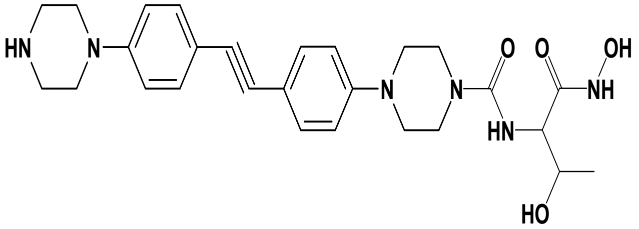 **63**	23.3	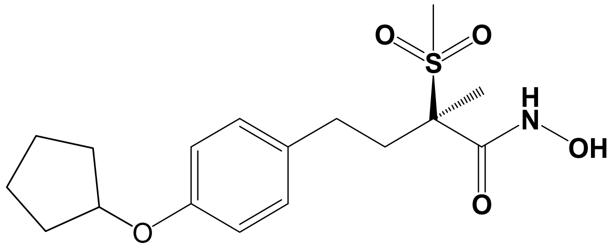 **64**	10.1
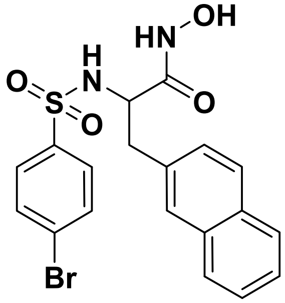 **BB-78484** **65**	400	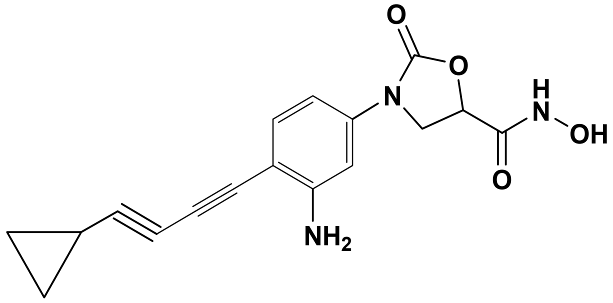 **66**	27

Table S15: Validation test set compounds.

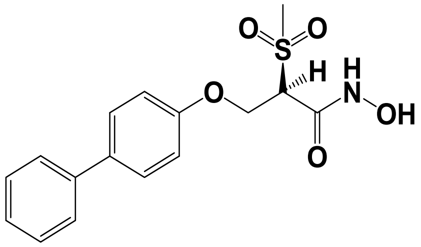 **67**	31.4	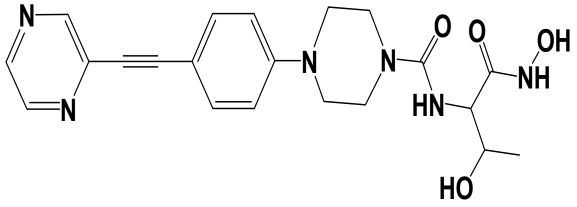 **68**	136
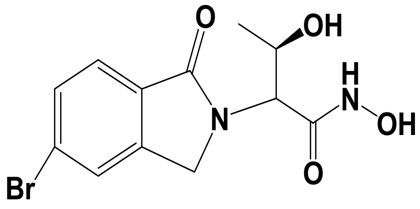 **69**	61	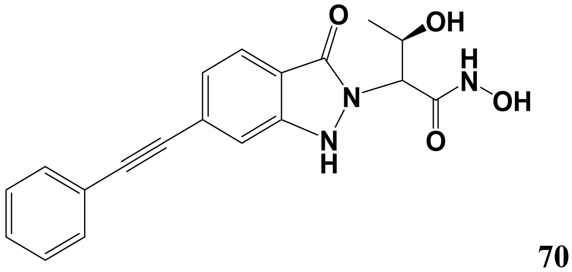 **70**	200
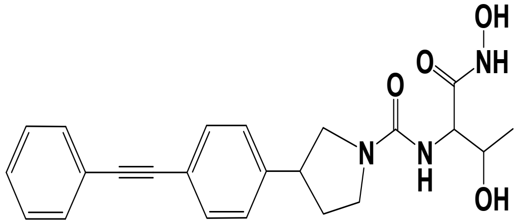 **71**	146.4	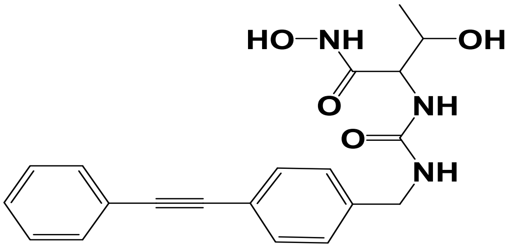 **72**	201.4
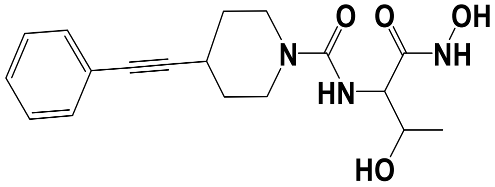 **73**	207	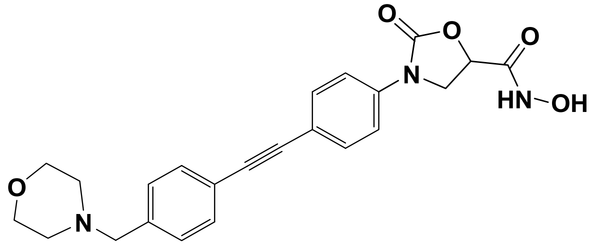 **74**	260
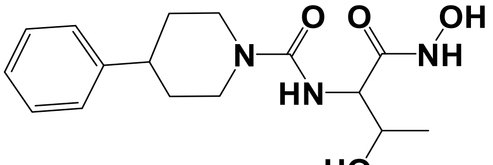 **75**	3944.5	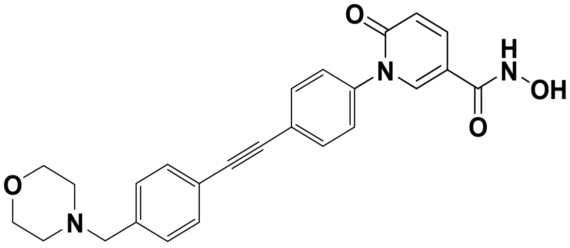 **76**	1000

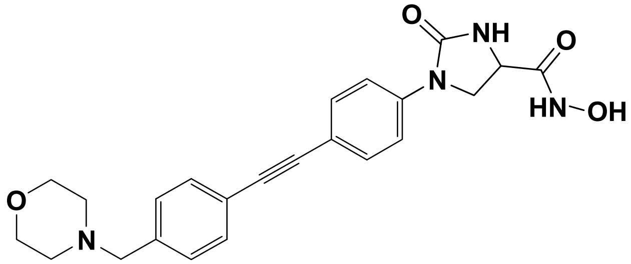 **77**	700	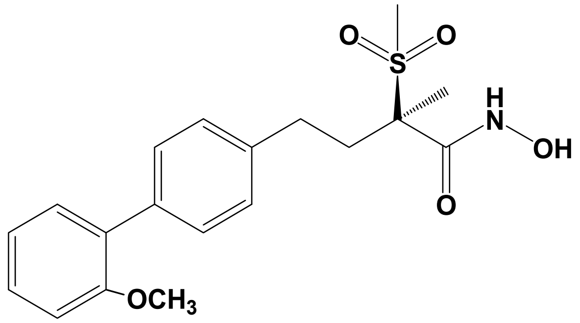 **78**	7.04
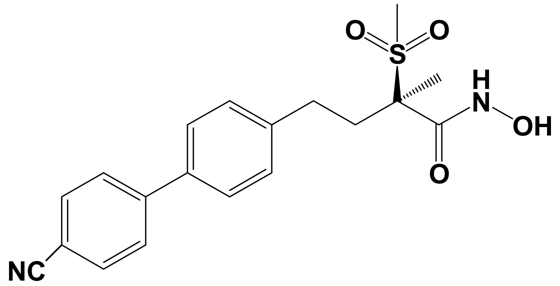 **79**	0.64	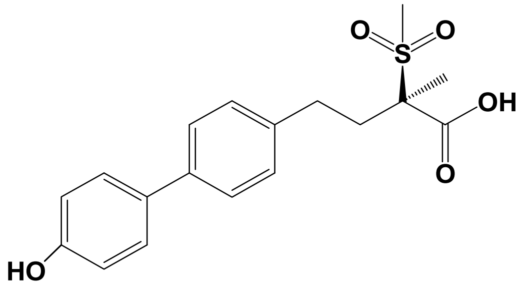 **80**	92.5
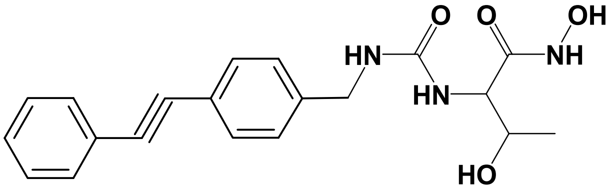 **81**	204.1	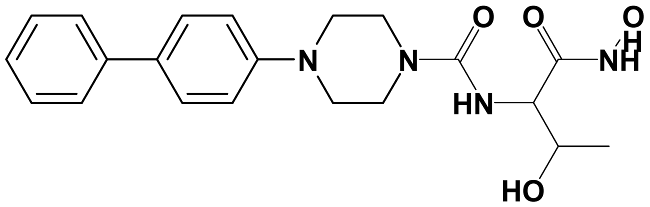 **82**	5.6
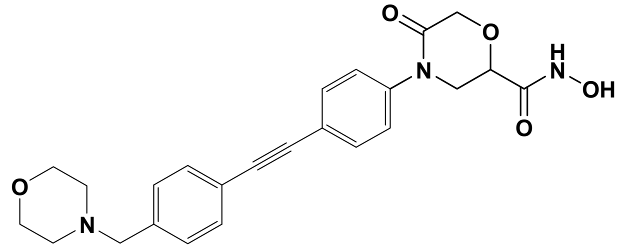 **83**	510		

Text S13: Cytotoxicity assay

Cell viability, in terms of mitochondrial metabolic function, was evaluated by the reduction of 3-(4,5-dimethyl-2-thiazolyl)-2,5-diphenyl-2-tetrazolium bromide (MTT) to insoluble formazan. Briefly, human neuroblastoma cells (SH-SY5Y, obtained from ATCC, order number CRL-2266) and human fetal lung fibroblasts (WI-38, obtained from ATCC, order number CCL-75) were seeded in a 96-well plate at 2×104 cells per well. Subsequently, cells were treated for 24 h with different concentrations of the studied compounds **8b** and **20b** (2.5-80 μM, selected as best candidates from enzyme inhibition assays). Then the treatment medium was replaced with MTT solution (0.5 mg ml^{-1}) in Hank's balanced salt solution (HBSS) for 2 h at 37 °C in 5% CO_2. After washing with HBSS, formazan crystals were dissolved in isopropanol. The amount of formazan was measured (λ = 570 nm, reference filter 690 nm) using a multilabel plate reader (VICTOR™ X3, PerkinElmer, Waltham, MA, USA) and an Anthos Zenyth 200rt microplate reader (Biochrom, UK). The cytotoxicity of the test compound was obtained using the following formula: $[(A - B)/A \times 100]$, where A represents the absorbance of untreated cells and B the absorbance of cells treated with different concentrations of the test compounds. Cytotoxic concentration for 50% of cells (IC_{50}) was determined by linear regression.

Text S14: Mechanism of action studies

***in vitro* enzyme inhibition**

Enzyme inhibition assays were performed using *E. coli* DNA gyrase and topoisomerase IV cleavage assay kits (Inspiralis®) according to the manufacturer's instructions[62]. The compounds with the highest activity against *E. coli* were selected to be tested on DNA gyrase, while the compounds with the highest activity against *S. aureus* were selected to be tested on topoisomerase IV. Compounds were diluted in DMSO and IC_{50} values were determined at a final concentration of 0.1, 1, 10, and 100 μM. Norfloxacin was used as a reference drug.

Metal complexation assay

Compounds were prepared as 30 μM solutions in methanol. Metals were prepared in varying concentrations from 15 to 35 μM in HEPES buffer (20 mM, pH 7.4)[63]. The absorption spectra of the compounds alone or in the presence of $MgCl_2$, $ZnCl_2$, and $CdCl_2$, respectively, were recorded at room temperature in a 1 cm quartz cell using UV-Visible Spectrophotometer (PG Instruments Limited, T80, United Kingdom). Additionally, the ratio of ligand/metal ion in the complex was determined by a molar ratio method[37], wherein fixed concentrations of the compounds (30 μM) were mixed with ascending concentrations of each metal (15–35 μM), and UV–vis absorption spectra were recorded.

Bacterial strains and growth conditions

Bacterial strain used in this study are listed in **Table S16**. *E. coli, S. aureus*, and *B. subtilis* were grown in Mueller Hinton broth, *P. aeruginosa* in cation-adjusted Muller Hinton II, and *M. tuberculosis* in Middlebrook 7H9 medium. *M. tuberculosis* was grown at 30 °C, all other strains at 37 °C. Expression of NeonGreen-GlpT in *E. coli* BCB472 was induced by addition of 20 μM isopropyl β- d-1-thiogalactopyranoside (IPTG) for 60 min. *B. subtilis* GFP-expressing strains were constantly grown in the presence of inducer (0.5% xylose for 2020, 0.3% xylose for MW10, 0.05% xylose for TNVS284, and 0.1% xylose for TNVS284, EKB46, and TNVS45).

Minimal inhibitory concentrations (MIC)

Minimal inhibitory concentrations against *E. coli, P. aeruginosa, S. aureus,* and *B. subtilis* were performed in a microdilution protocol according to CLSI guidelines as described previously[64, 65]

. Antimicrobial activity against *M. tuberculosis* was tested using a modified protocol according to[66].

Fluorescence microscopy

All microscopy performed on a Nikon Eclipse Ti2 equipped with a CFI Plan Apochromat DM Lambda 100X Oil objective (N.A. 1.45, W.D. 0.13mm), a Photometrics, PRIME BSI camera, a Lumencor Sola SE II FISH 365 light source, and an Okolab temperature incubation chamber. Images were obtained using the NIS elements AR software version 5.21.03 and analyzed with ImageJ and MicrobeJ [67, 68].

Bacterial cytological profiling (BCP)

BCP was performed using *E. coli* W3110 and *B. subtilis* DSM402. Strains were grown until an OD_{600} of 0.3 prior to treatment with 1xMIC of the respective compounds for 60 min. Samples were then stained with 1 µM FM6-64 and 1 µM DAPI for 5 min, spotted on agarose-covered microscopy slides as described previously[69], and observed by fluorescence microscopy. *E. coli* BCB472 was grown until and OD_{600} of 0.3 prior to addition of 20 µM IPTG and the respective compounds as described above. After 60 min, samples were withdrawn, spotted on agarose-covered slides, and microscopically examined.

Membrane potential measurements

Membrane potential was assessed with DiSC(3)5 as described previously[70]. In short, *B. subtilis* DSM402 was grown in presence of 50 µg/mL bovine albumin serum (BSA) and after reaching an OD_{600} of 0.3, 1 µM DiSC(3)5 was added to the cells. Antibiotics were added after the fluorescence baseline was stable and fluorescence was monitored over 30 min in a BMG Clariostar Plus plate reader at an excitation wavelength of 610-30 nm and an emission wavelength of 675-50 nm.

Checkerboard assays

Checkerboard assays were performed with *E. coli* W3110 according to[71]. The fractional inhibitory concentration index was calculated according to the formula FICI = $(MIC_A^{combi}/MIC_A^{alone}) + (MIC_B^{combi}/MIC_B^{alone})$. FICI values of ≤0.5 were defined as synergy, >0.5 to ≤4.0 as additive (no interaction), and >4.0 was defined as antagonism. Checkerboard assays were performed in duplicate.

LpxC overexpression assay

To assess LpxC as possible target, MICs were determined against a strain overexpressing the *lpxC* gene from the arabinose-inducible P_{BAD} promoter. To this end, *E. coli* BL21 DE03 carrying either pBO110 (P_{BAD}-*lpxC*) or pBAD24 (empty vector control)[72] were grown in presence of 100 µg/mL ampicillin to ensure plasmid maintenance. MICs were determined in Muller Hinton broth containing 0, 0.005, 0.01, or 0.05% arabinose. If LpxC is a target of the compound, the MIC should increase with rising arabinose concentrations due to the presence of more target molecules. As positive control, the specific LpxC inhibitor ACHN-975 was used[73]. As additional controls for the specificity of the assay, the outer membrane-permeabilizing lipopeptide polymyxin B and the reactive species-forming pro-drug nitrofurantoin were included.

Acetic acid/methanol fixation

Peptidoglycan integrity was tested in *B. subtilis* DSM402 using a previously published protocol [74, 75]. In short, *B. subtilis* was grown to an OD_{600} of 0.3, treated with antibiotics for 10 and 60 min as specified in the corresponding figure legends, and subsequently fixed in a 1:3 mixture of acetic acid and methanol. Samples were observed by phase contrast microscopy.

Membrane protein localization

GFP-expressing *B. subtilis* strains MW10 (GFP-MreB), TNVS175 (MurG-msfGFP), TNVS284 (MraY-msfGFP), EKB46 (msfGFP-PbpB), and TNVS45 (mGFP-PonA) were grown until early log phase in Muller Hinton broth supplemented with appropriate concentrations of xylose (see above). Cells were treated with 1x MIC of the respective compounds for 30 min (vancomycin) or 1 h (all other compounds) prior to microscopy. TNVS45, which showed a spotty localization with some compounds was additionally stained with FM4-64 to visualize co-localization with membrane patches. Samples were spotted on agarose-covered microscopy slides and observed by fluorescence microscopy. In the case of MreB, two separate images if the same field of view were recorded in a 30 sec interval and overlaid in ImageJ to visualize MreB mobility. A perfect overlap (yellow) indicates stalled MreB movement while distinct red and green spots are indicative of MreB mobility.

Table S16: Strains used in this study. i. a. = if applicable, *mgfp* = monomeric green-fluorescent protein, *msfgfp* = monomeric superfolder green-fluorescent protein, #Ciprofloxacin=R, *Nitrofurantoin=R, Cefadroxil=R, Penicillin G/V=R, Isoxa-pc=R, Cefuroxim=R, Cefotaxim=R, Ceftazidim=R, Imipenem=R, Tobramycin=R, Trim-Sulfa=R, Norfloxacin=R, Ciprofloxacin=R, Clindamycin=R, Fusidic acid=S, Vancomycin=S, Netilmic=R

**Species and strain**	**Relevant genotype**	**Reference**
E. coli W3110	*F-, IN(rrnD-rrnE)1*	https://doi.org/10.13145/bacdive4747.20201210.5
*E. coli**		clinical resistant isolate
E. coli BCB472	*psav057-NeonGreen-2GS-GlpT*	[76]
E. coli BL21DE03 pBAD24	P_{BAD}, *araC, rrnBT,* Amp^r	[77]
E. coli BL21DE03 pBO110	P_{BAD}-*lpxC*	[72]
K. pneumoniae ATCC10031		doi:10.13145/bacdive4968.20220920.7
P. aeruginosa PAO1		doi.org/10.13145/bacdive12801.20201210.5
S. aureus CCUG1800T		doi.org/10.13145/bacdive14487.20201210.5
S. aureus ATCC43300#		doi:10.13145/bacdive14464.20220920.7
M. tuberculosis MC26020	Δ*lysA* Δ*panCD*	[78]
B. subtilis 2020	*amyE::spc Pxyl-gfp-ftsZ*	[79]
B. subtilis DSM402	*trpC2*	doi.org/10.13145/bacdive1156.20201210.5
B. subtilis EKB46	*trpC2 amyE::spc Pxyl-msfgfp*	[80]
B. subtilis MW10	*trpC2 amyE::spc Pxyl-gfp-mreB*	[80]
B. subtilis TNVS45	*trpC2 amyE::spc Pxyl-mgfp-ponA*	[80]
B. subtilis TNVS175	*trpC2 amyE::spc Pxyl-murG-msfgfp*	[80]
B. subtilis TNVS284	*trpC2 amyE::spc Pxyl-mraY-msfgfp*	[80]

7. References

Text S15: Supplementary references

[1] W. Jun, S. Yumin, Y. Heng, Recent advances in quinolone hybrids with potential antibacterial activity against drug-resistant bacteria, Future medicinal chemistry 15(6) (2023) 555-578.
[2] H.K. Swedan, A.E. Kassab, E.M. Gedawy, S.E. Elmeligie, Topoisomerase II inhibitors design: Early studies and new perspectives, Bioorg Chem 136 (2023) 106548.
[3] H.H.H. Mohammed, D.M.E. Ali, M. Badr, A.G.K. Habib, A.M. Mahmoud, S.M. Farhan, S. Gany, S.A. Mohamad, A.M. Hayallah, S.H. Abbas, G.E.A. Abuo-Rahma, Synthesis and molecular docking of new N4-piperazinyl ciprofloxacin hybrids as antimicrobial DNA gyrase inhibitors, Mol Divers 27(4) (2023) 1751-1765.
[4] S.N. Pandeya, D. Sriram, P. Yogeeswari, S. Ananthan, Antituberculous activity of norfloxacin mannich bases with isatin derivatives, Chemotherapy 47(4) (2001) 266-9.
[5] S.N. Pandeya, D. Sriram, G. Nath, E. De Clercq, Synthesis, antibacterial, antifungal and anti-HIV activities of norfloxacin mannich bases, European journal of medicinal chemistry 35(2) (2000) 249-55.
[6] K. Khan, K. Khan, R. Siddiqui, N. Ambreen, N. Sultana, S. Tauseef, A. Ahmad, S. Perveen, P. Dr, H. Khan, Synthesis, antibacterial and antifungal evaluation of norfloxacin derivatives, journal of pharmacy research 55 (2012) 92.
[7] S.N. Pandeya, B.N. Singh, S.K. Shukla, M. Singh, Synthesis and antimicrobial activity of N-norfloxacin Mannich bases of isatin and its derivatives, Asian Journal of Chemistry 20 (2008) 5377-5382.
[8] M.A. Abdullah, G.E. Abuo-Rahma, E.M. Abdelhafez, H.A. Hassan, R.M. Abd El-Baky, Design, synthesis, molecular docking, anti-Proteus mirabilis and urease inhibition of new fluoroquinolone carboxylic acid derivatives, Bioorg Chem 70 (2017) 1-11.
[9] G.G. Rajulu, H.S. Bhojya Naik, A. Viswanadhan, J. Thiruvengadam, K. Rajesh, S. Ganesh, H. Jagadheshan, P.K. Kesavan, New Hydroxamic Acid Derivatives of Fluoroquinolones: Synthesis and Evaluation of Antibacterial and Anticancer Properties, Chemical and Pharmaceutical Bulletin 62(2) (2014) 168-175.
[10] U. Jacquemard, V. Bénéteau, M. Lefoix, S. Routier, J.-Y. Mérour, G. Coudert, Mild and selective deprotection of carbamates with Bu4NF, Tetrahedron 60(44) (2004) 10039-10047.
[11] D.F. Veber, S.R. Johnson, H.-Y. Cheng, B.R. Smith, K.W. Ward, K.D. Kopple, Molecular Properties That Influence the Oral Bioavailability of Drug Candidates, Journal of Medicinal Chemistry 45(12) (2002) 2615-2623.
[12] C.A. Lipinski, F. Lombardo, B.W. Dominy, P.J. Feeney, Experimental and computational approaches to estimate solubility and permeability in drug discovery and development settings1PII of original article: S0169-409X(96)00423-1. The article was originally published in Advanced Drug Delivery Reviews 23 (1997) 3–25.1, Advanced Drug Delivery Reviews 46(1) (2001) 3-26.
[13] I.M. Vlad, D.C. Nuta, C. Chirita, M.T. Caproiu, C. Draghici, F. Dumitrascu, C. Bleotu, S. Avram, A.M. Udrea, A.V. Missir, L.G. Marutescu, C. Limban, In Silico and In Vitro Experimental Studies of New Dibenz[b,e]oxepin-11(6H)one O-(arylcarbamoyl)-oximes Designed as Potential Antimicrobial Agents, Molecules (Basel, Switzerland) 25(2) (2020).

[14] P. Ertl, B. Rohde, P. Selzer, Fast calculation of molecular polar surface area as a sum of fragment-based contributions and its application to the prediction of drug transport properties, Journal of Medicinal Chemistry 43(20) (2000) 3714-3717.
[15] S. O'Hagan, D.B. Kell, The apparent permeabilities of Caco-2 cells to marketed drugs: magnitude, and independence from both biophysical properties and endogenite similarities, PeerJ 3 (2015) e1405.
[16] https://readycell.com/caco-2-permeability-protocol/, (Accessed at January 5,. 2022.).
[17] J. Yates, P. Arundel, On the Volume of Distribution at Steady State and Its Relationship With Two-Compartmental Models, Journal of Pharmaceutical Sciences 97 (2008) 111-122.
[18] A. Mansoor, N. Mahabadi, Volume of Distribution, StatPearls, StatPearls Publishing

Copyright © 2021, StatPearls Publishing LLC., Treasure Island (FL), 2021.
[19] T.S. Carpenter, D.A. Kirshner, E.Y. Lau, S.E. Wong, J.P. Nilmeier, F.C. Lightstone, A method to predict blood-brain barrier permeability of drug-like compounds using molecular dynamics simulations, Biophysical journal 107(3) (2014) 630-641.
[20] S.K. Bardal, J.E. Waechter, D.S. Martin, Chapter 2 - Pharmacokinetics, in: S.K. Bardal, J.E. Waechter, D.S. Martin (Eds.), Applied Pharmacology, W.B. Saunders, Philadelphia, 2011, pp. 17-34.
[21] J.M. Collins, Chapter 46 - Pharmacokinetics, Pharmacodynamics, and Pharmacogenetics1, in: J. Mendelsohn, P.M. Howley, M.A. Israel, J.W. Gray, C.B. Thompson (Eds.), The Molecular Basis of Cancer (Third Edition), W.B. Saunders, Philadelphia, 2008, pp. 547-552.
[22] L.C.a.R.I.o.D.-I.L.I.I.B.M.N.I.o.D.a.D.a.K.D.-N.U. 2020 Mar 10]. Available from: https://www.ncbi.nlm.nih.gov/books/NBK547850/, (Accessed at January 6,. 2022.).
[23] E. Adikwu, Fluoroquinolones Reported Hepatotoxicity, Pharmacology & Pharmacy 03 (2012) 328-336.
[24] A.K. Jain, D. Singh, K. Dubey, R. Maurya, S. Mittal, A.K. Pandey, Chapter 3 - Models and Methods for In Vitro Toxicity, in: A. Dhawan, S. Kwon (Eds.), In Vitro Toxicology, Academic Press2018, pp. 45-65.
[25] http://www.swissadme.ch/, (Accessed at January 12,. 2022.).
[26] J.B.T.D.J. Taylor, Comprehensive medicinal chemistry II, (2007).
[27] A. Zerroug, S. Belaidi, I. BenBrahim, L. Sinha, S. Chtita, Virtual screening in drug-likeness and structure/activity relationship of pyridazine derivatives as Anti-Alzheimer drugs, Journal of King Saud University - Science 31(4) (2019) 595-601.
[28] https://www.rcsb.org/structure/5CDQ, ((Accessed at March 27,. 2022)).
[29] https://www.rcsb.org/structure/2XKK, ((Accessed at March 23,.2022)).
[30] A. Wohlkonig, P.F. Chan, A.P. Fosberry, P. Homes, J. Huang, M. Kranz, V.R. Leydon, T.J. Miles, N.D. Pearson, R.L. Perera, A.J. Shillings, M.N. Gwynn, B.D. Bax, Structural basis of quinolone inhibition of type IIA topoisomerases and target-mediated resistance, Nature structural & molecular biology 17(9) (2010) 1152-3.
[31] https://www.rcsb.org/structure/6fv4 (Accessed at November 5,. 2021).
[32] https://www.rcsb.org/structure/6MOD, (Accessed at January 10,. 2022.).
[33] F. Cohen, J.B. Aggen, L.D. Andrews, Z. Assar, J. Boggs, T. Choi, P. Dozzo, A.N. Easterday, C.M. Haglund, D.J. Hildebrandt, M.C. Holt, K. Joly, A. Jubb, Z. Kamal, T.R. Kane, A.W. Konradi, K.M. Krause, M.S. Linsell, T.D. Machajewski, O. Miroshnikova, H.E. Moser, V. Nieto, T. Phan, C. Plato, A.W. Serio, J. Seroogy, A. Shakhmin, A.J. Stein, A.D. Sun, S. Sviridov, Z. Wang, K. Wlasichuk, W. Yang, X. Zhou, H. Zhu, R.T. Cirz, Optimization of LpxC Inhibitors for Antibacterial Activity and Cardiovascular Safety, ChemMedChem 14(16) (2019) 1560-1572.

[34] L. Baum, A. Ng, Curcumin interaction with copper and iron suggests one possible mechanism of action in Alzheimer's disease animal models, Journal of Alzheimer's disease : JAD 6 (2004) 367-77; discussion 443.
[35] T. Mayerhöfer, S. Pahlow, J. Popp, The Bouguer-Beer-Lambert Law: Shining Light on the Obscure, ChemPhysChem 21 (2020).
[36] D.F. Swinehart, The Beer-Lambert Law, Journal of Chemical Education 39(7) (1962) 333.
[37] M.L. Bolognesi, A. Cavalli, L. Valgimigli, M. Bartolini, M. Rosini, V. Andrisano, M. Recanatini, C. Melchiorre, Multi-target-directed drug design strategy: from a dual binding site acetylcholinesterase inhibitor to a trifunctional compound against Alzheimer's disease, J Med Chem 50(26) (2007) 6446-9.
[38] W. Zheng, J. Li, Z. Qiu, Z. Xia, W. Li, L. Yu, H. Chen, J. Chen, Y. Chen, Z. Hu, W. Zhou, B. Shao, Y. Cui, Q. Xie, H. Chen, Novel bis-(-)-nor-meptazinol derivatives act as dual binding site AChE inhibitors with metal-complexing property, Toxicology and applied pharmacology 264(1) (2012) 65-72.
[39] V. Uivarosi, Metal complexes of quinolone antibiotics and their applications: an update, Molecules (Basel, Switzerland) 18(9) (2013) 11153-97.
[40] A.A.M. Mohammed, G. Suaifan, M.B. Shehadeh, P.N. Okechukwu, Design, synthesis and antimicrobial evaluation of novel glycosylated-fluoroquinolones derivatives, European journal of medicinal chemistry 202 (2020) 112513.
[41] G. Marc, C. Araniciu, S.D. Oniga, L. Vlase, A. Pîrnău, G.C. Nadăș, C.Ș. Novac, I.A. Matei, M.C. Chifiriuc, L. Măruțescu, O. Oniga, Design, Synthesis and Biological Evaluation of New Piperazin-4-yl-(acetyl-thiazolidine-2,4-dione) Norfloxacin Analogues as Antimicrobial Agents, Molecules (Basel, Switzerland) 24(21) (2019).
[42] Z. Yu, G. Shi, Q. Sun, H. Jin, Y. Teng, K. Tao, G. Zhou, W. Liu, F. Wen, T. Hou, Design, synthesis and in vitro antibacterial/antifungal evaluation of novel 1-ethyl-6-fluoro-1,4-dihydro-4-oxo-7(1-piperazinyl)quinoline-3-carboxylic acid derivatives, European journal of medicinal chemistry 44(11) (2009) 4726-33.
[43] https://pubchem.ncbi.nlm.nih.gov/compound/15993159, (Accessed on March 23,.2021).
[44] M.J. Nieto, F.d.L. Alovero, R.H. Manzo, M.R. Mazzieri, A new class of fluoroquinolones: benzenesulfonamidefluoroquinolones (BSFQs), antibacterial activity and SAR studies, European journal of medicinal chemistry 34(3) (1999) 209-214.
[45] P.O. Venkataramana Reddy, S. Mishra, M.P. Tantak, K. Nikhil, R. Sadana, K. Shah, D. Kumar, Design, synthesis and in vitro cytotoxicity studies of novel β-carbolinium bromides, Bioorg Med Chem Lett 27(6) (2017) 1379-1384.
[46] A. Abdelrahman, S.G. Yerande, V. Namasivayam, T.A. Klapschinski, M.W. Alnouri, A. El-Tayeb, C.E. Müller, Substituted 4-phenylthiazoles: Development of potent and selective A1, A3 and dual A1/A3 adenosine receptor antagonists, European journal of medicinal chemistry 186 (2020) 111879.
[47] B. Han, Z. Zheng, D. Zheng, L. Zhang, P. Cui, J. Shi, C. Li, Application of poly(vinylphenyltrimethylammonium tribromide) resin as an efficient polymeric brominating agent in the α-bromination and α-bromoacetalization of acetophenones, Synthetic Communications 49(19) (2019) 2512-2520.
[48] N. Mayer, M. Schweiger, E. Fuchs, A.K. Migglautsch, C. Doler, G.F. Grabner, M. Romauch, M.-C. Melcher, R. Zechner, R. Zimmermann, R. Breinbauer, Structure-activity relationship studies for the development of inhibitors of murine adipose triglyceride lipase (ATGL), Bioorganic & medicinal chemistry 28(16) (2020) 115610.

[49] B. Rammurthy, P. Swamy, M. Naresh, K. Srujana, C. Durgaiah, G. Krishna Sai, N. Narender, A new and versatile one-pot strategy to synthesize alpha-bromoketones from secondary alcohols using ammonium bromide and oxone, New Journal of Chemistry 41(10) (2017) 3710-3714.
[50] S.A. Gamal El-din Abuo-rahma , Mai Shoman , Ebtihal Samır , Rehab Abdel-baky, New N - 4 piperazinyl derivatives of norfloxacin: design, synthesis, and correlation of calculated physicochemical parameters with antibacterial activity, Turkish Journal of Chemistry 42 (4) (2018) 1072 - 1085.
[51] H. Koga, A. Itoh, S. Murayama, S. Suzue, T. Irikura, Structure-activity relationships of antibacterial 6,7- and 7,8-disubstituted 1-alkyl-1,4-dihydro-4-oxoquinoline-3-carboxylic acids, J Med Chem 23(12) (1980) 1358-63.
[52] V. Merino, J. Freixas, M. del Val Bermejo, T.M. Garrigues, J.M. Plá-Delfina, J. Moreno, Biophysical Models as an Approach To Study Passive Absorption in Drug Development: 6-Fluoroquinolones, Journal of Pharmaceutical Sciences 84(6) (1995) 777-782.
[53] S. Oniga, M. Palage, C. Araniciu, G. Marc, O. Ovidiu, L. Vlase, V. Prisăcari, V. Valica, S. Curlat, U. Livia, Design, synthesis, molecular docking, and antibacterial activity evaluation of some novel norfloxacin analogues, Farmacia 66 (2018) 1048.
[54] L.L.Z.H.W.H.H. Guoqiang, Zhengzhou Industry Applied Technology College Location in patent: Paragraph 0031; 0032; 0034; 0046; 0048 (CN109400627, 2019, A).
[55] A. Singh, K. Raghuwanshi, V. Patel, D. Jain, R. Veerasamy, A. Dixit, H. Rajak, Assessment of 5-substituted Isatin as Surface Recognition Group: Design, Synthesis, and Antiproliferative Evaluation of Hydroxamates as Novel Histone Deacetylase Inhibitors, Pharmaceutical Chemistry Journal 51 (2017) 1-9.
[56] H.-Q. Liu, D.-C. Wang, F. Wu, W. Tang, P.-K. Ouyang, Synthesis and biological evaluation of 5′-phenyl-3′H-spiro-[indoline-3,2′-[1,3,4]oxadiazol]-2-one analogs, Chinese Chemical Letters 24(10) (2013) 929-933.
[57] P. Sai Prathima, R. Bikshapathi, V.J. Rao, Synthesis of isatin derivatives under metal free conditions using hypervalent iodine, Tetrahedron Letters 56(46) (2015) 6385-6388.
[58] R. Shrestha, G.J. Lee, Y.R. Lee, Synthesis of diverse isatins via ring contraction of 3-diazoquinoline-2,4-diones, RSC Advances 6(68) (2016) 63782-63787.
[59] D. Abuo-Rahma Gel, H.A. Sarhan, G.F. Gad, Design, synthesis, antibacterial activity and physicochemical parameters of novel N-4-piperazinyl derivatives of norfloxacin, Bioorganic & medicinal chemistry 17(11) (2009) 3879-86.
[60] A.A. Abdel-Aziz, A.S. El-Azab, A.M. Alanazi, Y.A. Asiri, I.A. Al-Suwaidan, A.R. Maarouf, R.R. Ayyad, T.Z. Shawer, Synthesis and potential antitumor activity of 7-(4-substituted piperazin-1-yl)-4-oxoquinolines based on ciprofloxacin and norfloxacin scaffolds: in silico studies, Journal of enzyme inhibition and medicinal chemistry 31(5) (2016) 796-809.
[61] http://biosig.unimelb.edu.au/pkcsm/prediction, (Accessed at January 8,. 2022.).
[62] M.R. Burrell, N.P. Burton, A. Maxwell, A high-throughput assay for DNA topoisomerases and other enzymes, based on DNA triplex formation, Methods in molecular biology (Clifton, N.J.) 613 (2010) 257-66.
[63] Y.R.P.H.S.S.M. Farhat, pH 7.4.” Protocol Place. Dec 2013. <http://protocol-place.com>, (Accessed on 5/20/2022.).
[64] C. Performance Standards for Antimicrobial Susceptibility Testing; 23rd Informational Supplement M100-S23. 2013, Wayne, PA, USA.

[65] C.A. Albada HB, Wenzel M, Penkova M, Bandow JE, Sahl HG, Metzler-Nolte N, Modulating the activity of short arginine-tryptophan containing antibacterial peptides with N-terminal metallocenoyl groups., Beilstein J Org Chem 8 (2012) 1753–1764.
[66] W.J. Schön T, Machado D, Borroni E, Wijkander M, Lina G, Mouton J, Matuschek E, Kahlmeter G, Giske C, Santin M, Cirillo DM, Viveiros M, Cambau E, Antimicrobial susceptibility testing of Mycobacterium tuberculosis complex isolates; the EUCAST broth microdilution reference method for MIC determination. , Clinical microbiology and infection : the official publication of the European Society of Clinical Microbiology and Infectious Diseases 26 (2020) 1488–1492.
[67] A.-C.I. Schindelin J, Frise E, Kaynig V, Longair M, Pietzsch T, Preibisch S, Rueden C, Saalfeld S, Schmid B, Tinevez JY, White DJ, Hartenstein V, Eliceiri K, Tomancak P, Cardona A, Fiji: An open-source platform for biological-image analysis. Nat Methods 9 (2012) 676–682.
[68] A. Ducret, E.M. Quardokus, Y.V. Brun, MicrobeJ, a tool for high throughput bacterial cell detection and quantitative analysis, Nature microbiology 1(7) (2016) 16077.
[69] G.D. Winkel JD, Seistrup KH, Hamoen LW, Strahl H, Analysis of antimicrobial-triggered membrane depolarisation using voltage sensitive dyes, Front Cell Dev Biol 4:29 (2016).
[70] J.D. Te Winkel, D.A. Gray, K.H. Seistrup, L.W. Hamoen, H. Strahl, Analysis of Antimicrobial-Triggered Membrane Depolarization Using Voltage Sensitive Dyes, Front Cell Dev Biol 4 (2016) 29.
[71] H.K. Schmidt S, Melzig MF, Bereswill S, Heimesaat MM, Glycyrrhizic Acid Decreases Gentamicin-Resistance in Vancomycin-Resistant Enterococci, Planta Med 82 (2016) 1540–1545.
[72] F. Führer, S. Langklotz, F. Narberhaus, The C-terminal end of LpxC is required for degradation by the FtsH protease, Mol Microbiol 59(3) (2006) 1025-36.
[73] K.M. Krause, C.M. Haglund, C. Hebner, A.W. Serio, G. Lee, V. Nieto, F. Cohen, T.R. Kane, T.D. Machajewski, D. Hildebrandt, C. Pillar, M. Thwaites, D. Hall, L. Miesel, M. Hackel, A. Burek, L.D. Andrews, E. Armstrong, L. Swem, A. Jubb, R.T. Cirz, Potent LpxC Inhibitors with In Vitro Activity against Multidrug-Resistant Pseudomonas aeruginosa, Antimicrob. Agents Chemother. 63(11) (2019).
[74] K.T. Schneider T, Wimmer R, Wiedemann I, Sass V, Pag U, Jansen A, Nielsen AK, Mygind PH, Raventós DS, Neve S, Ravn B, Bonvin AMJJ, De Maria L, Andersen AS, Gammelgaard LK, Sahl H-GH-G, Kristensen H-HH-H, Raventos DS, Neve S, Ravn B, Bonvin AMJJ, De Maria L, Andersen AS, Gammelgaard LK, Sahl H-GH-G, Kristensen H-HH-H, Plectasin, a fungal defensin, targets the bacterial cell wall precursor Lipid II, Science 328 (2010) 1168–1172.
[75] K.B. Wenzel M, Münch D, Raatschen N, Albada HB, Hamoen L, Metzler-Nolte N, Sahl HG, Bandow JE, Proteomic response of Bacillus subtilis to lantibiotics reflects differences in interaction with the cytoplasmic membrane, Antimicrob Agents Chemother 56 (2012) 5749–5757.
[76] M.N. Liu X, Bouhss A, den Blaauwen T, FtsW activity and lipid II synthesis are required for recruitment of MurJ to midcell during cell division in Escherichia coli. , Mol Microbiol 109 (2018) 855–884.
[77] L.M. Guzman, D. Belin, M.J. Carson, J. Beckwith, Tight regulation, modulation, and high-level expression by vectors containing the arabinose PBAD promoter, Journal of bacteriology 177(14) (1995) 4121-30.
[78] B.K. Larsen MH, Chen B, Hsu T, Sambandamurthy VK, Lackner AA, Aye PP, Didier P, Huang D, Shao L, Wei H, Letvin NL, Frothingham R, Haynes BF, Chen ZW, Jacobs Jr WR, Efficacy and safety of live attenuated persistent and rapidly cleared Mycobacterium tuberculosis vaccine candidates in non-human primates. , Vaccine 27 (2009) 4709–4717.

[79] P. Gamba, J.-W. Veening, N.J. Saunders, L.W. Hamoen, R.A. Daniel, Two-Step Assembly Dynamics of the Bacillus Divisome, 191(13) (2009) 4186-4194.
[80] A. Müller, M. Wenzel, H. Strahl, F. Grein, T.N.V. Saaki, B. Kohl, T. Siersma, J.E. Bandow, H.G. Sahl, T. Schneider, L.W. Hamoen, Daptomycin inhibits cell envelope synthesis by interfering with fluid membrane microdomains, Proceedings of the National Academy of Sciences of the United States of America 113(45) (2016) E7077-e7086.
